# Iron tris-mesityl: a homoleptic iron(ii) ferrate species for directed C–H activation

**DOI:** 10.1039/d5sc08832a

**Published:** 2026-02-03

**Authors:** Aleksa Radović, Maria C. Healy, Arnadeep Datta, Deborshee Das, Likun Cai, Steven Diaz, Achyut Ranjan Gogoi, Nikki J. Wolford, Stephanie H. Carpenter, William W. Brenessel, David McCamant, Osvaldo Gutierrez, Michael L. Neidig

**Affiliations:** a Department of Chemistry, University of Rochester 120 Trustee Rd Rochester NY14627 USA; b Department of Chemistry, Inorganic Chemistry Laboratory, University of Oxford South Parks Road Oxford OX1 3QR UK; c Department of Chemistry and Biochemistry, University of California, Los Angeles 607 Charles E. Young Drive East Los Angeles CA 90095 USA o.gutierrez@ucla.edu

## Abstract

C–H activation is a vital synthetic tool due to its superior atom economy and improved step efficiency making it amendable to late-stage functionalisation. In recent years iron has been gaining traction within this field due to its high abundance, low cost and low toxicity. Iron(0) phosphines for C–H activation *via* oxidative addition are well documented, however, only a handful of iron(ii) complexes competent at C–H activation *via* ligand-to-ligand hydrogen atom transfer (LLHT) or σ-bond metathesis have been identified. Herein we report the first homoleptic iron species capable of facilitating C–H activation, introducing a new class of well-defined iron(ii) complexes for this purpose, and detail the synthesis and characterisation of a range of tris-cyclometalated iron complexes using a variety of pyridine derived substrates. Density functional theory (DFT) calculations reveal that the C–H activation proceeds through a σ-bond metathesis pathway.

## Abstract 2

The homoleptic organoferrate complex FeMes_3_^−^ is identified as an effective iron(ii) species for directed C–H activation, yielding tris-cyclometalated iron products.

## Graphical abstract

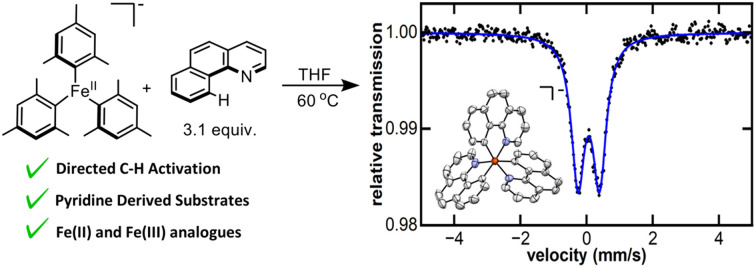

## Introduction

Transition metal-catalysed C–H bond activation is a powerful synthetic strategy for atom and step efficient synthesis across a range of substrates. This field has previously been dominated by precious metal catalysts, such as palladium, where both effective stoichiometric and catalytic reactions are well established.^1–3^ Motivated by the goal to develop sustainable and environmentally friendly synthetic methods, there has been substantial interest in the development of first row transition metal catalysed C–H activation. Iron in particular is a highly attractive candidate for such reactions due to its low cost, limited toxicity and high abundance, yet current systems, including the breadth of iron complexes competent for C–H activation, remain underdeveloped compared to their precious metal analogues.^4–6^

Over the past 50 years, iron catalysts have been highly sought after to activate a variety of C–H bonds as a method of generating Fe–C bonded organometallic species that allow for subsequent functionalisation.^7–11^ Historically, iron-catalysed C–H activation pathways have been classified into two general categories: (1) C–H activation by oxidative addition to a low-valent iron complex to form both Fe–C and Fe–H bonds^10–23^ and (2) σ-bond metathesis ligand-to-ligand hydrogen transfer mechanism (LLHT), commonly employing Fe(ii)–R complexes (R = amide, methyl, *etc.*) where the organic ligand acts as a base to remove the hydrogen from the substrate.^23–31^ The chemistry of low-valent iron has been widely explored for C–H activation over the past few decades, including numerous studies utilising Fe(0)(dmpe)_2_ (dmpe = 1,2-bis(dimethylphosphino)ethane), Fe(0)(dppe)_2_ (dppe = 1,2-bis(diphenylphosphino)ethane) and Fe(0)(PMe_3_)_4_ complexes for both stoichiometric and catalytic C–H activation while more recent examples include Fe(0)–NHC (NHC = N*-*heterocyclic carbene) styrene complexes for stereoselective synthesis.^12–14,32^ Additional approaches include the use of heterobimetallic species such as low-valent Fe–Al and Fe–Na complexes, as well as Fe(0)carbonyl complexes to facilitate C–H bond activation.^7,8,33–35^

**Fig. 1:**
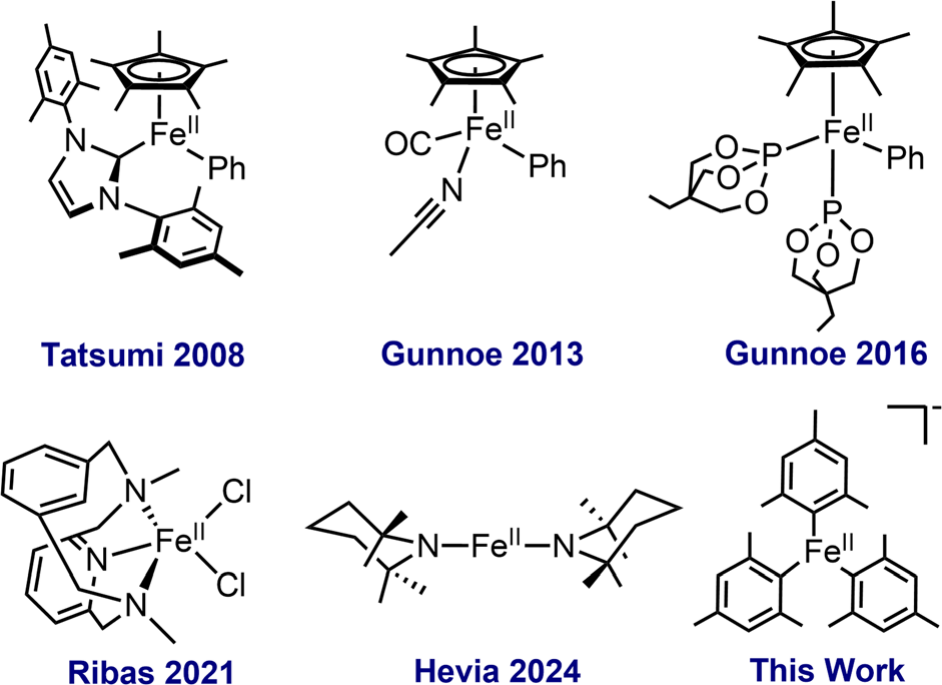
Examples of well-defined Fe(ii) complexes for C–H activation.

By contrast, the development of well-defined Fe(ii)–R complexes capable of C–H activation *via* σ-bond metathesis/LLHT has been less broadly explored. For example, half-sandwich Cp* complexes such as Cp*Fe(ii)PhL_2_ (L = P(OR)_3_) and Cp*Fe(CO)Ph(MeCN) have been demonstrated to C–H activate a variety of heteroaromatic substrates to generate metalated Fe(ii) products in a regioselective manner (Fig. 1).^24–26^ Additional examples include FeMe_2_(PMe_3_)_4_ for the formation of ferracycles from diphenylenimine and thiobenzophenones, as well as Fe(ii) amides that can activate aromatic C–H bonds to form organoiron products.^14,28^ Despite these advances, it is clear that the overall understanding of C–H activation by Fe(ii)–R complexes remains underdeveloped and the identification of new classes of Fe(ii) complexes capable of efficient C–H activation is critical to broadening the synthetic utility of iron C–H activation chemistry.

Over the past decade, several studies have identified the importance of homoleptic organoferrate species in iron-catalysed cross-coupling reactions where they readily activate a variety of C–X bonds (X= Br, Cl).^36–40^ Furthermore, recent mechanistic studies of directed C–H activation have implicated Fe(ii)-aryl intermediates supported by bisphosphine ligands as key species for C–H activation.^41–43^ Thus, we hypothesised that homoleptic Fe(ii) organoferrate complexes would be able to activate stronger C–H bonds, enabling facile directed C–H activation without the need for complex and often expensive supporting ligands commonly employed in C–H activation methods. Herein we demonstrate that [FeMes_3_][MgBr(THF)_5_] (FeMes_3_^−^) is efficient for directed C–H activation, identifying a new class of Fe(ii) complexes for this reaction.

## Results and discussion

### C–H activation of benzo(*h*)quinoline with FeMes_3_^−^

**Fig. 2:**
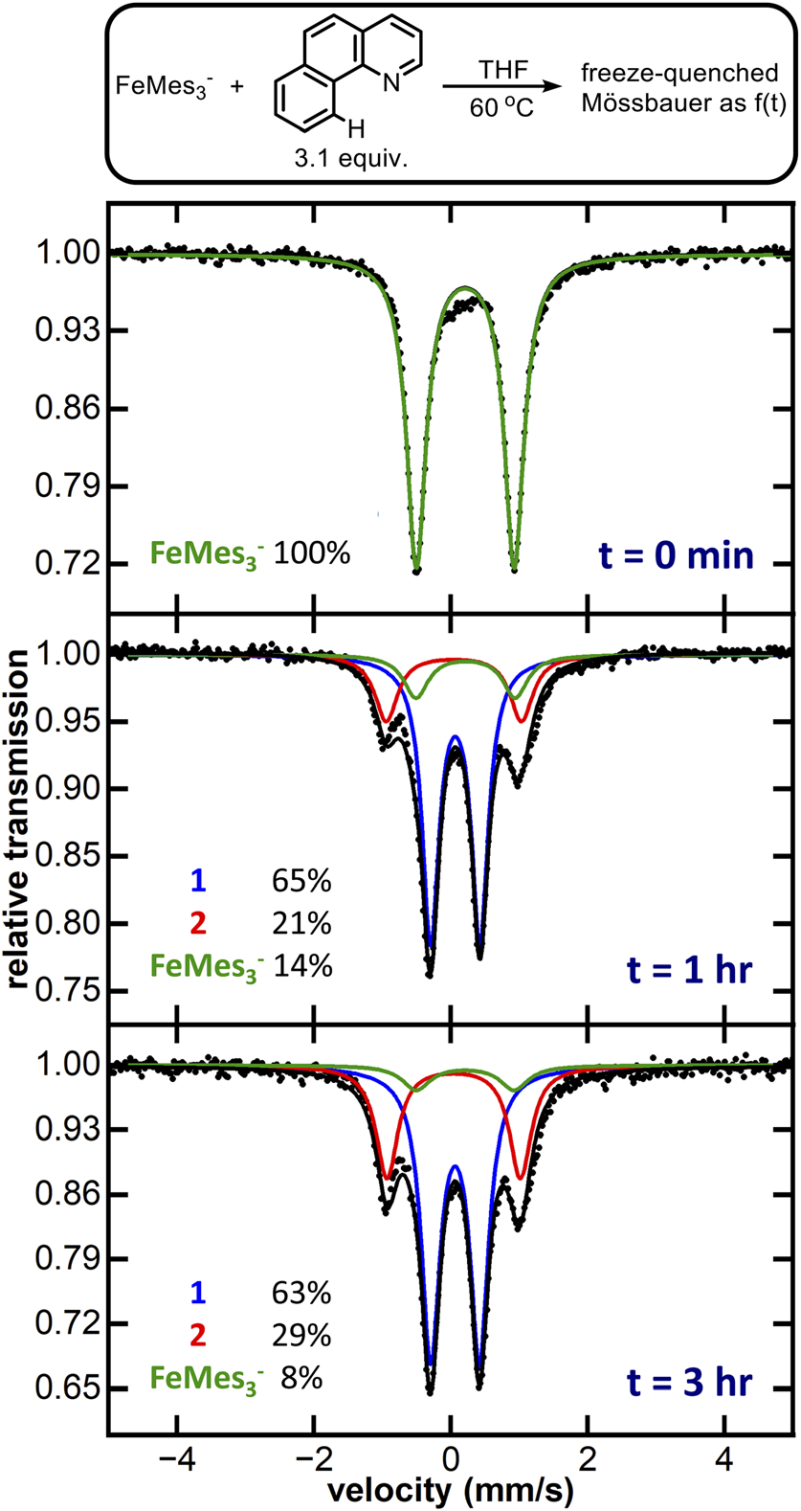
Zero-field, 80 K ^57^Fe Mössbauer spectra of FeMes_3_^−^ reacted with Hbzq at 60 °C. Black dotted trace: raw data. Solid black trace: best total fit. Coloured solid traces: simulation. Blue trace: 1*δ* = 0.06 |Δ*E*_Q_| = 0.71 mm s^−1^. Red trace: 2*δ* = 0.04 |Δ*E*_Q_| = 1.95 mm s^−1^. Green trace: FeMes_3_^−^*δ* = 0.21 |Δ*E*_Q_| = 1.43 mm s^−1^.

We initiated our investigation of homoleptic iron species with FeMes_3_^−^, an easily synthesised and thermally stable homoleptic Fe(ii) organoferrate, probing its C–H activation abilities by reaction of FeMes_3_^−^ (see SI for synthesis and isolation) with 3.1 equiv. of benzoquinoline (Hbzq), a common substrate for directed C–H activation with iron. After 6 hours at room temperature a colour change from pale yellow to dark green was observed. This reaction was investigated *via* 80 K ^57^Fe Mössbauer, with the Mössbauer spectrum revealing only partial conversion of FeMes_3_^−^ to a new iron species 1 (7% conversion, see SI, Fig. S1). Heating the reaction to 60 °C for 1 hour led to the near complete consumption of FeMes_3_^−^ (Fig. 2: green trace, *δ* = 0.21 mm s^−1^, |Δ*E*_Q_| = 1.43 mm s^−1^, 14% of iron) and formation of two new iron species: 1 (Fig. 2: blue trace, *δ* = 0.06 mm s^−1^, |Δ*E*_Q_| = 0.71 mm s^−1^, 65% of iron) and 2 (Fig. 2: red trace, *δ* = 0.04 mm s^−1^, |Δ*E*_Q_| = 1.95 mm s^−1^, 21% of iron). The same two iron species are also present after 3 hours, comprising 92% of total iron. Performing the same reaction using *in situ* generated FeMes_3_^−^ formed from the reaction of FeBr_2_ with 3.1 equiv. of MesMgBr yielded a similar mixture of 1 and 2 (see SI, Fig. S3). Note no reaction of MesMgBr with Hbzq is observed in the control reaction in the absence of iron (see SI, Fig. S19).

**Fig. 3:**
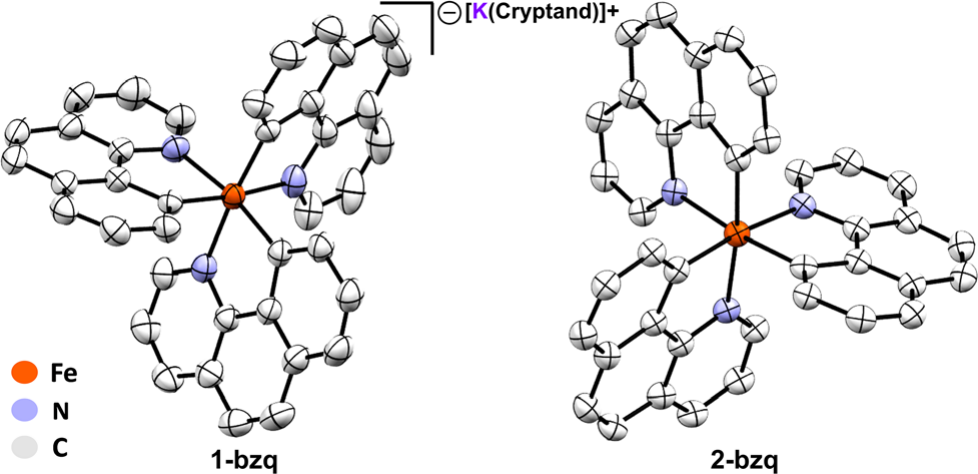
Crystal structures of 1-bzq and 2-bzq as identified by SC-XRD. Hydrogen atoms and potassium 2,2,2-cryptand counterion of 1-bzq omitted for clarity, thermal ellipsoids are shown at 50% probability. Selected average bond lengths (Å) and angles (°): 1-bzq Fe–N 1.9983, Fe–C 1.9515, N–Fe–N 91.90 C–Fe–C 93.05, C–Fe–N_*trans*_ 173.25, C–Fe–N 82.88. 2-bzq Fe–N 2.0511, Fe–C 1.9545, N–Fe–N 90.88 C–Fe–C 94.98, C–Fe–N_*trans*_ 173.02, C–Fe–N 82.62.

The observed reduction in isomer shifts for 1 and 2 compared to FeMes_3_^−^ (*i.e. δ* = 0.06 mm s^−1^ for 1*versus* 0.21 mm s^−1^ for FeMes_3_^−^) was suggestive of a significant ligation change to the iron centre, further corroborated by single crystal X-ray diffraction (SC-XRD). Crystallisation from the reaction mixture afforded two distinct crystals: purple-black needle-shaped crystals identified as the cyclometalated neutral homoleptic Fe(iii) tris-(benzoquinolinyl) complex Fe(bzq)_3_ (2-bzq) (Fig. 3) and green-black needle shaped crystals tentatively identified by SC-XRD as the Fe(ii) tris-(benzoquinolinyl) complex, [Fe(bzq)_3_][MgBr(THF)_5_] however, crystals were poorly diffracting therefore no detailed discussion of the bond metrics is provided. Observation of these two iron species is consistent with the species identified in the reaction by 80 K ^57^Fe Mössbauer spectroscopy, where the component with the higher isomer shift and smaller quadrupole splitting (Fig. 2: blue trace, *δ* = 0.06 mm s^−1^, |Δ*E*_Q_| = 0.71 mm s^−1^) can be attributed to the Fe(ii) complex, while the second component (Fig. 2: red trace, *δ* = 0.04 mm s^−1^, |Δ*E*_Q_| = 1.95 mm s^−1^) can be attributed to the Fe(iii) complex 2-bzq. The ^13^C{^1^H} NMR of the post reaction mixture confirmed the presence of mesitylene as the only organic by-product (see SI, Fig. S18).

**Fig. 4:**
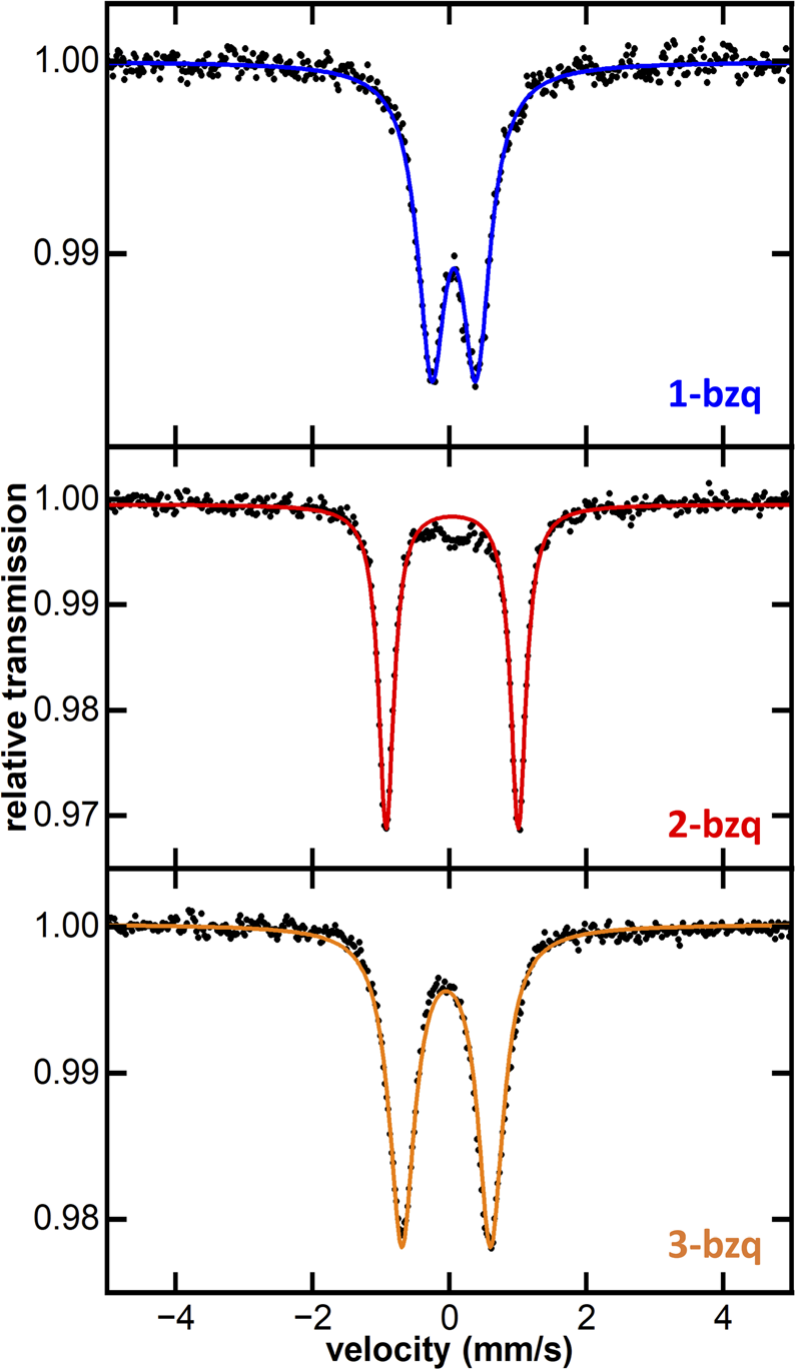
Zero-field, solid 80 K ^57^Fe Mössbauer spectra of isolated complexes 1-bzq, 2-bzq and 3-bzq. Black dotted trace: raw data. Coloured solid traces: simulation. 1-bzq*δ* = 0.06 |Δ*E*_Q_| = 0.65 mm s^−1^. 2-bzq*δ* = 0.04 |Δ*E*_Q_| = 1.95 mm s^−1^. 3-bzq*δ* = −0.05 |Δ*E*_Q_| = 1.30 mm s^−1^.

To isolate substantial quantities of pure C–H activation products for characterisation, and to obtain a crystal structure of the Fe(ii) tris-(benzoquinolinyl) complex, we sought to first isolate the Fe(iii) complex, allowing for successive quantitative reduction to the Fe(ii) complex. *In situ* formed FeMes_3_^−^ was reacted with Hbzq at 60 °C for 12 hours. Exposure of the reaction mixture to dry air at room temperature resulted in generation of the oxidised product 2-bzq in 85% isolated yield. The 80 K ^57^Fe Mössbauer spectrum of solid 2-bzq shows parameters of *δ* = 0.04 mm s^−1^ and |ΔEQ| = 1.95 mm s^−1^ (Fig. 4, red trace), identical to those observed *in situ* for product 2 and further supporting its assignment as the C–H activated product 2-bzq. The 10 K EPR spectrum of 2-bzq exhibits an axial signal with *g*_‖_ = 2.47 and *g*_⊥_ = 1.84 (see SI, Fig. S6). The distribution of the *g* values indicates localisation of the unpaired electron on the metal d-orbital with little to no ligand radical character.

Subsequent reduction of isolated 2-bzq with 1 equiv. of KC_8_ and 2,2,2-cryptand in acetonitrile (MeCN) at room temperature gave the reduced complex [Fe(bzq)_3_][K(2,2,2-cryptand)] (1-bzq) in 56% isolated yield. Crystals suitable for SC-XRD were obtained by vapour diffusion with diethyl ether, verifying the structure of 1-bzq as the Fe(ii) ate analogue of 2-bzq (Fig. 3). The 80 K ^57^Fe Mössbauer spectrum of solid 1-bzq is characterised by *δ* = 0.06 mm s^−1^ and |Δ*E*_Q_| = 0.65 mm s^−1^ (Fig. 4, blue trace), consistent with the assignment of *in situ* formed 1 as the Fe(ii) C–H activated product 1-bzq. The ^1^H NMR spectrum of 1-bzq did not display any broadened or paramagnetic signals associated with paramagnetic iron species, indicating that 1-bzq is a diamagnetic low-spin Fe(ii) complex (see SI, Fig. S20). Overall, these results suggest that 1-bzq is initially formed from three successive C–H activations of FeMes_3_^−^ and is subsequently oxidised to 2-bzq by trace oxygen *in situ*, consistent with facile oxidation due to the increased σ-donor properties of these cyclometalating ligands. This coincides with cyclic voltammetry (CV) studies carried out on 1-bzq in MeCN *vs.* a ferrocene standard (see SI, Fig. S7) which indicated the presence of two reversible redox couples with *E*_1/2_ = −1.66 V and −0.34 V *vs.* Fc/Fc^+^ assigned to Fe(ii)/Fe(iii) and Fe(iii)/Fe(iv) redox couples respectively. The cyclic voltammogram of 2-bzq in DCM *vs.* Fc/Fc^+^ yielded similar results with two reversible redox couples observed with *E*_1/2_ = −1.83 V and −0.48 V *vs.* Fc/Fc^+^, again attributed to Fe(ii)/Fe(iii) and Fe(iii)/Fe(iv) respectively (see SI Fig. S8).

Given that the CV studies indicated a reversible and accessible Fe(iii)/Fe(iv) couple, we attempted to complete an isostructural series comprising Fe(ii), Fe(iii) and Fe(iv) analogues, by reacting 2-bzq with 1 equiv. of cerium ammonium nitrate at room temperature in MeCN, successfully generating the Fe(iv) tris-cyclometalated analogue 3-bzq (SC-XRD in SI, Fig. S42). The 80 K ^57^Fe Mössbauer spectrum of solid 3-bzq has parameters of *δ* = −0.05 mm s^−1^ and |Δ*E*_Q_| = 1.30 mm s^−1^ (Fig. 4, orange trace). The decreased isomer shift of 3-bzq compared to 2-bzq is consistent with the formation of a paramagnetic *S* = 1 Fe(iv) complex.

### Directed C–H activation with FeMes_3_^−^

**Fig. 5:**
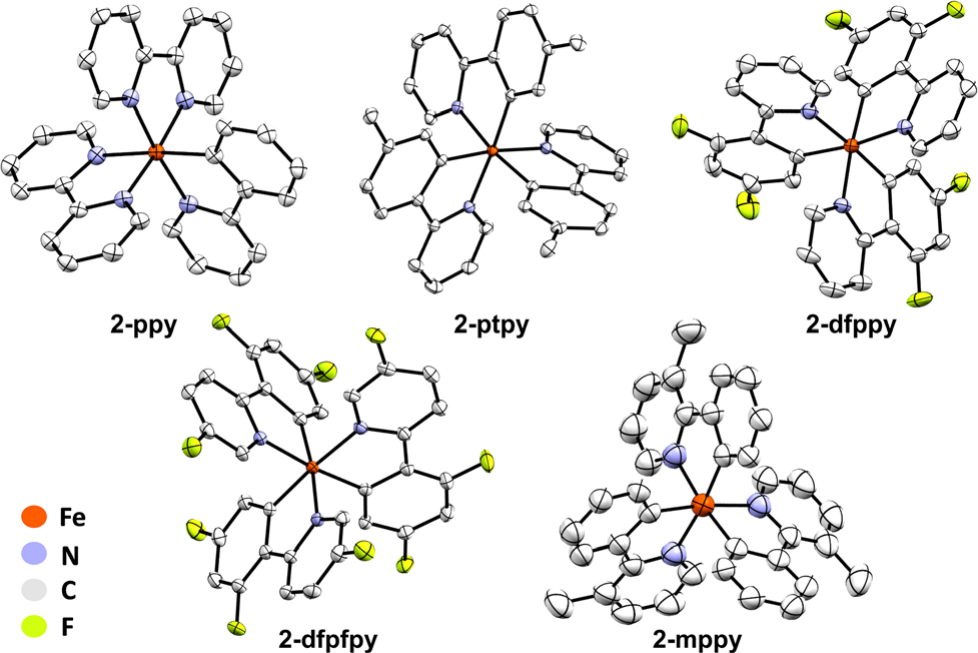
Crystal structures of Fe(iii) complexes 2-ppy, 2-ptpy, 2-dfppy, 2-dfpfpy and 2-mppy as identified by SC-XRD. Hydrogen atoms and solvent molecules omitted for clarity. Thermal *ellipsoids* are shown at 50% probability. Selected average bond lengths (Å) and angles (°): 2-ppy Fe–N 2.0090, Fe–C 1.9780, N–Fe–N 95.62, C–Fe–C 93.76, C–Fe–N_*trans*_ 174.76, C–Fe–N 81.68. 2-ptpy Fe–N 2.0357, Fe–C 1.9529, N–Fe–N 93.80, C–Fe–C 93.55, C–Fe–N_*trans*_ 173.98, C–Fe–N 82.15. 2-dfppy Fe–N 2.0252, Fe–C 1.9500, N–Fe–N 94.38 C–Fe–C 93.88, C–Fe–N_*trans*_ 174.93, C–Fe–N 82.04. 2-dfpfpy Fe–N 2.0223, Fe–C 1.9490, N–Fe–N 94.48, C–Fe–C 93.74, C–Fe–N_*trans*_ 174.49, C–Fe–N 82.03. 2-mppy Fe–N 2.0360, Fe–C 1.9436, N–Fe–N 94.48, C–Fe–C 94.84, C–Fe–N_*trans*_ 173.44, C–Fe–N 80.85.

Having established that FeMes_3_^−^ can C–H activate Hbzq, we next probed the viability of this synthetic procedure with a wider set of pyridine-derived directed substrates. 2-Phenylpyridine (Hppy), 2-(*p*-tolyl)pyridine (Hptpy) and 2-(2,4-difluorophenyl)pyridine (Hdfppy) were selected to evaluate the influence of the ligand π system and electron donating/withdrawing groups on C–H activation by FeMes_3_^−^. Fe(iii) products were targeted using subsequent treatment of C–H activated product solution with dry air as previously described. Utilising this procedure, tris-cyclometalated Fe(iii) complexes Fe(ppy)_3_ (2-ppy), Fe(ptpy)_3_ (2-ptpy), and Fe(dfppy)_3_ (2-dfppy) were also synthesised and structurally characterised by SC-XRD (Fig. 5). The 10 K EPR spectra of complexes 2-ppy, 2-ptpy, and 2-dfppy indicated that all of the Fe(iii) tris-cyclometalated products are low-spin *S* = 1/2 complexes based upon the observed axial EPR spectra and associated *g*-values observed (and further supported by ^57^Fe 80 K Mössbauer spectroscopy) (Table 1).

**Table 1:** 80 K ^57^Fe Mössbauer and 10 K EPR parameters of Fe(iii) tris-cyclometalated complexes

Complex	Mössbauer parameters		EPR parameters	
	*δ* (mm s^−1^)	|Δ*E*_Q_| (mm s^−1^)	*g*_‖_	*g*_⊥_
2-bzq	0.04	1.93	2.47	1.84
2-ppy	−0.01	1.82	2.42	1.90
2-ptpy	0.01	1.82	2.46	1.87
2-dfppy	−0.01	1.83	2.46	2.00

2-ppy, 2-ptpy and 2-dfppy could be easily reduced with potassium graphite at room temperature to generate the Fe(ii) analogues 1-ppy, 1-ptpy, and 1-dfppy as confirmed by SC-XRD ^57^Fe 80 K Mössbauer spectroscopy (see SI, Fig. S38–S41).

3-Methyl-2-phenylpyridine (Hmppy) and 2-(2,4-difluorophenyl)-5-fluoropyridine (Hdfpfpy) could also be employed to isolate crystals of Fe(mppy)_3_ (2-mppy) and Fe(dfpfpy)_3_ (2-dfpypy) for SC-XRD analysis (Fig. 5), though the use of these substrates gave very poor synthetic yields that did not permit further characterization or reduction attempts of the product complexes.

### Mechanism of directed C–H activation by FeMes_3_^−^

The ability of FeMes_3_^−^ to efficiently perform directed C–H activation of Hbzq (>90% solution yield of tris-cyclometalated products identified by freeze-trapped 80 K ^57^Fe Mössbauer spectroscopy) represents a new reactivity for this simple homoleptic ferrate. While the observation of mesitylene as the organic product of this reaction is suggestive of C–H activation *via* σ-bond metathesis or a ligand-to-ligand hydrogen transfer (LLHT) mechanism, we sought to further explore the intermediates involved in the formation of 1-bzq. As three successive C–H activations are required to form this product, we first sought to identify the iron product after the first C–H activation. To achieve this, FeMes_3_^−^ was reacted with 1 equiv. of Hbzq at 60 °C in THF (the previously identified reaction conditions for C–H activation). 80 K ^57^Fe Mössbauer spectroscopy of the reaction freeze-trapped after 3 hours contained both tris-cyclometalated product 1-bzq (27% of total iron by Mössbauer) and unreacted FeMes_3_^−^ (63% of total iron by Mössbauer) (see SI, Fig. S2). This result is consistent with the first C–H activation being the rate determining step of the three C–H activation steps involved in formation of 1-bzq, and resulting in no iron intermediates after the first and second C–H activations being observable *in situ*.

**Fig. 6:**
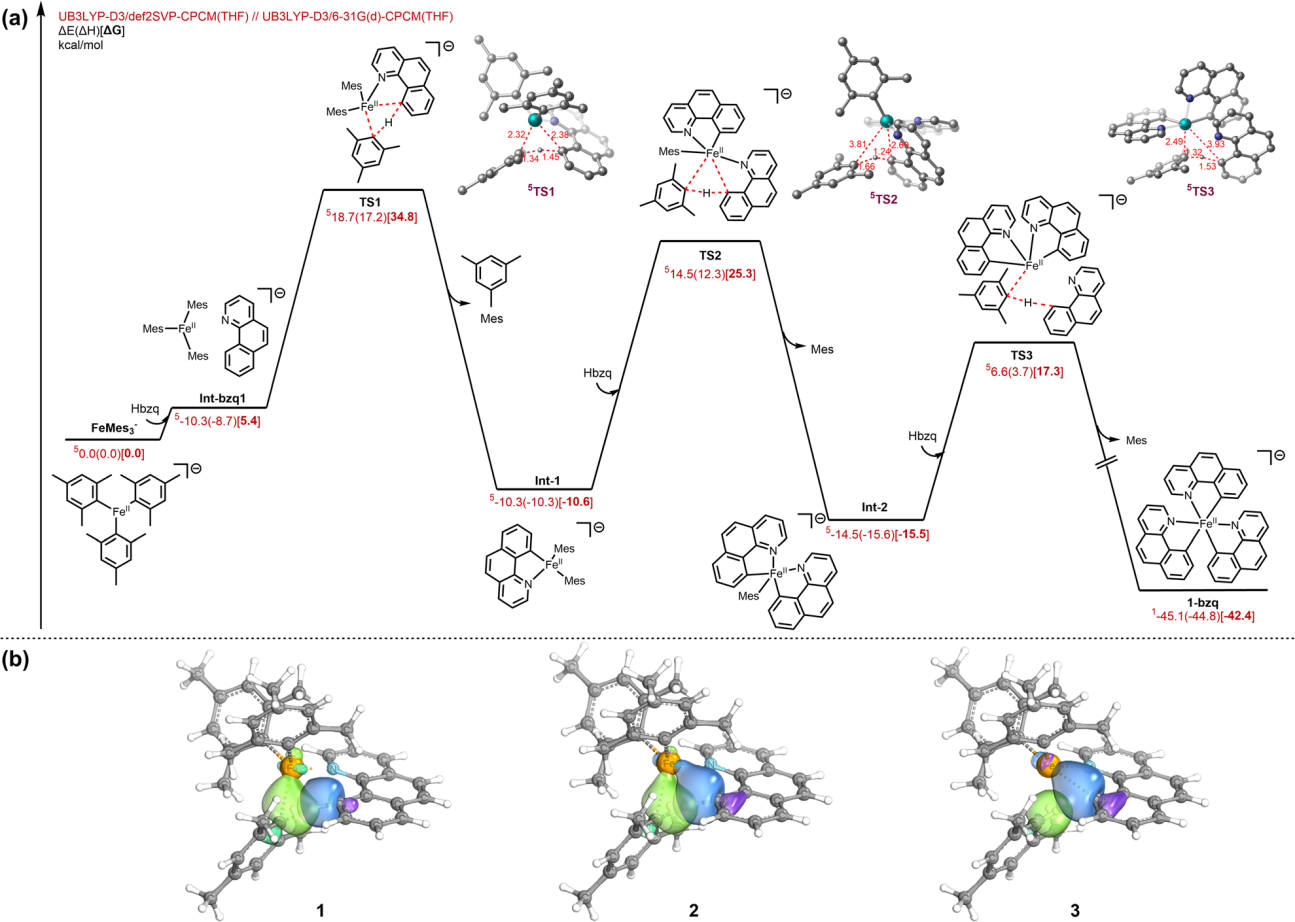
(a) Computational investigation into the full sp^2^ C–H activation pathway with associated transition states (TS1, TS2 & TS3) in lowest energy spin state. (b) Intrinsic bond orbital analysis of the associated σ-bond metathesis sp^2^ C–H activation step, 1 (stationary point from backward IRC of TS1), 2 (TS1), and 3 (stationary point from forward IRC of TS1). Multiplicities are in superscripts.

To probe the mechanistic manifold of this process, we employed dispersion-corrected density functional theory (DFT) at the UB3LYP-D3/def2-SVP-CPCM(THF)//UB3LYP-D3/6-31-G(d)-CPCM(THF) level of theory (Fig. 6a). The calculations indicate that FeMes_3_^−^ and Hbzq initially associate to form Int-bzq1 that allows for subsequent C(sp^2^)–H activation of Hbzq *via* transition state TS1 with an energy barrier of 29.4 kcal mol^−1^ (with respect to Int-bzq1 from IRC calculations), yielding the intermediate Int-1. This intermediate undergoes subsequent C–H activation *via*TS2 and TS3 to afford the final product 1-bzq (Fig. 6). The second C–H activation barrier *via*TS2 is comparable with the initial C–H activation *via*TS1. The third C–H activation barrier *via*TS3 is notably lower than both the first and second C–H activation steps. Each C–H activation step generates a progressively lower energy intermediate from the tri-coordinated FeMes_3_^−^ complex. Thus, we hypothesize that the formation of the more favourable octahedral complex 1-bzq is thermodynamically driven. Previous examples of similar C–H activation systems are reported to proceed *via* a σ-bond metathesis mechanism^6,43,44^ or LLHT where the iron centre is participating in the hydride transfer process.^31^ To differentiate between a LLHT process and a σ-bond metathesis mechanism, we performed intrinsic bond orbital (IBO) analysis (Fig. 6b) to trace the electron flow in TS1 from 1 (stationary point from backward IRC of TS1), 2 (TS1), and 3 (stationary point from forward IRC of TS1).

The analysis reveals the concerted participation of four distinct σ-bonds (mesityl_C–H, Hbzq_C–H, Fe–C_mesityl, and Fe–C_Hbzq) in the transition state. Crucially, unlike oxidative addition, the electron pair in the Fe–C_mesityl σ-bond is utilized to accommodate the transferring proton effectively forming a new mesityl_C–H bond rather than involving electron density from the iron d-orbitals. In this instance, IBO analysis characterizes the mechanism as a formal [2σ + 2σ] σ-bond metathesis process,^45^ rather than metal d-assisted σ-bond metathesis or LLHT mechanism. Therefore, while the Fe–N interaction is crucial for achieving the appropriate geometry required for the C–H activation transition state, the iron centre itself does not exhibit direct orbital involvement in the hydride transfer at the transition state.

### Excited state properties of tris-cyclometalated iron complexes

Beyond the synthetic impact of identifying FeMes_3_^−^ as a new complex for directed C–H activation reactions, the tris-cyclometalated iron products accessible in this reaction are also of interest with respect to the field of iron photoredox catalysis. There has been broad interest in the photophysics and excited state lifetimes of cyclometalated iron complexes across a variety of iron oxidation states. Therefore, we employed ultrafast transient absorption (TA) spectroscopy to probe the excited state properties of 1-bzq, 2-bzq and 3-bzq as representative examples of this class of tris-cyclometalated products. In addition to characterising the excited-state lifetimes of these species, the comparison of the transient spectra to those of the oxidised or reduced species can help identify the absorption band as either MLCT or LMCT in character.

**Fig. 7:**
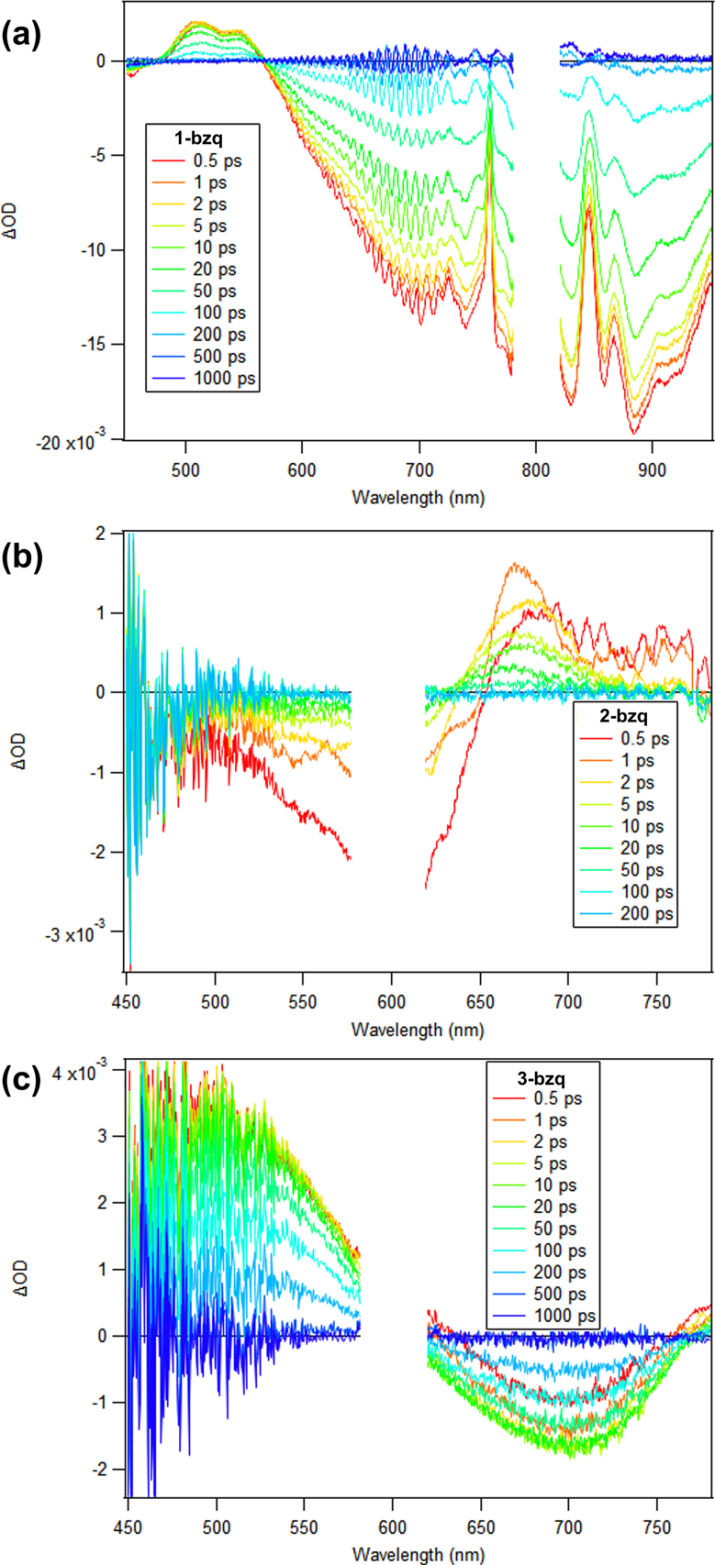
(a) Transient absorption spectra of 1-bzq in MeCN. (b) Transient absorption spectra of 2-bzq in DCM. (c) Transient absorption spectra of 3-bzq in MeCN. 2-bzq and 3-bzq were excited at 600 nm and 1-bzq was excited at 800 nm. The sharp features from 700–900 nm in (a) are artifacts from the spectral shape of the white-light continuum.

The excited state absorption spectrum of 1-bzq in MeCN (800 nm excitation to match the lowest energy charge transfer transition for the complex) shows an excited state absorption feature around 550 nm, while broad ground state bleach can be observed between 600 and 1000 nm (Fig. 7a). The TA kinetics can be fit to two exponential functions with 3 ps excited-state reorganization process and a 56 ps excited state lifetime (Fig. S27, Table S1). Excitation to the CT band should lead to the formation of a ^1^MLCT state which should rapidly undergo intersystem crossing to ^3^MLCT state, where absorption of this state should resemble the absorption spectrum of the oxidised, Fe(iii) complex. Comparison of the excited state spectrum with the difference spectrum of the Fe(iii)–Fe(ii) complexes (Fig. S16) reveals good agreement between difference spectrum and longer-lived component, which suggests that longer lived excited state corresponds to the ^3^MLCT state. Additionally, the lack of any long-lived bleach in Fig. 7a indicates that 1-bzq does not relax into a long-lived low-energy high spin state as [Fe^II^(bpy)_3_]^2+^ does.^46^

The excited state absorption (ESA) spectrum of 2-bzq in DCM (600 nm excitation corresponding to the lowest energy charge- transfer transition of the complex) shows only weak features around 670–760 nm, just to longer wavelengths relative to the ground-state absorption band, while broad ground-state bleach (GSB) can be observed between 550 and 650 nm (Fig. 7b). The dynamics can be fit with excited state lifetime of 0.33 ps (see SI, Fig. S27). Then, on the 10 ps time scale we observe the vibrational cooling of a hot ground-state species created by the ultrafast internal conversion to the ground state, which is apparent in the gradual shift of the peak of the ESA band from 670 to 655 nm on this time scale. Excitation to the CT band should lead to the formation of either a ^2^MLCT or a ^2^LMCT state, where absorption of this state should resemble the absorption spectrum of the oxidized Fe(iv) complex (for the MLCT transition) or the reduced, Fe(ii) complex (for the LMCT transition). Comparison of the excited state spectrum with the difference spectrum of the Fe(iv) and Fe(iii) complexes and the Fe(iii) and Fe(ii) complexes reveals better agreement with the Fe(iv)–Fe(iii) difference spectrum which suggests that the observed excited state likely corresponds to the ^2^MLCT state (see SI, Fig. S15–S18). While both difference spectra would predict a broad ESA from 650–800 nm, only the Fe(iv)–Fe(iii) difference predicts a significant bleach from 450–650 nm. Such short excited state lifetime is significantly lower compared to the Fe(iii) complexes with NHC based ligands, which exhibit excited state lifetimes of 100 ps for [Fe(btz)_3_]^3+^ and 2 ns for [Fe(phtmeimb)_2_]^+^. This suggests that in the case of the 2-bzq complex the energy difference between excited CT and an excited metal centred state is not sufficient to suppress non-radiative decay from this state.

Lastly, the excited state absorption spectrum of 3-bzq shows a broad ESA feature spanning <450 nm to 600 nm, while a broad GSB can be observed from 620–760 nm (Fig. 7c). The excited state kinetics are fit to reveal an excited state lifetime of ∼161 ps (see SI, Fig. S27). Excitation to the Fe(iv) CT band should lead to the formation of ^3^LMCT state, where absorption of this state should resemble the absorption spectrum of the Fe(iii) complex. The excited state spectrum and difference spectrum of Fe(iv)–Fe(iii) complexes (see SI, Fig. S14) are in good agreement suggesting that observed excited state corresponds to the ^3^LMCT state. This excited charge transfer state is significantly longer lived compared to the Fe(ii) and Fe(iii) complexes, indicating that Fe(iv) complexes may be good alternative to Fe(ii) and Fe(iii) complexes in photochemical applications. Notably, it is significantly longer lived compared to the previously characterised homoleptic Fe(iv) complexes (0.8 ps for [Fe(phtmeimb)_2_]^2+^ and 1.4 ps for [Fe(ImP)_2_]^2+^).^47,48^

A complete transient absorption characterisation of the excited state lifetimes of 1-ppy, 2-ppy, 1-ptpy, 2-ptpy, 1-dfppy and 2-dfppy is presented in the SI (Fig. S26, S28 and S29).

## Conclusions

In this work, a new Fe(ii) species competent at directed C–H activation has been identified allowing for the development of a simple synthetic method using easily accessible iron salt, MesMgBr and a pyridine derived ligand precursor for directed C–H activation. DFT calculations revealed that transition state for the C–H activation process goes through a formal [2σ + 2σ] σ-bond metathesis pathway without any interaction with the iron centre. It is demonstrated that this method enables access to a wide variety of tris-cyclometalated iron complexes that have historically been synthetically challenging to access. This new class of iron complexes for C–H activation may enable new routes to functionalised products using sustainable iron catalysts.

## Author contributions

A. R., M. C. H., N. J. W. and S. H. C. provided the experimental/spectroscopic data. A. D., D. D., A. R. G. and O. G. provided the computational data. A. D. and D. D. contributed equally to the computational studies. L. C. and S. D. collected and analysed transient absorption data. M. L. N., O. G. and D. McC. supervised this work. A. R., M. C. H. and M. L. N. prepared the original draft, all authors contributed to subsequent drafts.

## Conflicts of interest

There are no conflicts to declare.

## Supplementary Material

SC-017-D5SC08832A-s001

SC-017-D5SC08832A-s002

## Data Availability

The data supporting the findings of this study are available within the paper and its supplementary information (SI). Supplementary information: synthetic and experimental procedures, additional spectroscopic data, SC-XRD data and DFT calculations. See DOI: https://doi.org/10.1039/d5sc08832a.
