# Supplementary material for: Iron tris-mesityl: a homoleptic iron(ii) ferrate species for directed C–H activation
Source: Chem Sci. 2026 Feb 3;17(14):7002–9. doi: 10.1039/d5sc08832a (PMC12896185; doi:10.1039/d5sc08832a)
Supplement: SC-017-D5SC08832A-s001 [file SC-017-D5SC08832A-s001.pdf]

Supplementary Information (SI) for Chemical Science.
This journal is © The Royal Society of Chemistry 2026

This version of the Supplementary Information, published 19 February 2026, replaces the version published previously on 3 February 2026.

Supporting Information for

Iron Tris-Mesityl: A Homoleptic Iron(II) Ferrate Species for Directed C-H Activation

Aleksa Radović,[a†] Maria C. Healy,[b†] Arnadeep Datta,[c] Deborshee Das,[c] Likun Cai,[a] Steven Diaz,[a] Achyut Ranjan Gogoi,[c] Nikki J. Wolford,[a] Stephanie H. Carpenter,[a] William W. Brenessel,[a] David McCamant[a*], Osvaldo Gutierrez[c*] and Michael L Neidig[b*]

Table of Contents

1. **Experimental Procedures** S3
2. **Mössbauer Spectra** S8
3. **EPR Spectra** S12
4. **Cyclic Voltammograms** S13
5. **UV-Vis Spectra** S15
6. **NMR Spectra** S19
7. **Transient Absorption Spectra and Kinetics.** S23
8. **Single Crystal X-Ray Diffraction** S28
9. **Density Functional Theory** S43
10. **References** S136

1. Experimental Procedures

1.1 General Considerations – All solvents and chemicals were purchased from commercial sources. All air and moisture sensitive manipulations were carried out in an MBraun inert-atmosphere (N_2) dry box equipped with a direct liquid nitrogen inlet line. All anhydrous solvents were further dried using activated alumina/4 Å molecular sieves and stored under N_2-atmosphere over 4 Å molecular sieves. NMR spectra were recorded at 400 MHz on a Bruker DPX-400 spectrometer.

1.2 ^{57}Fe Mössbauer Spectroscopy – All samples were prepared in an N_2 atmosphere dry glovebox equipped with a liquid N_2 fill port to enable sample freezing to 77 K. Each sample was loaded into a Mössbauer sample cup manufactured from Delrin and stored, handled, and loaded under liquid N_2. Low-temperature ^{57}Fe Mössbauer measurements were performed using a See Co. MS4 Mössbauer spectrometer integrated with a Janis SVT-400T He/N_2 cryostat for measurements at 80 K. Isomer shifts were determined relative to α-Fe at 298 K. All Mössbauer spectra were fit using the program WMoss (SeeCo). Errors of the fit analyses were the following: $\delta \pm 0.02$ mm/s and $\Delta E_Q \pm 3\%$. For multicomponent fits, the quantitation errors of individual components were ± 3%.

1.3 Electron Paramagnetic Resonance (EPR) Spectroscopy – All samples for EPR spectroscopy were prepared in an N_2 atmosphere glove box equipped with a liquid N_2 fill port to enable sample freezing to 77 K. EPR samples were prepared in 4 mm OD suprasil quartz EPR tubes from Wilmad Labglass. X-band EPR spectra were recorded on a Bruker EMXplus spectrometer equipped with a 4119HS cavity and an Oxford ESR-900 helium flow cryostat for measurements at 10 K. The instrumental parameters employed for all samples were as follows: 1 mW power; modulation amplitude 8 Gauss; frequency ≈ 9.38199 GHz; modulation frequency 100 kHz. Simulation of the EPR spectra was performed using EasySpin.[1]

1.4 Cyclic Voltammetry – Cyclic voltammetry (CV) was carried out in the glovebox under inert conditions with EMStat4s. Electrodes: Working – glassy carbon. Counter – platinum wire. Pseudo reference: Silver wire. Reference – Ag/AgCl (leakproof). Electrolyte - $[NBu_4][PF_6]$. Solvent – MeCN or DCM.

1.5 UV-Vis-NIR Spectroscopy – Spectra were collected using a Cary 6000i UV-Vis-NIR spectrometer fitted with a Unisoku cryostat in an air-tight 1 cm quartz cuvettes. Samples for TA spectroscopy measurements were measured before and after experiment in 1 or 2 mm air-tight quartz cuvettes.

1.6 Transient absorption (TA) spectroscopy – For transient absorption measurements, an 800 nm 1-kHz femtosecond laser system (Spectra Physics Spitfire Pro) was used to generate both broadband probe and actinic pump pulses. The broadband probe pulse was generated by focusing a portion of the 800 nm fundamental through either a sapphire or calcium fluoride crystal. The sapphire continuum provided a probe from approximately 480 to 1000 nm and the calcium fluoride provided a probe from approximately 320 nm to 900 nm. The reader will notice increased noise at the edges of the probe spectrum, where there were limited detector counts. When using a calcium fluoride crystal to generate a broadband probe, the crystal was translated continuously to ensure a stable continuum generation. The 800 nm actinic pump was used directly from the output of the amplifier with no further compression. A 600 nm actinic pump was made by using a home-build non-collinear optical parametric amplifier (NOPA) and compressed using a prism compressor. A 400 nm pump was made by frequency doubling the 800 nm fundamental using a BBO crystal; the 400 nm pump was used without further compression. Pump pulse energy was varied from 0 to 200 nJ/pulse, to confirm the kinetics were independent of pump power and the pump beam was

focused to a 40-60 micron beam diameter at the sample. Every other pump pulse was blocked using an optical chopper while every probe pulse was dispersed using a monochromator (Acton, 300 mm fl, 150 gr/mm) and collected onto a CCD (Princeton Instruments, Pixis 100BR). Samples were contained in 1 or 2 mm air-tight quartz cuvettes and translated continuously to prevent photodamage. Transient absorption scans were collected by averaging 1000 pump-on:pump-off spectra per time point using LabView (National Instruments) and data processing was performed in Igor Pro 8 (Wavemetrics). Additionally, UV-visible absorption spectra were collected of each sample before and after irradiation, and no photodamage to the sample was observed.

1.7 Single crystal X-ray diffraction (SC-XRD) – A crystal was placed onto a thin glass optical fiber or a nylon loop and mounted on a Rigaku XtaLAB Synergy-S Dualflex diffractometer equipped with a HyPix-6000HE HPC area detector for data collection at specified temperature for all cases except $[FeMes_3]^-[MgBr(THF)_5]^+$. A preliminary set of cell constants and an orientation matrix were calculated from a small sampling of reflections.[2] A short pre-experiment was run, from which an optimal data collection strategy was determined. The full data collection was carried out using a PhotonJet (Cu) X-ray source. The structure was solved using SHELXT[3] and refined using SHELXL.[4] The space group was determined based on systematic absences and intensity statistics. Most or all non-hydrogen atoms were assigned from the solution. Full-matrix least squares/difference Fourier cycles were performed which located any remaining non-hydrogen atoms. All non-hydrogen atoms were refined with anisotropic displacement parameters. All hydrogen atoms were placed in ideal positions and refined as riding atoms with relative isotropic displacement parameters. Structure manipulation and figure generation were performed using Olex2.[5] Single-crystal X-ray diffraction data of $[FeMes_3]^-[MgBr(THF)_5]^+$ was collected on an Oxford Diffraction/Agilent SuperNova diffractometer equipped with a 135 mm Atlas CCD area detector. The crystal was selected under a nitrogen atmosphere, mounted on a MicroMesh sample pin and cooled using an Oxford Cryosystems open flow N2 cooling device. Data was collected at 150 K using mirror monochromated Cu K(α) radiation (wavelength = 1.5418 Å; Oxford Diffraction Supernova). Data was then processed using CrysAlisPro package,[6] structures solved ab initio from the integrated intensities using SHELXT[3] and refined using SHELXL[4] with the graphical interface OLEX2.[5]

1.8 Density functional theory (DFT) calculations – DFT calculations were carried out using Gaussian 16 (Rev.A03)[7] for structure optimizations, frequency and TDDFT calculations, while ORCA software (v 4.2.1)[8], was used for calculation of ^{57}Fe Mössbauer parameters. Optimized structures were calculated using B3LYP[9-12] functional along with atom-pairwise dispersion correction with the Becke-Johnson damping, D3BJ[13] and def2-TZVP[14] basis set on all atoms. Experimentally obtained crystal structures were used as starting point for geometry optimizations. Charged complexes were optimized without counter-ion. To account for solvent effects PCM[15-17] solvent model was used. Frequency calculations were performed to ensure that the stationary points were true energy minima. TDDFT calculations were performed utilizing hybrid TPSSh[18, 19] functional, considering contributions from lowest 50 excited states. Contributions to the excited state transitions were calculated using Multiwfn[20] software. The ^{57}Fe Mössbauer parameters were calculated using core polarized CP(PPP)[21] basis set for Fe and def2-TZVP for all other atoms. Scalar relativistic effects were included through second-order Douglas–Kroll–Hess approximation.[22] Isomer shifts were calculated from electron densities at the Fe nucleus using a previously reported procedure.[23, 24]

1.9 Synthesis of Complexes

1.9.1 Synthesis of $FeMes_3^-$ - $FeMes_3^-$ was prepared using a modified preparation based upon previously reported syntheses of a similar Fe(II) homoleptic ferrate species.[25] A 14 mL scintillation vial was charged with $FeBr_2$ (17 mg, 0.078 mmol) and THF (4 mL) Mesityl magnesium bromide solution (0.50 mL, 1M in THF, 0.50 mmol) was added dropwise. The solution was stirred at room temperature for 5 minutes, then 2 mL of Et_2O was added. The solution was filtered over celite and stored in the glovebox freezer at -30 °C. After two days, colourless needle-shaped crystals suitable for SC-XRD were formed (33 mg, 48% yield). **^{57}Fe Mössbauer parameters (solid):** δ = 0.21 mm/s, $|\Delta EQ|$ = 1.43 mm/s. **^{1}H NMR (400 MHz, MeCN-*d_3*):** δ= 127.60 (s, 6H, *meta*-CH), 111.67 (s, 9H, *para*-CH_3), 23.31 (s, 18H, *ortho*-CH_3), 3.62(s, THF), 1.77 (s,THF).

1.9.2 Preparation of Tris-cyclometalated Fe(III) complexes

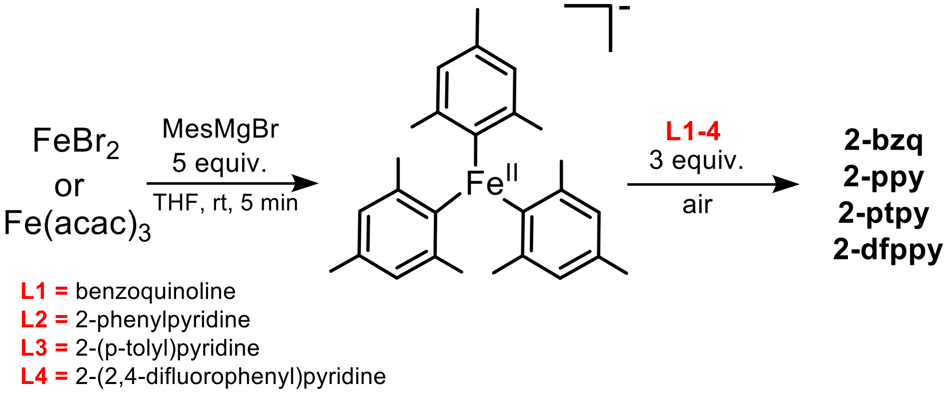

Scheme 1. General method for the preparation of tris-cyclometalated Fe(III) complexes using either $FeBr_2$ or $Fe(acac)_3$.

1.9.2.1 General Method A - A 14 mL scintillation vial fitted with a teflon stir bar was charged with $Fe(acac)_3$ (132.0 mg, 0.372 mmol), **L1-4** (1.120 mmol), and 4 mL of THF. After stirring the mixture at RT for 10 min, MesMgBr (2240 µL, 1.0 M in THF, 2.24 mmol) was added dropwise to the reaction mixture at RT. The resulting pale yellow solution was stirred for 1 h at RT. The vial was removed from the glovebox and exposed to dry air. The reaction mixture was left in the glovebox for 2 days, yielding crystals suitable for SC-XRD. Formed solids were washed with MeCN (**2-bzq**) or MeOH (**2-ppy**, **2-ptpy**, **2-dfppy**) prior to further use.

1.9.2.2 General Method B - A 14 mL scintillation vial fitted with a Teflon stir bar was charged with $FeBr_2$ (30 mg, 0.14 mmol) and THF (4 mL). Mesityl magnesium bromide solution (0.43 mL, 1 M in THF, 0.43 mmol) was added dropwise. Benzoquinoline (75 mg, 0.43 mmol) was added. The solution was heated to 60 °C and stirred for 12 hours. The vial was removed from the glovebox and exposed to air. The vial was returned to the glovebox and allowed to stir for a further 2 hours at 60 °C. The precipitate was collected via suction filtration and washed with MeCN (**2-bzq**) or chilled MeOH (**2-ppy, 2-ptpy, 2-dfppy**). The solid was used without any further purification.

1.9.2.3 Fe(III) Complex Characterisation

2-bzq – **Method A:** Dark needle shaped crystals, 25% yield. **Method B:** Dark blue powder, 85% yield. **^{57}Fe Mössbauer parameters** (solid sample): δ = 0.04, |ΔE_Q| = 1.95 mm/s. **EPR parameters** (solid sample): $g_{||}$ = 2.47, $g_{\perp}$ = 1.84. **UV-Vis-NIR** (DCM): λ (ε) = 602 (4610), 549 (4060), 499 (3300), 431 (2650), 360 (10170), 347 (9930) nm (M^{-1} cm^{-1}).

2-ppy – **Method A:** dark violet crystals, 48% yield. **Method B:** dark violet powder, 28% yield. **^{57}Fe Mössbauer parameters** (solid sample): δ = -0.01, |ΔE_Q| = 1.82 mm/s. **EPR parameters** (solid sample): $g_{||}$ = 2.42, $g_{\perp}$ = 1.90. **UV-Vis-NIR** (DCM): λ (ε) = 578 (4660), 487 (3050), 370 (3880) nm (M^{-1} cm^{-1}); (MeCN): λ (ε) = 568 (4100), 476 (2700), 366 (3670) nm (M^{-1} cm^{-1}).

2-ptpy – **Method A:** dark violet crystals, < 10% yield. **Method B:** violet powder, 39% yield. Note: the use of $FeBr_2$ in method B resulted in the desired product **2-ppy** forming, however the resulting product could not be isolated. Instead, $Fe(acac)_3$ was used and the reaction mixture was cooled to -60 °C to precipitate the product. **^{57}Fe Mössbauer parameters** (solid sample): δ = 0.01, |ΔE_Q| = 1.82 mm/s. **EPR parameters** (solid sample): $g_{||}$ = 2.46, $g_{\perp}$ = 1.87. **UV-Vis-NIR** (MeCN): λ (ε) = 566 (4450), 502 (3580) nm (M^{-1} cm^{-1}).

2-dfppy – **Method A:** orange crystals, < 10% yield. **Method B:** orange powder, 32% yield. **^{57}Fe Mössbauer parameters** (solid sample): δ = -0.01, |ΔE_Q| = 1.83 mm/s. **EPR parameters** (solid sample): $g_{||}$ = 2.46, $g_{\perp}$ = 2.00. **UV-Vis-NIR** (MeCN): λ (ε) = 515 (3600), 476 (4350) nm (M^{-1} cm^{-1}).

1.9.3 Preparation of Tris-cyclometalated Fe(II) complexes

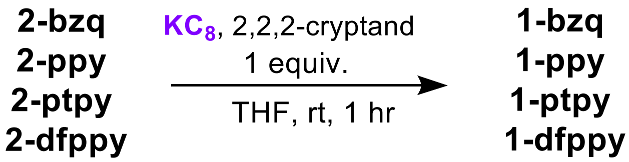

Scheme 2. General method for the preparation of tris-cyclometalated Fe(II) complexes.

1.9.3.1 General Method - To a 14 mL scintillation vial fitted with a teflon stir bar was added **2-ppy**, **2-ptpy** or **2-dfppy** (0.052 mmol), potassium graphite (7.1 mg, 0.052 mmol), 4,7,13,16,21,24-Hexaoxa-1,10-diazabicyclo[8.8.8]hexacosane (2.2.2 cryptand, 19.6 mg, 0.052 mmol) and 4 mL of THF (**2-ppy**, **2-ptpy**) or 4 mL of DME (**2-dfppy**). After stirring mixture at RT for 1 h, solution was filtrated through Celite and 4 mL of Et_2O were added prior storing solution at -30 °C. Crystals suitable for SC-XRD were observed after 1 day. Crystals were collected via suction filtration and washed with Et_2O (3 x 1 mL).

1.9.3.2 Synthesis of 1-bzq– A 14 mL scintillation vial was charged with a teflon stir bar, $Fe(bzq)_3$ (15 mg, 0.025 mmol), potassium graphite (3.4 mg, 0.025 mmol), 4,7,13,16,21,24-Hexaoxa-1,10-diazabicyclo[8.8.8]hexacosane (2.2.2-cryptand, 9.4 mg, 0.025 mmol) and MeCN (4 mL). After stirring mixture at RT for 1 h, solution was filtered through Celite and 8 mL of Et_2O was added. The precipitate was collected via suction filtration and washed with Et_2O (3 x 2 mL) (54 % yield). To grow crystals suitable for SC-XRD, the reaction mixture post filtration (0.5 mL) was set up for vapor diffusion with Et_2O (1 mL). After two days stored at room temperature in the glovebox, crystals suitable for SC-XRD were formed.

1.9.3.3 Fe(II) Complex Characterisation

1-bzq – Dark green crystals, 54% yield. **^{57}Fe Mössbauer parameters** (solid sample): δ = 0.06, |ΔE_Q| = 0.65 mm/s. **UV-Vis-NIR** (**MeCN**): λ (ε) = 918 (12180), 696 (10230), 628 (9000), 535 (5440), 476 (5780), 436 (5870), 356 (26310) nm (M^{-1} cm^{-1}). **^{1}H NMR (400 MHz, MeCN-*d*$_3$):** δ=7.60-7.65 (m, 9H, *j = 7 Hz,* CH), 7.49 (d, 3H, *j = 8 Hz,* CH), 6.93 (d, 3H, *j = 8 Hz,* CH), 6.80-6.86 (m, 6H, *j = 8 Hz,* CH), 6.66 (d, 3H, *j = 8 Hz,* CH), 3.58 (s, 12H, CH_2), 3.52 (t, 12H, *j = 6 Hz,* CH_2), 2.53 (t, 12H, *j = 4 Hz,* CH_2).

1-ppy – Dark crystal, 30 % yield suitable for SC-XRD were formed.**^{57}Fe Mössbauer parameters** (solid sample): δ = 0.04, |ΔE_Q| = 0.83 mm/s. **UV-Vis-NIR** (**MeCN**): λ (ε) = 900 (8100), 748 (14930), 535 (10340), 418 (10340) nm (M^{-1} cm^{-1}). **^{1}H NMR (400 MHz, MeCN-*d*$_3$):** δ = 7.56 (s, 8H, CH), 7.34 (s, 4H, CH), 6.39 (s, 4H, CH), 6.23 (s, 4H, CH), 5.86 (s, 4H, CH), 3.56-3.51 (d, 24H, *j =20 Hz*, CH_2), 2.52 (s, 12 H, CH_2).

1-ptpy - **^{57}Fe Mössbauer parameters** (solid sample): δ = 0.04, |ΔE_Q| = 0.81 mm/s. **UV-Vis-NIR** (MeCN): λ (ε) = 900 (4460), 748 (9410), 542 (7570), 425 (7150) nm (M^{-1} cm^{-1}). **^{1}H NMR (400 MHz, MeCN-*d*$_3$):** δ = 7.47-7.55 (m, 6H, *J= 8 Hz*, CH), 7.17-7.21 (t, 3H, *J=8 Hz*, CH), 6.40 (s, 3H, CH), 6.22-6.24 (d, 3H, *J= 8Hz,* CH), 6.13 (s, 6H, CH), 3.50-3.56 (m, 24H, CH_2), 2.51-2.52 (m, 12H, CH_2).

1-dfppy – **^{57}Fe Mössbauer parameters** (solid sample): δ = 0.03, |ΔE_Q| = 0.65 mm/s. **UV-Vis-NIR** (MeCN): λ (ε) = 716 (8980), 685 (9290), 569 (6510), 503 (4950), 415 (8270) nm (M^{-1} cm^{-1}). **^{1}H NMR (400 MHz, MeCN-*d*$_3$):** δ = 8.05 (s, 3H, CH), 7.36-7.26 (d, 6H, *j= 40* Hz, CH), 6.66 (s, 3H, CH), 6.02 (s, 6H, CH), 3.54 (s, 24H, CH_2), 2.51 (s, 12 H, CH_2). **^{19}F NMR (400 MHz, MeCN-*d*$_3$):** δ = -113.72, -118.39.

1.9.4 Preparation of tris-cyclometalated Fe(IV) complex

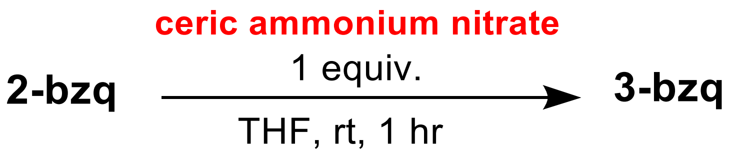

Scheme 3. General method for the preparation of tris-cyclometalated Fe(Iv) complexes

3-bzq – To a 20 mL scintillation vial fitted with a teflon stir bar was added $Fe(bzq)_3$ (0.025 mmol), ceric ammonium nitrate (13.7 mg, 0.025 mmol) and 4 mL of MeCN. After stirring the mixture at RT for 1 h, the solution was filtrated through Celite and 2 mL of Et_2O was added prior to storing the solution at -30 °C. After a day, dark green crystals (5.2 mg, 10% yield) suitable for SC-XRD were formed. **^{57}Fe Mössbauer parameters** (solid sample): δ = -0.05, |ΔE_Q| = 1.30 mm/s. **UV-Vis-NIR** (MeCN): λ (ε) = 702 (6160), 515 (2340), 403 (9100), 352 (21740) nm (M^{-1} cm^{-1}).

2. Mössbauer spectra

2.1 Solution Mössbauer Spectra

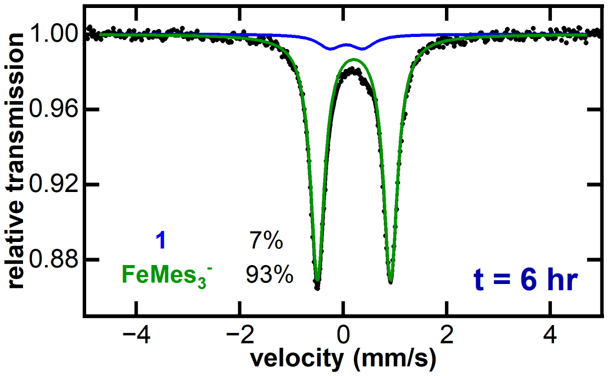

Figure S1. Zero-field, 80 K ^{57}Fe Mössbauer Spectrum of **FeMes$_3^-$** reacted with 3.1 equiv. of Hbzq at RT for 6 hours. Black dotted trace: raw data. Black solid trace: best fit. Coloured traces: simulation. **FeMes$_3^-$:** δ = 0.21, |ΔE_Q| = 1.43 mm/s. **1:** δ = 0.06, |ΔE_Q| = 0.71 mm/s.

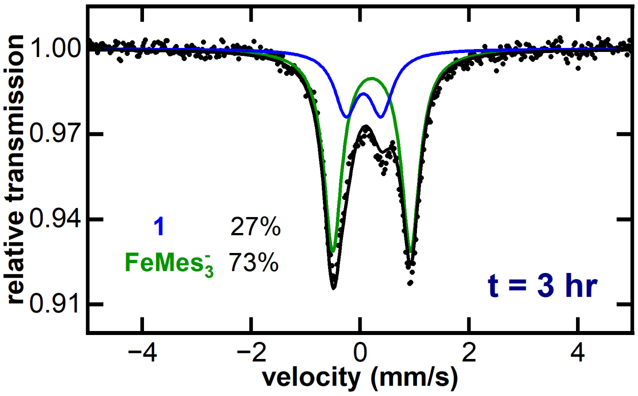

Figure S2. Zero-field, 80 K ^{57}Fe Mössbauer Spectrum of **FeMes$_3^-$** reacted with 1 equiv. of Hbzq at 60 °C for 3 hours. Black dotted trace: raw data. Black solid trace: best fit. Coloured traces: simulation. **FeMes$_3^-$**, green trace: δ = 0.21, |ΔEQ| = 1.43 mm/s. **1,** blue trace: δ = 0.06, |ΔEQ| = 0.71 mm/s.

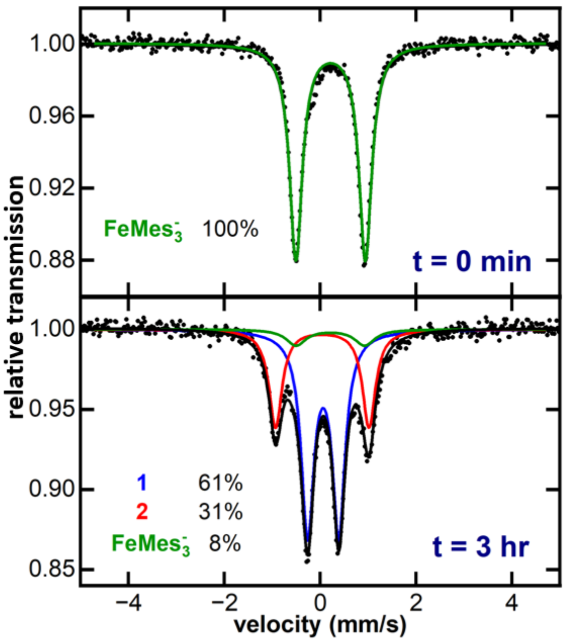

Figure S3. Zero-field, 80 K ^{57}Fe Mössbauer spectra of in situ generated **$FeMes_3^-$** reacted with 3.1 equiv. of Hbzq at 60 °C for 3 hours. Black dotted trace: raw data. Black solid trace: best fit. Coloured traces: simulation. **$FeMes_3^-$**, green trace: δ = 0.21, $|\Delta E_Q|$ = 1.43 mm/s. **1**, blue trace: δ = 0.06, $|\Delta E_Q|$ = 0.71 mm/s. **2**, red trace: δ = 0.04, $|\Delta E_Q|$ = 1.95 mm/s.

2.2 Solid Mössbauer Spectra

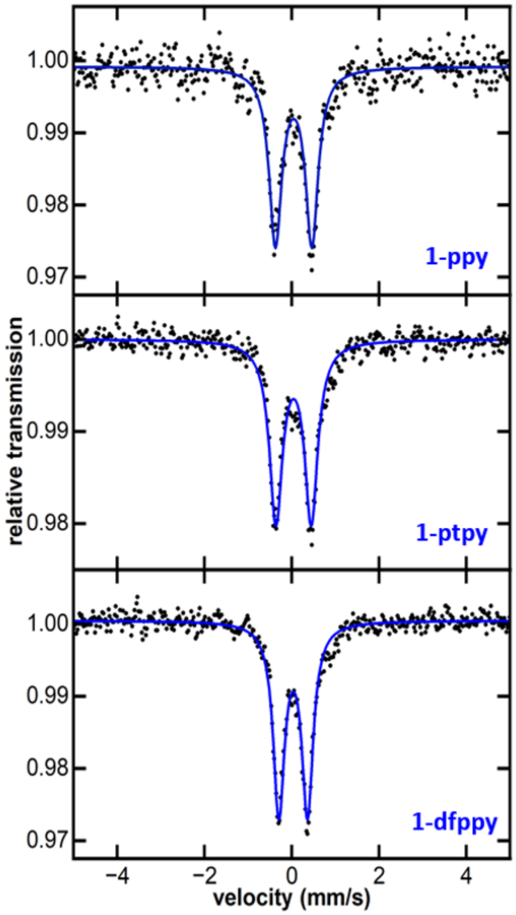

Figure S4. Zero-field, 80 K ^{57}Fe Mössbauer spectra of solid **1-ppy, 1-ptpy** and **1-dfppy**. Black dotted trace: raw data. Blue trace: simulation. **1-ppy:** $\delta = 0.04$, $|\Delta E_Q| = 0.83$ mm/s. **1-ptpy:** $\delta = 0.04$, $|\Delta E_Q| = 0.81$ mm/s **1-dfppy** $\delta = 0.03$, $|\Delta E_Q| = 0.65$ mm/s.

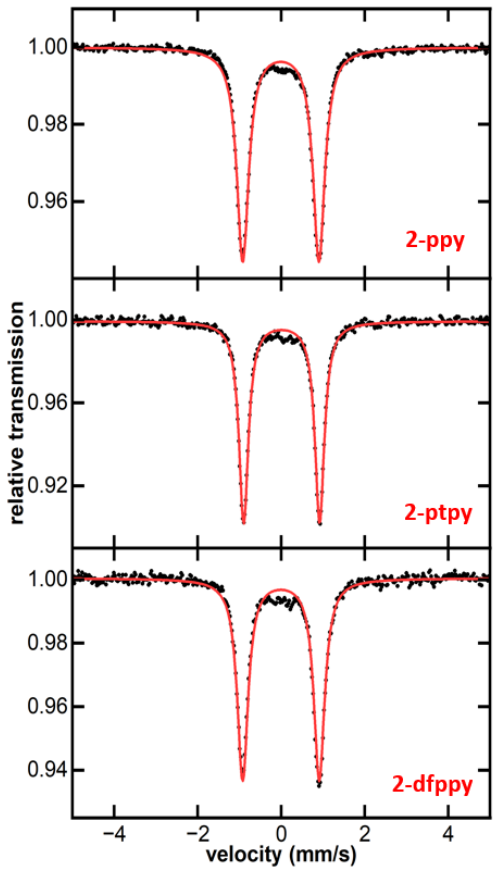

Figure S5. Zero-field, 80 K ^{57}Fe Mössbauer spectra of solid samples **2-ppy**, **2-ptpy** and **2-dfppy**. Black dotted trace: raw data. Red trace: simulation. **2-ppy:** δ = -0.01, $|\Delta E_Q|$ = 1.82 mm/s. **2-ptpy:** δ = 0.01, $|\Delta E_Q|$ = 1.82 mm/s. **2-dfppy:** δ = -0.01, $|\Delta E_Q|$ = 1.83 mm/s.

3. EPR spectra

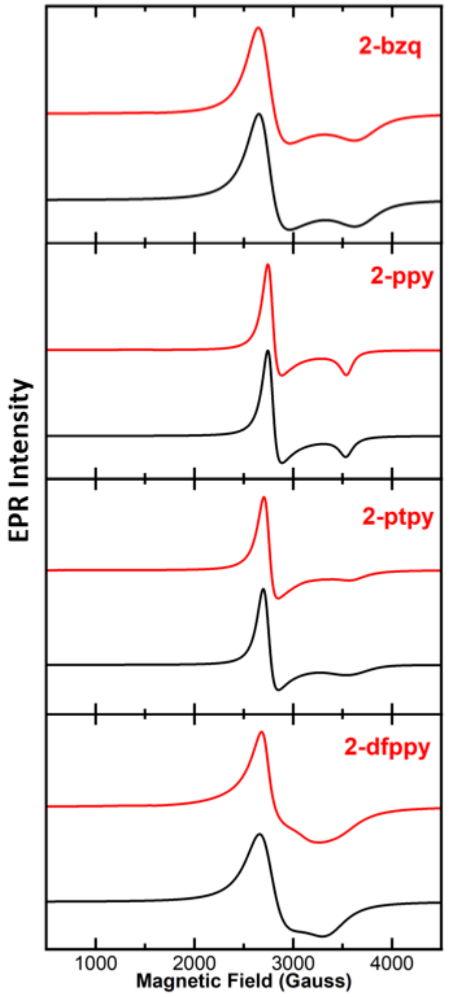

Figure S6. 10 K EPR spectra of solid **2-bzq**, **2-ppy**, **2-ptpy** and **2-dfppy**. Black trace: experimental data. Red trace: corresponding simulations.

4. Cyclic Voltammograms

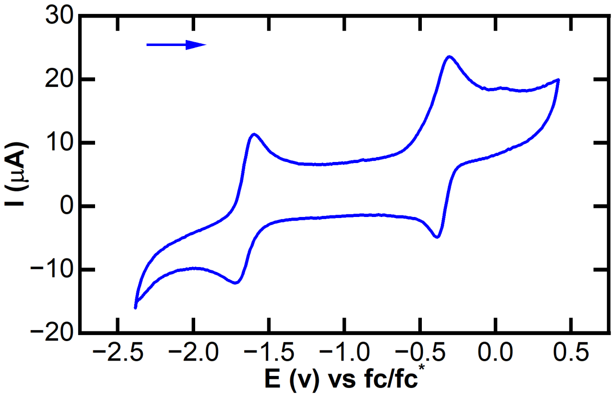

Figure S7. Cyclic voltammogram of **1-bzq** in 0.3 M $[NBu_4][PF_6]$ in MeCN at 298 K, scan rate = 100 mV s^{-1}.

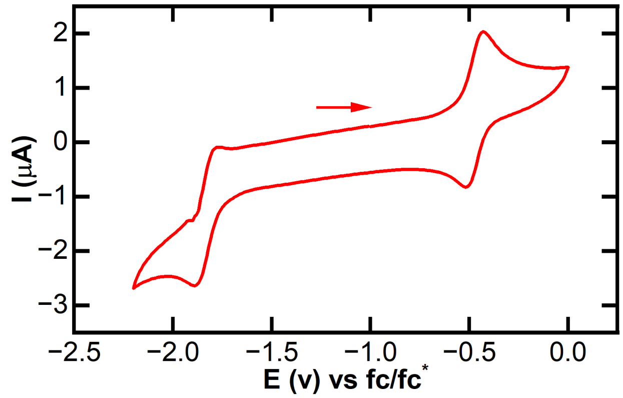

Figure S8. Cyclic voltammogram of **2-bzq** in 0.1 M $[NBu_4][PF_6]$ in DCM at 298 K, scan rate = 25 mV s^{-1}.

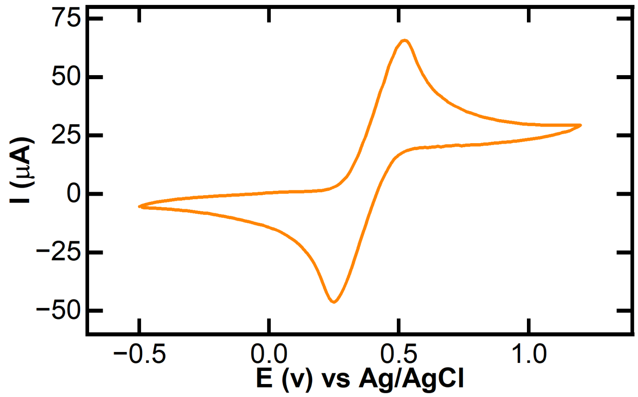

Figure S9. Cyclic voltammogram of ferrocene in 0.3 M $[NBu_4][PF_6]$ in MeCN at 298 K, scan rate = 100 mV s^{-1}.

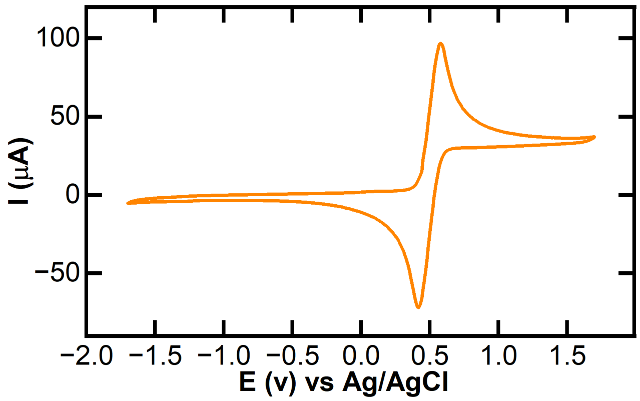

Figure S10. Cyclic voltammogram of ferrocene in 0.3 M $[NBu_4][PF_6]$ in DCM at 298 K, scan rate = 100 mV s^{-1}.

5. UV-VIS Spectra

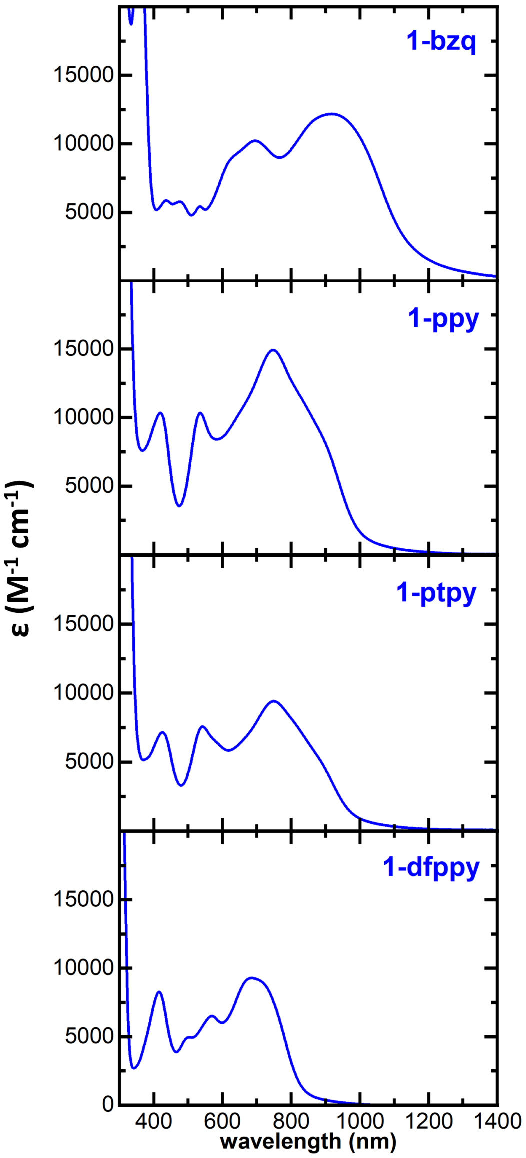

Figure S11. Electronic absorption spectra of complexes **1-bzq, 1-ppy**, **1-ptpy** and **1-dfppy** in MeCN.

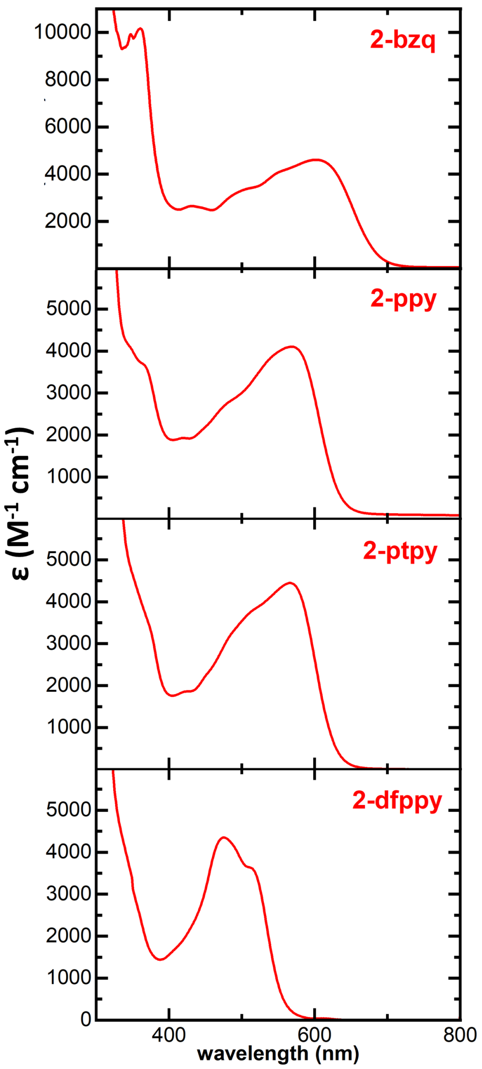

Figure S12. Electronic absorption spectra of complexes **2-bzq** in DCM and **2-ppy**, **2-ptpy** and **2-dfppy** in MeCN.

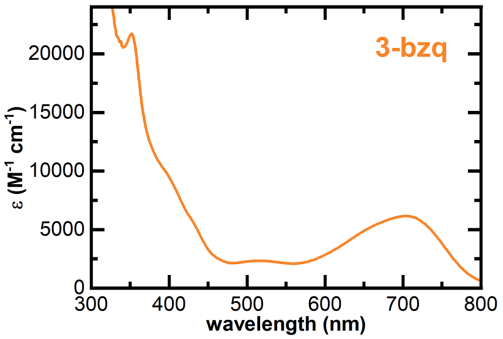

Figure S13. Electronic absorption spectra of complex **3-bzq** in MeCN.

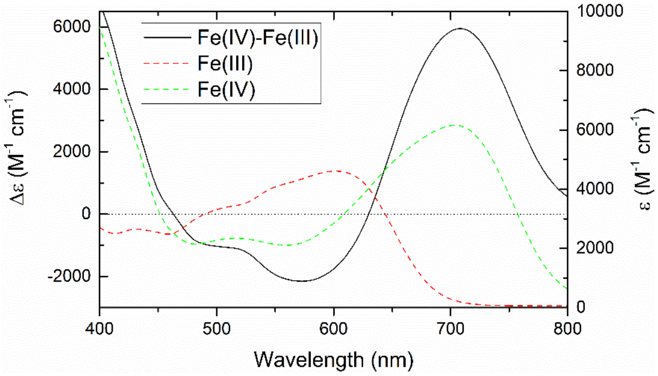

Figure S14. Electronic absorption difference spectrum of Fe(IV) and Fe(III) complexes with bzq ligand.

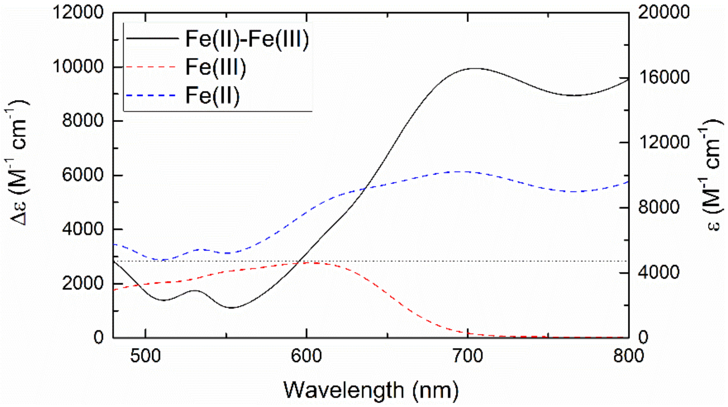

Figure S15. Electronic absorption difference spectrum of Fe(II) and Fe(III) complexes with bzq ligand.

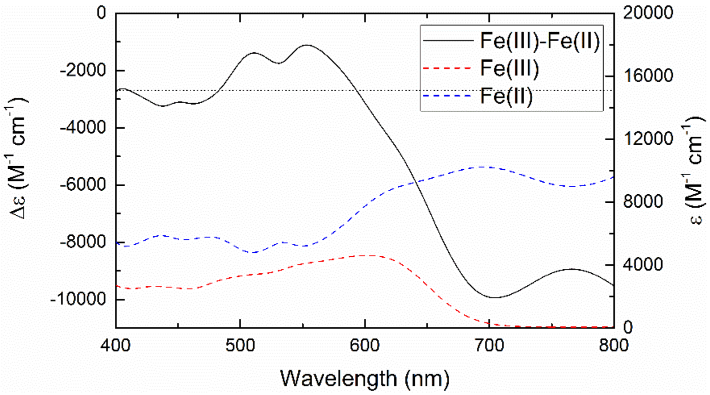

Figure S16. Electronic absorption difference spectrum of Fe(III) and Fe(II) complexes with bzq ligand.

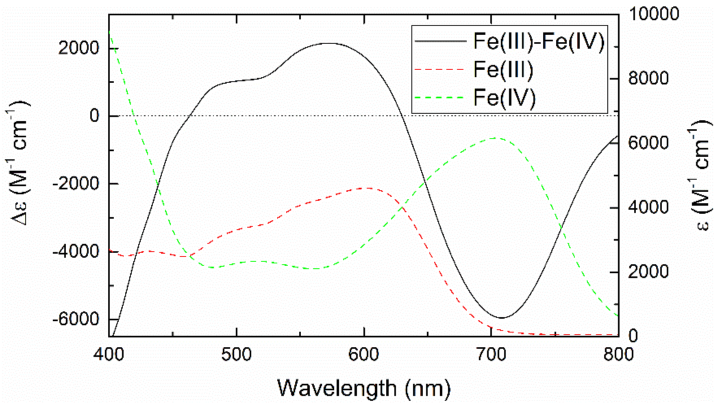

Figure S17. Electronic absorption difference spectrum of Fe(III) and Fe(IV) complexes with bzq ligand.

6. NMR data

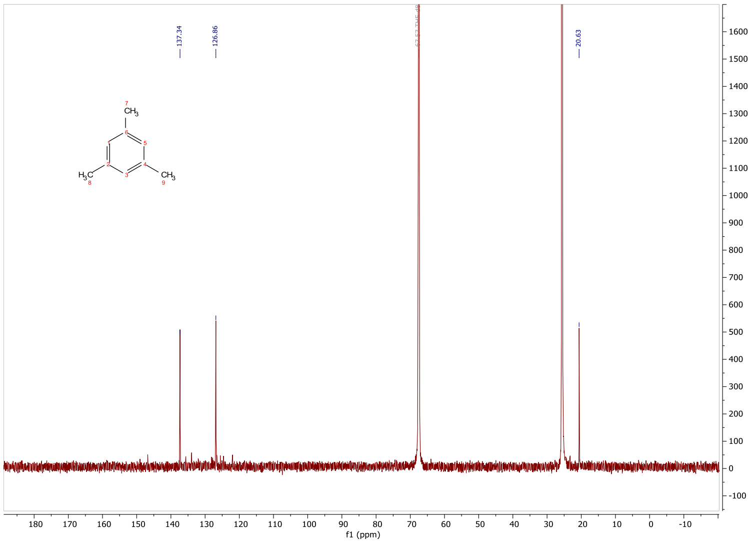

Figure S18. $^{13}C\{^1H\}$ NMR spectrum of crude reaction mixture in THF. $FeBr_2$ (30 mg, 0.14 mmol) was suspended in THF (4 mL). Mesityl Magnesium Bromide solution (0.43 mL, 1 M in THF, 0.43 mmol) was added dropwise. Hbzq (75 mg, 0.43 mmol) was added. The solution was heated to 60 °C for 6 hours, at which point the iron precipitated. The solution was filtered over celite and NMR was run on the crude reaction.

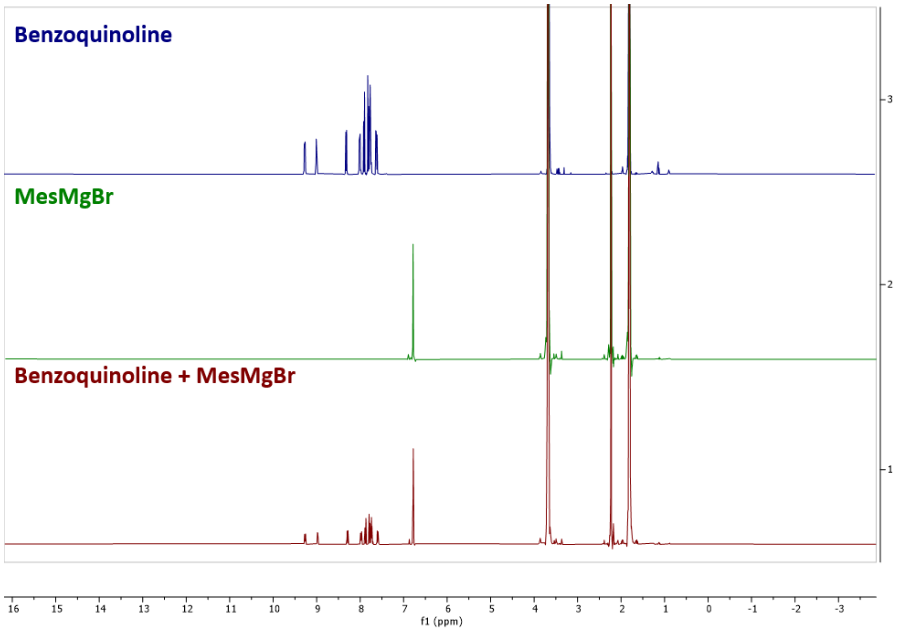

Figure S19. ^1H NMR of the reaction of benzoquinoline ligand (blue spectrum) with mesityl magnesium bromide (green spectrum) in MeCN-d_3 resulting in no discernable changes (red spectrum).

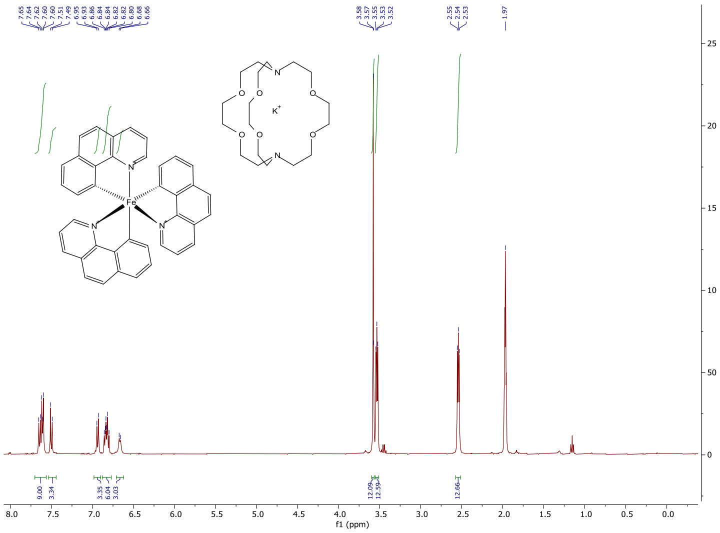

Figure S20. ^{1}H NMR spectrum of complex **1-bzq** in MeCN-d_3.

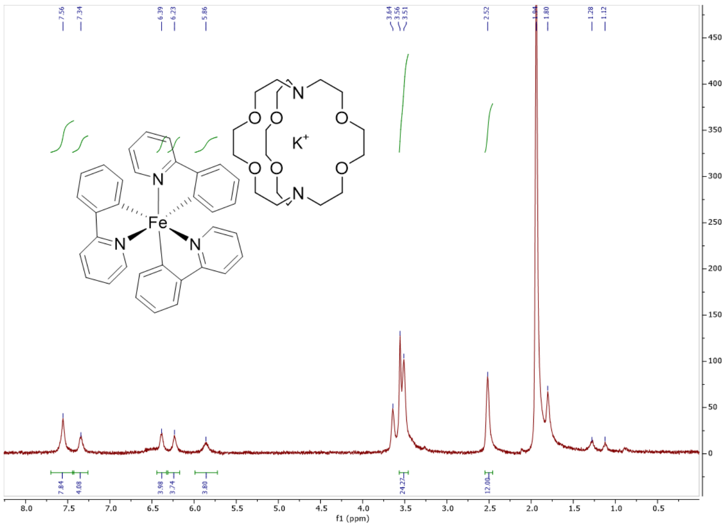

Figure S21. ^{1}H NMR spectrum of complex **1-ppy** in MeCN-d_3. Slight broadening of the signals is attributed to trace amounts of the paramagnetic Fe(III) complex **2-ppy** in solution.

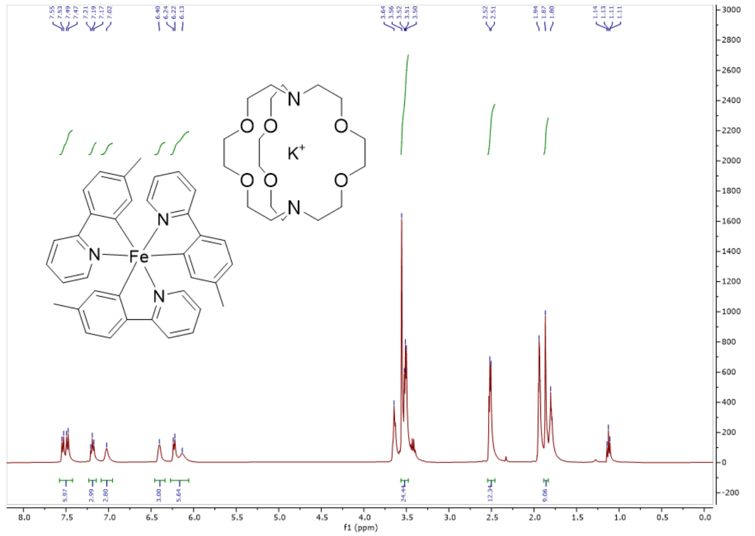

Figure S22. ^{1}H NMR spectrum of complex **1-ptpy** in MeCN-d_3.

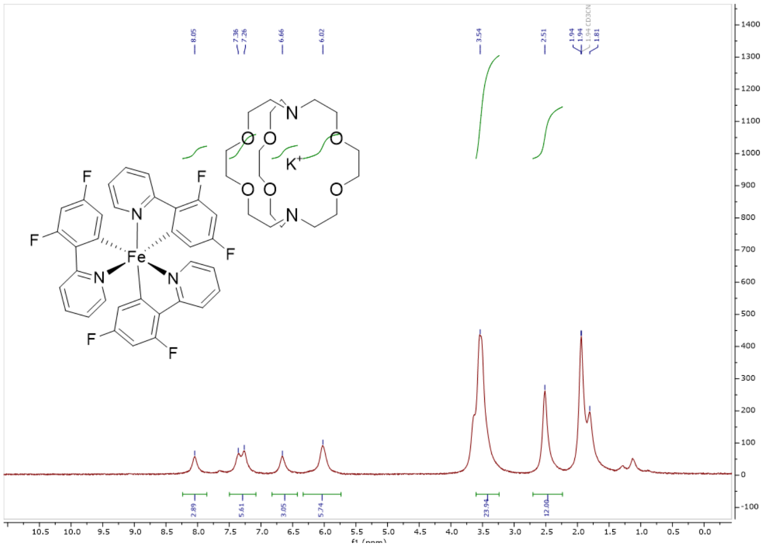

Figure S23. ^{1}H NMR spectrum of complex **1-dfppy** in MeCN-d_3. Slight broadening of the signals is attributed to trace amounts of the paramagnetic Fe(III) complex **2-dfppy** in solution.

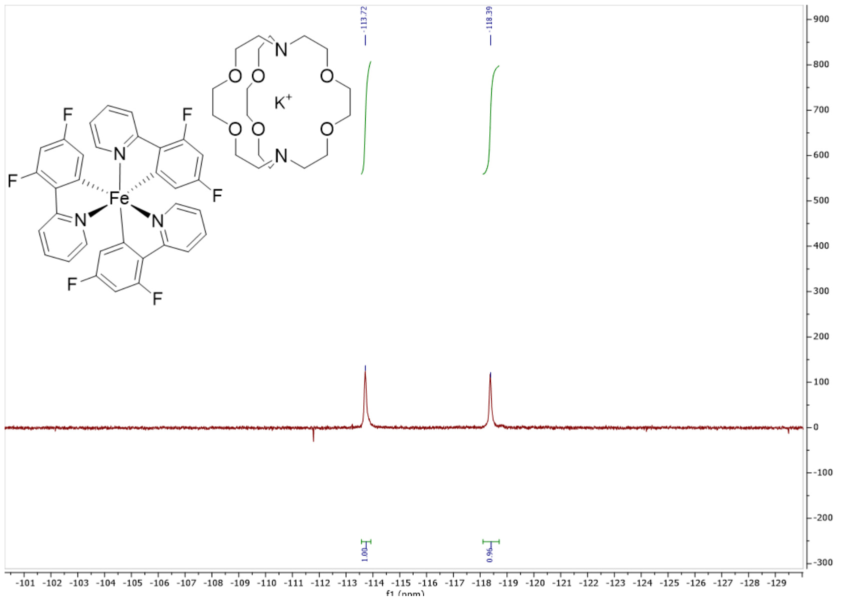

Figure S24. ^{19}F NMR spectrum of complex **1-dfppy** in MeCN-d_3.

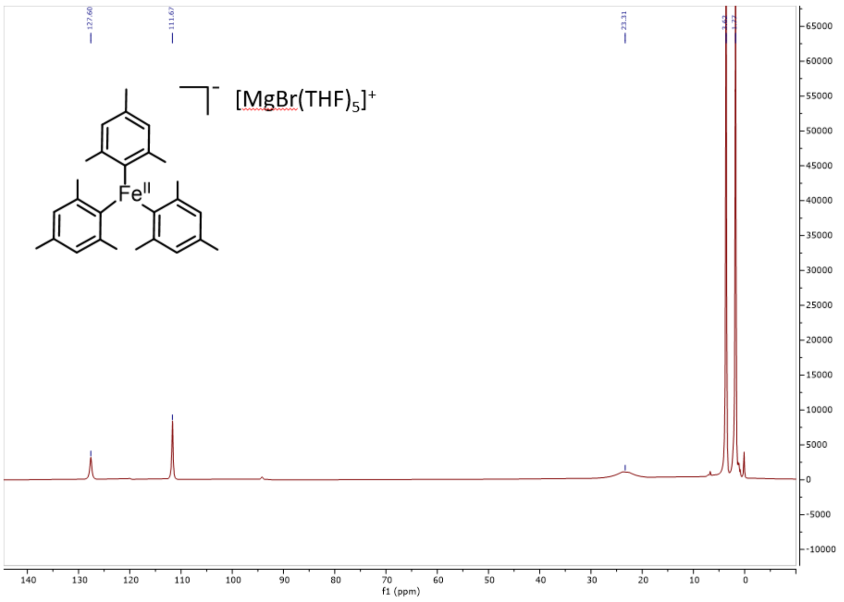

Figure S25. ^{1}H NMR spectrum of **FeMes$_3$**$^-$ ([Fe(Mes)$_3$][MgBr(THF)$_5$]) in THF-d_8.

7. Transient Absorption Spectra and Kinetics

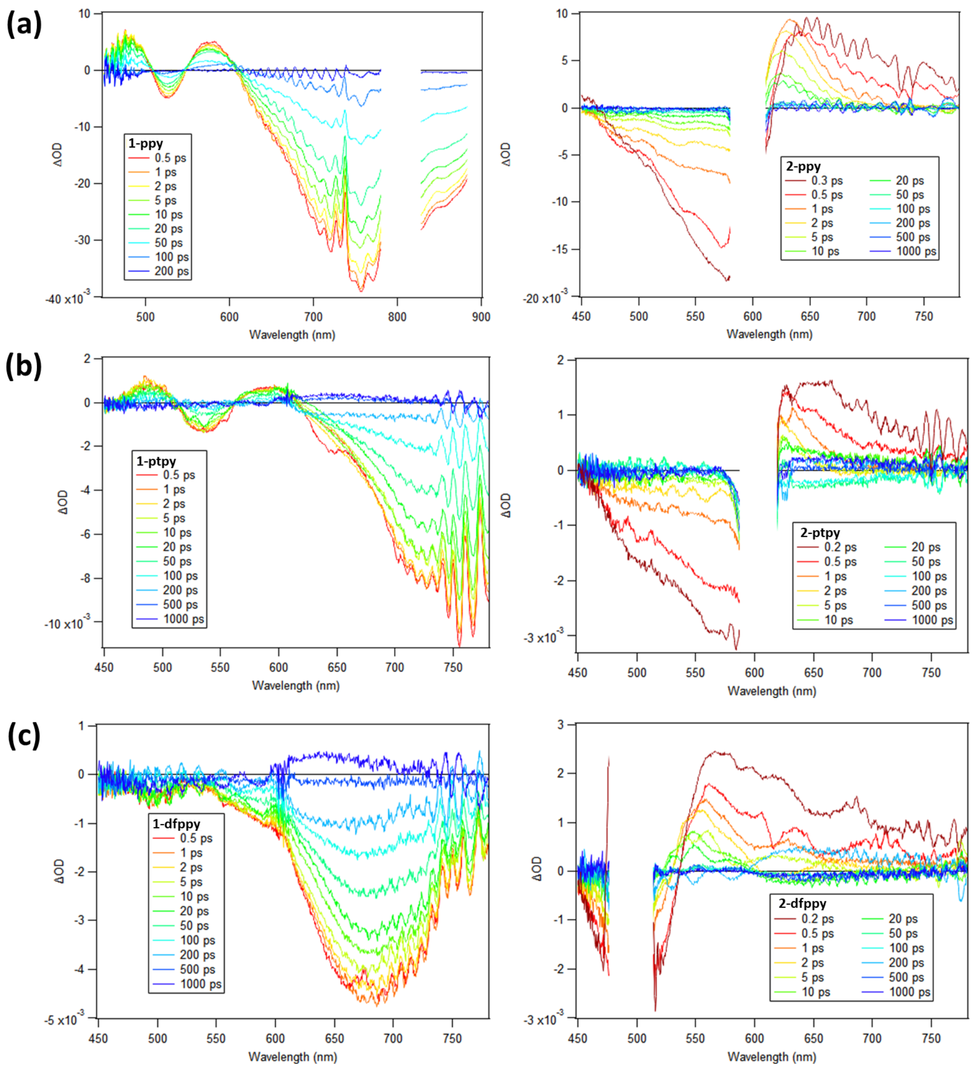

Figure S26. Transient absorption spectra of (a) **1-ppy** and **2-ppy** (b) **1-ptpy** and **2-ptpy**, and (c) **1-dfppy** and **2-dfppy.**

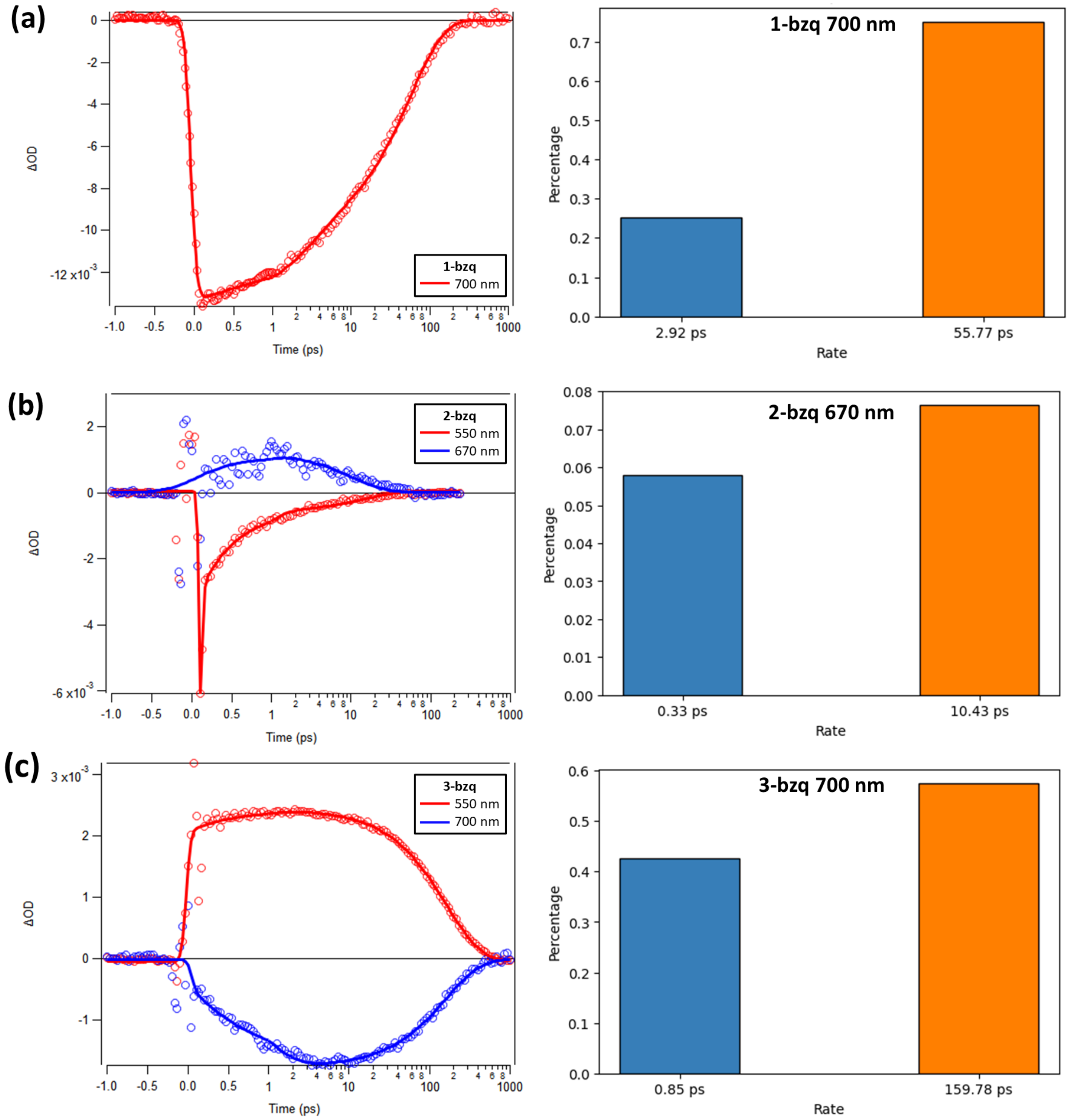

Figure S27. (left) Transient absorption kinetic traces (unfilled circles) alongside their fitted curves (solid lines), and (right) the percentages of decay versus time constants for the selected kinetic traces of **(a) 1-bzq**, **(b) 2-bzq**, and **(c) 3-bzq**. **1-bzq** exhibits a 56 ps lifetime. **2-bzq** exhibits an ultrafast relaxation to the ground state in 0.33 ps, followed by vibrational cooling in 10 ps. **3-bzq** exhibits a 160 ps excited-state lifetime.

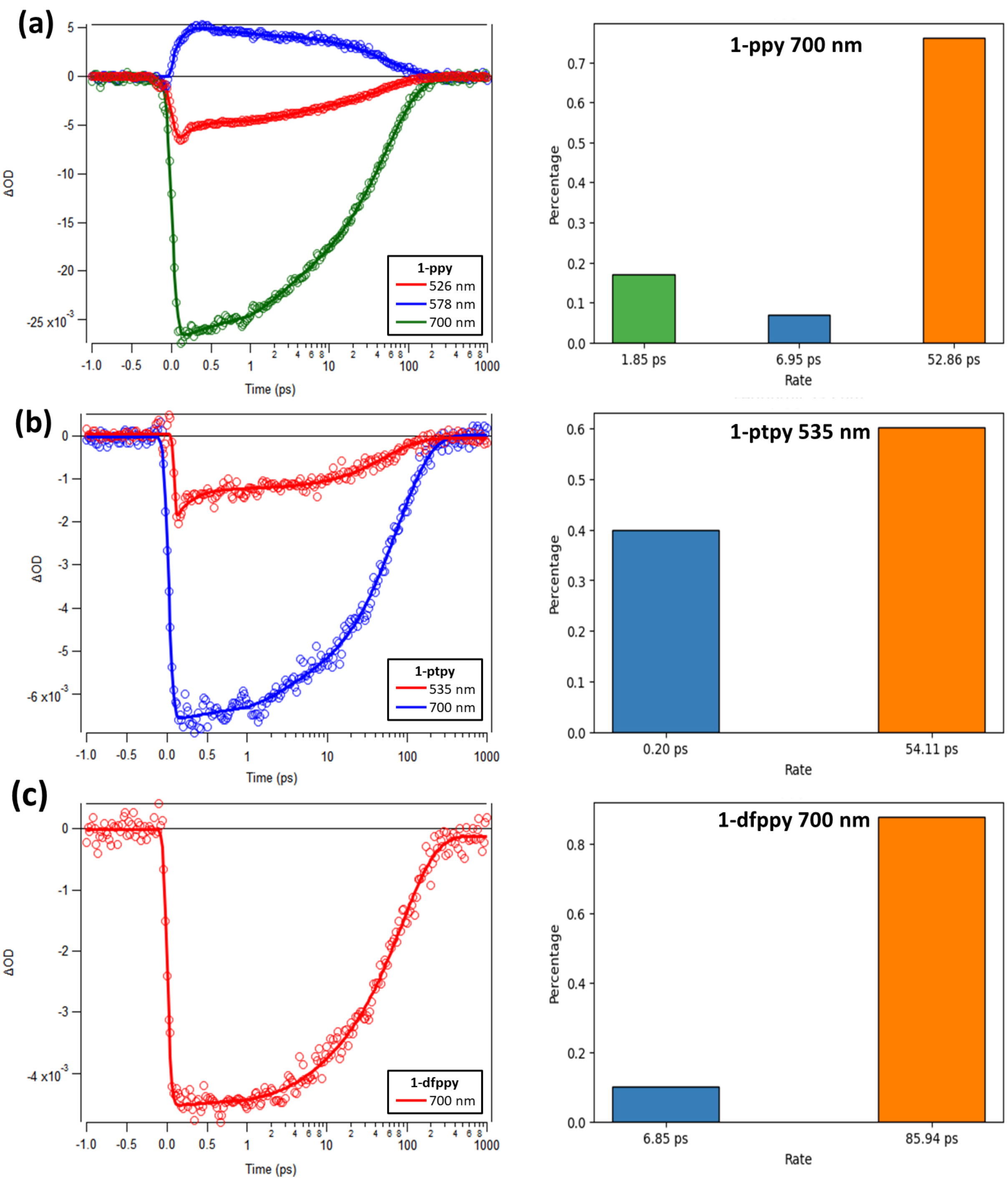

Figure S28. (left) Transient absorption kinetic traces (unfilled circles) alongside their fitted curves (solid lines), and (right) the percentages of decay versus time constants for the selected kinetic traces of (a) **1-ppy**, (b) **1-ptpy** and (c) **1-dfppy** and **1-ppy** exhibits a 47-53 ps excited-state lifetime. We interpret the kinetics of **1-ptpy** to imply a 54 ps excited state lifetime. **1-dfppy** exhibits a clear 86 ps lifetime.

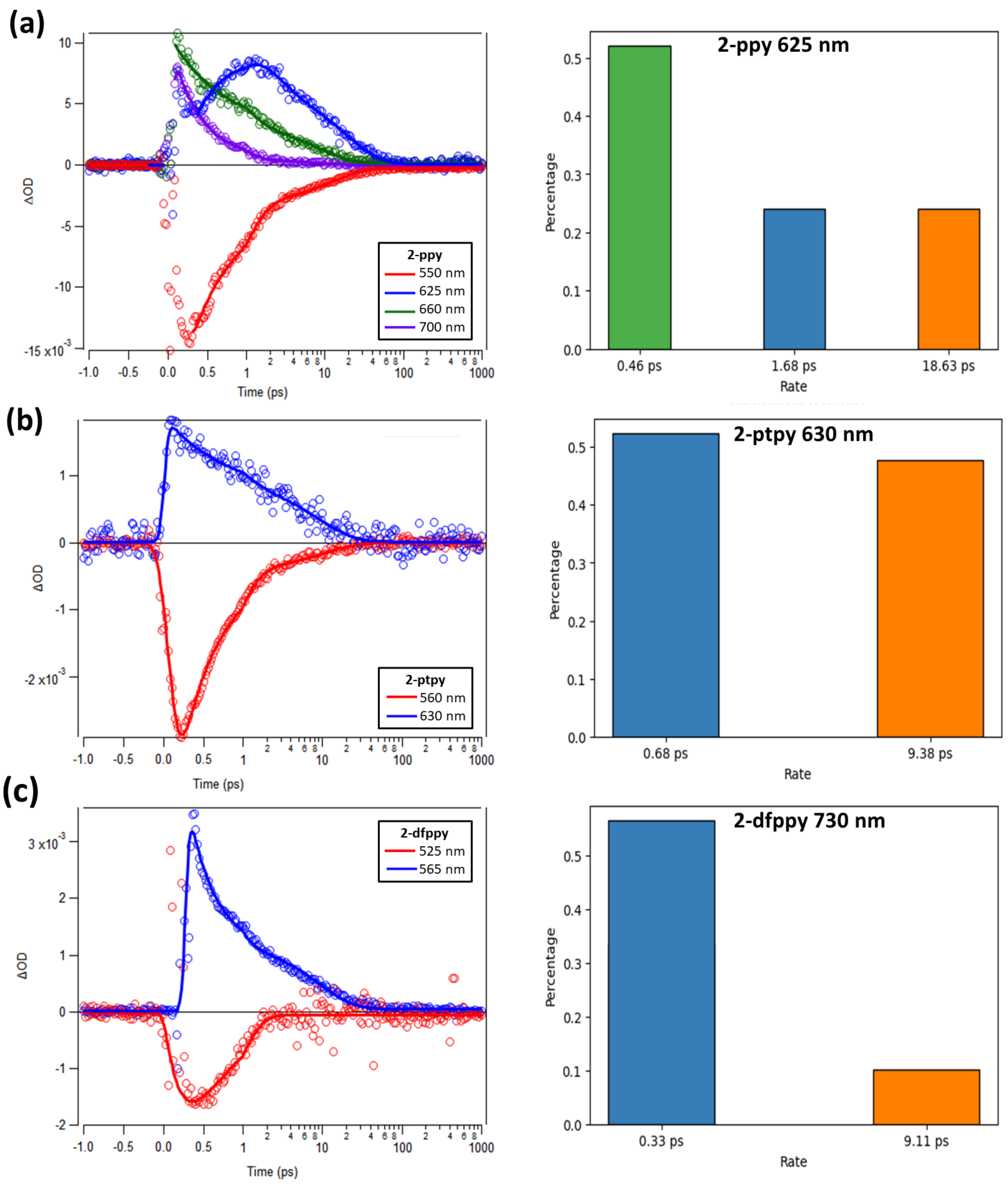

Figure S29. (left) Transient absorption kinetic traces (unfilled circles) alongside their fitted curves (solid lines), and (right) the percentages of decay versus time constants for the selected kinetic traces of (a) **2-ppy**, (b) **2-ptpy** and (c) **2-dfppy**. **2-ppy** exhibits ultrafast relaxation to the ground state in 0.5 ps, followed by vibrational cooling in 2-19 ps. **2-ptpy** exhibits a <1 ps excited state lifetime followed by vibrational cooling in 9 ps. **1-dfppy** shows complicated rapid relaxation that we interpret as 0.3 ps relaxation to the ground state followed by vibrational cooling in 9 ps.

Table S1. Multiexponential regression results for all kinetic traces.

Sample Wavelength (nm)	*t1 (ps)*	*Ampl.%*	*t2 (ps)*	*Ampl.%*	*t3 (ps)*	*Ampl.%*	*Long-term ampl.%*
1-bzq							
700	55.76	-74.9%	2.92	-25.1%	--	--	--
2-bzq							
550	0.012	-86.6%	0.45	-10.7%	14.87	-2.7%	--
670	0.016	86.6%	0.33	-5.8%	10.43	7.6%	--
3-bzq							
550	163.60	86.5%	0.546	-13.5%	--	--	--
700	159.78	-57.4%	0.847	42.6%	--	--	--
1-ppy							
526	0.13	43.4%	3.14	15.3%	47.46	41.4%	--
578	0.15	-45.0%	0.37	23.0%	55.53	32.0%	--
700	1.85	16.7%	6.95	7.3%	52.86	76.1%	--
2-ppy							
550	0.60	79.2%	12.13	19.3%	--	--	1.5%
625	0.46	-51.9%	1.68	23.5%	18.63	24.4%	0.2%
660	0.64	70.5%	8.40	28.5%	--	--	1.0%
700	0.37	94.1%	5.66	5.8%	--	--	0.1%
1-ptpy							
535	54.11	-60.2%	0.20	-39.8%	--	--	--
700	75.69	-88.6%	2.51	-11.4%	--	--	--
2-ptpy							
560	0.49	-89.6%	10.20	-10.4%	--	--	--
630	0.68	52.3%	9.38	47.7%	--	--	--
1-dfppy							
700	85.94	-87.5%	6.85	-10.0%	--	--	-2.4%
2-dfppy							
525	0.30	50.2%	0.43	-49.8%	--	--	--
565	0.33	-33.4%	0.33	56.5%	9.11	10.1%	--

8. Single Crystal X-Ray Diffraction

Table S2. Selected structural parameters of Fe(II) complexes.

Complex	$Fe\text{-}N_{avg}$ (Å)	$Fe\text{-}C_{avg}$ (Å)	$N\text{-}Fe\text{-}N_{avg}$ (°)	$C\text{-}Fe\text{-}C_{avg}$ (°)	trans $C\text{-}Fe\text{-}N_{avg}$ (°)	$C\text{-}Fe\text{-}N_{avg}$ (°)
**1-bzq**	1.9983	1.9515	91.90	93.05	173.25	82.88
**1-ppy**	1.9930	1.9540	93.08	92.56	171.99	81.68
**1-ptpy**	1.9869	1.9420	93.60	94.45	174.30	82.37
**1-dfppy**	1.9880	1.9320	93.27	94.36	174.35	82.22

Table S3. Selected structural parameters of Fe(III) complexes.

Complex	$Fe\text{-}N_{avg}$ (Å)	$Fe\text{-}C_{avg}$ (Å)	$N\text{-}Fe\text{-}N_{avg}$ (°)	$C\text{-}Fe\text{-}C_{avg}$ (°)	trans $C\text{-}Fe\text{-}N_{avg}$ (°)	$C\text{-}Fe\text{-}N_{avg}$ (°)
**2-bzq**	2.0511	1.9545	90.88	94.98	173.02	82.62
**2-ppy**	2.0090	1.9780	95.62	93.76	174.76	81.68
**2-ptpy**	2.0357	1.9529	93.80	93.55	173.98	82.15
**2-dfppy**	2.0252	1.9500	94.38	93.88	174.93	82.04
**2-mppy**	2.0360	1.9436	94.18	94.84	173.44	80.85
**2-dfpfpy**	2.0223	1.9490	94.48	93.74	174.49	82.03

Table S4. Selected structural parameters of Fe(IV) complex.

Complex	$Fe\text{-}N_{avg}$ (Å)	$Fe\text{-}C_{avg}$ (Å)	$N\text{-}Fe\text{-}N_{avg}$ (°)	$C\text{-}Fe\text{-}C_{avg}$ (°)	trans $C\text{-}Fe\text{-}N_{avg}$ (°)	$C\text{-}Fe\text{-}N_{avg}$ (°)
**3-bzq**	2.051	1.972	92.30	90.44	173.56	84.30

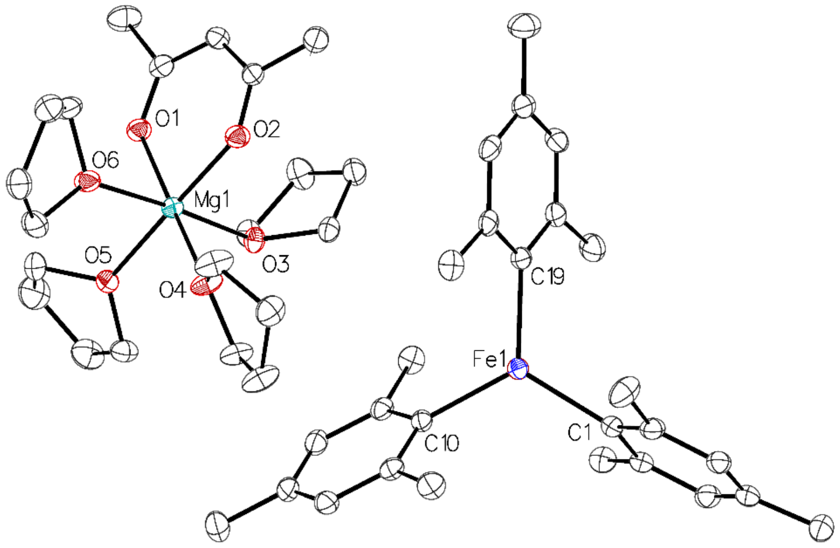

Figure S30. Crystal structure of **[Fe(Mes)$_3$][Mg(acac)(THF)$_4$]**. Hydrogen atoms are omitted for clarity and the thermal ellipsoids are shown at 50% probability. Crystal was isolated from a reaction mixture of **2-bzq** method A.

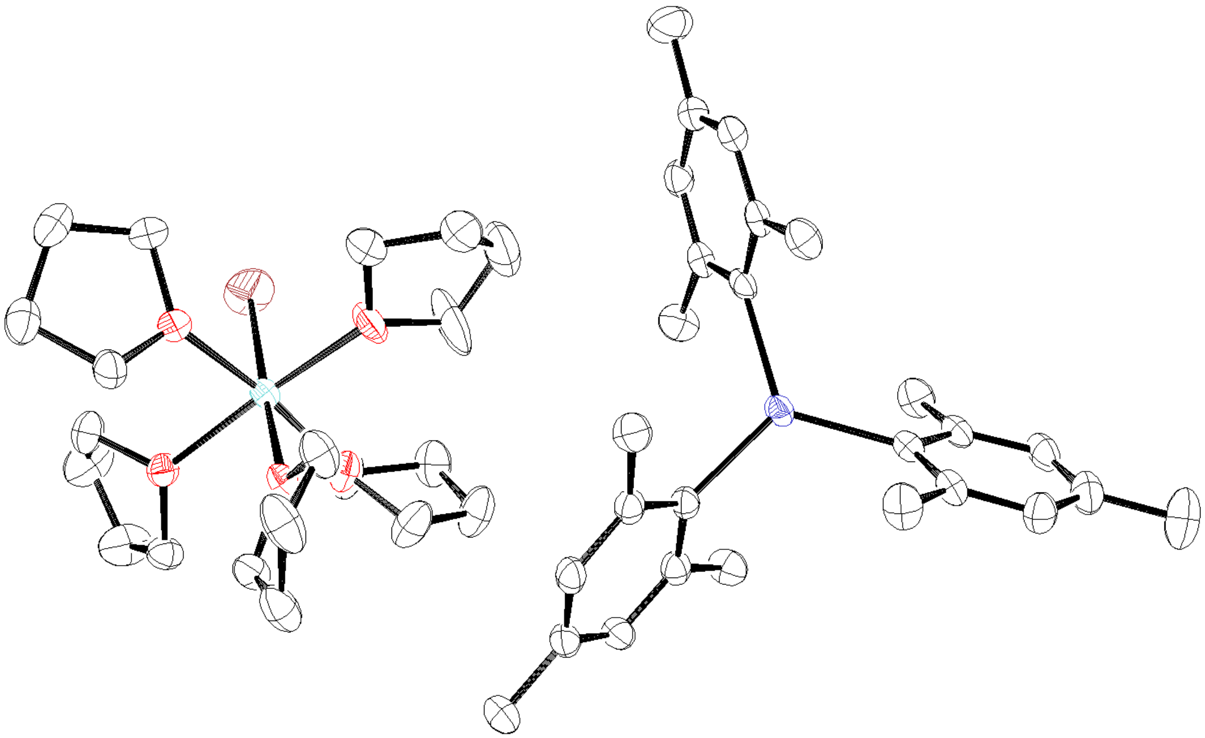

Figure S31. Crystal structure of **[Fe(Mes)$_3$][MgBr(THF)$_5$]**. Hydrogen atoms are omitted for clarity and the thermal ellipsoids are shown at 50% probability.

Table S5. Crystal data and structure refinement of **[Fe(Mes)$_3$][Mg(acac)(THF)$_4$]** and **[Fe(Mes)$_3$][MgBr(THF)$_5$]**.

	**[Fe(Mes)$_3$][Mg(acac)(THF)$_4$]**	**[Fe(Mes)$_3$][MgBr(THF)$_5$]**.
Empirical formula	C48 H72 Fe Mg O6	C47 H73 Br Fe Mg O5
Formula weight	825.21 g mol^{-1}	878.13 g mol^{-1}
Temperature	100.00(10) K	150 K
Wavelength	1.54184 Å	1.54184 Å
Crystal system	orthorhombic	monoclinic
Space group	$P2_12_12_1$	
Unit cell dimensions	*a* = 12.38100(10) Å α = 90°	*a* = 12.4231Å α = 90°
	b = 15.93360(10) Å β = 90°	*b* = 17.6759 Å β = 95.467°
	c = 23.8962(2) Å γ = 90°	*c* = 21.3412 Å γ = 90°
Volume	4714.10(6) Å^3	4665.0 Å^3
Z	4	4
Density (calculated)	1.163 g cm^{-3}	1.250 g cm^{-3}
Absorption coefficient	3.037 mm^{-1}	4.047 mm^{-1}
F(000)	1784	1872
Crystal color, morphology	colourless, block	Colourless, needle
Crystal size	0.357 x 0.218 x 0.16 mm^3	0.38 x 0.12 x 0.07 mm^3
Theta range for data collection	3.334 to 78.369°	7.922 to 154.006°
Index ranges	$-15 \le h \le 12$, $-20 \le k \le 20$, $-30 \le l \le 29$	$-15 \le h \le 14$, $-21 \le k \le 22$, $-24 \le l \le 26$
Reflections collected	38695	41369
Independent reflections	9906 [*R*(int) = 0.0396]	9585 [*R*(int) = 0.0925]
Observed reflections	9541	6868
Completeness to theta = 74.504°	100.0%	97.1%
Absorption correction	Multi-scan	Gaussian
Max. and min. transmission	1.00000 and 0.33973	0.295 and 1.000
Refinement method	Full-matrix least-squares on F^2	Full-matrix least-squares on F^2
Data / restraints / parameters	9906 / 0 / 516	9585 / 0 / 505
Goodness-of-fit on F^2	1.051	1.025
Final *R* indices [*I*>2sigma(*I*)]	*R*1 = 0.0301, *wR*2 = 0.0782	*R*1 = 0.0593, *wR*2 = 0.1344
R indices (all data)	*R*1 = 0.0314, *wR*2 = 0.0790	*R*1 = 0.0858, *wR*2 = 0.1486
Absolute structure parameter	-0.0091(12)	
Largest diff. peak and hole	0.407 and -0.294 e.Å^{-3}	0.77 and -1.03 e.Å^{-3}

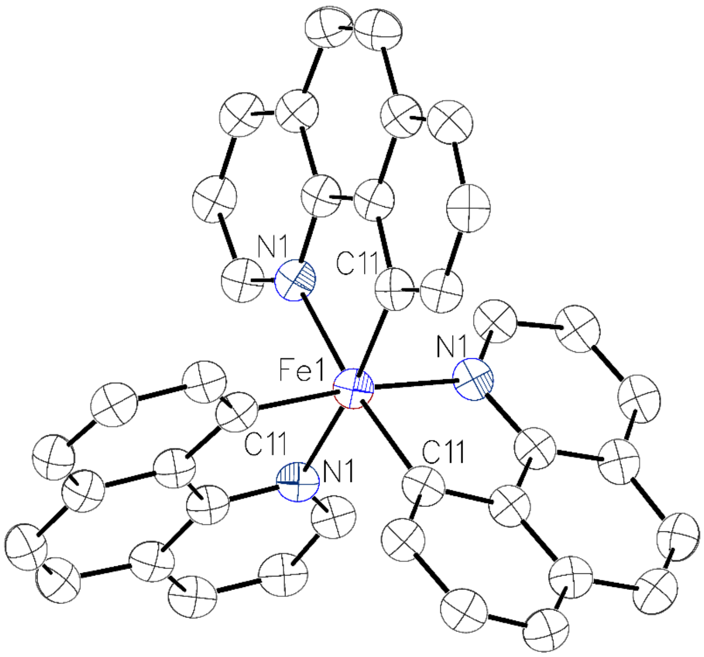

Figure S32. Crystal structure of **2-bzq**. Hydrogen atoms are omitted for clarity and the thermal ellipsoids are shown at 50% probability.

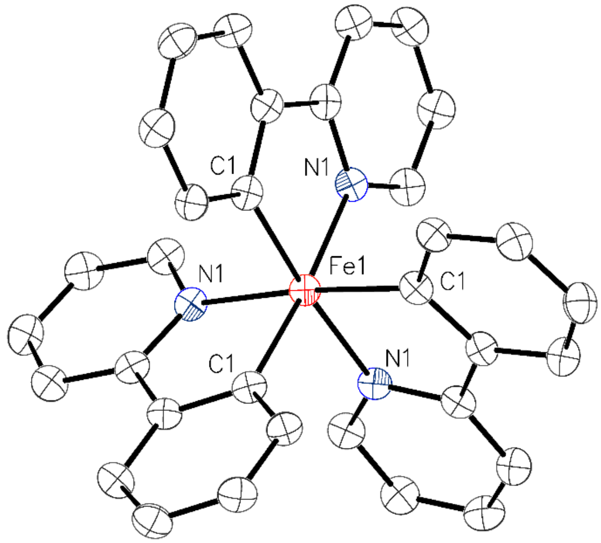

Figure S33. Crystal structure of **2-ppy**. Hydrogen atoms are omitted for clarity and the thermal ellipsoids are shown at 50% probability.

Table S6. Crystal data and structure refinement of **2-bzq** and **2-ppy**.

	**2-bzq**	**2-ppy**
Empirical formula	C39 H24 Fe N3	C33 H24 Fe N3
Formula weight	590.46	518.40
Temperature	100.00(10) K	100.01(10) K
Wavelength	1.54184 Å	1.54184 Å
Crystal system	trigonal	trigonal
Space group	*P*-3*c*1	*P*-3*c*1
Unit cell dimensions	*a* = 15.0670(6) Å α= 90°	*a* = 16.5115(2) Å α = 90°
	b = 15.0670(6) Å β = 90°	*b* = 16.5115(2) Å β = 90°
	c = 15.2096(6) Å γ = 120°	*c* = 15.46360(10) Å γ = 120°
Volume	2990.2(3) $Å^3$	3651.02(9) $Å^3$
Z	4	6
Density (calculated)	1.312 Mg/m^3	1.415 Mg/m^3
Absorption coefficient	4.286 mm^{-1}	5.177 mm^{-1}
F(000)	1220	1614
Crystal color, morphology	purple-black, needle	red-violet, needle
Crystal size	0.251 x 0.099 x 0.071 mm^3	0.309 x 0.051 x 0.035 mm^3
Theta range for data collection	3.387 to 77.925°	3.090 to 80.186°
Index ranges	-9 ≤ *h* ≤ 18, -19 ≤ *k* ≤ 11, -18 ≤ *l* ≤ 19	-21 ≤ *h* ≤ 21, -20 ≤ *k* ≤ 21, -19 ≤ *l* ≤ 14
Reflections collected	9024	29954
Independent reflections	2104 [*R*(int) = 0.0365]	2644 [*R*(int) = 0.0414]
Observed reflections	1860	2494
Completeness to theta = 74.504°	100.0%	99.8%
Absorption correction	Multi-scan	Multi-scan
Max. and min. transmission	1.00000 and 0.54887	1.00000 and 0.59775
Refinement method	Full-matrix least-squares on F^2	Full-matrix least-squares on F^2
Data / restraints / parameters	2104 / 0 / 130	2644 / 0 / 170
Goodness-of-fit on F^2	1.096	1.093
Final *R* indices [*I*>2sigma(*I*)]	*R*1 = 0.0389, *wR*2 = 0.1099	*R*1 = 0.0424, *wR*2 = 0.1145
R indices (all data)	*R*1 = 0.0433, *wR*2 = 0.1130	*R*1 = 0.0444, *wR*2 = 0.1166
Largest diff. peak and hole	0.227 and -0.435 e.$Å^{-3}$	2.123 and -0.335 e.$Å^{-3}$

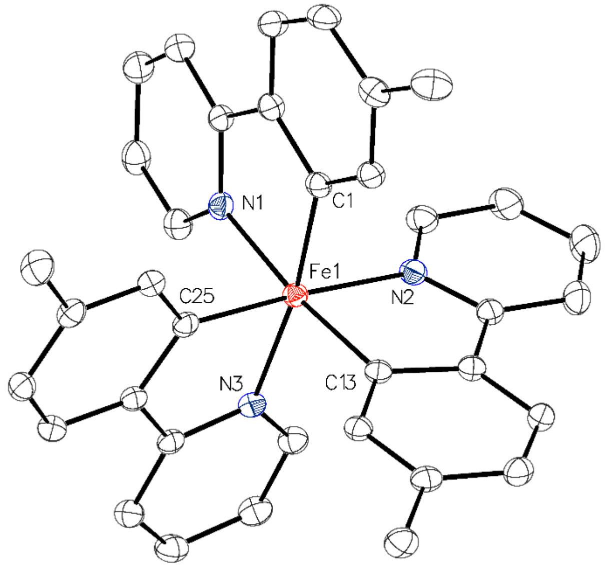

Figure S34. Crystal structure of **2-ptpy**. Hydrogen atoms and solvent molecules are omitted for clarity and the thermal ellipsoids are shown at 50% probability.

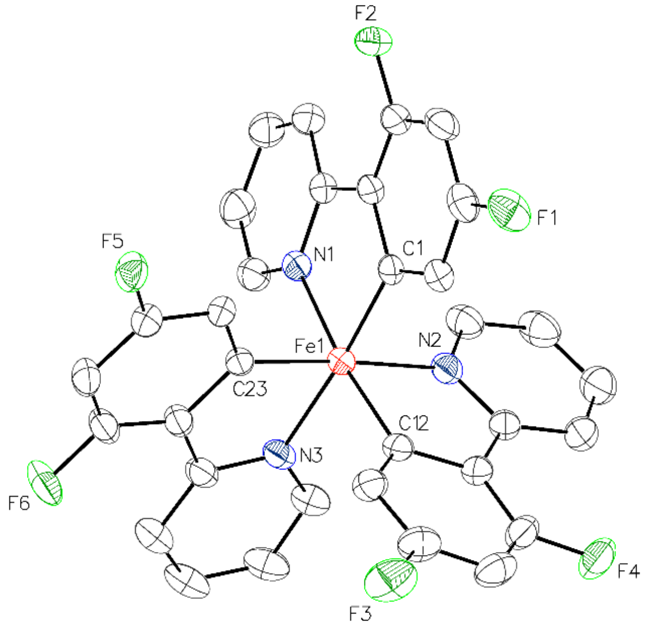

Figure S35. Crystal structure of **2-dfppy**. Hydrogen atoms are omitted for clarity and the thermal ellipsoids are shown at 50% probability.

Table S7. Crystal data and structure refinement of **2-ptpy** and **2-dfppy**.

	**2-ptpy**	**2-dfppy**
Empirical formula	C40 H38 Fe N3 O	C33 H18 F6 Fe N3
Formula weight	632.58	626.35
Temperature	100.00(10) K	99.99(10) K
Wavelength	1.54184 Å	1.54184 Å
Crystal system	monoclinic	monoclinic
Space group	$P2_1/n$	$P2_1/n$
Unit cell dimensions	*a* = 15.08620(10) Å α = 90°	*a* = 9.10500(10) Å α = 90°
	b = 9.95440(10) Å β= 91.4460(10)°	*b* = 23.3523(2) Å β= 97.9310(10)°
	c = 20.74630(10) Å γ= 90°	*c* = 12.21890(10) Å γ= 90°
Volume	3114.56(4) $Å^3$	2573.17(4) $Å^3$
Z	4	4
Density (calculated)	1.349 Mg/m^3	1.617 Mg/m^3
Absorption coefficient	4.167 mm^{-1}	5.361 mm^{-1}
F(000)	1332	1268
Crystal color, morphology	dark violet, plate	orange, needle
Crystal size	0.19 x 0.129 x 0.055 mm^3	0.133 x 0.041 x 0.019 mm^3
Theta range for data collection	3.580 to 80.207°	3.786 to 80.348°
Index ranges	-18 ≤ *h* ≤ 15, -12 ≤ *k* ≤ 12, -26 ≤ *l* ≤ 26	-11 ≤ *h* ≤ 11, -23 ≤ *k* ≤ 29, -15 ≤ *l* ≤ 15
Reflections collected	54363	41066
Independent reflections	6710 [*R*(int) = 0.0394]	5567 [*R*(int) = 0.0391]
Observed reflections	6236	4954
Completeness to theta = 74.504°	100.0%	100.0%
Absorption correction	Multi-scan	Multi-scan
Max. and min. transmission	1.00000 and 0.72581	1.00000 and 0.78666
Refinement method	Full-matrix least-squares on F^2	Full-matrix least-squares on F^2
Data / restraints / parameters	6710 / 0 / 409	5567 / 0 / 388
Goodness-of-fit on F^2	1.075	1.090
Final *R* indices [*I*>2sigma(*I*)]	*R*1 = 0.0349, *wR*2 = 0.0893	*R*1 = 0.0402, *wR*2 = 0.1096
R indices (all data)	*R*1 = 0.0379, *wR*2 = 0.0910	*R*1 = 0.0456, *wR*2 = 0.1128
Largest diff. peak and hole	0.422 and -0.390 e.$Å^{-3}$	0.573 and -0.351 e.$Å^{-3}$

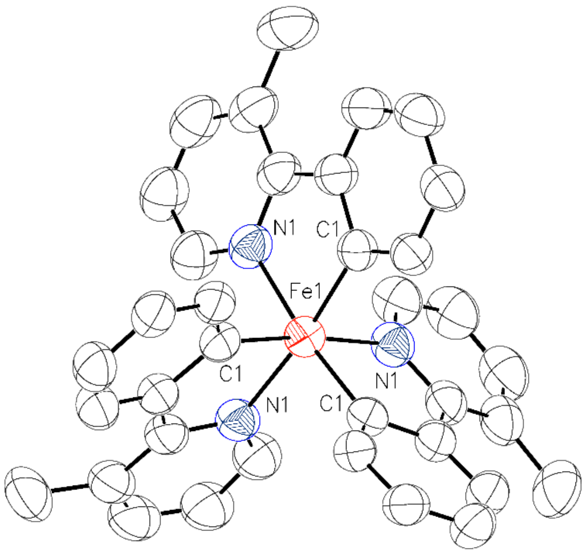

Figure S36. Crystal structure of **2-mppy**. Hydrogen atoms are omitted for clarity and the thermal ellipsoids are shown at 50% probability.

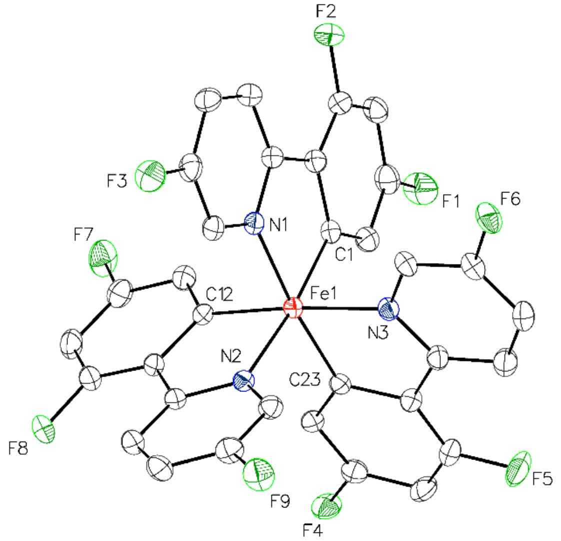

Figure S37. Crystal structure of **2-dfpfpy**. Hydrogen atoms are omitted for clarity and the thermal ellipsoids are shown at 50% probability.

Table S8. Crystal data and structure refinement of **2-mppy** and **2-dfpfpy**.

	**2-mppy**	**2-dfpfpy**
Empirical formula	C36 H30 Fe N3	C33 H15 F9 Fe N3
Formula weight	560.48	680.33
Temperature	173.00(10) K	100.00(10) K
Wavelength	1.54184 Å	1.54184 Å
Crystal system	trigonal	monoclinic
Space group	*R*-3	$P2_1/n$
Unit cell dimensions	*a* = 15.0750(4) Å α= 90°	*a* = 9.46120(10) Å α= 90°
	b = 15.0750(4) Å β= 90°	*b* = 23.5947(2) Å β= 98.1220(10)°
	c = 28.6238(8) Å γ= 120°	*c* = 12.11550(10) Å γ= 90°
Volume	5633.4(3) $Å^3$	2677.47(4) $Å^3$
Z	6	4
Density (calculated)	0.991 Mg/m^3	1.688 Mg/m^3
Absorption coefficient	3.384 mm^{-1}	5.375 mm^{-1}
F(000)	1758	1364
Crystal color, morphology	dark red, block	orange, plate
Crystal size	0.326 x 0.197 x 0.16 mm^3	0.119 x 0.08 x 0.032 mm^3
Theta range for data collection	3.721 to 81.301°	3.747 to 80.187°
Index ranges	$-17 \leq h \leq 18$, $-18 \leq k \leq 15$, $-36 \leq l \leq 36$	$-12 \leq h \leq 12$, $-29 \leq k \leq 29$, $-15 \leq l \leq 13$
Reflections collected	16014	33566
Independent reflections	2720 [*R*(int) = 0.0323]	5726 [*R*(int) = 0.0352]
Observed reflections	2384	5139
Completeness to theta = 74.504°	100.0%	99.9%
Absorption correction	Multi-scan	Multi-scan
Max. and min. transmission	1.00000 and 0.57851	1.00000 and 0.81725
Refinement method	Full-matrix least-squares on F^2	Full-matrix least-squares on F^2
Data / restraints / parameters	2720 / 0 / 122	5726 / 0 / 415
Goodness-of-fit on F^2	1.108	1.113
Final *R* indices [*I*>2sigma(*I*)]	*R*1 = 0.0383, *wR*2 = 0.1111	*R*1 = 0.0366, *wR*2 = 0.0933
R indices (all data)	*R*1 = 0.0431, *wR*2 = 0.1164	*R*1 = 0.0419, *wR*2 = 0.0957
Largest diff. peak and hole	0.317 and -0.211 e.$Å^{-3}$	0.341 and -0.458 e.$Å^{-3}$

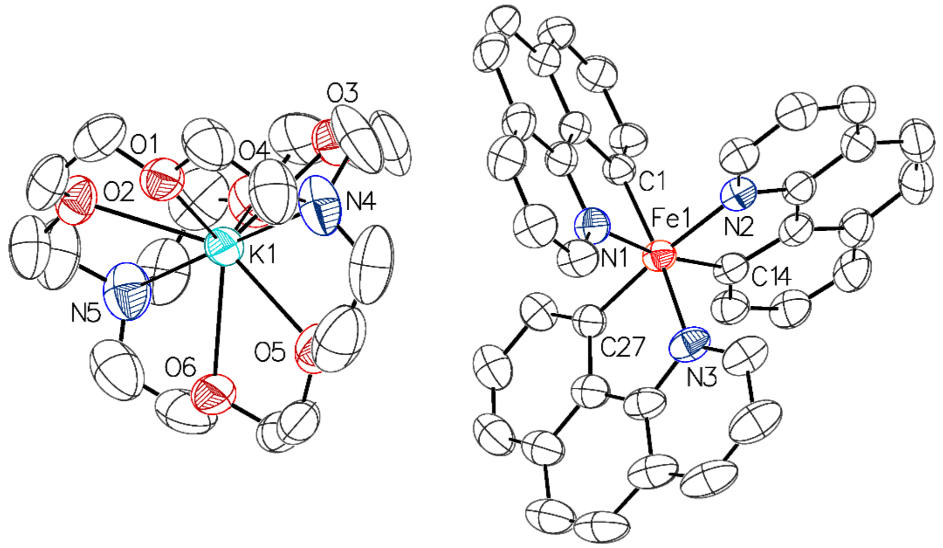

Figure S38. Crystal structure of **1-bzq**. Hydrogen atoms are omitted for clarity and the thermal ellipsoids are shown at 50% probability.

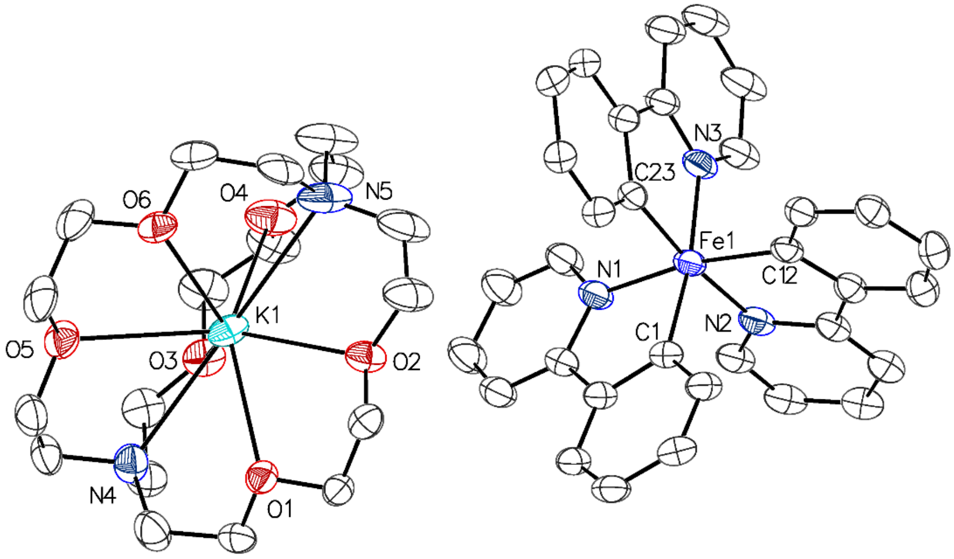

Figure S39. Crystal structure of **1-ppy**. Hydrogen atoms and solvent molecules are omitted for clarity and the thermal ellipsoids are shown at 50% probability.

Table S9. Crystal data and structure refinement of **1-bzq** and **1-ppy**.

	**1-bzq**	**1-ppy**
Empirical formula	C57 H60 Fe K N5 O6	C59 H76 Fe K N5 O8
Formula weight	1006.05	1078.19
Temperature	232.99(10) K	100.00(10) K
Wavelength	1.54184 Å	1.54184 Å
Crystal system	monoclinic	triclinic
Space group	$P2_1/n$	P-1
Unit cell dimensions	a = 12.6152(2) Å α= 90°	a = 12.9303(2) Å α= 91.8400(10)°
	b = 13.0318(2) Å β= 99.3230(10)°	b = 13.4933(2) Å β= 90.5310(10)°
	c = 31.1990(4) Å γ= 90°	c = 15.5169(2) Å γ= 93.0570(10)°
Volume	5061.33(13) $Å^3$	2701.86(7) $Å^3$
Z	4	2
Density (calculated)	1.320 Mg/m^3	1.325 Mg/m^3
Absorption coefficient	3.576 mm^{-1}	3.409 mm^{-1}
F(000)	2120	1148
Crystal color, morphology	green-black, needle	orange-black, plate
Crystal size	0.246 x 0.122 x 0.072 mm^3	0.3 x 0.12 x 0.033 mm^3
Theta range for data collection	2.871 to 77.879°	3.282 to 80.614°
Index ranges	$-15 \leq h \leq 15$, $-16 \leq k \leq 14$, $-39 \leq l \leq 39$	$-16 \leq h \leq 16$, $-17 \leq k \leq 14$, $-19 \leq l \leq 19$
Reflections collected	66201	91029
Independent reflections	10634 [R(int) = 0.0675]	11619 [R(int) = 0.0632]
Observed reflections	9158	10296
Completeness to theta = 74.504°	99.7%	99.8%
Absorption correction	Multi-scan	Multi-scan
Max. and min. transmission	1.00000 and 0.54361	1.00000 and 0.64132
Refinement method	Full-matrix least-squares on F^2	Full-matrix least-squares on F^2
Data / restraints / parameters	10634 / 0 / 631	11619 / 162 / 785
Goodness-of-fit on F^2	1.051	1.079
Final R indices [I>2sigma(I)]	$R1$ = 0.0464, $wR2$ = 0.1285	$R1$ = 0.0550, $wR2$ = 0.1542
R indices (all data)	$R1$ = 0.0531, $wR2$ = 0.1342	$R1$ = 0.0610, $wR2$ = 0.1603
Largest diff. peak and hole	0.463 and -0.374 e.$Å^{-3}$	0.677 and -0.748 e.$Å^{-3}$

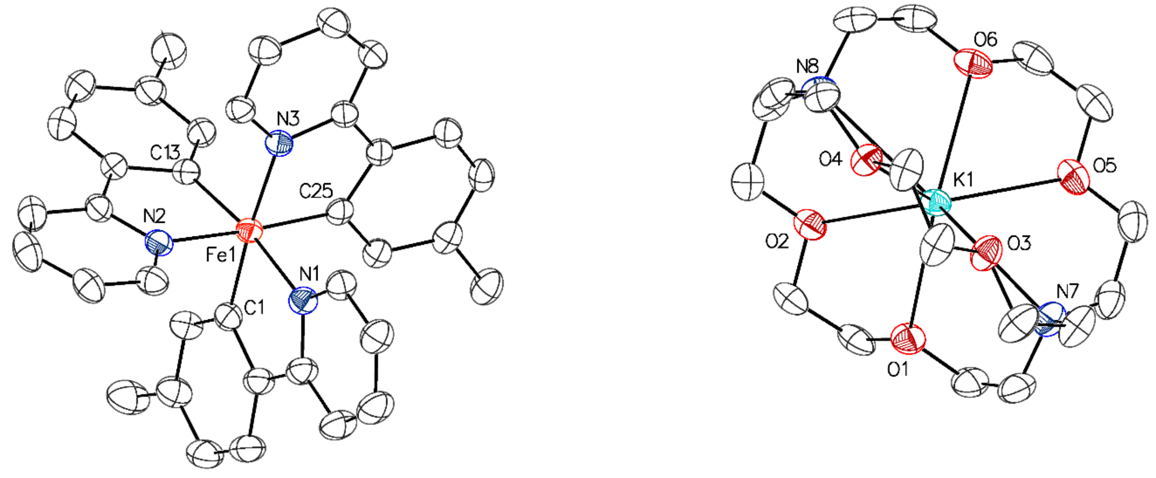

Figure S40. Crystal structure of **1-ptpy**. Hydrogen atoms and solvent molecules are omitted for clarity and the thermal ellipsoids are shown at 50% probability.

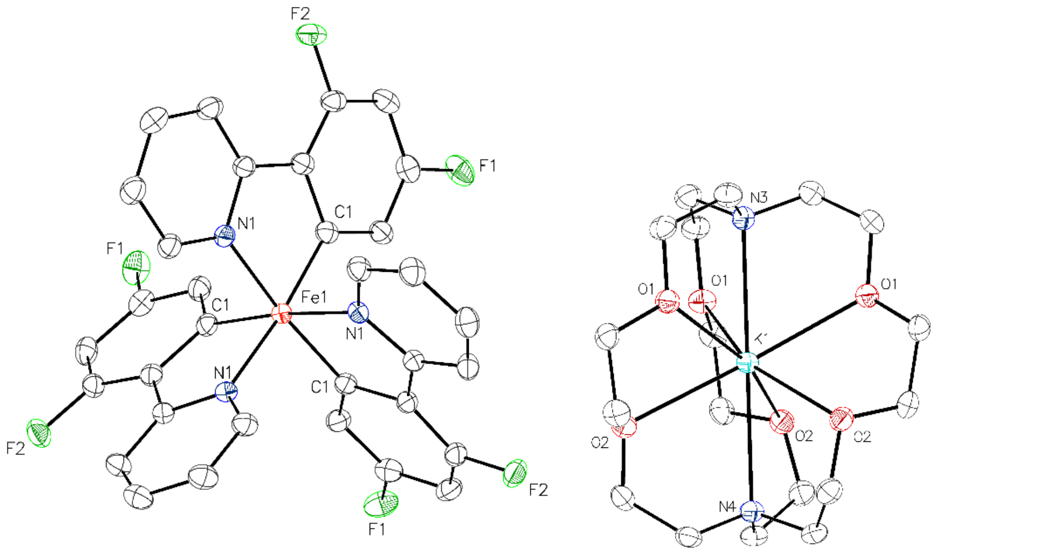

Figure S41. Crystal structure of **1-dfppy**. Hydrogen atoms are omitted for clarity and the thermal ellipsoids are shown at 50% probability.

Table S10. Crystal data and structure refinement of **1-ptpy** and **1-dfppy**.

	**1-ppy**	**1-dfppy**
Empirical formula	C62 H83 Fe K N5 O8	C51 H54 F6 Fe K N5 O6
Formula weight	1121.28	1041.94
Temperature	100.00(11) K	100.00(10) K
Wavelength	1.54184 Å	1.54184 Å
Crystal system	triclinic	trigonal
Space group	*P*-1	*R*3*c*
Unit cell dimensions	*a* = 17.7676(2) Å α= 114.6990(10)°	*a* = 14.66160(10) Å α= 90°
	b = 19.7393(2) Å β= 111.7550(10)°	*b* = 14.66160(10) Å β= 90°
	c = 20.0446(2) Å γ= 91.6040(10)°	*c* = 75.2940(6) Å γ= 120°
Volume	5791.03(12) Å^3	14017.0(2) Å^3
Z	4	12
Density (calculated)	1.286 Mg/m^3	1.481 Mg/m^3
Absorption coefficient	3.200 mm^{-1}	4.083 mm^{-1}
F(000)	2396	6504
Crystal color, morphology	dark blue, block	dark blue, block
Crystal size	0.142 x 0.109 x 0.06 mm^3	0.188 x 0.121 x 0.092 mm^3
Theta range for data collection	3.301 to 77.978°	3.674 to 80.403°
Index ranges	-22 ≤ *h* ≤ 22, -24 ≤ *k* ≤ 22, -25 ≤ *l* ≤ 25	-18 ≤ *h* ≤ 18, -18 ≤ *k* ≤ 18, -91 ≤ *l* ≤ 95
Reflections collected	89789	99250
Independent reflections	23903 [*R*(int) = 0.0335]	6745 [*R*(int) = 0.0438]
Observed reflections	20234	6574
Completeness to theta = 74.504°	99.7%	100.0%
Absorption correction	Multi-scan	Multi-scan
Max. and min. transmission	1.00000 and 0.88290	1.00000 and 0.85026
Refinement method	Full-matrix least-squares on F^2	Full-matrix least-squares on F^2
Data / restraints / parameters	23903 / 200 / 1559	6745 / 1 / 421
Goodness-of-fit on F^2	1.023	1.090
Final *R* indices [*I*>2sigma(*I*)]	*R*1 = 0.0490, *wR*2 = 0.1297	*R*1 = 0.0340, *wR*2 = 0.0978
R indices (all data)	*R*1 = 0.0583, *wR*2 = 0.1351	*R*1 = 0.0347, *wR*2 = 0.0984
Largest diff. peak and hole	0.545 and -0.615 e.Å^{-3}	0.297 and -0.802 e.Å^{-3}

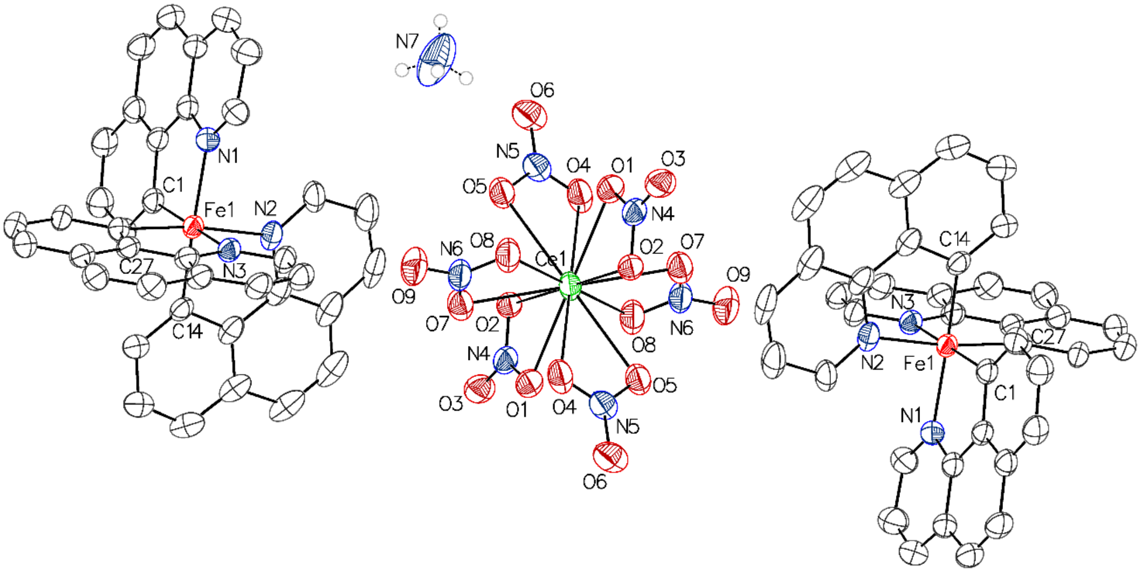

Figure S42. Crystal structure of **3-bzq**. Hydrogen atoms and solvent molecules are omitted for clarity and the thermal ellipsoids are shown at 50% probability.

Table S16. Crystal data and structure refinement of **3-bzq.**

	**3-bzq**
Empirical formula	C94 H92 Ce Fe2 N13 O26
Formula weight	2071.62
Temperature	100.00(10) K
Wavelength	1.54184 Å
Crystal system	triclinic
Space group	*P*-1
Unit cell dimensions	*a* = 11.9758(3) Å α= 105.165(2)°
	b = 13.1767(3) Å β= 106.901(3)°
	c = 16.3831(4) Å γ= 101.067(2)°
Volume	2284.72(10) $Å^3$
Z	1
Density (calculated)	1.506 Mg/m^3
Absorption coefficient	7.019 mm^{-1}
F(000)	1065
Crystal color, morphology	brown-green, plate
Crystal size	0.147 x 0.115 x 0.029 mm^3
Theta range for data collection	3.632 to 77.895°
Index ranges	$-15 \leq h \leq 12$, $-16 \leq k \leq 16$, $-20 \leq l \leq 20$
Reflections collected	53682
Independent reflections	9500 [*R*(int) = 0.0752]
Observed reflections	7795
Completeness to theta = 74.504°	99.2%
Absorption correction	Multi-scan
Max. and min. transmission	1.00000 and 0.53611
Refinement method	Full-matrix least-squares on F^2
Data / restraints / parameters	9500 / 127 / 680
Goodness-of-fit on F^2	1.052
Final *R* indices [*I*>2sigma(*I*)]	*R*1 = 0.0596, *wR*2 = 0.1608
R indices (all data)	*R*1 = 0.0717, *wR*2 = 0.1703
Largest diff. peak and hole	1.618 and -1.904 e.$Å^{-3}$

9. Density Functional Theory

9.1 Computation Details

All geometry optimizations of intermediates and transition states were achieved using spin- unrestricted UB3LYP[10]-D3[26]/6-31G(d)[27] method in THF solvent using the CPCM solvent model[28] with “opt=noeigen” and “guess=(mix,always)” keywords as implemented in Gaussian16[6]. Frequency calculations were also done for all the stationary points, and transition states were characterized by the presence of one unique imaginary frequency, which suggested that they were first-order saddle points on the potential energy surface. Intrinsic Reaction Coordinate (IRC) calculations were done on the transition states to verify that they were the correct transition state associated with the reaction. The endpoint geometries obtained from the IRC calculations were further optimized to verify the authenticity of the transition state. The thermochemistry: enthalpy (ΔH) and free energy (ΔG) were obtained at the temperature of 298 K. Energies were further corrected using single point calculations at UB3LYP-D3/def2SVP[14]-CPCM[28](THF) level of theory. All structural figures were generated with CYLview.[30] Distances in structural figures are shown in Å and energies are in kcal/mol.

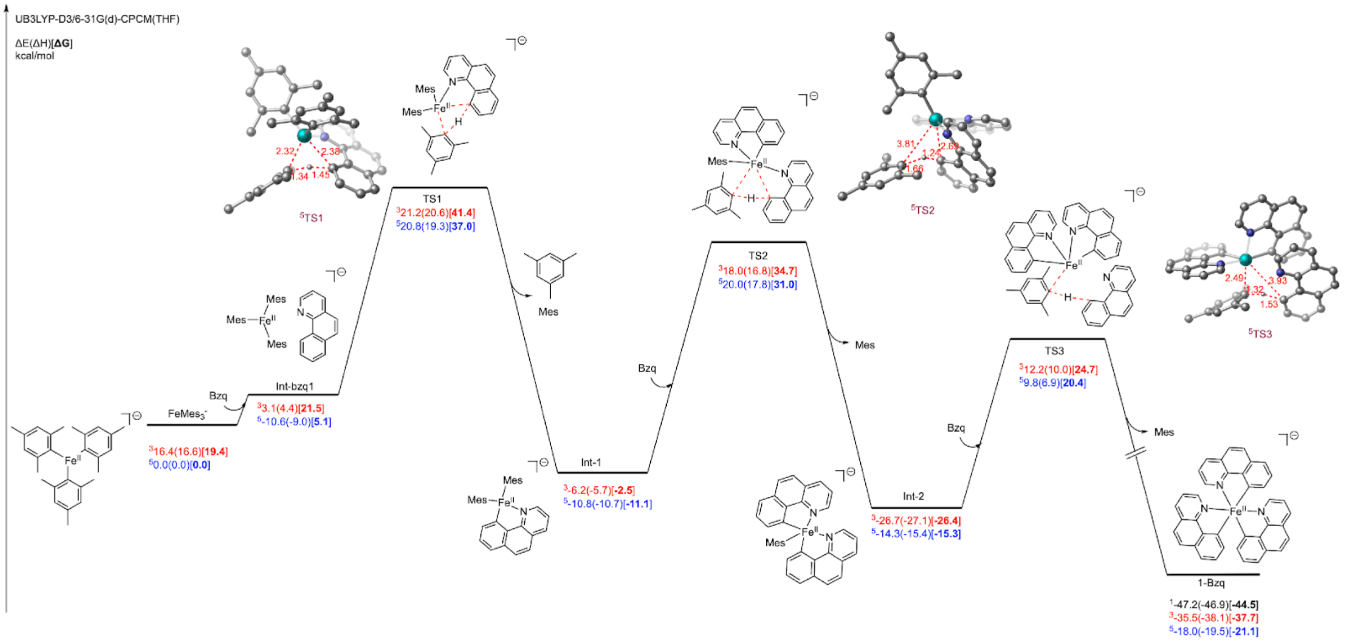

Figure S43. Gibbs free energy profile for full C-H activation pathways calculated at UB3LYP-D3-6-31G(d)-CPCM(THF) for triplet and quintet spin states.

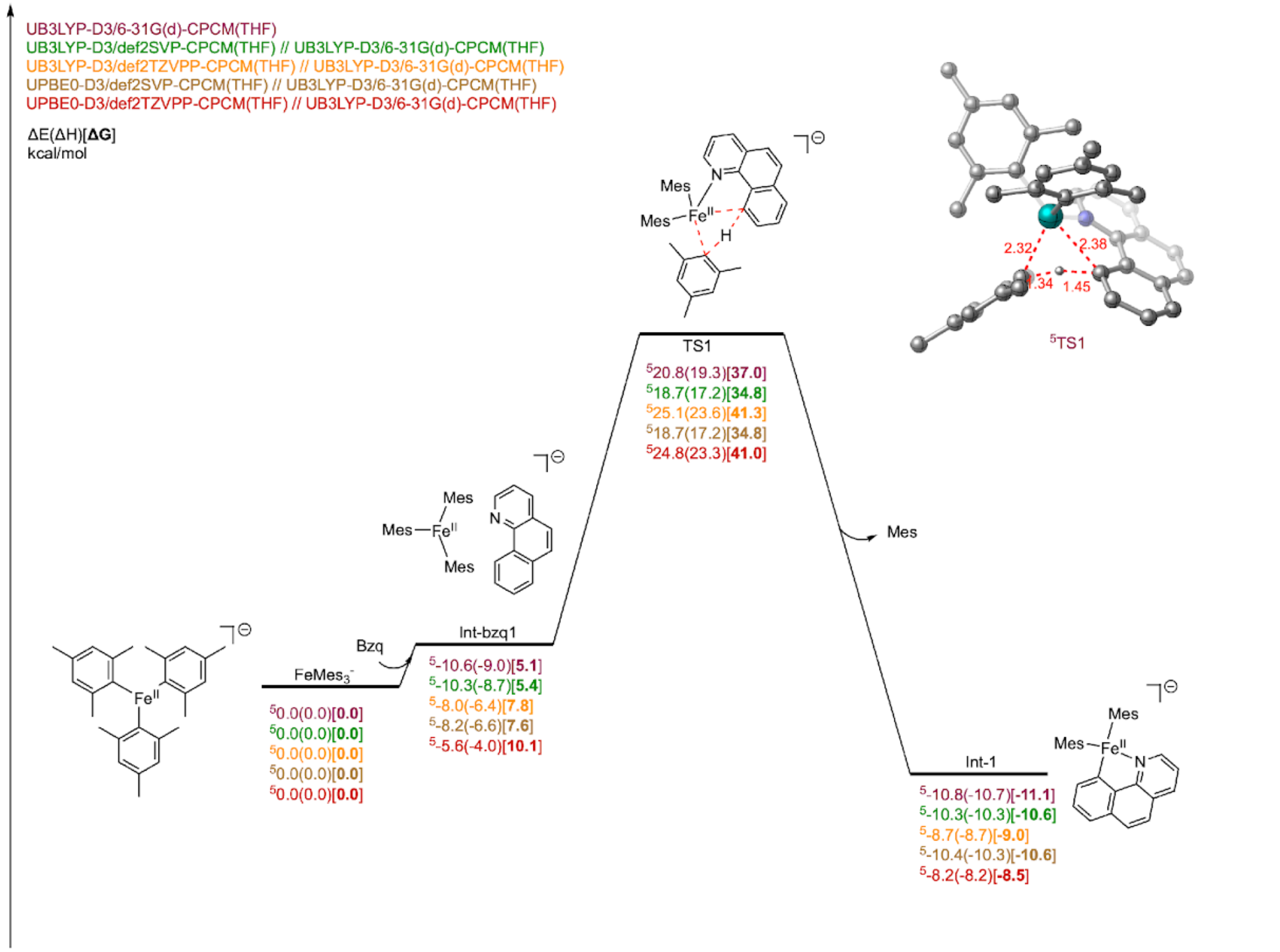

Figure S44. Gibbs free energy profile for first sp^2 C-H activation step calculated at five different levels of theory for quintet spin state (lowest energy spin state).

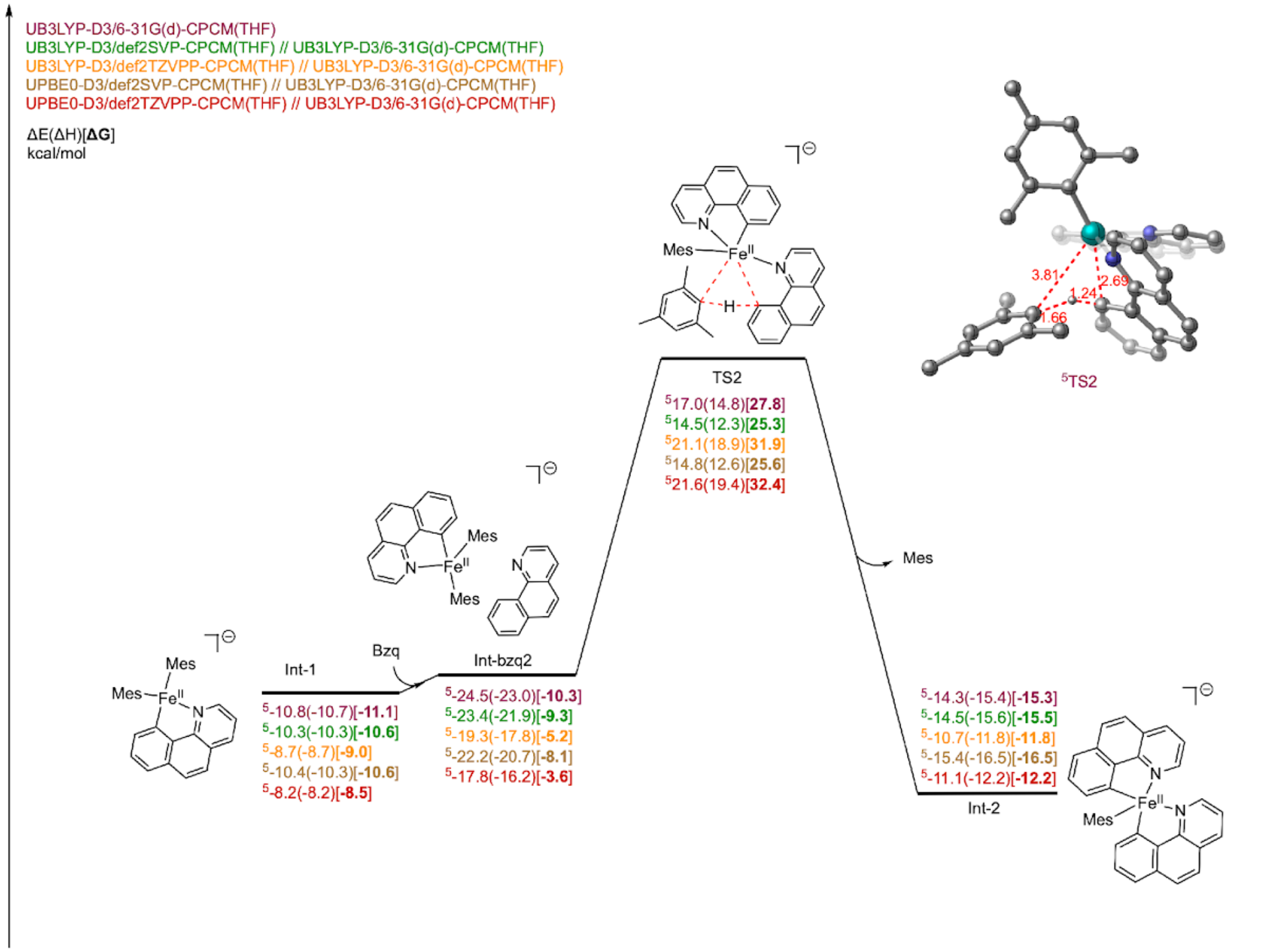

Figure S45. Gibbs free energy profile for second sp^2 C-H activation step calculated at five different levels of theory for quintet spin state (lowest energy spin state).

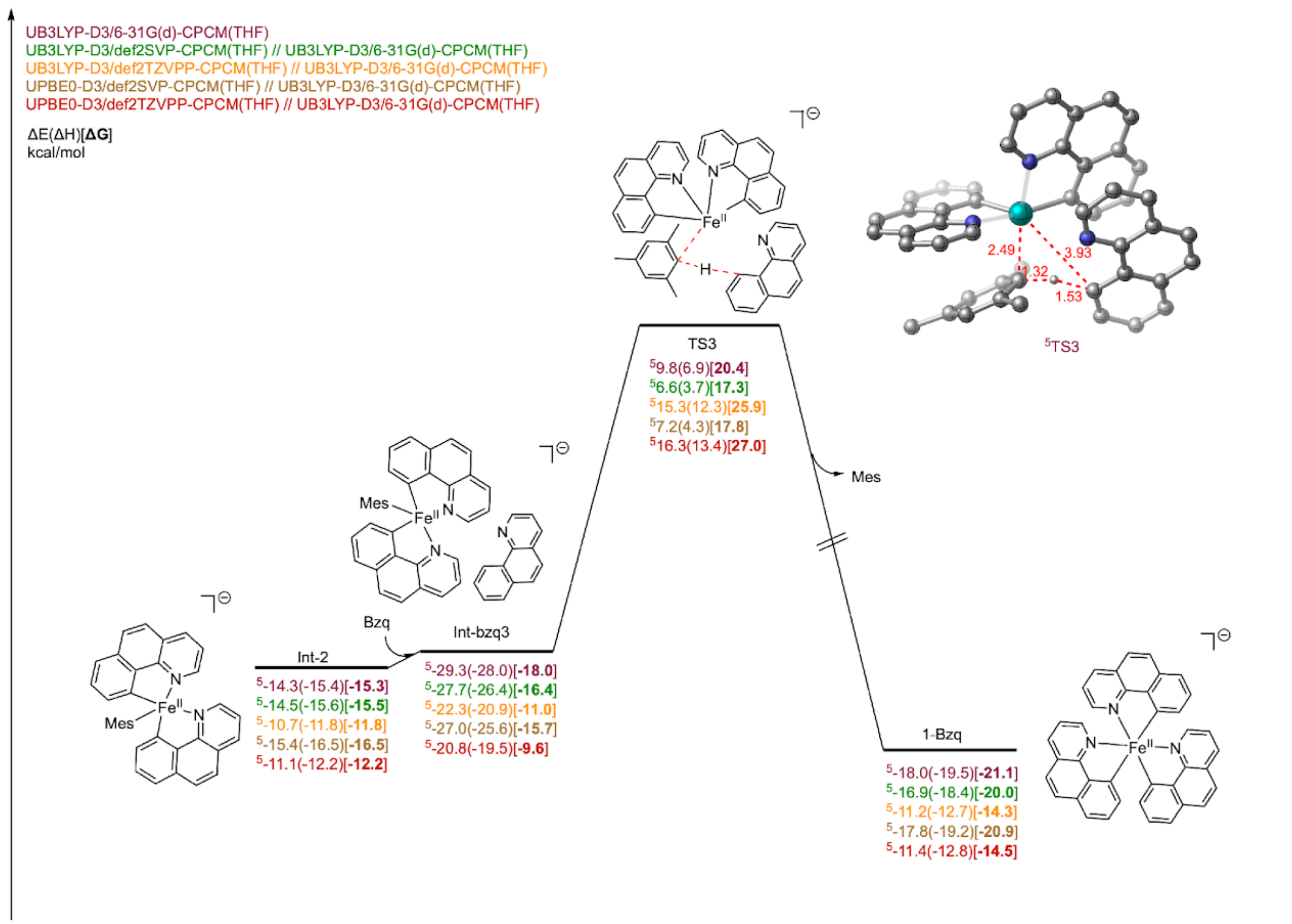

Figure S46. Gibbs free energy profile for third sp^2 C-H activation step calculated at five different levels of theory for quintet spin state (lowest energy spin state).

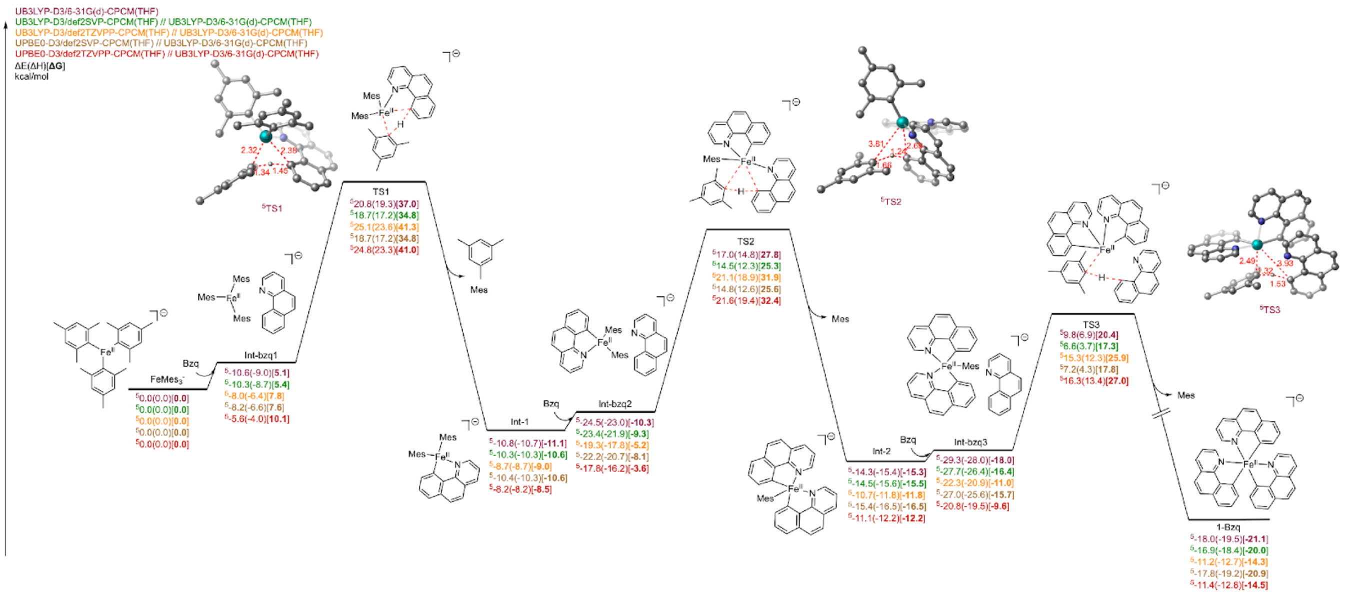

Figure S47. Gibbs free energy profile for the full sp^2 C-H activation pathway calculated at five different levels of theory for quintet spin state (lowest energy spin state).

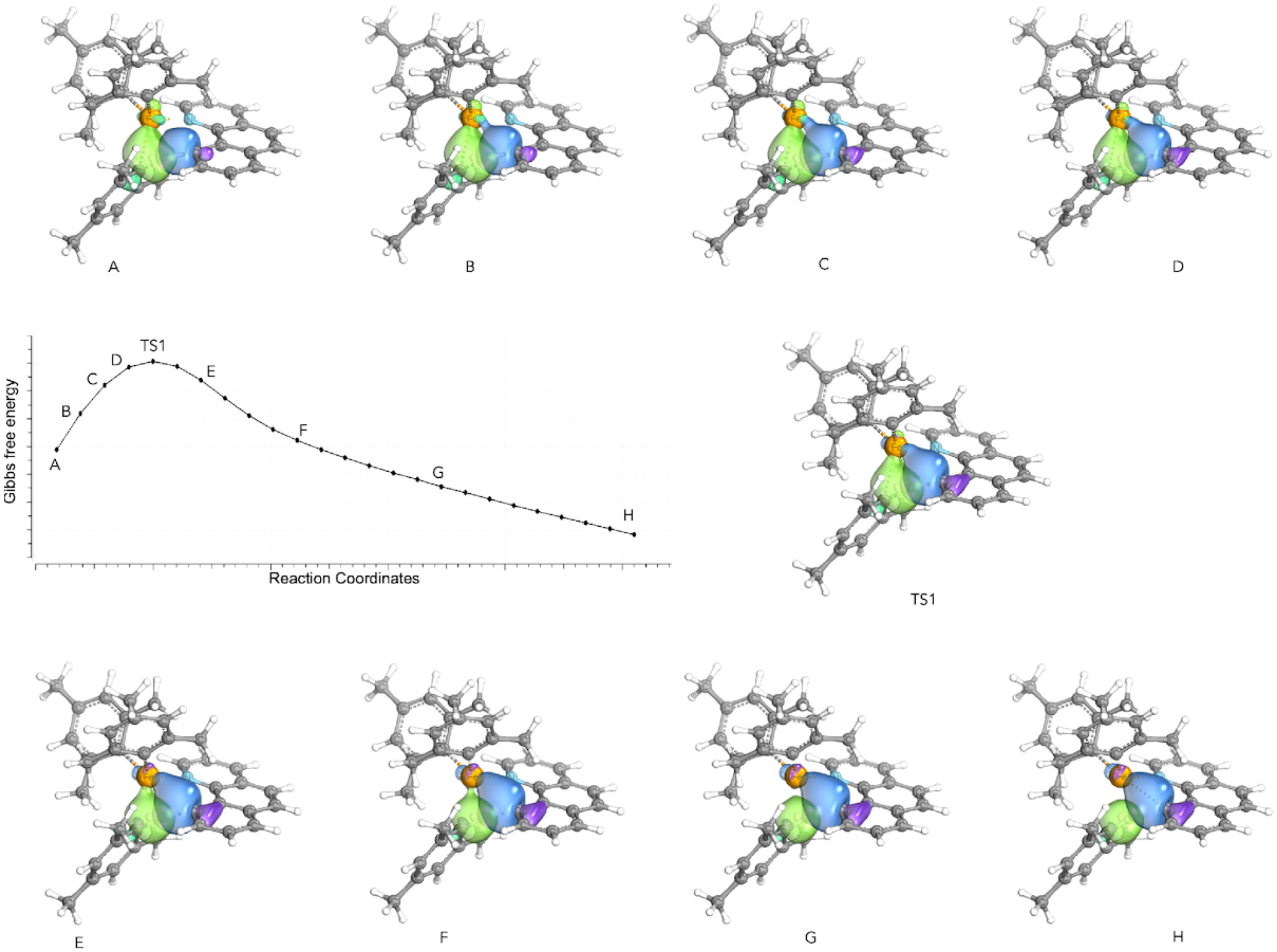

Figure S48. Intrinsic bond orbitals (IBO) analysis for the transition state of the first sp^2 C-H activation step **TS1** for quintet spin state (lowest energy spin state) along with its intrinsic reaction coordinates (IRC).

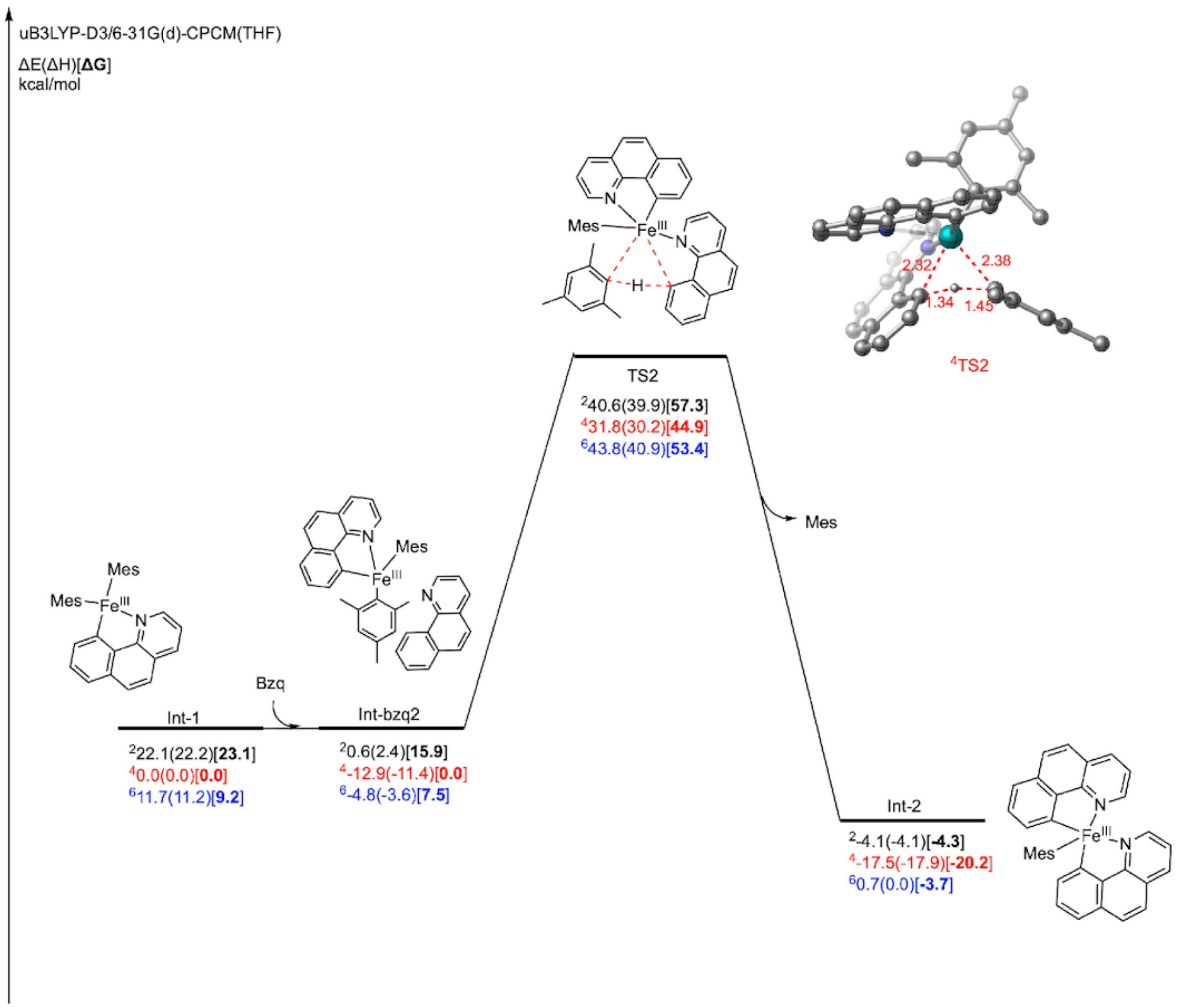

Figure S49. Gibbs free energy profile for the second sp^2 C-H activation step starting from the oxidized Fe(III)-intermediate **Int-1** calculated at UB3LYP-D3-6-31G(d)-CPCM(THF) for doublet, quartet and sextet spin states.

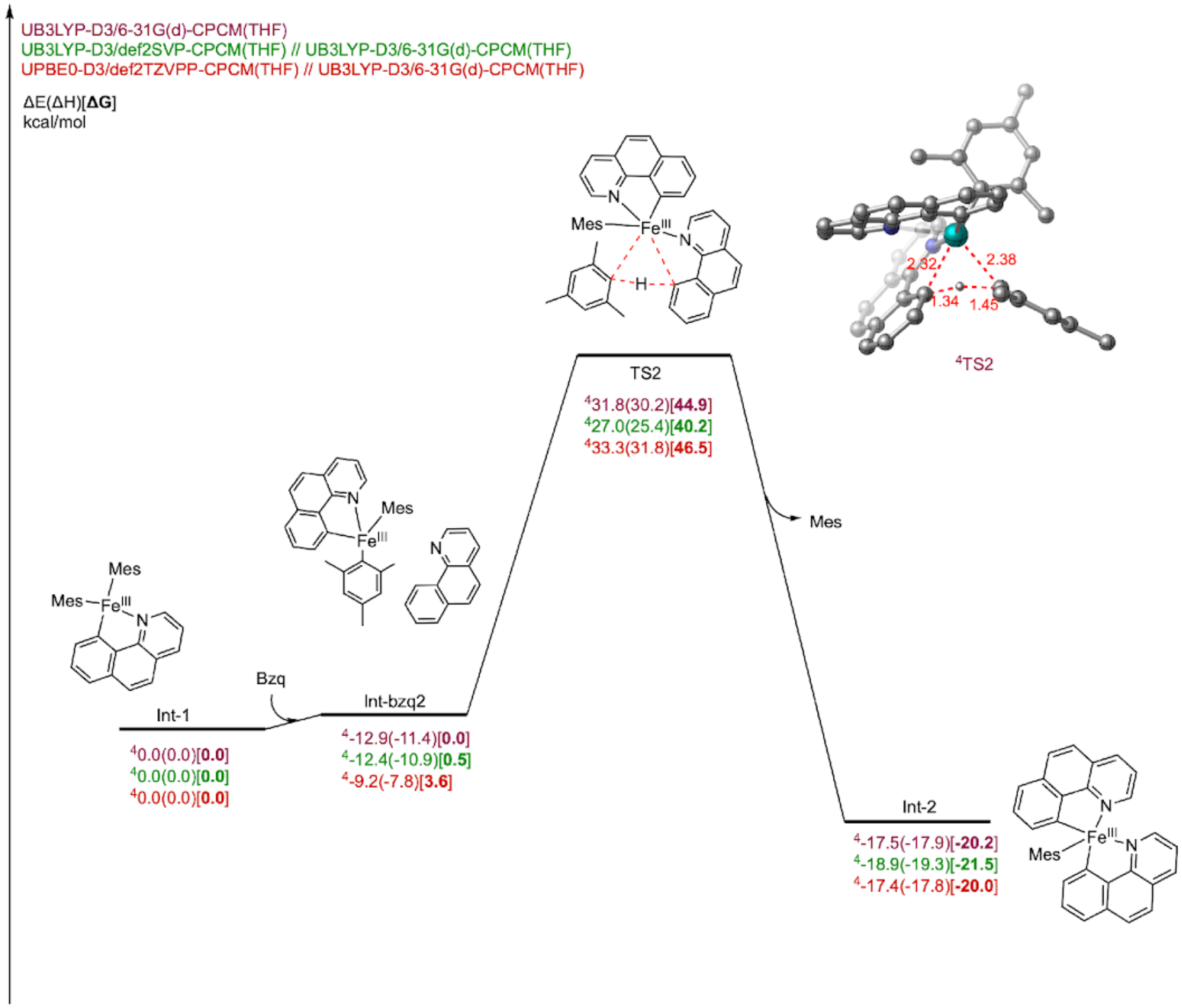

Figure S50. Gibbs free energy profile for the second sp^2 C-H activation step starting from the oxidized Fe(III)-intermediate **Int-1** at three different levels of theory for quartet spin state (lowest energy spin state).

9.2 Molecular Coordinates

Cartesian coordinates (xyz format) and energies of all the structures involved in each reaction mechanism studied calculated at the UB3LYP-D3/6-31-G(d)-CPCM(THF) level of theory.

Bzq

E(UB3LYP) = -555.602274562 A.U.

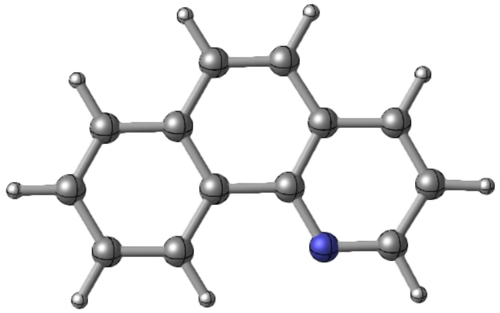

C	-5.072335	-1.880815	17.89566	H	-1.000249	-1.684563	17.892672
C	-3.690014	-1.907187	17.894494	H	-5.634674	-2.810431	17.894515
C	-2.942208	-0.705599	17.895961	H	-3.158092	-2.855413	17.89243
C	-3.633895	0.540727	17.898677	H	-5.562016	1.496515	17.90187
C	-5.046604	0.54254	17.899815	H	-6.840546	-0.629684	17.899238
C	-5.754183	-0.646327	17.898336	H	0.301771	0.41072	17.895243
C	-1.505368	-0.721946	17.89476	H	-0.904518	5.077055	17.904358
C	-2.865439	1.774035	17.900199	H	-3.399464	5.005152	17.906252
C	-1.442171	1.709369	17.898933	C	-0.727875	2.927641	17.900479
C	-0.78503	0.433802	17.896178	H	0.359204	2.909559	17.899579
C	-1.42022	4.122066	17.903115	N	-3.534213	2.954742	17.9028
C	-2.828748	4.077507	17.904177				

- Thermochemistry -

Temperature 298.150 Kelvin. Pressure 1.00000 Atm.

Zero-point correction= 0.183226 (Hartree/Particle)

Thermal correction to Energy= 0.192475

Thermal correction to Enthalpy= 0.193419

Thermal correction to Gibbs Free Energy= 0.148556

Sum of electronic and zero-point Energies= -555.419049

Sum of electronic and thermal Energies= -555.409800

Sum of electronic and thermal Enthalpies= -555.408856

Sum of electronic and thermal Free Energies= -555.453719

Charge = 0 Multiplicity = 1

Mes

E(UB3LYP) = -350.218135105 A.U.

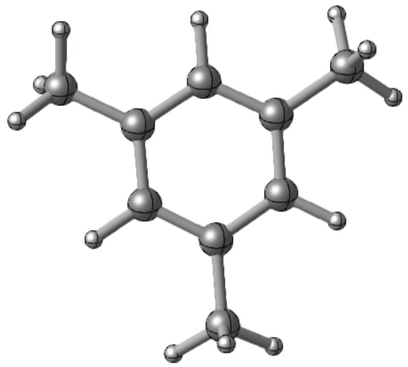

C	-0.750507	3.418193	-0.341011	C	-0.749631	5.82552	-0.363479
C	0.649501	3.400024	-0.341612	C	-1.465358	4.622165	-0.354433
C	1.334785	4.621041	-0.358363	H	-1.295316	2.475575	-0.334235
C	0.650512	5.842593	-0.364372	H	2.423537	4.620511	-0.365609

H	-1.293667	6.768542	-0.374337	H	1.598219	7.480335	0.686826
C	-2.976712	4.622109	-0.396004	H	2.379149	7.060995	-0.840037
H	-3.391325	5.516857	0.080742	C	1.405251	2.091322	-0.296398
H	-3.343225	4.606189	-1.43122	H	2.374676	2.170297	-0.800242
H	-3.39193	3.742662	0.107982	H	1.60221	1.784979	0.73976
C	1.406989	7.15149	-0.343492	H	0.838159	1.28327	-0.770921
H	0.843115	7.949343	-0.838699				

- Thermochemistry -

Temperature 298.150 Kelvin. Pressure 1.00000 Atm.

Zero-point correction= 0.183343 (Hartree/Particle)

Thermal correction to Energy= 0.193254

Thermal correction to Enthalpy= 0.194198

Thermal correction to Gibbs Free Energy= 0.146041

Sum of electronic and zero-point Energies= -350.034792

Sum of electronic and thermal Energies= -350.024881

Sum of electronic and thermal Enthalpies= -350.023937

Sum of electronic and thermal Free Energies= -350.072094

Charge = 0 Multiplicity = 1

$^{3}FeMes_3^{-}$

E(UB3LYP) = -2312.50877228 A.U.

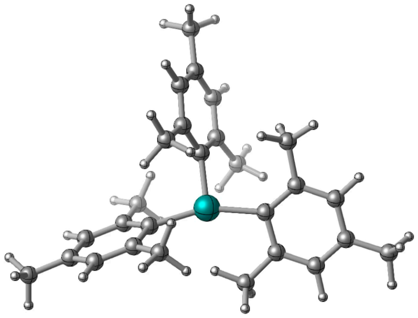

Fe	3.287957	4.204532	17.776792	C	-0.823106	3.779481	19.016147
C	0.163215	1.677013	19.569501	H	-1.686717	4.446184	18.996274
C	-0.967595	2.500143	19.560338	C	0.401841	4.221051	18.494776

C	1.393391	2.104185	19.048477	H	1.174753	6.25676	18.515298
C	5.28655	1.223151	15.518241	H	0.82443	5.645111	16.905912
C	4.524541	1.071622	14.353963	H	-0.503558	6.137737	17.973782
C	3.380647	1.867672	14.229873	C	-2.282598	2.036944	20.146163
H	2.774392	1.785033	13.326381	H	-2.412317	0.953397	20.037935
C	2.991731	2.767787	15.230622	H	-2.351736	2.261006	21.220573
C	4.924286	2.124654	16.527601	H	-3.134747	2.528733	19.662045
C	5.626732	7.492767	18.847753	C	2.569053	1.149206	19.123862
C	6.470797	7.87272	17.805583	H	3.066879	1.065231	18.153636
C	6.411829	7.132591	16.61359	H	3.327731	1.514039	19.830045
H	7.061176	7.423499	15.786254	H	2.267691	0.147695	19.455881
C	5.546774	6.043403	16.461143	H	0.079505	0.673566	19.990164
C	4.755673	6.400167	18.701641	C	1.560143	3.392247	18.471625
C	3.866663	6.032184	19.88415	C	3.749399	2.914101	16.419771
H	3.304028	6.895451	20.261217	C	4.907006	0.069054	13.288386
H	3.111093	5.264818	19.645972	H	5.994193	-0.066199	13.23374
H	4.448519	5.630366	20.724085	H	4.472254	-0.922831	13.48146
C	5.551175	5.295188	15.144098	H	4.557791	0.381989	12.29695
H	5.954062	4.283549	15.266213	C	1.759984	3.619768	15.016271
H	4.533998	5.15969	14.762286	H	1.057302	3.499367	15.848354
H	6.145672	5.814971	14.382376	H	2.023263	4.686974	14.987779
C	7.4139	9.047713	17.935874	H	1.245059	3.37772	14.078472
H	8.455044	8.750629	17.753444	C	5.832881	2.289717	17.726657
H	7.179272	9.835718	17.20773	H	6.205817	3.320497	17.776825
H	7.364599	9.493924	18.935233	H	5.29313	2.111969	18.664655
H	5.643215	8.053522	19.783745	H	6.691773	1.607934	17.691616
C	4.686401	5.633784	17.516268	H	6.194623	0.629803	15.636528
C	0.471484	5.635984	17.943397				

- Thermochemistry -

Temperature 298.150 Kelvin. Pressure 1.00000 Atm.

Zero-point correction= 0.517654 (Hartree/Particle)
Thermal correction to Energy= 0.549995
Thermal correction to Enthalpy= 0.550939
Thermal correction to Gibbs Free Energy= 0.450370
Sum of electronic and zero-point Energies= -2311.991118
Sum of electronic and thermal Energies= -2311.958778
Sum of electronic and thermal Enthalpies= -2311.957834
Sum of electronic and thermal Free Energies= -2312.058403

Charge = -1 Multiplicity = 3

$^5FeMes_3^-$

E(UB3LYP) = -2312.53483045 A.U..

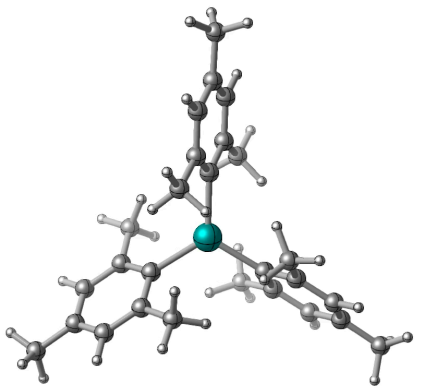

Fe	3.405605	3.862411	17.269041	C	3.812486	1.174806	13.832865
C	0.151071	2.460604	19.889681	H	3.265569	1.014257	12.902297
C	-0.881186	3.38512	19.698181	C	3.386196	2.163737	14.730895
C	-0.662702	4.428503	18.789631	C	5.198105	1.603666	16.203225
H	-1.458646	5.15477	18.617688	C	5.65733	7.001237	19.320952
C	0.550461	4.549053	18.098707	C	5.883132	8.005173	18.370655
C	1.3693	2.575034	19.202872	C	5.401989	7.794596	17.074247
C	5.624173	0.619287	15.299849	H	5.573701	8.560485	16.316162
C	4.937248	0.385967	14.102625	C	4.708838	6.622442	16.734835

C	4.96963	5.826469	18.988411	C	1.609634	3.625277	18.284529
C	4.755851	4.763393	20.051145	C	4.062942	2.41423	15.951296
H	5.13342	5.069078	21.034397	C	5.377472	-0.70155	13.147829
H	3.690904	4.518485	20.149207	H	6.460802	-0.864381	13.194692
H	5.259744	3.826734	19.775768	H	4.898858	-1.66381	13.380139
C	4.184561	6.463765	15.319125	H	5.117002	-0.457446	12.111087
H	4.351275	5.442596	14.953341	C	2.166348	3.000863	14.390491
H	3.09886	6.63153	15.280593	H	1.433655	2.973962	15.207597
H	4.65135	7.164137	14.615804	H	2.437796	4.057241	14.258731
C	6.599057	9.284615	18.742803	H	1.667295	2.668006	13.472122
H	7.089088	9.739194	17.873848	C	5.972448	1.810887	17.492031
H	5.904406	10.033948	19.148722	H	6.269186	2.861658	17.606969
H	7.363416	9.110744	19.509638	H	5.350833	1.57265	18.365742
H	6.032072	7.141042	20.336176	H	6.876834	1.192638	17.546697
C	4.468414	5.596941	17.681305	H	6.506976	0.019558	15.527584
C	0.739492	5.691151	17.116739				
H	1.637682	6.271661	17.361216				
H	0.892477	5.309529	16.097887				
H	-0.119574	6.372449	17.093194				
C	-2.180926	3.277956	20.464449				
H	-2.389829	2.24315	20.759977				
H	-2.158204	3.877321	21.38583				
H	-3.029787	3.638201	19.871034				
C	2.462617	1.555241	19.46508				
H	2.936248	1.244232	18.525613				
H	3.261501	1.985845	20.085422				
H	2.090194	0.66164	19.980536				
H	-0.001749	1.634706	20.586225				

- Thermochemistry -

Temperature 298.150 Kelvin. Pressure 1.00000 Atm.

Zero-point correction= 0.516294 (Hartree/Particle)
Thermal correction to Energy= 0.549541
Thermal correction to Enthalpy= 0.550486
Thermal correction to Gibbs Free Energy= 0.445502
Sum of electronic and zero-point Energies= -2312.018536
Sum of electronic and thermal Energies= -2311.985289
Sum of electronic and thermal Enthalpies= -2311.984345
Sum of electronic and thermal Free Energies= -2312.089328

Charge = -1 Multiplicity = 5

^{3}Int-bzq1

E(UB3LYP) = -2868.13222979 A.U.

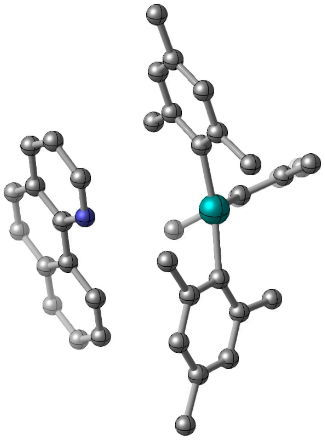

Fe	3.002891	3.995911	17.30331	C	0.567541	3.459573	18.608037
C	-0.546	3.516877	19.459959	C	6.133273	1.238351	15.954051
C	-0.976062	4.734211	19.990172	C	5.548307	0.585171	14.864518
C	-0.283907	5.891064	19.60547	C	4.288727	1.030489	14.455696
H	-0.625293	6.854678	19.986938	H	3.813913	0.555563	13.595544
C	0.825968	5.842714	18.750265	C	3.625626	2.073733	15.120277

C	5.486155	2.284905	16.62639	H	2.134219	5.1847	24.441401
C	5.46798	7.207448	18.59361	H	4.369365	4.208361	24.872157
C	5.708748	7.965351	17.441709	H	0.848284	4.504125	22.404803
C	5.132168	7.515504	16.249122	H	7.199616	1.08646	22.649793
H	5.288763	8.090993	15.335275	H	5.700042	-0.842795	18.486398
C	4.360718	6.346945	16.19428	H	3.458447	0.208753	18.169621
C	4.689638	6.043102	18.566609	N	3.507541	1.28777	19.913482
H	5.889343	7.541071	19.543366	H	1.799776	2.863457	20.83694
C	4.12381	5.566935	17.354046	C	1.500619	7.151183	18.391891
H	-1.077102	2.600158	19.721294	H	2.404525	7.312681	18.990914
C	1.314094	4.607563	18.245601	H	1.834001	7.153459	17.350685
C	4.193536	2.740484	16.244287	H	0.831835	8.006115	18.553838
H	7.124577	0.924321	16.284857	C	0.967448	2.101223	18.053071
C	2.552037	4.455815	23.752027	H	1.976221	2.09607	17.612995
C	3.801018	3.912199	23.993582	H	0.98966	1.328959	18.828307
C	4.365181	2.96758	23.102619	H	0.280118	1.771837	17.262789
C	3.624598	2.579159	21.949052	C	-2.132173	4.804349	20.962493
C	2.34971	3.143485	21.726026	H	-2.697701	5.736989	20.851034
C	1.822335	4.068054	22.609157	H	-2.826276	3.967353	20.824683
C	5.666565	2.403308	23.333239	H	-1.78269	4.764533	22.004181
C	4.211581	1.62852	21.021733	C	2.25572	2.478214	14.599873
C	5.504166	1.094619	21.288251	H	2.031713	2.009996	13.633296
C	6.213058	1.505046	22.466334	H	1.455467	2.188229	15.292274
C	6.039899	0.175714	20.357307	H	2.178962	3.563366	14.476743
H	7.026616	-0.246471	20.532212	C	6.2072	2.921091	17.796743
C	5.311021	-0.160904	19.234995	H	7.249348	2.585291	17.867743
C	4.044837	0.431851	19.05807	H	6.18911	4.01121	17.72258
H	6.213697	2.712802	24.220438	H	5.718751	2.663892	18.742974

C	6.242549	-0.565898	14.171975	C	4.413807	5.3137	19.861123
H	7.332676	-0.446128	14.18517	H	4.736484	4.269282	19.813343
H	6.021766	-1.526944	14.658917	H	4.913504	5.782562	20.718218
H	5.925944	-0.657447	13.126172	H	3.337704	5.282771	20.064613
C	3.755777	5.925996	14.873406	C	6.580451	9.200512	17.480217
H	4.037261	6.597555	14.052984	H	6.294088	9.92048	16.703938
H	4.065388	4.905679	14.619101	H	6.515716	9.709518	18.449786
H	2.657956	5.909075	14.932607	H	7.641732	8.960964	17.317143

- Thermochemistry -

Temperature 298.150 Kelvin. Pressure 1.00000 Atm.

Zero-point correction= 0.702455 (Hartree/Particle)
Thermal correction to Energy= 0.745057
Thermal correction to Enthalpy= 0.746002
Thermal correction to Gibbs Free Energy= 0.623381
Sum of electronic and zero-point Energies= -2867.429775
Sum of electronic and thermal Energies= -2867.387172
Sum of electronic and thermal Enthalpies= -2867.386228
Sum of electronic and thermal Free Energies= -2867.508848

Charge = -1 Multiplicity = 3

^{5}Int-bzq1

E(UB3LYP) = -2868.15402620 a.u.

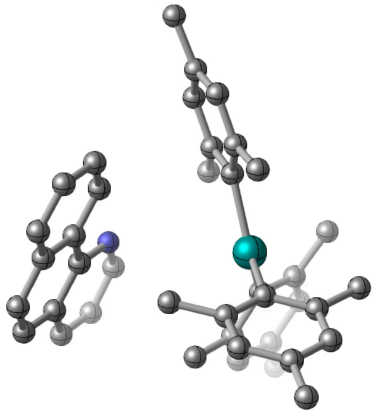

Fe	3.239247	4.204115	17.431677	C	5.821977	1.015738	15.800208
C	-0.635469	2.8746	19.149543	C	5.140345	0.526391	14.678661
C	-1.184961	4.019434	19.727319	C	3.933239	1.141193	14.333097
C	-0.503783	5.227418	19.535868	H	3.391284	0.785935	13.454959
H	-0.922149	6.141258	19.960866	C	3.409318	2.199323	15.092464
C	0.701768	5.282596	18.824579	C	5.306085	2.071975	16.562858
C	0.568665	2.916653	18.424592	C	6.013625	7.395069	18.768748

C	6.144488	8.223278	17.64886	H	0.847564	4.882949	22.050085
C	5.414492	7.884742	16.50304	H	7.268662	1.654037	22.678859
H	5.492401	8.520326	15.619121	H	5.602868	-1.155986	19.130171
C	4.590593	6.750904	16.477677	H	3.310831	-0.236727	18.729972
C	5.189448	6.258735	18.747262	N	3.427119	1.19903	20.189251
H	6.565508	7.645348	19.676637	H	1.736357	2.944636	20.8271
C	4.451503	5.889087	17.595399	C	1.383007	6.633727	18.699295
H	-1.154871	1.922016	19.268153	H	2.279727	6.686523	19.328201
C	1.298553	4.122394	18.254502	H	1.731219	6.814974	17.677539
C	4.068344	2.689046	16.246451	H	0.716196	7.454653	18.991476
H	6.773079	0.560115	16.080796	C	1.046495	1.606009	17.825355
C	2.61178	5.128274	23.292351	H	2.09503	1.661823	17.530121
C	3.87874	4.654057	23.580801	H	0.932412	0.776211	18.533601
C	4.410828	3.5434	22.884624	H	0.467771	1.347687	16.926413
C	3.619202	2.909424	21.883159	C	-2.45117	3.959507	20.551601
C	2.326523	3.40755	21.608103	H	-3.068433	4.854845	20.409329
C	1.83332	4.501458	22.298365	H	-3.059006	3.084403	20.294521
C	5.736457	3.059815	23.156182	H	-2.227753	3.893982	21.626001
C	4.180295	1.78622	21.151859	C	2.110941	2.845198	14.644565
C	5.501721	1.343559	21.449648	H	1.49624	2.172318	14.033605
C	6.261993	2.007433	22.469438	H	1.522909	3.165993	15.511113
C	6.013553	0.255541	20.708472	H	2.304156	3.745467	14.043412
H	7.019945	-0.102097	20.912431	C	6.090986	2.565456	17.762905
C	5.235753	-0.32846	19.728745	H	7.066754	2.073948	17.857261
C	3.943821	0.187881	19.506707	H	6.246238	3.649441	17.707517
H	6.320876	3.561112	23.923918	H	5.539814	2.379338	18.69152
H	2.218277	5.988335	23.827792	C	5.680548	-0.64408	13.887385
H	4.487454	5.136306	24.342088	H	6.776851	-0.63931	13.857996

H	5.377754	-1.604932	14.327834
H	5.314691	-0.635233	12.854016
C	3.80403	6.436065	15.21738
H	4.021932	7.128298	14.394927
H	4.011565	5.415331	14.870069
H	2.722159	6.478063	15.410244
C	5.058512	5.429956	20.009273
H	5.153342	4.360784	19.794714
H	5.80902	5.688704	20.766275
H	4.071891	5.556392	20.473949
C	7.065943	9.422875	17.66337
H	6.69433	10.224754	17.014028
H	7.178773	9.832204	18.674345
H	8.074139	9.165643	17.307524

- Thermochemistry -

Temperature   298.150 Kelvin. Pressure   1.00000 Atm.

Zero-point correction=   0.701570 (Hartree/Particle)
Thermal correction to Energy=   0.745533
Thermal correction to Enthalpy=   0.746478
Thermal correction to Gibbs Free Energy=   0.619178
Sum of electronic and zero-point Energies=   -2867.452456
Sum of electronic and thermal Energies=   -2867.408493
Sum of electronic and thermal Enthalpies=   -2867.407549
Sum of electronic and thermal Free Energies=   -2867.534848

Charge = -1 Multiplicity = 5

^{3}TS1

E(UB3LYP) = -2868.10338580   A.U.

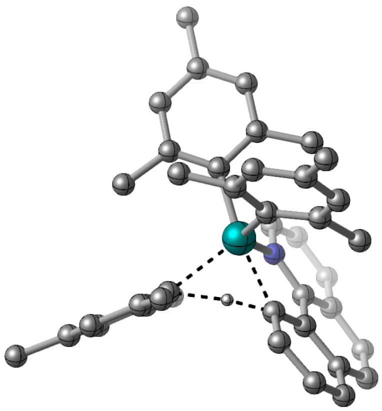

Fe	3.699515	3.731444	18.648443	C	5.884937	2.096242	15.277924
C	-0.742699	2.45133	19.005261	C	4.952171	1.712704	14.313494
C	-1.502167	3.594653	18.728043	C	3.612665	1.94437	14.616913
C	-0.816839	4.803089	18.641199	H	2.853647	1.656718	13.887965
H	-1.378926	5.720376	18.459107	C	3.201608	2.533322	15.827133
C	0.579459	4.880374	18.795665	C	5.503839	2.688305	16.491038
C	0.648217	2.503881	19.145959	C	6.378117	7.246732	18.643998

C	5.915205	8.068838	17.61614	H	-1.253386	1.493299	19.109645
C	4.842212	7.588589	16.863401	C	1.367441	3.721968	18.993626
H	4.471916	8.188347	16.030121	C	4.135883	2.946801	16.822619
C	4.237117	6.351775	17.134865	C	5.37539	1.051949	13.021247
C	5.776342	6.01348	18.945199	H	6.312665	1.475287	12.639646
C	6.422934	5.233987	20.078518	H	5.54388	-0.026853	13.151198
H	6.208959	4.168507	20.009158	H	4.612278	1.166663	12.242564
C	3.142262	5.919496	16.179944	C	1.700779	2.671495	15.99397
H	3.551891	5.310853	15.365011	H	1.295207	1.896407	16.653065
H	2.39962	5.29826	16.676256	H	1.414587	3.619527	16.446143
H	2.630584	6.782015	15.73366	H	1.189197	2.578247	15.027802
C	6.536391	9.419187	17.338742	C	6.638245	3.048902	17.430278
H	7.593227	9.443961	17.630433	H	6.752102	4.132743	17.514346
H	6.473776	9.681307	16.275615	H	6.459517	2.680188	18.443398
H	6.031818	10.222084	17.89563	H	7.590497	2.628665	17.084335
H	7.237235	7.573638	19.232834	H	6.945102	1.932232	15.079376
C	4.652873	5.523214	18.2169	C	3.584078	5.884097	22.677114
C	1.169483	6.277536	18.76417	C	4.097422	4.893841	23.507171
H	2.257417	6.258488	18.748245	C	4.361987	3.61393	22.981051
H	0.832072	6.836058	17.88176	C	4.103467	3.380676	21.599793
H	0.847664	6.852556	19.643505	C	3.555061	4.376863	20.737583
C	-2.998538	3.509662	18.532282	C	3.296621	5.620884	21.328733
H	-3.467128	2.860626	19.282526	C	4.894479	2.543763	23.781571
H	-3.469401	4.496889	18.598416	C	4.472741	2.125778	21.030937
H	-3.252904	3.091453	17.548341	C	4.988647	1.075949	21.839769
C	1.3619	1.201539	19.433206	C	5.178861	1.323574	23.242248
H	0.654431	0.371243	19.541653	C	5.307371	-0.142535	21.212931
H	1.957303	1.259517	20.34945	H	5.697893	-0.968096	21.802606

C	5.125143	-0.263987	19.84247
C	4.646059	0.830767	19.115496
H	5.069123	2.727636	24.839415
H	3.393755	6.876532	23.080594
H	4.294936	5.093866	24.557723
H	2.883516	6.419555	20.724958
H	5.57551	0.521631	23.859995
H	5.361603	-1.186921	19.322749
H	4.523132	0.781416	18.04024
N	4.327155	2.006446	19.674391
H	2.058239	0.948636	18.626921
H	7.513101	5.364339	20.069892
H	6.060212	5.567559	21.058459
H	2.633641	4.017229	19.897986

- Thermochemistry -

Temperature 298.150 Kelvin. Pressure 1.00000 Atm.

Zero-point correction= 0.700869 (Hartree/Particle)
Thermal correction to Energy= 0.742118
Thermal correction to Enthalpy= 0.743063
Thermal correction to Gibbs Free Energy= 0.626269
Sum of electronic and zero-point Energies= -2867.402516
Sum of electronic and thermal Energies= -2867.361267
Sum of electronic and thermal Enthalpies= -2867.360323
Sum of electronic and thermal Free Energies= -2867.477117

Charge = -1 Multiplicity = 3

^{5}TS1

E(UB3LYP) = -2868.10390734 A.U.

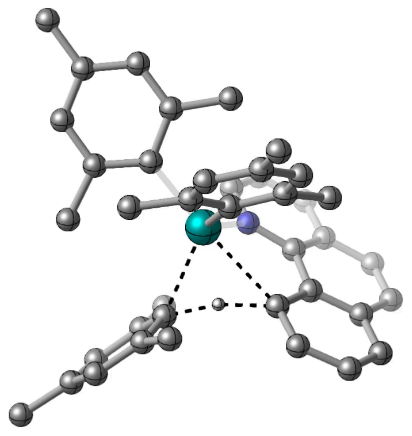

Fe	3.655464	3.607516	18.50151	C	5.710469	1.824101	15.002686
C	-0.800527	2.663972	18.836557	C	4.727657	1.488343	14.068471
C	-1.440001	3.814591	18.357646	C	3.395564	1.674958	14.446266
C	-0.703434	4.999675	18.332423	H	2.606522	1.412524	13.739629
H	-1.189759	5.92057	18.008205	C	3.050271	2.183927	15.708615
C	0.640799	5.039669	18.734907	C	5.380521	2.339192	16.266349
C	0.544195	2.678216	19.218305	C	6.486389	7.065683	18.497853

C	6.091988	7.838526	17.403095	H	2.414568	6.249737	21.257274
C	5.001081	7.384132	16.656521	H	5.79541	0.482957	23.929731
H	4.687174	7.954114	15.780413	H	5.361622	-1.209516	19.447648
C	4.312784	6.211042	17.000359	H	4.387927	0.73697	18.216512
C	5.800116	5.895095	18.861095	N	4.253939	1.956756	19.8533
H	7.351271	7.383635	19.082697	H	2.400809	4.014229	19.963309
C	4.670824	5.431775	18.135261	C	1.312093	6.39805	18.795684
H	-1.368727	1.73608	18.909107	H	2.394027	6.309641	18.905379
C	1.320517	3.860941	19.117814	H	1.110399	6.991428	17.89538
C	4.031234	2.545971	16.669725	H	0.926762	6.975258	19.647589
H	6.759561	1.682081	14.73829	C	1.160115	1.393639	19.72704
C	3.336347	5.708241	23.116024	H	1.847668	0.96188	18.990361
C	4.003163	4.731912	23.838626	H	1.748955	1.567623	20.632807
C	4.343096	3.509719	23.219609	H	0.394631	0.641593	19.950464
C	4.01689	3.322914	21.837886	C	-2.87623	3.768099	17.889152
C	3.307949	4.302634	21.092226	H	-3.340688	4.760238	17.913571
C	2.974555	5.472544	21.774697	H	-2.94743	3.401084	16.855721
C	4.995511	2.462565	23.954896	H	-3.480192	3.094375	18.508643
C	4.444651	2.097658	21.200953	C	1.574489	2.324976	16.023772
C	5.053916	1.061005	21.97022	H	0.95113	2.187586	15.131319
C	5.318579	1.279423	23.363597	H	1.25201	1.586988	16.7675
C	5.388652	-0.147113	21.328295	H	1.345744	3.300848	16.459531
H	5.846154	-0.949437	21.90217	C	6.527677	2.690502	17.196249
C	5.127925	-0.292305	19.978334	H	7.498521	2.404251	16.773623
C	4.572089	0.788951	19.284392	H	6.543764	3.76563	17.401969
H	5.218948	2.628942	25.006866	H	6.427915	2.19574	18.168856
H	3.077297	6.651477	23.594928	C	5.091178	0.973656	12.693749
H	4.261181	4.887758	24.884068	H	6.041361	0.4266	12.706617

H	4.319629	0.3028	12.297474	H	7.395706	5.30575	20.216711
H	5.203821	1.794379	11.970911	H	5.801531	5.424237	20.981847
C	3.19372	5.764252	16.077636	H	6.160027	4.056672	19.946239
H	2.800695	6.595231	15.478193	C	6.805579	9.12389	17.048509
H	3.543424	4.984431	15.390896	H	7.855301	9.102155	17.36438
H	2.362037	5.322444	16.631689	H	6.781019	9.314306	15.968868
C	6.322889	5.130962	20.063161	H	6.341447	9.992671	17.537084

- Thermochemistry -

Temperature 298.150 Kelvin. Pressure 1.00000 Atm.

Zero-point correction= 0.697895 (Hartree/Particle)
Thermal correction to Energy= 0.740562
Thermal correction to Enthalpy= 0.741506
Thermal correction to Gibbs Free Energy= 0.619804
Sum of electronic and zero-point Energies= -2867.406012
Sum of electronic and thermal Energies= -2867.363345
Sum of electronic and thermal Enthalpies= -2867.362401
Sum of electronic and thermal Free Energies= -2867.484103

Charge = -1 Multiplicity = 5

^{3}Int-A

E(UB3LYP) = -2517.92885570 A.U.

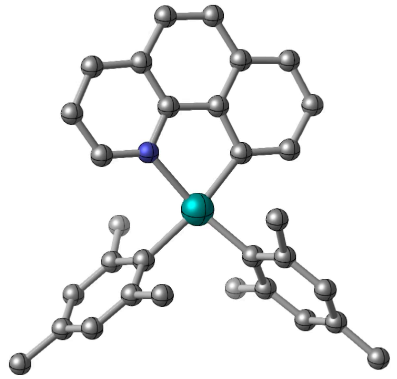

Fe	0.04107	0.000702	0.082036	H	4.371589	-2.701775	1.426766
N	-1.227026	-1.597315	-0.403361	C	2.742371	-3.977184	0.863678
C	1.307946	-1.488892	0.405594	H	3.290329	-4.901748	1.033316
C	2.639861	-1.534927	0.857489	C	1.406361	-4.024389	0.412724
H	3.164499	-0.594477	1.023902	C	0.702684	-5.253298	0.150834
C	3.33844	-2.738137	1.081812	H	1.227567	-6.193444	0.31085

C	-0.589944	-5.264537	-0.292606	H	5.581186	3.89901	-0.959487
H	-1.101524	-6.203694	-0.48926	H	4.399482	5.198042	-0.795662
C	-1.303062	-4.035703	-0.509742	H	5.177478	4.538191	0.643722
C	-2.631274	-3.955559	-0.971467	C	1.008626	2.205701	2.34537
H	-3.181641	-4.868339	-1.187931	H	-0.05152	2.392234	2.140094
C	-3.223311	-2.7127	-1.1463	H	1.062951	1.214069	2.816695
H	-4.24423	-2.620119	-1.501908	H	1.357917	2.942374	3.079366
C	-2.485719	-1.55737	-0.844341	C	2.137669	0.409066	-2.257585
H	-2.912734	-0.56386	-0.933782	H	2.373949	-0.604184	-1.909823
C	-0.624277	-2.81633	-0.245443	H	1.097803	0.384716	-2.60552
C	0.731358	-2.787474	0.205235	H	2.77177	0.635622	-3.123951
C	-1.492055	1.320697	0.066539	H	-3.173457	3.772504	-1.688085
C	-2.381265	1.312739	1.17969	C	-1.009134	2.275378	-2.22893
C	-3.486093	2.172526	1.270166	H	0.042044	2.475602	-1.998694
H	-4.127805	2.132583	2.152487	H	-1.031427	1.304002	-2.742973
C	-3.791856	3.079098	0.250625	H	-1.363598	3.033467	-2.9389
C	-2.95307	3.084797	-0.869282	C	-2.154996	0.314113	2.303031
C	-1.843319	2.231914	-0.964959	H	-2.235702	-0.716679	1.93257
C	1.490742	1.341845	0.014981	H	-1.153143	0.409391	2.738697
C	2.330365	1.405779	-1.131413	H	-2.88027	0.431093	3.117565
C	1.813909	2.240558	1.063665	C	-5.000955	3.984284	0.334482
C	3.370551	2.339508	-1.232992	H	-4.829129	4.935844	-0.183405
C	2.868383	3.158131	0.952091	H	-5.889262	3.527513	-0.126312
C	3.657917	3.238493	-0.199854	H	-5.26303	4.211035	1.375003
H	3.983978	2.358913	-2.135728	H	3.079705	3.831163	1.785077
C	4.758963	4.266789	-0.33316				

- Thermochemistry -

Temperature   298.150 Kelvin. Pressure   1.00000 Atm.

Zero-point correction=   0.518134 (Hartree/Particle)
Thermal correction to Energy=   0.549537
Thermal correction to Enthalpy=   0.550482
Thermal correction to Gibbs Free Energy=   0.453914
Sum of electronic and zero-point Energies=   -2517.410722
Sum of electronic and thermal Energies=   -2517.379318
Sum of electronic and thermal Enthalpies=   -2517.378374
Sum of electronic and thermal Free Energies=   -2517.474942

Charge = -1 Multiplicity = 3

[5]Int-A

E(UB3LYP) = -2517.93615532   A.U.

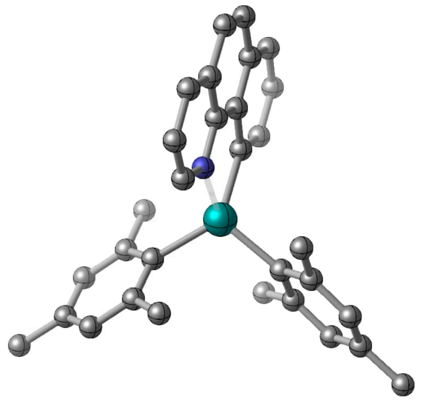

Fe	0.040309	0.129055	0.20347	C	1.343228	-4.142594	2.382102
N	-0.579683	-1.489794	-1.18245	H	1.62023	-5.112742	2.789877
C	0.609858	-1.565215	1.304662	C	0.738365	-4.072301	1.107831
C	1.206568	-1.722889	2.561804	C	0.465867	-5.250697	0.329868
H	1.39974	-0.839399	3.172977	H	0.746746	-6.218474	0.741237
C	1.570878	-2.976684	3.097598	C	-0.135066	-5.176935	-0.892136
H	2.033367	-3.033662	4.082197	H	-0.339813	-6.07647	-1.467779

C	-0.516153	-3.90821	-1.446125
C	-1.15257	-3.775532	-2.697176
H	-1.37013	-4.664475	-3.284551
C	-1.494477	-2.516294	-3.160086
H	-1.987425	-2.378242	-4.116935
C	-1.183747	-1.401945	-2.366246
H	-1.422689	-0.398238	-2.701544
C	-0.246325	-2.72458	-0.702686
C	0.388561	-2.78606	0.595512
C	-1.688608	1.290849	0.51854
C	-2.32517	1.318452	1.791069
C	-3.412761	2.159206	2.060175
H	-3.867891	2.153465	3.05197
C	-3.929222	3.021266	1.084293
C	-3.311952	3.020116	-0.16721
C	-2.222118	2.177648	-0.449298
C	1.65301	1.339773	-0.367305
C	2.32085	1.306566	-1.616801
C	2.103073	2.332478	0.543105
C	3.345368	2.212458	-1.936142
C	3.128225	3.231518	0.217589
C	3.766706	3.187683	-1.027282
H	3.829692	2.157773	-2.912566
C	4.896444	4.136686	-1.360532
H	4.982256	4.29679	-2.44175
H	4.755168	5.114419	-0.884333
H	5.866238	3.750692	-1.014853
C	1.450699	2.432342	1.910478
H	0.37394	2.620603	1.818667
H	1.547144	1.485808	2.459108
H	1.890233	3.225483	2.527745
C	1.916899	0.279789	-2.658301
H	1.841022	-0.72026	-2.217734
H	0.925653	0.501619	-3.075649
H	2.624899	0.23778	-3.495396
H	-3.685349	3.693268	-0.941088
C	-1.634069	2.250557	-1.848804
H	-1.628243	3.278216	-2.235595
H	-0.605352	1.878368	-1.863718
H	-2.219623	1.653578	-2.563372
C	-1.796217	0.444239	2.912736
H	-1.688921	-0.597223	2.592609
H	-0.789817	0.761795	3.214574
H	-2.440334	0.476335	3.800442
C	-5.122537	3.903361	1.376423
H	-5.185058	4.741498	0.672841
H	-6.066628	3.345238	1.30048
H	-5.079958	4.31682	2.391574
H	3.440304	3.982673	0.945181

- Thermochemistry -

Temperature   298.150 Kelvin. Pressure   1.00000 Atm.

Zero-point correction=   0.516112 (Hartree/Particle)
Thermal correction to Energy=   0.548839
Thermal correction to Enthalpy=   0.549783
Thermal correction to Gibbs Free Energy=   0.447588
Sum of electronic and zero-point Energies=   -2517.420043
Sum of electronic and thermal Energies=   -2517.387316
Sum of electronic and thermal Enthalpies=   -2517.386372
Sum of electronic and thermal Free Energies=   -2517.488567

Charge = -1 Multiplicity = 5

^{5}Int-bzq2

E(UB3LYP) = -3073.56026705   A.U.

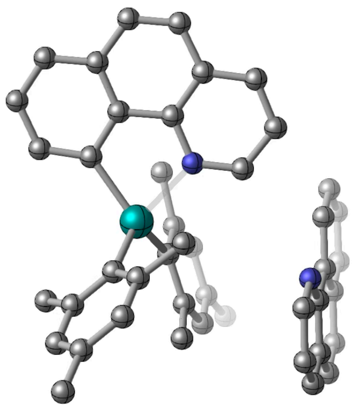

Fe	-0.401943	-0.251885	1.147946	H	-0.915115	-2.248424	6.155602
N	0.364328	-2.08332	0.221689	C	-0.164908	-3.687083	4.75499
C	-0.369607	-1.545418	2.821131	H	-0.083496	-4.489717	5.485366
C	-0.72194	-1.394432	4.168506	C	0.212328	-3.925895	3.414694
H	-1.085105	-0.42722	4.520326	C	0.719415	-5.20135	2.984494
C	-0.627561	-2.432928	5.121192	H	0.7984	-6.000548	3.719234

C	1.107481	-5.419282	1.695136	C	5.502475	1.588694	-2.643123
H	1.49739	-6.386309	1.386234	C	4.194295	1.86593	-3.104572
C	1.010347	-4.374426	0.715417	C	3.213245	0.833626	-3.069936
C	1.405923	-4.536472	-0.628599	C	3.575138	-0.438317	-2.573931
H	1.80628	-5.492794	-0.95721	C	4.858966	-0.681879	-2.11931
C	1.280645	-3.476699	-1.510793	C	3.842743	3.165903	-3.602775
H	1.575991	-3.566259	-2.551517	C	1.869455	1.124013	-3.537763
C	0.748874	-2.265411	-1.039786	C	1.564883	2.428289	-4.022374
H	0.638232	-1.414938	-1.706308	C	2.583034	3.438211	-4.041299
C	0.493179	-3.109123	1.111839	C	-0.68581	1.659793	-4.431987
C	0.09256	-2.855724	2.477656	C	-0.285583	0.404696	-3.935348
C	-2.350495	0.382306	0.715197	H	4.608853	3.936825	-3.616581
C	-2.897221	1.468696	1.445224	H	6.838141	0.140549	-1.795426
C	-4.154107	2.011389	1.14359	H	6.245819	2.381458	-2.671082
H	-4.532417	2.855618	1.722673	H	2.827503	-1.220824	-2.549684
C	-4.938851	1.495309	0.105647	H	5.116938	-1.663929	-1.732493
C	-4.422456	0.417777	-0.621415	H	2.325288	4.427025	-4.412336
C	-3.165253	-0.130494	-0.322499	H	-1.706975	1.814253	-4.765254
C	1.150651	1.100711	0.686998	H	-1.001372	-0.413935	-3.888195
C	2.462417	0.909049	1.202027	C	0.250013	2.673811	-4.473426
C	0.978918	2.248865	-0.129881	H	-0.010653	3.661713	-4.845284
C	3.500505	1.818985	0.955467	N	0.940098	0.136347	-3.505101
C	2.03011	3.146848	-0.377571	C	-2.653595	-1.283309	-1.164577
C	3.301916	2.954567	0.163856	H	-1.766652	-0.983903	-1.736395
H	4.493905	1.62873	1.365269	H	-2.333782	-2.122658	-0.536226
H	-5.014152	0.00087	-1.438299	H	-3.404661	-1.649081	-1.875958
H	1.861036	4.003455	-1.02996	C	-2.081556	2.085532	2.567156
C	5.83172	0.337978	-2.154178	H	-1.094428	2.397619	2.201963

H	-2.57388	2.957724	3.014781
H	-1.896106	1.356191	3.365997
C	-6.308677	2.061716	-0.195622
H	-7.090116	1.580981	0.410548
H	-6.35634	3.136332	0.018113
H	-6.583423	1.914125	-1.246838
C	2.788093	-0.336429	2.00676
H	2.734517	-1.232372	1.374904
H	3.794144	-0.294134	2.442529
H	2.063958	-0.497275	2.812177
C	-0.356402	2.540997	-0.788276
H	-0.788103	1.637306	-1.227344
H	-1.097916	2.903497	-0.067194
H	-0.256883	3.291425	-1.580954
C	4.425011	3.931949	-0.095403
H	4.581849	4.610014	0.755641
H	5.373459	3.410505	-0.271489
H	4.21701	4.550921	-0.975101

- Thermochemistry -

Temperature 298.150 Kelvin. Pressure 1.00000 Atm.

Zero-point correction= 0.700855 (Hartree/Particle)
Thermal correction to Energy= 0.744614
Thermal correction to Enthalpy= 0.745558
Thermal correction to Gibbs Free Energy= 0.619126
Sum of electronic and zero-point Energies= -3072.859412
Sum of electronic and thermal Energies= -3072.815653
Sum of electronic and thermal Enthalpies= -3072.814709
Sum of electronic and thermal Free Energies= -3072.941141

Charge = -1 Multiplicity = 5

^{3}TS2

E(UB3LYP) = -3073.49251671 A.U.

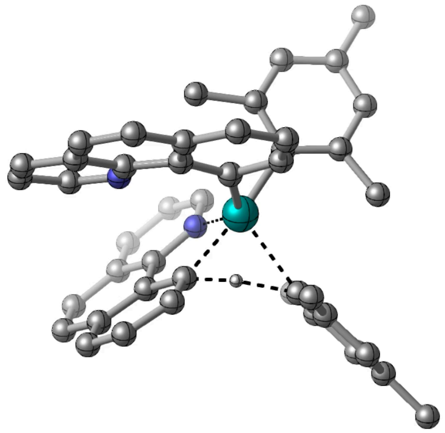

Fe	0.278406	0.335677	-0.064587	C	0.327652	-3.530662	2.923099
N	0.682512	-2.280037	-1.790321	H	0.412235	-4.324362	3.662717
C	0.206702	-1.418144	0.956544	C	0.717112	-3.785216	1.591155
C	-0.214672	-1.269616	2.291435	C	1.237024	-5.084472	1.259927
H	-0.577261	-0.297057	2.611899	H	1.326433	-5.816758	2.060341
C	-0.163784	-2.28376	3.260714	C	1.621633	-5.401548	-0.004034
H	-0.490682	-2.080036	4.279432	H	2.027317	-6.382887	-0.240543

C	1.459385	-4.447712	-1.060091	C	2.457201	0.141163	-2.100527
C	1.782619	-4.770579	-2.394494	C	2.302615	-0.091623	-0.701827
H	2.199455	-5.751036	-2.615593	C	3.342232	-0.794879	-0.0812
C	1.555965	-3.849728	-3.399173	C	3.676557	0.001673	-4.239417
H	1.791159	-4.065017	-4.437304	C	1.36533	0.728104	-2.807659
C	0.983736	-2.618852	-3.034179	C	1.450567	0.988371	-4.20384
H	0.766805	-1.871208	-3.796777	C	2.65487	0.622268	-4.896735
C	0.927705	-3.146757	-0.778731	C	-0.811743	1.810982	-4.090931
C	0.609655	-2.752589	0.593986	C	-0.819962	1.49379	-2.727434
C	-1.691999	0.650653	0.276783	H	4.569205	-0.303008	-4.781547
C	-2.238686	1.600187	1.186848	H	5.228855	-1.826529	-0.269835
C	-3.622593	1.843561	1.265556	H	5.492194	-1.327455	-2.688926
H	-3.986558	2.590676	1.97272	H	1.829466	0.909104	-0.001398
C	-4.546766	1.161428	0.47678	H	3.274361	-1.018229	0.977039
C	-4.038318	0.177183	-0.374441	H	2.721094	0.825454	-5.96279
C	-2.664675	-0.087257	-0.466523	H	-1.699705	2.237291	-4.546477
C	1.495358	2.152276	0.91772	H	-1.702983	1.652789	-2.119316
C	2.183242	2.036665	2.148421	C	0.329121	1.555271	-4.83722
C	1.353227	3.475032	0.41343	H	0.362811	1.771668	-5.902191
C	2.661505	3.164769	2.838539	N	0.229725	0.96955	-2.086951
C	1.843667	4.584295	1.113111	C	-2.251563	-1.242423	-1.352664
C	2.508642	4.454391	2.3377	H	-1.446997	-0.977128	-2.038924
H	3.16491	3.023061	3.796312	H	-1.845744	-2.060195	-0.747473
H	-4.733484	-0.412464	-0.974118	H	-3.098509	-1.629179	-1.933658
H	1.693756	5.582629	0.700245	C	-1.378925	2.403045	2.144523
C	4.462536	-1.256791	-0.791758	H	-0.545233	1.816344	2.532846
C	4.611936	-0.989373	-2.147024	H	-0.92326	3.273378	1.660169
C	3.604141	-0.278574	-2.830398	H	-1.974307	2.767877	2.991251

C	-6.025713	1.469433	0.53537
H	-6.325785	2.177063	-0.251101
H	-6.632235	0.56592	0.396943
H	-6.305486	1.919337	1.495297
C	2.427699	0.700538	2.82078
H	3.500223	0.467015	2.869236
H	2.059068	0.707114	3.855714
H	1.921005	-0.108377	2.30182
C	0.600562	3.753633	-0.868299
H	-0.397344	3.305188	-0.829677
H	0.48636	4.830294	-1.040581
H	1.10411	3.326691	-1.740964
C	3.053019	5.662303	3.065304
H	3.233392	5.445627	4.124333
H	4.007276	5.997216	2.634731
H	2.36283	6.512829	3.006655

- Thermochemistry -

Temperature 298.150 Kelvin. Pressure 1.00000 Atm.

Zero-point correction= 0.698467 (Hartree/Particle)

Thermal correction to Energy= 0.740285

Thermal correction to Enthalpy= 0.741229

Thermal correction to Gibbs Free Energy= 0.623088

Sum of electronic and zero-point Energies= -3072.794050

Sum of electronic and thermal Energies= -3072.752232

Sum of electronic and thermal Enthalpies= -3072.751288

Sum of electronic and thermal Free Energies= -3072.869429

Charge = -1 Multiplicity = 3

^{5}TS2

E(UB3LYP) = -3073.49413799 A.U.

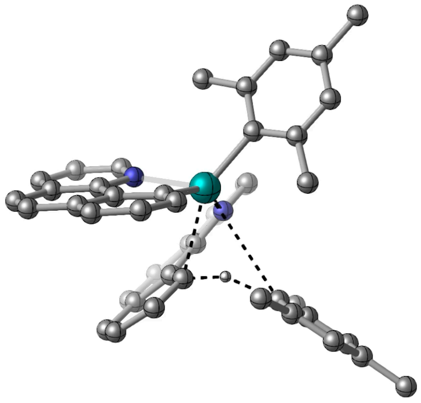

Fe	-0.290315	-0.425414	-0.173697	N	0.180536	-2.135535	-1.490738

C	0.206289	-1.879182	1.261303	C	1.956401	2.453643	0.927859
C	0.164011	-1.901495	2.657912	C	2.062371	2.494156	2.330432
H	-0.240177	-1.045509	3.196928	C	2.189874	3.668514	0.242507
C	0.653736	-2.979429	3.428159	C	2.347342	3.688428	3.019361
H	0.606329	-2.933347	4.515018	C	2.480508	4.855803	0.931315
C	1.215019	-4.086433	2.813655	C	2.560479	4.885397	2.331717
H	1.614672	-4.910284	3.401392	H	2.403641	3.687339	4.110723
C	1.263783	-4.152443	1.403235	H	-5.458952	0.024957	-1.278547
C	1.834279	-5.279935	0.717018	H	2.647156	5.781778	0.375822
H	2.246202	-6.09272	1.311984	C	4.430175	-1.388191	-0.276148
C	1.867209	-5.344813	-0.644475	C	4.600386	-1.255132	-1.649696
H	2.30388	-6.20167	-1.151713	C	3.652418	-0.535816	-2.408075
C	1.309679	-4.288578	-1.441077	C	2.512522	-0.002391	-1.737711
C	1.281885	-4.321873	-2.849953	C	2.370308	-0.054617	-0.324486
H	1.715405	-5.169783	-3.374674	C	3.354679	-0.759493	0.374221
C	0.693959	-3.279496	-3.54597	C	3.806662	-0.314941	-3.821221
H	0.646541	-3.275734	-4.629947	C	1.471019	0.597712	-2.529716
C	0.150604	-2.2101	-2.820845	C	1.673116	0.85262	-3.91519
H	-0.318615	-1.377511	-3.334404	C	2.881667	0.386121	-4.536889
C	0.744235	-3.159325	-0.78577	C	-0.533814	1.829345	-3.973323
C	0.735459	-3.058771	0.655476	C	-0.683369	1.458955	-2.628446
C	-2.273946	0.283613	0.014703	H	4.695111	-0.70623	-4.31252
C	-2.597228	1.211786	1.039349	H	5.155952	-1.958768	0.300622
C	-3.901267	1.695376	1.216002	H	5.462133	-1.692619	-2.148863
H	-4.106933	2.411863	2.012564	H	1.874008	0.940653	0.233629
C	-4.952441	1.279354	0.391512	H	3.29157	-0.814408	1.459847
C	-4.65667	0.36258	-0.620855	H	3.021692	0.570609	-5.599129
C	-3.353347	-0.125315	-0.808829	H	-1.348323	2.326392	-4.489805

H	-1.615453	1.621212	-2.096236
C	0.642985	1.500106	-4.625487
H	0.775665	1.718623	-5.682319
N	0.273407	0.84891	-1.925745
C	-3.108851	-1.094123	-1.95073
H	-2.564128	-0.607155	-2.771771
H	-2.488842	-1.936058	-1.62716
H	-4.042747	-1.489628	-2.367663
C	-1.510043	1.688388	1.98202
H	-1.101829	0.850764	2.560622
H	-0.657024	2.11695	1.442241
H	-1.872761	2.43944	2.694308
C	-6.362879	1.77848	0.612031
H	-6.37151	2.813236	0.974735
H	-6.953441	1.737823	-0.310606
H	-6.892933	1.173016	1.360961
C	1.882804	1.219841	3.136681
H	1.230405	1.367489	4.008696
H	1.450069	0.429985	2.520577
H	2.843736	0.84679	3.523072
C	2.129016	3.701797	-1.274992
H	1.12902	3.441113	-1.641121
H	2.389696	4.688668	-1.678717
H	2.813769	2.965742	-1.71382
C	2.894197	6.167734	3.062839
H	3.96974	6.392842	3.022346
H	2.375267	7.030003	2.62531
H	2.616239	6.110467	4.121778

- Thermochemistry -

Temperature 298.150 Kelvin. Pressure 1.00000 Atm.

Zero-point correction= 0.695092 (Hartree/Particle)
Thermal correction to Energy= 0.738707
Thermal correction to Enthalpy= 0.739651
Thermal correction to Gibbs Free Energy= 0.613776
Sum of electronic and zero-point Energies= -3072.799046
Sum of electronic and thermal Energies= -3072.755431
Sum of electronic and thermal Enthalpies= -3072.754487
Sum of electronic and thermal Free Energies= -3072.880362

Charge = -1 Multiplicity = 5

^{3}Int-B

E(UB3LYP) = -2723.34567729 A.U.

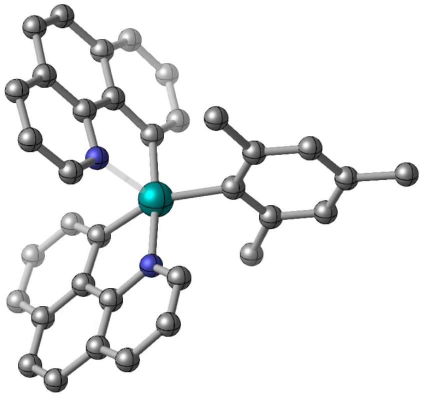

Fe	4.778547	3.24726	19.540728	C	2.712873	-0.936479	20.71707
N	2.822581	3.392342	18.619585	H	2.189547	-1.853498	20.979465
N	5.248442	5.11826	19.07218	C	2.048642	0.06136	19.974357
C	4.124749	1.492439	20.030535	C	0.684124	-0.072487	19.53473
C	4.721854	0.448827	20.756738	H	0.149183	-0.9869	19.785046
H	5.761414	0.548156	21.068135	C	0.057537	0.907928	18.819429
C	4.035457	-0.731681	21.09892	H	-0.973913	0.785963	18.496567
H	4.550142	-1.504093	21.669313	C	0.744808	2.122627	18.476614

C	0.16864	3.175022	17.738412	C	5.794901	5.559022	17.921377
H	-0.860134	3.092855	17.395581	H	6.138694	4.782772	17.24628
C	0.924866	4.305995	17.457322	C	4.817619	6.070662	19.973263
H	0.510017	5.133984	16.890955	C	4.230611	5.55585	21.164014
C	2.247623	4.370564	17.918651	C	6.275069	2.460181	18.442179
H	2.870945	5.239066	17.721462	C	6.156432	1.946028	17.127338
C	2.092832	2.272105	18.902469	C	7.580099	2.448117	18.997522
C	2.762402	1.25276	19.652055	C	7.269326	1.46147	16.422828
C	4.114999	4.132667	21.248457	C	8.682447	1.954704	18.286224
C	3.552216	3.667878	22.443395	C	8.5503	1.457044	16.984944
H	3.43173	2.594258	22.589306	H	7.135238	1.071843	15.412125
C	3.116234	4.536045	23.469653	H	9.668751	1.958601	18.753754
H	2.678162	4.119501	24.376124	C	9.749694	0.961513	16.207456
C	3.223992	5.916717	23.341824	H	9.466505	0.197546	15.473373
H	2.876779	6.579097	24.132583	H	10.24126	1.772823	15.650757
C	3.789368	6.462885	22.169491	H	10.507786	0.526157	16.869834
C	3.936445	7.874137	21.933996	C	7.791355	2.996367	20.396338
H	3.595073	8.568209	22.700084	H	7.549538	4.066531	20.436784
C	4.484088	8.352917	20.775278	H	7.124768	2.506136	21.118041
H	4.58077	9.424237	20.613183	H	8.82345	2.866658	20.744835
C	4.948872	7.462516	19.745353	C	4.795346	1.915658	16.462334
C	5.521214	7.882066	18.525418	H	4.075173	1.36388	17.077198
H	5.633182	8.941981	18.3116	H	4.383731	2.927449	16.355285
C	5.935978	6.915695	17.612904	H	4.829231	1.455756	15.46689
H	6.383075	7.198521	16.664567				

- Thermochemistry -

Temperature   298.150 Kelvin. Pressure   1.00000 Atm.

Zero-point correction=   0.515666 (Hartree/Particle)
Thermal correction to Energy=   0.547353
Thermal correction to Enthalpy=   0.548298
Thermal correction to Gibbs Free Energy=   0.451092
Sum of electronic and zero-point Energies=   -2722.830011
Sum of electronic and thermal Energies=   -2722.798324
Sum of electronic and thermal Enthalpies=   -2722.797380
Sum of electronic and thermal Free Energies=   -2722.894586

Charge = -1 Multiplicity = 3

^{5}Int-B

E(UB3LYP) = -2723.32589341   A.U.

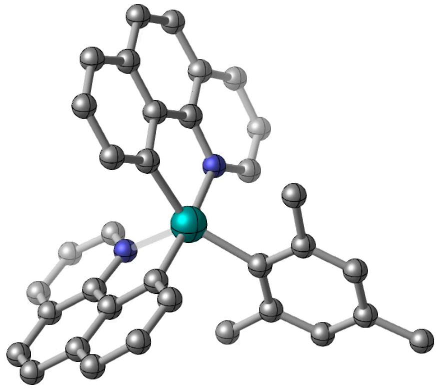

Fe	4.695666	3.29183	19.504337	H	4.528367	-1.452398	21.705168
N	2.744436	3.375545	18.560273	C	2.721657	-0.950591	20.666497
N	5.162595	5.1743	18.983748	H	2.220431	-1.884838	20.908662
C	4.060977	1.506361	20.039909	C	2.050476	0.020135	19.891775
C	4.673093	0.505482	20.794369	C	0.708377	-0.161651	19.403162
H	5.690881	0.650222	21.151487	H	0.199294	-1.095319	19.632589
C	4.013265	-0.701163	21.109496	C	0.0699	0.80058	18.674392

H	-0.945238	0.644779	18.3182
C	0.72447	2.041003	18.36265
C	0.135067	3.087122	17.622981
H	-0.880709	2.974673	17.251944
C	0.853211	4.247378	17.376319
H	0.421673	5.066984	16.81153
C	2.165569	4.350869	17.865507
H	2.776747	5.232402	17.695158
C	2.051595	2.235117	18.824815
C	2.734479	1.233242	19.596685
C	4.190975	4.159046	21.278236
C	3.700035	3.659543	22.474963
H	3.57981	2.584654	22.603711
C	3.340424	4.521905	23.551854
H	2.960826	4.092887	24.478355
C	3.462668	5.892287	23.435671
H	3.182452	6.544309	24.262136
C	3.959891	6.47497	22.236926
C	4.124279	7.870606	22.036249
H	3.855123	8.558262	22.836415
C	4.626418	8.35884	20.840155
H	4.749537	9.432691	20.705601
C	5.000935	7.502293	19.7614
C	5.529326	7.948902	18.50863
H	5.669679	9.010059	18.321936
C	5.875288	6.977682	17.540624
H	6.2904	7.28141	16.582087
C	5.694568	5.634252	17.794936
H	5.960001	4.862071	17.080909
C	4.835093	6.105505	19.950304
C	4.323347	5.583737	21.173474
C	6.200206	2.480374	18.442162
C	6.100916	1.932569	17.145304
C	7.482032	2.498519	19.039428
C	7.234773	1.433535	16.485106
C	8.600954	1.987417	18.369296
C	8.499288	1.450329	17.080994
H	7.127981	1.016684	15.482848
H	9.575392	2.012138	18.858901
C	9.719459	0.936224	16.349639
H	9.451378	0.182559	15.600074
H	10.245731	1.743547	15.820608
H	10.441734	0.483793	17.039626
C	7.650325	3.102208	20.420394
H	7.37067	4.163607	20.415129
H	6.994169	2.617749	21.155444
H	8.68091	3.021313	20.78485
C	4.757428	1.877939	16.446905
H	4.034349	1.297498	17.0321
H	4.327534	2.880937	16.33524
H	4.830101	1.427552	15.450233

- Thermochemistry -

Temperature   298.150 Kelvin. Pressure   1.00000 Atm.

Zero-point correction=   0.514231 (Hartree/Particle)
Thermal correction to Energy=   0.546194
Thermal correction to Enthalpy=   0.547138
Thermal correction to Gibbs Free Energy=   0.448868
Sum of electronic and zero-point Energies=   -2722.811663
Sum of electronic and thermal Energies=   -2722.779700
Sum of electronic and thermal Enthalpies=   -2722.778755
Sum of electronic and thermal Free Energies=   -2722.877026

Charge = -1 Multiplicity = 5

[5]Int-bzq3

E(UB3LYP) = -3278.95204134 A.U.

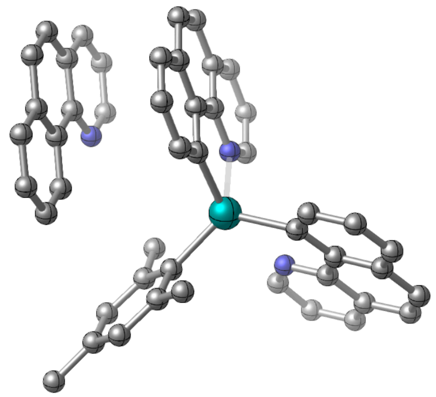

Fe	4.348142	3.787241	19.815166	H	5.270097	1.155762	21.784698
N	2.824896	3.324503	18.215919	C	4.21581	-0.568287	21.071796
N	5.108624	1.349386	15.671111	H	4.668362	-1.2244	21.814708
N	3.733464	6.559823	19.194307	C	3.310351	-1.08895	20.161879
C	4.035555	1.709495	20.109555	H	3.045918	-2.144432	20.173744
C	4.556587	0.80121	21.040092	C	2.7222	-0.238034	19.201293

C	1.764179	-0.726696	18.248673	C	3.302508	0.546876	14.289323
H	1.505415	-1.783589	18.271791	H	2.562014	0.775301	13.529338
C	1.191648	0.096955	17.326617	C	4.182147	1.547741	14.745396
H	0.474193	-0.285349	16.604673	H	4.1185	2.555897	14.33855
C	1.532144	1.489638	17.279229	C	5.215651	0.117613	16.230055
C	0.96993	2.380395	16.34412	C	6.221534	-0.096454	17.255706
H	0.25418	2.007828	15.614954	C	3.407361	4.790018	21.405419
C	1.331984	3.715793	16.366369	C	3.192072	4.046217	22.575798
H	0.912532	4.430186	15.665262	H	3.458967	2.989727	22.569684
C	2.260126	4.141518	17.329236	C	2.647289	4.581496	23.760699
H	2.556368	5.182437	17.396414	H	2.508348	3.947848	24.635828
C	2.48018	2.002875	18.210637	C	2.284591	5.914729	23.810777
C	3.098669	1.139897	19.19458	H	1.860987	6.348466	24.714717
C	7.08968	0.933653	17.677405	C	2.467849	6.735352	22.674926
C	8.020473	0.712276	18.677411	C	2.108963	8.125567	22.729163
H	8.652809	1.532749	19.004115	H	1.684554	8.512277	23.653938
C	8.121012	-0.556826	19.282802	C	2.299451	8.952191	21.665178
H	8.850075	-0.725527	20.071073	H	2.033862	10.005601	21.718547
C	7.287856	-1.58523	18.879565	C	2.862841	8.446575	20.448118
H	7.357555	-2.565236	19.34589	C	3.090076	9.281759	19.334351
C	6.3233	-1.381031	17.865104	H	2.82976	10.336076	19.397937
C	5.442745	-2.436538	17.448647	C	3.64143	8.752558	18.184251
H	5.534992	-3.405752	17.932783	H	3.836139	9.36492	17.309184
C	4.508671	-2.2382	16.477876	C	3.942457	7.378905	18.170935
H	3.84364	-3.042696	16.173611	H	4.370866	6.928575	17.278246
C	4.369098	-0.959664	15.843723	C	3.212092	7.062803	20.345216
C	3.402667	-0.712281	14.845222	C	3.024368	6.167546	21.481266
H	2.739274	-1.516018	14.535364	C	6.379396	4.159989	19.3634

C	7.384299	4.011617	20.355835	H	6.027951	4.001882	22.057969
C	6.853398	4.479511	18.063558	H	6.954926	2.517744	21.864761
C	8.748121	4.177811	20.068733	C	5.877365	4.614402	16.910384
C	8.219623	4.632846	17.783241	H	6.192876	5.377157	16.185655
C	9.191029	4.485735	18.778396	H	5.766517	3.66596	16.367467
H	9.485143	4.060829	20.865506	H	4.888688	4.874455	17.288482
H	8.537339	4.873468	16.766813	C	10.658938	4.693283	18.478983
H	7.003162	1.914398	17.229496	H	11.294498	4.121988	19.165907
C	7.009983	3.611177	21.772397	H	10.907698	4.387419	17.45564
H	7.747699	3.958862	22.507244	H	10.949148	5.749442	18.576265

- Thermochemistry -

Temperature 298.150 Kelvin. Pressure 1.00000 Atm.

Zero-point correction= 0.699640 (Hartree/Particle)
Thermal correction to Energy= 0.743510
Thermal correction to Enthalpy= 0.744454
Thermal correction to Gibbs Free Energy= 0.617060
Sum of electronic and zero-point Energies= -3278.252401
Sum of electronic and thermal Energies= -3278.208531
Sum of electronic and thermal Enthalpies= -3278.207587
Sum of electronic and thermal Free Energies= -3278.334982

Charge = -1 Multiplicity = 5

^{3}TS3

E(UB3LYP) = -3278.88595734 A.U.

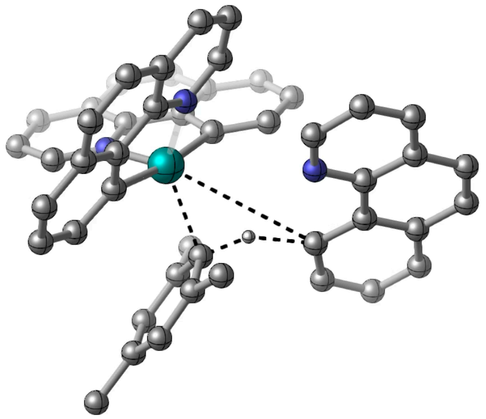

Fe	3.803207	3.546073	19.37993	H	4.360257	0.981114	17.370639
N	2.357571	3.405616	20.853391	C	2.926203	-0.514857	17.942743
N	6.05087	2.640466	16.222953	H	3.278967	-1.22024	17.190957
N	3.180874	4.250673	17.475268	C	1.882507	-0.886546	18.785363
C	3.158624	1.707745	18.997338	H	1.41475	-1.865389	18.700881
C	3.542451	0.749272	18.045002	C	1.433227	0.024656	19.76302

C	0.371457	-0.262217	20.692066	C	5.798964	3.116216	13.869647
H	-0.124464	-1.229204	20.629311	H	5.464084	3.813635	13.107615
C	-0.016354	0.639987	21.642672	C	5.677446	3.439032	15.234381
H	-0.817279	0.402193	22.33877	H	5.248176	4.394974	15.529188
C	0.628012	1.921224	21.752068	C	6.607179	1.435943	15.929944
C	0.304937	2.897006	22.713278	C	7.041921	0.574063	17.026959
H	-0.485621	2.705761	23.434988	C	4.202672	5.504352	19.636699
C	1.0081	4.094738	22.726727	C	4.700785	6.238506	20.72041
H	0.786112	4.868725	23.454402	H	4.970644	5.713653	21.635245
C	2.02036	4.309575	21.781624	C	4.910011	7.633168	20.67218
H	2.581285	5.236546	21.758883	H	5.305819	8.147145	21.547169
C	1.66838	2.219688	20.833	C	4.629385	8.362038	19.524574
C	2.080968	1.290536	19.835437	H	4.797312	9.436317	19.487129
C	6.866432	0.957564	18.39077	C	4.114874	7.695182	18.391525
C	7.379903	0.073916	19.338978	C	3.791968	8.374663	17.165488
H	7.289255	0.335731	20.397608	H	3.960329	9.448385	17.109271
C	8.009714	-1.147912	19.018353	C	3.281718	7.707987	16.088803
H	8.386788	-1.800971	19.806024	H	3.038919	8.23735	15.170722
C	8.140127	-1.523746	17.69296	C	3.045123	6.291887	16.144927
H	8.610546	-2.467004	17.420451	C	2.493193	5.546046	15.082839
C	7.664224	-0.671201	16.6696	H	2.230593	6.046489	14.153895
C	7.808956	-1.051941	15.291267	C	2.291439	4.186417	15.239737
H	8.276708	-2.009391	15.066429	H	1.86927	3.581469	14.444279
C	7.380377	-0.246953	14.280349	C	2.652486	3.578306	16.453097
H	7.496196	-0.543822	13.240031	H	2.526393	2.513606	16.607298
C	6.778144	1.021312	14.570301	C	3.372301	5.598818	17.341037
C	6.352171	1.893012	13.54479	C	3.909494	6.287746	18.481962
H	6.471312	1.589593	12.506577	C	5.958365	2.950928	20.17703

C	5.723157	2.401134	21.464177
C	6.921394	4.004869	20.098807
C	6.300705	2.981491	22.609693
C	7.513305	4.531617	21.240228
C	7.186165	4.053683	22.521628
H	6.07555	2.559427	23.59003
H	8.237168	5.342011	21.146992
H	6.071726	2.159619	19.204718
C	4.88934	1.151654	21.653274
H	4.780979	0.615132	20.710423
H	5.353732	0.485803	22.392165
H	3.879327	1.380611	22.013593
C	7.304599	4.529239	18.739465
H	8.202335	5.158223	18.778913
H	7.462885	3.694727	18.048514
H	6.487167	5.127509	18.318579
C	7.791578	4.68494	23.753439
H	7.586315	4.094082	24.652881
H	8.879902	4.791063	23.659197
H	7.390137	5.693947	23.922473

- Thermochemistry -

Temperature 298.150 Kelvin. Pressure 1.00000 Atm.

Zero-point correction= 0.695546 (Hartree/Particle)
Thermal correction to Energy= 0.737966
Thermal correction to Enthalpy= 0.738911
Thermal correction to Gibbs Free Energy= 0.618983
Sum of electronic and zero-point Energies= -3278.190412
Sum of electronic and thermal Energies= -3278.147991
Sum of electronic and thermal Enthalpies= -3278.147047
Sum of electronic and thermal Free Energies= -3278.266974

Charge = -1 Multiplicity = 3

^{5}TS3

E(UB3LYP) = -3278.88982255 A.U.

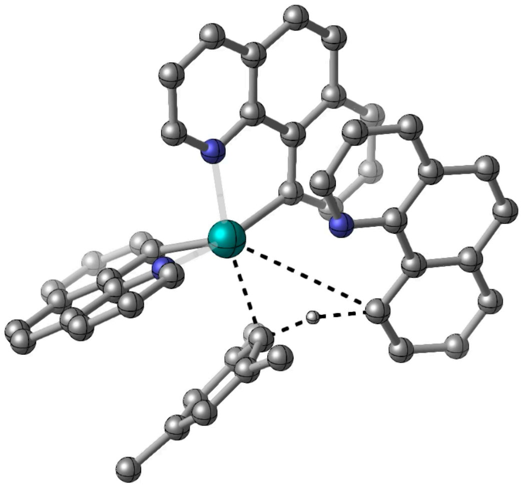

Fe	4.161068	3.284874	19.4418	C	3.825606	1.221641	19.630869
N	2.082736	3.095202	18.591407	C	4.625073	0.19332	20.138381
N	4.722072	1.700186	16.603166	H	5.622556	0.425236	20.498059
N	4.090247	5.408305	18.531762	C	4.224296	-1.158766	20.15716

H	4.900242	-1.916286	20.551129	H	4.316292	-2.700623	15.342878
C	2.988739	-1.536539	19.657931	C	4.412637	-0.601349	15.904148
H	2.677391	-2.579339	19.659804	C	3.215153	-0.235035	15.253915
C	2.122469	-0.554955	19.130098	H	2.636472	-0.997306	14.736196
C	0.836618	-0.897672	18.587421	C	2.795539	1.079503	15.283426
H	0.535219	-1.943571	18.595761	H	1.876155	1.399456	14.802881
C	0.003801	0.048928	18.067955	C	3.600225	2.006738	15.968984
H	-0.965431	-0.224198	17.657369	H	3.293854	3.050038	16.013068
C	0.391222	1.430378	18.04997	C	5.152118	0.409403	16.597322
C	-0.426049	2.448512	17.519418	C	6.377307	0.062042	17.308567
H	-1.396284	2.193286	17.099665	C	3.845828	4.4666	21.127357
C	0.017556	3.75987	17.542237	C	3.688898	4.110712	22.47239
H	-0.586964	4.570259	17.147395	H	3.555839	3.059587	22.733115
C	1.278431	4.033941	18.093677	C	3.721924	5.045266	23.52861
H	1.657492	5.049316	18.133745	H	3.600668	4.706069	24.556679
C	1.659409	1.797763	18.583524	C	3.913354	6.394424	23.272699
C	2.554431	0.806604	19.13272	H	3.947602	7.119704	24.083157
C	7.081802	1.02706	18.08632	C	4.059942	6.835596	21.93892
C	8.247071	0.580866	18.707938	C	4.262712	8.222956	21.620219
H	8.808887	1.287144	19.329009	H	4.30706	8.939711	22.43816
C	8.737114	-0.73889	18.603262	C	4.395294	8.649592	20.33145
H	9.661453	-1.031217	19.102566	H	4.543094	9.70278	20.104708
C	8.023874	-1.674448	17.874041	C	4.331898	7.716407	19.243308
H	8.366025	-2.70537	17.798828	C	4.430039	8.106259	17.892818
C	6.825615	-1.298906	17.221297	H	4.570606	9.156777	17.648764
C	6.062053	-2.278416	16.498992	C	4.336904	7.152842	16.894217
H	6.430765	-3.302711	16.46702	H	4.397115	7.420114	15.844337
C	4.896296	-1.95085	15.877125	C	4.165528	5.811062	17.268133

H	4.102598	5.032558	16.515053
C	4.152419	6.333013	19.530819
C	4.013038	5.865475	20.89265
C	6.631699	3.563541	19.232834
C	6.977103	3.77618	20.591838
C	7.020745	4.575766	18.318192
C	7.508816	4.99935	21.019904
C	7.544473	5.794114	18.758847
C	7.758198	6.041214	20.120485
H	7.712455	5.156249	22.080007
H	7.782776	6.573507	18.033585
H	6.623058	2.370683	18.665512
C	6.77069	2.669625	21.603007
H	7.091963	2.967076	22.608172
H	5.715111	2.387583	21.657677
H	7.330304	1.772242	21.312193
C	6.907701	4.30487	16.834507
H	6.809683	5.229935	16.253532
H	7.80765	3.782505	16.477218
H	6.06683	3.640409	16.622859
C	8.215075	7.398066	20.603557
H	8.771295	7.327285	21.545755
H	8.855836	7.897067	19.866558
H	7.353923	8.057077	20.783226

- Thermochemistry -

Temperature 298.150 Kelvin. Pressure 1.00000 Atm.

Zero-point correction= 0.693730 (Hartree/Particle)
Thermal correction to Energy= 0.736770
Thermal correction to Enthalpy= 0.737714
Thermal correction to Gibbs Free Energy= 0.616015
Sum of electronic and zero-point Energies= -3278.196093
Sum of electronic and thermal Energies= -3278.153052
Sum of electronic and thermal Enthalpies= -3278.152108
Sum of electronic and thermal Free Energies= -3278.273808

Charge = -1 Multiplicity = 5

^{1}1-bzq

E(UB3LYP) = -2928.76251476 A.U.

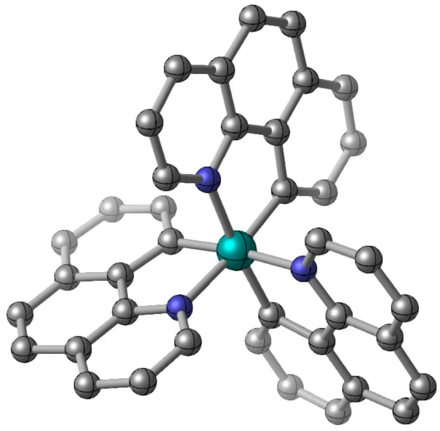

Fe	4.265031	3.063565	18.645505	C	4.477818	0.160764	19.677352
N	2.269369	3.38192	18.668401	H	5.564949	0.184037	19.614651
N	4.222669	2.393717	16.739542	C	3.861159	-1.022388	20.135476
N	4.643025	4.960249	18.059438	H	4.48247	-1.875716	20.407557
C	3.75182	1.307289	19.309157	C	2.478435	-1.130418	20.250274

H	2.013289	-2.048274	20.604286
C	1.676862	-0.023555	19.898097
C	0.240097	-0.018927	19.982681
H	-0.261433	-0.920673	20.330262
C	-0.498393	1.081861	19.645192
H	-1.583409	1.064006	19.720363
C	0.136217	2.287941	19.188107
C	-0.550067	3.466648	18.830173
H	-1.635229	3.503596	18.88914
C	0.182379	4.567303	18.410306
H	-0.3077	5.494482	18.129448
C	1.582395	4.484242	18.344678
H	2.17406	5.332095	18.017685
C	1.551492	2.291398	19.091398
C	2.334011	1.153613	19.440778
C	6.151866	2.606799	18.51271
C	7.231016	2.680345	19.410951
H	7.052976	3.054434	20.418181
C	8.540553	2.282748	19.068387
H	9.333363	2.363908	19.812106
C	8.849249	1.788625	17.804449
H	9.863289	1.485831	17.551076
C	7.820457	1.68252	16.844166
C	8.020749	1.178888	15.510858
H	9.022793	0.86849	15.219257
C	6.991064	1.077952	14.616024
H	7.165003	0.690723	13.614491
C	5.656686	1.47493	14.974193
C	4.540368	1.39385	14.116397
H	4.657786	1.011253	13.105351
C	3.30179	1.803924	14.587624
H	2.417196	1.753888	13.960169
C	3.186363	2.294103	15.89824
H	2.228158	2.621283	16.286466
C	5.449761	1.982912	16.28239
C	6.511631	2.092496	17.225503
C	4.444392	3.830567	20.42535
C	4.370166	3.29379	21.722759
H	4.18187	2.227468	21.840606
C	4.536352	4.075548	22.885196
H	4.467929	3.59777	23.862585
C	4.785957	5.443144	22.818753
H	4.911015	6.036634	23.72225
C	4.877336	6.058707	21.55211
C	5.14077	7.460885	21.362355
H	5.267766	8.0881	22.243235
C	5.239326	8.013658	20.115046
H	5.442765	9.075512	19.995369
C	5.08287	7.209412	18.933917
C	5.181246	7.697752	17.614703
H	5.386374	8.750951	17.43836
C	5.017552	6.813339	16.558613
H	5.089763	7.148663	15.528528
C	4.752099	5.460144	16.822513
H	4.618823	4.752063	16.012106
C	4.811631	5.828108	19.109157

C	4.705767	5.238304	20.40145

- Thermochemistry -

Temperature 298.150 Kelvin. Pressure 1.00000 Atm.

Zero-point correction= 0.517354 (Hartree/Particle)
Thermal correction to Energy= 0.547791
Thermal correction to Enthalpy= 0.548735
Thermal correction to Gibbs Free Energy= 0.457392
Sum of electronic and zero-point Energies= -2928.245160
Sum of electronic and thermal Energies= -2928.214724
Sum of electronic and thermal Enthalpies= -2928.213780
Sum of electronic and thermal Free Energies= -2928.305123

Charge = -1 Multiplicity = 1

^{3}1-bzq

E(UB3LYP) = -2928.74387734 A.U.

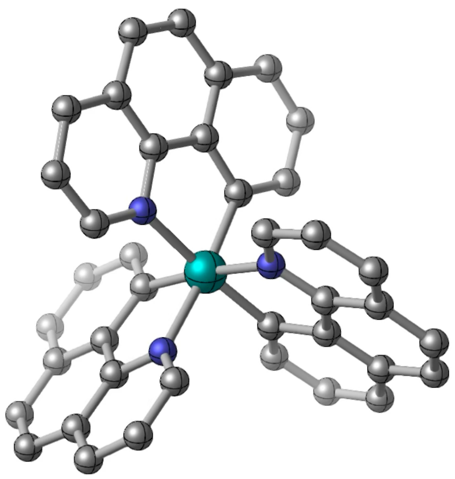

Fe	4.285595	3.042845	18.676496	C	4.487363	0.154616	19.692886
N	2.264259	3.392949	18.696995	H	5.572445	0.181898	19.622724
N	4.215377	2.363502	16.743277	C	3.864467	-1.02581	20.166769
N	4.674087	4.958382	18.055459	H	4.482896	-1.879298	20.441701
C	3.74611	1.27956	19.328618	C	2.486653	-1.121817	20.286947

H	2.020523	-2.036639	20.647622	C	4.541723	1.454461	14.098386
C	1.675379	-0.016838	19.932083	H	4.661631	1.101796	13.077253
C	0.245353	-0.017038	20.00659	C	3.300997	1.875652	14.571187
H	-0.258739	-0.916265	20.355581	H	2.425569	1.860588	13.92862
C	-0.49317	1.083854	19.639965	C	3.171085	2.318607	15.890037
H	-1.579243	1.059636	19.696711	H	2.216556	2.640965	16.289044
C	0.136595	2.28342	19.176141	C	5.441532	1.969528	16.289602
C	-0.548805	3.456506	18.773631	C	6.50776	2.063823	17.233564
H	-1.634931	3.487274	18.803874	C	4.454189	3.838201	20.455588
C	0.187981	4.556461	18.342695	C	4.375707	3.284173	21.733866
H	-0.306998	5.470049	18.027022	H	4.178889	2.220294	21.84603
C	1.584335	4.493806	18.319225	C	4.558505	4.070656	22.897818
H	2.184622	5.337454	17.999563	H	4.490044	3.596124	23.875921
C	1.555015	2.300626	19.106662	C	4.819863	5.429896	22.822071
C	2.333394	1.161148	19.470754	H	4.952475	6.023497	23.724524
C	6.18153	2.591196	18.516519	C	4.912271	6.055071	21.5546
C	7.236956	2.670482	19.425796	C	5.165787	7.451384	21.367099
H	7.054096	3.049557	20.428713	H	5.295848	8.08083	22.245406
C	8.547703	2.257294	19.081629	C	5.237143	8.005815	20.10982
H	9.341407	2.335652	19.823564	H	5.422381	9.071089	19.990001
C	8.844987	1.758407	17.823035	C	5.071813	7.208415	18.932266
H	9.857512	1.449713	17.570273	C	5.124936	7.70061	17.603989
C	7.819027	1.6518	16.852316	H	5.301403	8.757956	17.424058
C	8.022349	1.162467	15.522764	C	4.948073	6.812411	16.545852
H	9.022683	0.850611	15.228451	H	4.982753	7.157734	15.516772
C	6.987584	1.089463	14.61838	C	4.728952	5.456389	16.803035
H	7.165752	0.721913	13.610062	H	4.599317	4.740209	16.000245
C	5.659254	1.491065	14.970091	C	4.829411	5.819488	19.103767

C	4.737185	5.233634	20.401893

- Thermochemistry -

Temperature 298.150 Kelvin. Pressure 1.00000 Atm.

Zero-point correction= 0.511812 (Hartree/Particle)
Thermal correction to Energy= 0.543164
Thermal correction to Enthalpy= 0.544108
Thermal correction to Gibbs Free Energy= 0.449549
Sum of electronic and zero-point Energies= -2928.232065
Sum of electronic and thermal Energies= -2928.200713
Sum of electronic and thermal Enthalpies= -2928.199769
Sum of electronic and thermal Free Energies= -2928.294328

Charge = -1 Multiplicity = 3

^{5}1-bzq

E(UB3LYP) = -2928.71599685 A.U.

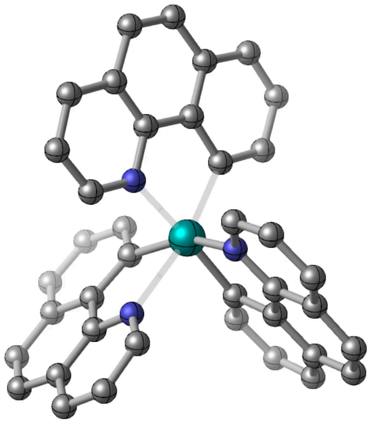

Fe	4.263114	3.107558	18.974335	H	5.624395	0.236788	19.875185
N	2.244376	3.334752	18.75766	C	3.941923	-1.106481	20.142476
N	4.093751	2.436407	16.575672	H	4.574815	-1.937744	20.451153
N	4.669113	5.085774	18.299927	C	2.576906	-1.288989	20.050083
C	3.771644	1.234327	19.439677	H	2.131068	-2.255196	20.283042
C	4.541737	0.145976	19.825149	C	1.729119	-0.215539	19.65447

C	0.318341	-0.310332	19.548353	C	3.17543	1.776964	14.457703
H	-0.168792	-1.259101	19.767547	H	2.307167	1.728404	13.808209
C	-0.441001	0.795369	19.186758	C	3.068375	2.349672	15.738457
H	-1.524371	0.705647	19.116807	H	2.128533	2.758297	16.102424
C	0.144304	2.064276	18.911724	C	5.302736	1.944136	16.208122
C	-0.584437	3.244092	18.550512	C	6.370868	2.022862	17.178112
H	-1.667687	3.2132	18.469373	C	4.532793	4.103326	20.84939
C	0.136804	4.434009	18.316103	C	4.494815	3.674889	22.176751
H	-0.386059	5.347614	18.040878	H	4.290486	2.625641	22.394529
C	1.513751	4.456141	18.422404	C	4.715532	4.550396	23.264639
H	2.092446	5.35345	18.233573	H	4.674747	4.169322	24.284046
C	1.559156	2.170266	19.005427	C	4.984726	5.893875	23.050395
C	2.354817	1.0439	19.368521	H	5.153767	6.569003	23.886717
C	6.104667	2.530712	18.482919	C	5.041678	6.395745	21.730803
C	7.18893	2.57486	19.362077	C	5.316689	7.776494	21.438257
H	7.031371	2.951991	20.370644	H	5.483672	8.459519	22.268825
C	8.477892	2.136481	18.993499	C	5.369457	8.237154	20.154655
H	9.290766	2.190339	19.715699	H	5.577173	9.284074	19.947866
C	8.718737	1.627619	17.727209	C	5.153816	7.351957	19.044198
H	9.708892	1.277133	17.44426	C	5.198238	7.769981	17.698586
C	7.663117	1.555761	16.791554	H	5.40341	8.812119	17.466196
C	7.854742	1.00846	15.474479	C	4.981267	6.846211	16.69149
H	8.848659	0.657656	15.203972	H	5.008559	7.131882	15.645146
C	6.829599	0.908429	14.580039	C	4.721716	5.51116	17.038626
H	6.991139	0.479821	13.593757	H	4.547003	4.756355	16.281741
C	5.51494	1.371375	14.922519	C	4.879379	5.982449	19.308275
C	4.408721	1.296793	14.049582	C	4.815022	5.483338	20.659062
H	4.536742	0.860381	13.061653				

- Thermochemistry -

Temperature 298.150 Kelvin. Pressure 1.00000 Atm.

Zero-point correction= 0.512989 (Hartree/Particle)
Thermal correction to Energy= 0.544917
Thermal correction to Enthalpy= 0.545861
Thermal correction to Gibbs Free Energy= 0.448156
Sum of electronic and zero-point Energies= -2928.203008
Sum of electronic and thermal Energies= -2928.171080
Sum of electronic and thermal Enthalpies= -2928.170136
Sum of electronic and thermal Free Energies= -2928.267841

Charge = -1 Multiplicity = 5

[2]Int-A

E(UB3LYP) = -2517.81640164 A.U.

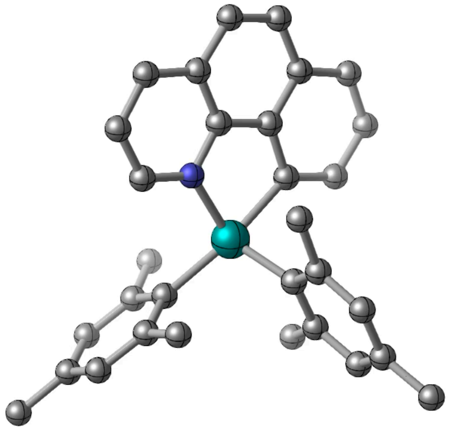

Fe	-0.141453	0.058208	0.53768	C	2.517223	-3.874642	1.513488
N	-0.894639	-1.327638	-0.682804	H	3.062989	-4.800089	1.679963
C	1.099258	-1.420036	1.093933	C	1.465402	-3.8451	0.569974
C	2.1533	-1.509388	1.999574	C	1.057075	-4.98313	-0.211274
H	2.47377	-0.626388	2.5497	H	1.584375	-5.923711	-0.071232
C	2.847586	-2.723335	2.213554	C	0.034106	-4.903501	-1.113792
H	3.663876	-2.752537	2.931894	H	-0.260081	-5.772588	-1.695593

C	-0.678146	-3.671131	-1.316991	H	4.506952	4.762069	-0.571031
C	-1.744877	-3.500626	-2.223741	H	5.593601	3.380023	-0.732455
H	-2.075738	-4.339374	-2.830289	C	1.122222	2.236429	2.242311
C	-2.365624	-2.265993	-2.327313	H	0.074665	2.54136	2.128017
H	-3.195198	-2.108093	-3.007359	H	1.120664	1.286955	2.795853
C	-1.916796	-1.200212	-1.533457	H	1.627069	2.978013	2.869564
H	-2.385664	-0.224583	-1.569162	C	1.741127	-0.192175	-2.187405
C	-0.285293	-2.54545	-0.556659	H	1.88262	-1.166477	-1.706882
C	0.783108	-2.613613	0.39201	H	0.697365	-0.155863	-2.516931
C	-1.674162	1.35444	0.497237	H	2.376079	-0.155091	-3.078213
C	-2.563637	1.351503	1.603411	H	-3.272326	3.803013	-1.304636
C	-3.669614	2.210098	1.650424	C	-1.057957	2.371834	-1.760634
H	-4.336004	2.176953	2.512321	H	-1.532698	2.933345	-2.572852
C	-3.943619	3.107072	0.613788	H	-0.114002	2.867938	-1.509113
C	-3.075734	3.116057	-0.481581	H	-0.784829	1.384624	-2.146464
C	-1.961193	2.267331	-0.548105	C	-2.342832	0.400337	2.768589
C	1.389613	1.078637	-0.005081	H	-2.4022	-0.650648	2.454001
C	2.073317	0.927641	-1.227658	H	-1.353169	0.540989	3.221604
C	1.798602	2.085349	0.895981	H	-3.08739	0.544857	3.558519
C	3.099291	1.824285	-1.554995	C	-5.121155	4.051683	0.688359
C	2.833565	2.95794	0.539431	H	-5.494148	4.30873	-0.309403
C	3.492142	2.850526	-0.689547	H	-5.9491	3.617908	1.260449
H	3.611602	1.712415	-2.509777	H	-4.846262	4.993561	1.182931
C	4.623592	3.784767	-1.052252	H	3.131429	3.738769	1.23828
H	4.681594	3.943101	-2.134864				

- Thermochemistry -

Temperature 298.150 Kelvin. Pressure 1.00000 Atm.

Zero-point correction= 0.521196 (Hartree/Particle)
Thermal correction to Energy= 0.552379
Thermal correction to Enthalpy= 0.553323
Thermal correction to Gibbs Free Energy= 0.457659
Sum of electronic and zero-point Energies= -2517.295206
Sum of electronic and thermal Energies= -2517.264023
Sum of electronic and thermal Enthalpies= -2517.263079
Sum of electronic and thermal Free Energies= -2517.358743

Charge = 0 Multiplicity = 2

^{4}Int-A

E(UB3LYP) = -2517.85155582 A.U.

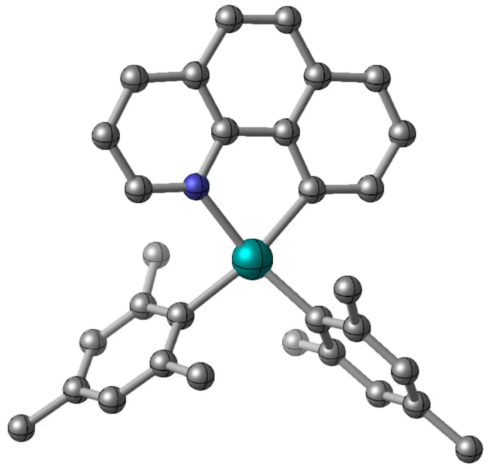

Fe	0.024477	0.053855	-0.020508	H	4.244282	-2.66822	1.719891
N	-1.220183	-1.550733	-0.545626	C	2.653398	-3.92174	1.024608
C	1.289542	-1.46856	0.472877	H	3.180247	-4.84969	1.233176
C	2.569592	-1.485421	1.01753	C	1.353782	-3.970329	0.471054
H	3.073947	-0.544345	1.224111	C	0.675201	-5.200339	0.158469
C	3.243559	-2.697439	1.294749	H	1.18822	-6.138203	0.357279

C	-0.580856	-5.21029	-0.377132	H	5.785695	3.624598	0.175166
H	-1.077463	-6.148007	-0.610402	C	0.972696	2.119229	2.331254
C	-1.276535	-3.981691	-0.643889	H	-0.07498	2.359548	2.120236
C	-2.573079	-3.904962	-1.194203	H	0.988159	1.10691	2.755418
H	-3.104795	-4.819361	-1.443903	H	1.341393	2.809889	3.096623
C	-3.157042	-2.667632	-1.410822	C	2.140067	0.45205	-2.331784
H	-4.152443	-2.579954	-1.831787	H	2.207014	-0.590407	-1.997029
C	-2.446669	-1.507155	-1.068495	H	1.148512	0.577365	-2.780704
H	-2.867906	-0.516324	-1.196922	H	2.886522	0.608861	-3.117431
C	-0.624863	-2.762147	-0.337655	H	-3.160404	3.867132	-1.601512
C	0.697907	-2.736077	0.214857	C	-0.975558	2.413266	-2.185588
C	-1.530523	1.347958	0.056767	H	-1.346066	3.182585	-2.87153
C	-2.409585	1.26101	1.165867	H	0.061043	2.650772	-1.92475
C	-3.513995	2.117446	1.271856	H	-0.952907	1.461444	-2.732037
H	-4.169224	2.038004	2.138876	C	-2.196529	0.219215	2.247668
C	-3.800695	3.068861	0.288689	H	-2.277715	-0.794639	1.836454
C	-2.949897	3.13902	-0.818655	H	-1.199973	0.295636	2.695497
C	-1.83596	2.297845	-0.945915	H	-2.933554	0.313837	3.052184
C	1.511213	1.342696	-0.027704	C	-5.012808	3.964903	0.399326
C	2.350611	1.392357	-1.164769	H	-4.844275	4.930744	-0.089871
C	1.802069	2.179865	1.069971	H	-5.890096	3.508975	-0.079916
C	3.41337	2.303832	-1.206136	H	-5.278437	4.153932	1.445332
C	2.877877	3.074871	0.998672	H	3.08515	3.722245	1.849825
C	3.693136	3.157562	-0.134147				
H	4.044753	2.341773	-2.092949				
C	4.866628	4.108678	-0.182527				
H	5.061223	4.454612	-1.203766				
H	4.697366	4.987629	0.449076				

- Thermochemistry -

Temperature 298.150 Kelvin. Pressure 1.00000 Atm.

Zero-point correction= 0.520699 (Hartree/Particle)
Thermal correction to Energy= 0.552182
Thermal correction to Enthalpy= 0.553126
Thermal correction to Gibbs Free Energy= 0.455972
Sum of electronic and zero-point Energies= -2517.330857
Sum of electronic and thermal Energies= -2517.299374
Sum of electronic and thermal Enthalpies= -2517.298430
Sum of electronic and thermal Free Energies= -2517.395584

Charge = 0 Multiplicity = 4

^{6}Int-A

E(UB3LYP) = -2517.83289945 A.U.

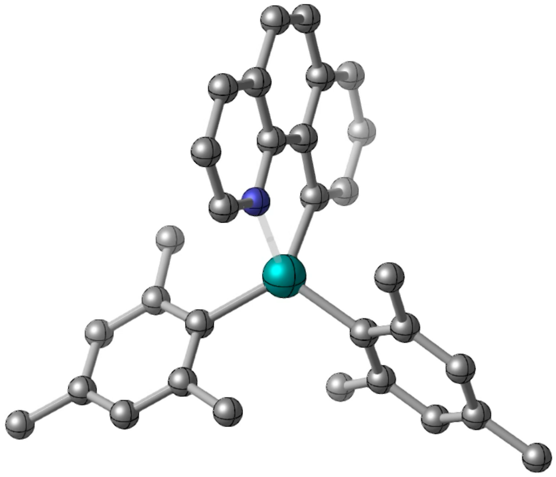

Fe	0.041407	0.267474	0.085105	C	1.847987	-2.577105	3.00887
N	-0.651639	-1.330799	-1.134954	H	2.406442	-2.577961	3.942142
C	0.730416	-1.295891	1.24427	C	1.529646	-3.781743	2.401716
C	1.451667	-1.349283	2.433692	H	1.831084	-4.724997	2.850423
H	1.717415	-0.428586	2.950649	C	0.805364	-3.792194	1.188828

C	0.435503	-5.008571	0.515426	H	5.278948	3.885575	-2.563681
H	0.735175	-5.955072	0.958859	H	5.07977	4.805228	-1.058931
C	-0.278787	-4.995561	-0.647184	H	6.063019	3.339242	-1.079515
H	-0.556503	-5.923441	-1.139381	C	1.374839	2.704924	1.688003
C	-0.682081	-3.754652	-1.247426	H	0.327556	2.968083	1.498029
C	-1.426622	-3.664388	-2.441204	H	1.360291	1.827631	2.348422
H	-1.723508	-4.573951	-2.956371	H	1.840154	3.526839	2.241977
C	-1.777806	-2.422111	-2.945988	C	1.956664	0.060529	-2.620618
H	-2.355599	-2.325572	-3.858293	H	1.947853	-0.922181	-2.133462
C	-1.367744	-1.27251	-2.25831	H	0.938837	0.235262	-2.991044
H	-1.614377	-0.278573	-2.618422	H	2.62097	-0.001063	-3.488865
C	-0.314747	-2.543084	-0.607481	H	-3.472817	4.009823	-0.552723
C	0.425756	-2.540962	0.627426	C	-1.169496	2.92168	-1.391144
C	-1.664097	1.304035	0.52832	H	-1.660354	3.726877	-1.948062
C	-2.523247	0.918659	1.586554	H	-0.203087	3.292233	-1.032567
C	-3.688705	1.645864	1.859521	H	-0.940095	2.118067	-2.10264
H	-4.333132	1.334173	2.680929	C	-2.205279	-0.290703	2.443848
C	-4.049296	2.764269	1.100781	H	-2.11101	-1.196908	1.833497
C	-3.207687	3.142391	0.050898	H	-1.246988	-0.174744	2.962205
C	-2.033502	2.43453	-0.242373	H	-2.981574	-0.467732	3.19596
C	1.665162	1.373898	-0.462492	C	-5.327302	3.518089	1.386911
C	2.384167	1.151127	-1.659189	H	-5.259259	4.562395	1.063148
C	2.108566	2.409865	0.393686	H	-6.179256	3.069053	0.858197
C	3.493574	1.943371	-1.980748	H	-5.569521	3.506292	2.45548
C	3.221021	3.188819	0.04996	H	3.542384	3.986168	0.719385
C	3.929719	2.969507	-1.135886				
H	4.030038	1.759858	-2.911226				
C	5.14736	3.795858	-1.479648				

- Thermochemistry -

Temperature 298.150 Kelvin. Pressure 1.00000 Atm.

Zero-point correction= 0.519432 (Hartree/Particle)
Thermal correction to Energy= 0.551424
Thermal correction to Enthalpy= 0.552368
Thermal correction to Gibbs Free Energy= 0.451962
Sum of electronic and zero-point Energies= -2517.313468
Sum of electronic and thermal Energies= -2517.281476
Sum of electronic and thermal Enthalpies= -2517.280531
Sum of electronic and thermal Free Energies= -2517.380937

Charge = 0 Multiplicity = 6

^{2}Int-A_bzq

E(UB3LYP) = -3073.45282449 A.U.

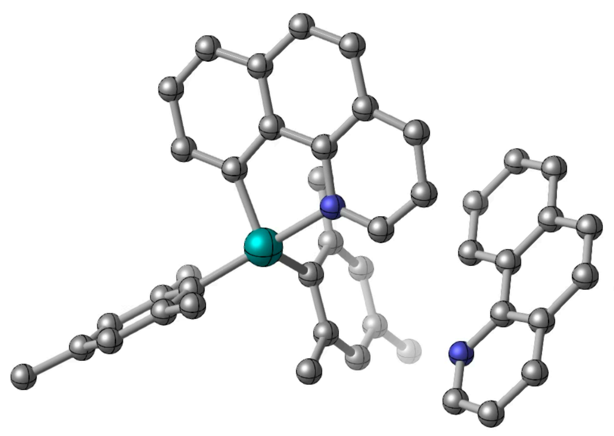

Fe	-0.868644	-0.128328	0.461595	H	-1.710238	-3.02708	4.673859
N	0.382917	-1.332631	-0.672017	C	-0.405935	-3.956671	3.252499
C	-0.787847	-1.572687	1.72026	H	-0.260294	-4.854701	3.847675
C	-1.410015	-1.749347	2.953492	C	0.229714	-3.842489	1.99446
H	-2.054335	-0.971075	3.350449	C	1.074199	-4.861816	1.428549
C	-1.214973	-2.928434	3.710615	H	1.240092	-5.771492	2.000658

C	1.659033	-4.711614	0.202139	C	5.573616	-1.091149	-2.006685
H	2.289944	-5.494798	-0.209315	C	4.767909	-0.53899	-3.030241
C	1.450147	-3.520344	-0.574106	C	3.78228	0.432613	-2.689263
C	1.991206	-3.287	-1.857451	C	3.631369	0.818165	-1.338453
H	2.624524	-4.039153	-2.32024	C	4.429193	0.257164	-0.356814
C	1.712311	-2.10057	-2.513366	C	4.90594	-0.965054	-4.395159
H	2.124664	-1.889955	-3.493318	C	2.925468	0.96084	-3.736408
C	0.911123	-1.136303	-1.877757	C	3.088543	0.50069	-5.07512
H	0.714208	-0.175595	-2.340158	C	4.101597	-0.469887	-5.377101
C	0.633681	-2.506148	-0.02411	C	1.250685	1.935016	-5.700846
C	0.015832	-2.650638	1.257104	C	1.165126	2.319092	-4.347676
C	-2.487801	0.741568	1.10238	H	5.666334	-1.70561	-4.630006
C	-2.644188	1.523103	2.273007	H	6.025607	-1.13877	0.088863
C	-3.905771	2.011999	2.63867	H	6.321919	-1.834187	-2.271418
H	-3.999286	2.611755	3.543901	H	2.868668	1.542183	-1.078478
C	-5.050455	1.755672	1.877435	H	4.290524	0.558983	0.67615
C	-4.899119	0.99233	0.716892	H	4.209614	-0.804804	-6.405574
C	-3.650385	0.488833	0.33011	H	0.563944	2.350806	-6.431199
C	0.461593	1.249056	0.674541	H	0.404649	3.030492	-4.030333
C	1.555473	1.230073	1.565012	C	2.219738	1.019734	-6.060591
C	0.288563	2.379025	-0.158489	H	2.321523	0.686962	-7.090607
C	2.399037	2.346609	1.653966	N	1.965104	1.85768	-3.396562
C	1.161657	3.469667	-0.054255	C	-3.566059	-0.330438	-0.948017
C	2.221431	3.479318	0.855108	H	-2.88656	0.125157	-1.680947
H	3.228927	2.324086	2.359585	H	-3.203701	-1.34985	-0.757124
H	-5.773297	0.782099	0.101	H	-4.544916	-0.425952	-1.429843
H	1.01324	4.327655	-0.708762	C	-1.460058	1.879381	3.147421
C	5.406518	-0.702495	-0.690277	H	-0.799068	1.020895	3.289352

H	-0.849016	2.662046	2.684219
H	-1.782053	2.236767	4.132073
C	-6.407116	2.261161	2.311364
H	-7.078886	2.394367	1.456015
H	-6.894803	1.556372	2.999029
H	-6.329942	3.220154	2.836169
C	1.881658	0.024524	2.418548
H	1.045178	-0.268204	3.059267
H	2.108539	-0.845035	1.791526
H	2.752718	0.215829	3.054255
C	-0.816843	2.439936	-1.193945
H	-0.734692	1.618106	-1.918333
H	-1.807758	2.371536	-0.732408
H	-0.771745	3.370341	-1.76899
C	3.178599	4.645906	0.932092
H	4.058655	4.47782	0.29621
H	2.70768	5.575907	0.595257
H	3.544408	4.799553	1.953643

- Thermochemistry -

Temperature 298.150 Kelvin. Pressure 1.00000 Atm.

Zero-point correction= 0.706194 (Hartree/Particle)
Thermal correction to Energy= 0.748386
Thermal correction to Enthalpy= 0.749330
Thermal correction to Gibbs Free Energy= 0.628838
Sum of electronic and zero-point Energies= -3072.746631
Sum of electronic and thermal Energies= -3072.704439
Sum of electronic and thermal Enthalpies= -3072.703494
Sum of electronic and thermal Free Energies= -3072.823987

Charge = 0 Multiplicity = 2

^{4}Int-A_bzq

E(UB3LYP) = -3073.47437833 A.U.

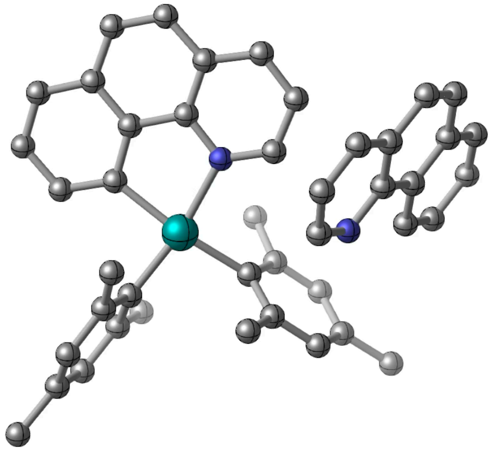

Fe	-0.575543	-0.196485	0.833189	C	-2.764822	-3.682275	2.495359
N	0.795968	-1.444854	-0.137524	H	-3.613642	-3.905561	3.137837
C	-1.351884	-1.994538	1.41763	C	-2.038393	-4.722912	1.939474
C	-2.426963	-2.334382	2.232835	H	-2.308613	-5.757096	2.138686
H	-3.034975	-1.54642	2.670747	C	-0.933517	-4.442299	1.103958

C	-0.130155	-5.465556	0.489053	H	0.959431	4.550425	-0.690147
H	-0.388466	-6.505622	0.673281	C	6.191108	1.428584	-1.218572
C	0.934158	-5.157654	-0.309145	C	6.303628	0.558694	-2.287372
H	1.530122	-5.94236	-0.766894	C	5.192275	0.289711	-3.120995
C	1.294634	-3.789604	-0.559606	C	3.951437	0.94061	-2.854731
C	2.383232	-3.387774	-1.361992	C	3.859727	1.82418	-1.755741
H	3.005722	-4.140824	-1.838251	C	4.959631	2.059541	-0.949603
C	2.652065	-2.040789	-1.533494	C	5.287889	-0.642122	-4.210985
H	3.484402	-1.706853	-2.140749	C	2.805683	0.643718	-3.698238
C	1.832167	-1.093386	-0.900224	C	2.943412	-0.29738	-4.758804
H	2.01084	-0.029598	-0.99155	C	4.210534	-0.927593	-4.994427
C	0.514186	-2.770426	0.037318	C	0.606658	0.046027	-5.251168
C	-0.611482	-3.078122	0.869352	C	0.575392	0.962918	-4.18154
C	-2.241833	0.69775	1.378502	H	6.243938	-1.126066	-4.393711
C	-2.387966	1.334827	2.62798	H	7.049423	1.620854	-0.580755
C	-3.594634	1.97159	2.948305	H	7.247762	0.06169	-2.496158
H	-3.690532	2.470041	3.912108	H	2.910594	2.302114	-1.541884
C	-4.678989	1.980843	2.065765	H	4.863633	2.724368	-0.097411
C	-4.53579	1.313236	0.844794	H	4.291236	-1.641098	-5.81046
C	-3.346244	0.660806	0.497121	H	-0.291893	-0.151443	-5.826857
C	0.604314	1.452549	0.737578	H	-0.351696	1.476319	-3.933795
C	1.732888	1.516192	1.591966	C	1.800786	-0.584463	-5.537557
C	0.356037	2.568661	-0.097203	H	1.875425	-1.301366	-6.351389
C	2.537917	2.664454	1.63145	N	1.625919	1.256426	-3.427771
C	1.174586	3.703405	-0.03924	C	-3.277546	-0.106949	-0.804846
C	2.270881	3.774733	0.827143	H	-2.469962	0.256395	-1.449676
H	3.396715	2.690624	2.301403	H	-3.072662	-1.166716	-0.608859
H	-5.375246	1.29089	0.150918	H	-4.214447	-0.036481	-1.367281

C	-1.261166	1.330106	3.634307
H	-0.941722	0.302015	3.848792
H	-0.385103	1.858735	3.243073
H	-1.555701	1.796621	4.580032
C	-5.982028	2.650411	2.437447
H	-6.492493	3.052415	1.55515
H	-6.672321	1.941113	2.914297
H	-5.823821	3.472551	3.143945
C	2.116253	0.338909	2.46866
H	1.300766	0.047313	3.139793
H	2.353061	-0.542858	1.861892
H	2.991669	0.564293	3.087032
C	-0.793722	2.55406	-1.078324
H	-0.777914	1.630516	-1.66597
H	-1.759199	2.585794	-0.561503
H	-0.745894	3.396848	-1.776307
C	3.16224	4.994743	0.847765
H	3.840576	5.003457	-0.016373
H	2.577386	5.920837	0.803277
H	3.781703	5.02574	1.750519

- Thermochemistry -

Temperature 298.150 Kelvin. Pressure 1.00000 Atm.

Zero-point correction= 0.705065 (Hartree/Particle)
Thermal correction to Energy= 0.747940
Thermal correction to Enthalpy= 0.748884
Thermal correction to Gibbs Free Energy= 0.625000
Sum of electronic and zero-point Energies= -3072.769314
Sum of electronic and thermal Energies= -3072.726438
Sum of electronic and thermal Enthalpies= -3072.725494
Sum of electronic and thermal Free Energies= -3072.849378

Charge = 0 Multiplicity = 4

[6]Int-A_bzq

E(UB3LYP) = -3073.46150286 A.U.

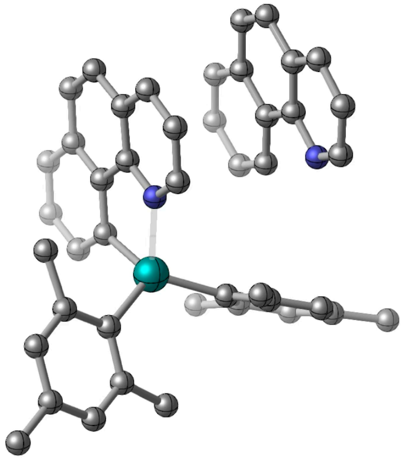

Fe	-0.444016	0.31404	0.990965	C	0.830044	-3.249754	3.355817
N	-0.050197	-0.689656	-0.856584	H	0.90949	-3.585824	4.387124
C	0.213608	-1.477461	1.776772	C	1.225196	-4.090881	2.328423
C	0.326713	-1.960012	3.078225	H	1.613524	-5.083566	2.542934
H	0.036363	-1.329876	3.916538	C	1.11131	-3.668737	0.985126

C	1.466442	-4.516391	-0.120151	H	1.690958	4.755762	-0.624392
H	1.864083	-5.505481	0.094131	C	4.141603	-1.57526	0.159904
C	1.30089	-4.109822	-1.410907	C	4.24308	-2.183288	-1.077342
H	1.562975	-4.762411	-2.239057	C	3.850985	-1.500108	-2.25189
C	0.770754	-2.810032	-1.708598	C	3.339324	-0.174166	-2.145798
C	0.537241	-2.35209	-3.020376	C	3.255431	0.43095	-0.873019
H	0.779576	-2.993001	-3.86245	C	3.652451	-0.257755	0.259025
C	0.009583	-1.089349	-3.221943	C	3.937237	-2.128463	-3.540216
H	-0.174114	-0.703264	-4.21785	C	2.893629	0.505755	-3.349091
C	-0.268745	-0.286874	-2.108696	C	2.989511	-0.159608	-4.605708
H	-0.684125	0.704022	-2.230203	C	3.528745	-1.486978	-4.670515
C	0.454076	-1.940014	-0.635109	C	1.974556	1.783742	-5.615415
C	0.606728	-2.361581	0.733188	C	1.930226	2.356918	-4.329107
C	-2.470882	0.627189	1.183777	H	4.330886	-3.140372	-3.594708
C	-2.980775	1.777108	1.843358	H	4.431173	-2.114653	1.057359
C	-4.360813	2.000509	1.928619	H	4.612048	-3.201919	-1.162066
H	-4.725414	2.892425	2.437317	H	2.85806	1.434991	-0.791619
C	-5.286558	1.104126	1.386427	H	3.572124	0.223343	1.22727
C	-4.791651	-0.04234	0.76168	H	3.591971	-1.974683	-5.640045
C	-3.415474	-0.294462	0.661731	H	1.593181	2.33146	-6.471283
C	0.793303	1.945985	1.123216	H	1.51141	3.352869	-4.19202
C	1.751373	2.067769	2.162465	C	2.513028	0.518766	-5.74851
C	0.773588	2.960844	0.140173	H	2.56888	0.03319	-6.719763
C	2.691779	3.102446	2.151613	N	2.371492	1.752563	-3.234344
C	1.725502	3.99268	0.152908	C	-2.987236	-1.579772	-0.020326
C	2.711122	4.067777	1.136661	H	-2.637384	-1.398284	-1.043475
H	3.42107	3.169519	2.958388	H	-2.165613	-2.063425	0.516392
H	-5.49536	-0.764067	0.347971	H	-3.817342	-2.291765	-0.079922

C	-2.059651	2.796146	2.48515
H	-1.325899	2.314505	3.141288
H	-1.482267	3.347093	1.735656
H	-2.623027	3.520976	3.082717
C	-6.772286	1.345934	1.517661
H	-7.333907	0.837599	0.726401
H	-7.151674	0.971162	2.478151
H	-7.010079	2.41476	1.473582
C	1.765677	1.09573	3.32324
H	0.756612	0.952527	3.727479
H	2.116813	0.104181	3.019163
H	2.407909	1.448939	4.137036
C	-0.256368	2.987454	-0.970825
H	0.180457	2.61426	-1.904797
H	-1.138403	2.38398	-0.728473
H	-0.604443	4.009068	-1.163403
C	3.764032	5.150695	1.115877
H	4.702593	4.779824	0.681913
H	3.445502	6.01072	0.517119
H	3.996814	5.504868	2.126724

- Thermochemistry -

Temperature   298.150 Kelvin. Pressure   1.00000 Atm.

Zero-point correction=   0.704736 (Hartree/Particle)
Thermal correction to Energy=   0.747529
Thermal correction to Enthalpy=   0.748473
Thermal correction to Gibbs Free Energy=   0.624326
Sum of electronic and zero-point Energies=   -3072.756766
Sum of electronic and thermal Energies=   -3072.713974
Sum of electronic and thermal Enthalpies=   -3072.713029
Sum of electronic and thermal Free Energies=   -3072.837177

Charge = 0 Multiplicity = 6

^{2}TS2

E(UB3LYP) = -3073.38915869   A.U.

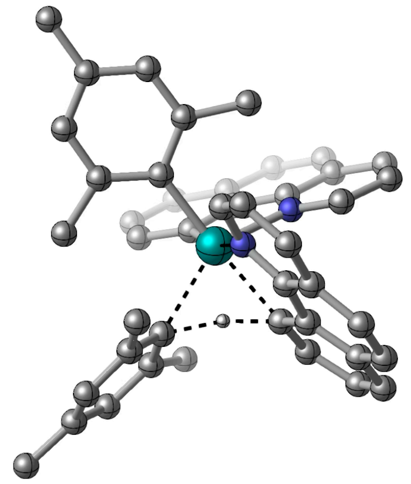

Fe	0.496472	0.051827	-0.350145	C	-0.193167	-1.432799	3.712301
N	0.689985	-1.880745	-1.095998	H	-0.401882	-1.041975	4.706313
C	0.248834	-0.944106	1.33943	C	-0.077501	-2.805023	3.530379
C	-0.037545	-0.528412	2.64563	H	-0.193085	-3.49461	4.362861
H	-0.107123	0.526292	2.875039	C	0.195206	-3.297446	2.241027

C	0.336964	-4.699131	1.944554	H	1.697658	5.168674	1.004243
H	0.229278	-5.410611	2.760144	C	4.918953	-0.959713	-1.442519
C	0.600543	-5.1452	0.681821	C	4.978358	-0.446688	-2.726443
H	0.705975	-6.207331	0.477708	C	3.874	0.255531	-3.255749
C	0.739211	-4.221203	-0.410022	C	2.722379	0.424724	-2.435887
C	0.994871	-4.594714	-1.740579	C	2.687342	-0.029396	-1.094766
H	1.107711	-5.645005	-1.996275	C	3.791087	-0.734276	-0.627576
C	1.097835	-3.610328	-2.713308	C	3.871305	0.783617	-4.592023
H	1.293424	-3.859262	-3.75054	C	1.536868	0.983136	-3.018055
C	0.939595	-2.269691	-2.349391	C	1.556025	1.487421	-4.346563
H	1.002294	-1.490073	-3.09616	C	2.768942	1.395919	-5.108542
C	0.600924	-2.835259	-0.130875	C	-0.783054	1.992309	-4.091747
C	0.339585	-2.354742	1.182419	C	-0.723867	1.436627	-2.807046
C	-1.553177	0.287445	-0.074866	H	4.772553	0.679668	-5.190649
C	-2.142608	1.267046	0.774679	H	5.758924	-1.52379	-1.045492
C	-3.537925	1.405671	0.894689	H	5.863558	-0.5877	-3.341128
H	-3.922238	2.179977	1.559208	H	2.079498	0.768214	-0.205285
C	-4.436761	0.601776	0.207617	H	3.803399	-1.125435	0.380708
C	-3.880167	-0.347214	-0.648261	H	2.774844	1.793824	-6.119447
C	-2.495342	-0.510815	-0.804792	H	-1.726483	2.381221	-4.458885
C	1.709355	1.746341	0.792654	H	-1.606237	1.389958	-2.182564
C	2.452491	1.500728	1.972705	C	0.35959	2.016161	-4.868704
C	1.48953	3.108835	0.438765	H	0.344788	2.420975	-5.876987
C	2.837062	2.564262	2.807708	N	0.394578	0.941275	-2.268224
C	1.906527	4.138126	1.287055	C	-2.117081	-1.559235	-1.834378
C	2.567403	3.891033	2.494333	H	-1.373101	-1.188342	-2.541503
H	3.382186	2.334377	3.722552	H	-1.710464	-2.466412	-1.378816
H	-4.54827	-0.982957	-1.228363	H	-2.996887	-1.856092	-2.414467

C	-1.378262	2.27252	1.612353
H	-0.319647	2.058742	1.660119
H	-1.484301	3.285532	1.201456
H	-1.772675	2.304108	2.63558
C	-5.931272	0.732612	0.383082
H	-6.46336	0.561824	-0.560245
H	-6.31584	-0.001263	1.104722
H	-6.206424	1.72646	0.753337
C	2.95246	0.14271	2.4249
H	2.550035	-0.677645	1.839629
H	4.048557	0.114498	2.372469
H	2.67157	-0.045293	3.467126
C	0.739503	3.532201	-0.801934
H	-0.241568	3.054428	-0.848284
H	0.585777	4.615762	-0.811065
H	1.276867	3.268569	-1.717271
C	2.962137	5.023814	3.411251
H	2.0865	5.433688	3.931957
H	3.675255	4.694399	4.174001
H	3.416701	5.850464	2.852563

- Thermochemistry -

Temperature 298.150 Kelvin. Pressure 1.00000 Atm.

Zero-point correction= 0.704097 (Hartree/Particle)
Thermal correction to Energy= 0.744547
Thermal correction to Enthalpy= 0.745492
Thermal correction to Gibbs Free Energy= 0.631103
Sum of electronic and zero-point Energies= -3072.685062
Sum of electronic and thermal Energies= -3072.644611
Sum of electronic and thermal Enthalpies= -3072.643667
Sum of electronic and thermal Free Energies= -3072.758056

Charge = 0 Multiplicity = 2

^{4}TS2

E(UB3LYP) = -3073.40322741 A.U.

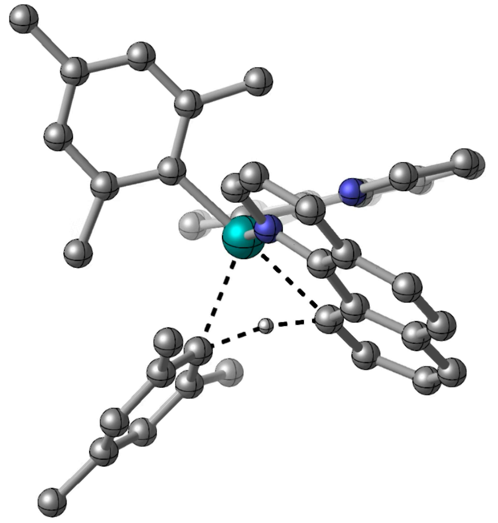

Fe	0.236315	0.108036	-0.257635	H	-0.688483	-1.694105	4.496396
N	0.685569	-2.047475	-1.406706	C	0.163317	-3.217126	3.250569
C	0.021812	-1.259942	1.172793	H	0.234493	-3.956179	4.044809
C	-0.427108	-0.998832	2.466057	C	0.575312	-3.562464	1.945332
H	-0.828928	-0.022345	2.707456	C	1.073644	-4.875821	1.636831
C	-0.347019	-1.955242	3.497369	H	1.153917	-5.597114	2.446716

C	1.430092	-5.227015	0.36931	C	4.799229	-0.702714	-2.628434
H	1.797941	-6.225988	0.150205	C	3.706391	0.00672	-3.174177
C	1.306257	-4.288641	-0.708775	C	2.587309	0.289174	-2.337002
C	1.60339	-4.608836	-2.049602	C	2.586857	-0.06438	-0.962249
H	1.966337	-5.603673	-2.295054	C	3.69077	-0.754538	-0.474332
C	1.414145	-3.658073	-3.036052	C	3.698323	0.432158	-4.54573
H	1.617404	-3.874175	-4.079554	C	1.414913	0.871006	-2.937358
C	0.946362	-2.387046	-2.662792	C	1.440798	1.29477	-4.296796
H	0.788714	-1.618821	-3.413479	C	2.622641	1.073973	-5.079543
C	0.84313	-2.973577	-0.428644	C	-0.847806	2.009486	-4.047181
C	0.482465	-2.582454	0.911939	C	-0.812609	1.518158	-2.737604
C	-1.725552	0.509514	0.069393	H	4.576047	0.231539	-5.154917
C	-2.206745	1.551834	0.896231	H	5.613072	-1.659119	-0.891115
C	-3.582886	1.845804	0.935574	H	5.651405	-0.932147	-3.263128
H	-3.91872	2.661822	1.574678	H	2.009154	0.753114	-0.10106
C	-4.525013	1.133017	0.201341	H	3.732175	-1.038089	0.571603
C	-4.051915	0.075011	-0.5793	H	2.62519	1.403187	-6.114849
C	-2.692116	-0.247801	-0.648584	H	-1.759921	2.456933	-4.426136
C	1.697896	1.834848	0.884627	H	-1.68721	1.568944	-2.101967
C	2.254103	1.553913	2.15173	C	0.281363	1.886468	-4.834144
C	1.595988	3.200468	0.511291	H	0.284705	2.229666	-5.865265
C	2.570422	2.593874	3.043718	N	0.270817	0.958915	-2.189227
C	1.937424	4.214307	1.411197	C	-2.30665	-1.429202	-1.508603
C	2.406084	3.932845	2.700599	H	-1.607463	-1.144098	-2.300637
H	2.971032	2.341815	4.025904	H	-1.80765	-2.200412	-0.915218
H	-4.762957	-0.525612	-1.145248	H	-3.186354	-1.877044	-1.981974
H	1.831792	5.255195	1.106114	C	-1.328102	2.422513	1.769332
C	4.777024	-1.099113	-1.302894	H	-0.42535	1.91155	2.097398

H	-0.99189	3.318298	1.235397	H	2.262454	-0.615336	1.935107
H	-1.880485	2.759392	2.653808	C	1.079458	3.62672	-0.844821
C	-5.9944	1.482602	0.237724	H	0.031272	3.338925	-0.976643
H	-6.295398	2.035368	-0.66256	H	1.143753	4.712819	-0.969636
H	-6.619781	0.58321	0.283693	H	1.643273	3.162764	-1.659462
H	-6.236805	2.109334	1.102606	C	2.718501	5.046292	3.672691
C	2.581512	0.153825	2.633168	H	1.799063	5.474047	4.094877
H	3.662687	0.052022	2.796139	H	3.329112	4.6908	4.509504
H	2.089358	-0.063349	3.588417	H	3.255834	5.867262	3.183151

- Thermochemistry -

Temperature 298.150 Kelvin. Pressure 1.00000 Atm.

Zero-point correction= 0.701477 (Hartree/Particle)
Thermal correction to Energy= 0.743085
Thermal correction to Enthalpy= 0.744030
Thermal correction to Gibbs Free Energy= 0.625530
Sum of electronic and zero-point Energies= -3072.701750
Sum of electronic and thermal Energies= -3072.660142
Sum of electronic and thermal Enthalpies= -3072.659198
Sum of electronic and thermal Free Energies= -3072.777698

Charge = 0 Multiplicity = 4

^{6}TS2

E(UB3LYP) = -3073.38404240 A.U.

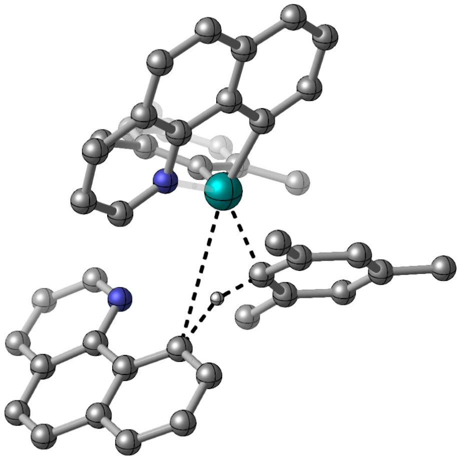

Fe	0.115128	0.073277	0.63387	C	-0.551509	-1.756658	4.662311
N	0.780136	-1.81319	-0.158538	H	-0.874883	-1.477981	5.663237
C	-0.016729	-1.058205	2.36571	C	-0.256347	-3.082429	4.385316
C	-0.431063	-0.763571	3.664814	H	-0.342687	-3.84447	5.15622
H	-0.667429	0.265141	3.935297	C	0.167265	-3.451479	3.089243

C	0.507582	-4.805856	2.743152	H	1.438073	4.433623	1.702901
H	0.423254	-5.571626	3.510905	C	5.50474	0.474906	-2.497801
C	0.936705	-5.139271	1.491669	C	5.193756	0.391182	-3.842025
H	1.199295	-6.165305	1.248339	C	3.853772	0.497497	-4.284442
C	1.053783	-4.14111	0.465735	C	2.80676	0.669132	-3.32063
C	1.505783	-4.41361	-0.841977	C	3.161496	0.725048	-1.95847
H	1.785035	-5.429487	-1.109437	C	4.473516	0.654071	-1.547622
C	1.592932	-3.389465	-1.770452	C	3.534569	0.457464	-5.684744
H	1.940826	-3.568975	-2.781752	C	1.441378	0.812175	-3.795582
C	1.212489	-2.096073	-1.386711	C	1.17937	0.804655	-5.19713
H	1.245582	-1.263132	-2.077513	C	2.255226	0.609364	-6.124959
C	0.702768	-2.801899	0.778142	C	-1.143077	1.202143	-4.679734
C	0.2669	-2.429667	2.102165	C	-0.786545	1.172301	-3.317717
C	-1.769198	0.782034	0.218731	H	4.346577	0.315642	-6.393961
C	-2.32627	1.909308	0.868676	H	6.539354	0.407216	-2.169866
C	-3.639027	2.330595	0.597279	H	5.979367	0.256274	-4.581352
H	-4.032061	3.208126	1.110867	H	2.20867	0.915136	-0.70449
C	-4.457091	1.650353	-0.303515	H	4.725145	0.740652	-0.492895
C	-3.93784	0.49738	-0.905852	H	2.027203	0.592853	-7.187722
C	-2.634258	0.059375	-0.649971	H	-2.175965	1.369799	-4.968083
C	1.922093	1.301516	0.42153	H	-1.543555	1.308894	-2.550165
C	2.657747	0.74753	1.504231	C	-0.153036	1.002292	-5.620719
C	1.526142	2.670982	0.487842	H	-0.380124	1.002097	-6.684048
C	2.852392	1.509827	2.666739	N	0.450165	0.96853	-2.881748
C	1.760831	3.395392	1.651186	C	-2.17128	-1.227233	-1.303931
C	2.402063	2.824866	2.764317	H	-1.265731	-1.071737	-1.897725
H	3.39426	1.066855	3.500505	H	-1.927467	-1.988112	-0.552411
H	-4.571201	-0.075974	-1.582492	H	-2.938393	-1.644608	-1.965001

C	-1.543104	2.711982	1.889221
H	-2.198847	3.088984	2.682792
H	-0.75257	2.122989	2.363086
H	-1.055391	3.581281	1.432039
C	-5.853084	2.132282	-0.623134
H	-6.554581	1.295516	-0.721575
H	-6.23359	2.806175	0.152088
H	-5.876083	2.682085	-1.574079
C	3.278993	-0.630227	1.433297
H	2.757912	-1.329379	2.095696
H	3.245327	-1.036464	0.421668
H	4.325735	-0.591103	1.755578
C	0.855083	3.315435	-0.696805
H	-0.126753	2.863319	-0.875427
H	0.72394	4.391191	-0.543078
H	1.440774	3.150443	-1.607192
C	2.592372	3.629191	4.026999
H	2.949607	4.641569	3.805401
H	1.642135	3.736027	4.566668
H	3.307213	3.152974	4.705325

- Thermochemistry -

 Temperature   298.150 Kelvin. Pressure   1.00000 Atm.

 Zero-point correction=   0.698277 (Hartree/Particle)
 Thermal correction to Energy=   0.740939
 Thermal correction to Enthalpy=   0.741883
 Thermal correction to Gibbs Free Energy=   0.619859
 Sum of electronic and zero-point Energies=   -3072.685766
 Sum of electronic and thermal Energies=   -3072.643103
 Sum of electronic and thermal Enthalpies=   -3072.642159
 Sum of electronic and thermal Free Energies=   -3072.764183

 Charge = 0 Multiplicity = 6

[2]Int-B

E(UB3LYP) = -2723.24220749   A.U.

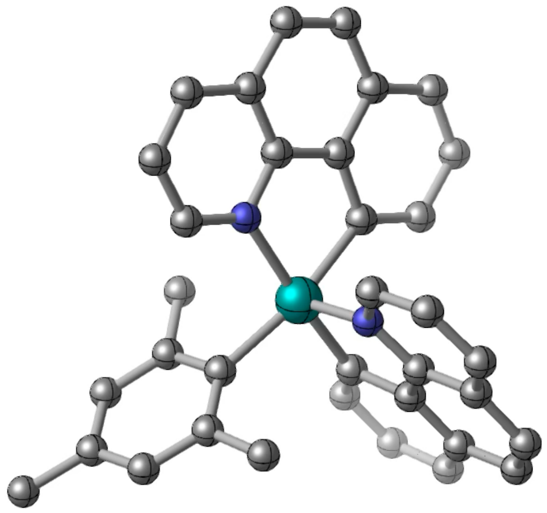

Fe	4.776175	3.286742	19.587625	C	4.212235	-0.667448	21.29038
N	3.026203	3.31909	18.724785	H	4.731199	-1.401414	21.903204
N	5.321566	5.246512	19.122058	C	2.923861	-0.946652	20.845139
C	4.240481	1.511648	20.181992	H	2.439532	-1.884647	21.104838
C	4.866773	0.540308	20.963789	C	2.243255	-0.00163	20.049679
H	5.87905	0.700662	21.329931	C	0.910569	-0.17452	19.530723

H	0.382286	-1.097777	19.756977	H	6.862922	7.342738	16.989229
C	0.304276	0.78633	18.769121	C	6.012375	5.681432	18.067096
H	-0.701658	0.633396	18.387632	H	6.341866	4.91466	17.374008
C	0.974608	2.019951	18.45232	C	4.883281	6.148315	20.049979
C	0.436558	3.063934	17.675683	C	4.159782	5.610475	21.165323
H	-0.564043	2.973831	17.261859	C	6.040269	2.431033	18.292025
C	1.198082	4.202758	17.449737	C	5.88259	1.848296	17.01551
H	0.812984	5.025877	16.857803	C	7.348229	2.48783	18.824761
C	2.487226	4.299019	17.991047	C	7.001091	1.378526	16.312016
H	3.104483	5.174811	17.832475	C	8.451411	1.998594	18.116729
C	2.282914	2.195825	18.959017	C	8.295501	1.445096	16.842505
C	2.92561	1.204423	19.747157	H	6.860619	0.935009	15.326237
C	3.960193	4.19977	21.210666	H	9.444891	2.048443	18.562872
C	3.251719	3.724816	22.312383	C	9.486379	0.951944	16.052546
H	3.062535	2.657313	22.406538	H	9.219309	0.107135	15.407522
C	2.764931	4.589264	23.319967	H	9.888112	1.740007	15.400572
H	2.217005	4.172586	24.162709	H	10.301234	0.632256	16.711691
C	2.969906	5.9587	23.252246	C	7.554678	3.123179	20.189663
H	2.590697	6.617624	24.029668	H	7.700836	4.20757	20.115868
C	3.680013	6.505844	22.16024	H	6.695841	2.961171	20.866661
C	3.936845	7.914298	22.013509	H	8.421309	2.704703	20.713154
H	3.564814	8.589316	22.780851	C	4.511562	1.700978	16.391705
C	4.629689	8.411919	20.946695	H	3.858303	1.090963	17.025813
H	4.815256	9.478624	20.854343	H	4.015315	2.670796	16.275751
C	5.130632	7.537788	19.922434	H	4.564188	1.227811	15.405058
C	5.857145	7.969014	18.793151				
H	6.064945	9.027541	18.660333				
C	6.300239	7.040063	17.865506				

- Thermochemistry -

Temperature 298.150 Kelvin. Pressure 1.00000 Atm.

Zero-point correction= 0.520418 (Hartree/Particle)
Thermal correction to Energy= 0.551335
Thermal correction to Enthalpy= 0.552280
Thermal correction to Gibbs Free Energy= 0.458095
Sum of electronic and zero-point Energies= -2722.721790
Sum of electronic and thermal Energies= -2722.690872
Sum of electronic and thermal Enthalpies= -2722.689928
Sum of electronic and thermal Free Energies= -2722.784113

Charge = 0 Multiplicity = 2

^{4}Int-B

E(UB3LYP) = -2723.26360528 A.U.

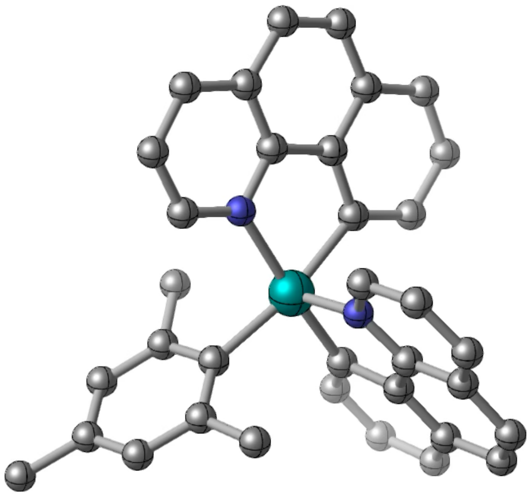

Fe	4.646262	3.262232	19.452487	H	5.694	0.642994	21.061133
N	2.706688	3.368639	18.511741	C	4.005437	-0.701327	21.072136
N	5.157409	5.18819	18.96104	H	4.52924	-1.454428	21.656551
C	4.03591	1.48995	19.98894	C	2.698355	-0.936139	20.66941
C	4.666265	0.498877	20.735729	H	2.195159	-1.863048	20.933278

C	2.011756	0.034123	19.90759	H	5.787672	8.976548	18.395264
C	0.656938	-0.142756	19.454712	C	5.90324	6.991858	17.567126
H	0.143899	-1.06626	19.712628	H	6.316753	7.299873	16.613059
C	0.015136	0.811669	18.719761	C	5.664745	5.630595	17.809586
H	-1.008088	0.660325	18.386529	H	5.897621	4.868022	17.073825
C	0.675965	2.038429	18.369235	C	4.862217	6.086477	19.947211
C	0.08075	3.068517	17.61235	C	4.328013	5.541885	21.16112
H	-0.941168	2.95338	17.260364	C	6.19285	2.472238	18.436976
C	0.802026	4.216666	17.32513	C	6.102868	1.889012	17.155546
H	0.368395	5.024489	16.74562	C	7.465427	2.561291	19.042996
C	2.118964	4.326329	17.797921	C	7.257115	1.426135	16.507671
H	2.72005	5.20826	17.59649	C	8.603472	2.086061	18.379267
C	2.011446	2.234251	18.804388	C	8.519464	1.517232	17.103564
C	2.698468	1.236083	19.577701	H	7.170083	0.977055	15.518492
C	4.141248	4.132034	21.229814	H	9.576541	2.160289	18.864592
C	3.642498	3.641806	22.433884	C	9.75889	1.040392	16.381035
H	3.479783	2.571803	22.549051	H	9.52945	0.228836	15.681487
C	3.335609	4.496316	23.518472	H	10.217318	1.850512	15.79712
H	2.945568	4.07218	24.441431	H	10.519467	0.680038	17.082865
C	3.517507	5.867635	23.423901	C	7.609667	3.197651	20.41192
H	3.275553	6.518903	24.260468	H	7.385815	4.271499	20.371535
C	4.025097	6.42829	22.23001	H	6.907506	2.762632	21.133896
C	4.248621	7.83875	22.051242	H	8.62213	3.080857	20.813066
H	4.009802	8.508444	22.874227	C	4.758485	1.743657	16.474083
C	4.749573	8.344273	20.885239	H	4.075508	1.143125	17.086184
H	4.91194	9.412342	20.768642	H	4.27242	2.716814	16.336125
C	5.078767	7.477464	19.787283	H	4.847582	1.267572	15.491746
C	5.60954	7.916624	18.556621				

- Thermochemistry -

Temperature 298.150 Kelvin. Pressure 1.00000 Atm.

Zero-point correction= 0.519189 (Hartree/Particle)
Thermal correction to Energy= 0.550773
Thermal correction to Enthalpy= 0.551717
Thermal correction to Gibbs Free Energy= 0.454267
Sum of electronic and zero-point Energies= -2722.744416
Sum of electronic and thermal Energies= -2722.712832
Sum of electronic and thermal Enthalpies= -2722.711888
Sum of electronic and thermal Free Energies= -2722.809338

Charge = 0 Multiplicity = 4

^{6}Int-B

E(UB3LYP) = -2723.23450761 A.U.

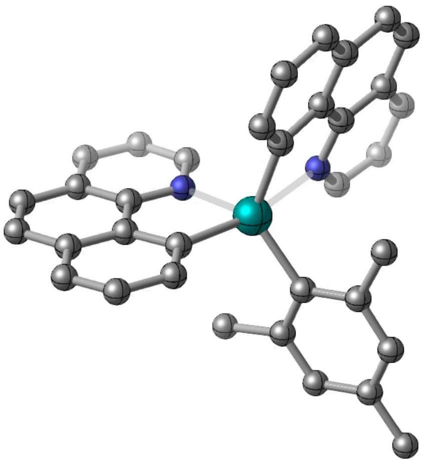

Fe	4.479434	3.026701	19.506261	H	5.143804	0.248215	21.288096
N	2.536147	3.149725	18.492914	C	3.400616	-1.010225	21.127122
N	4.87198	5.263826	18.771925	H	3.827566	-1.790641	21.753455
C	3.642857	1.186253	20.062805	C	2.128848	-1.17609	20.601335
C	4.146824	0.159344	20.857279	H	1.556281	-2.07704	20.808486

C	1.562966	-0.168321	19.788128
C	0.247607	-0.278764	19.217922
H	-0.32996	-1.178309	19.41776
C	-0.279786	0.714446	18.445467
H	-1.276445	0.620251	18.02293
C	0.466884	1.910885	18.173219
C	-0.027754	2.974294	17.391186
H	-1.023152	2.905075	16.960255
C	0.756816	4.095382	17.183676
H	0.403996	4.932017	16.590709
C	2.036735	4.141784	17.754965
H	2.679661	5.001836	17.615318
C	1.771567	2.040084	18.718176
C	2.338163	0.999135	19.537084
C	4.353622	4.136041	21.243877
C	4.082522	3.639641	22.516801
H	3.919722	2.571277	22.647149
C	4.022239	4.482759	23.649027
H	3.809509	4.058548	24.627875
C	4.241311	5.844455	23.525079
H	4.206671	6.493213	24.397169
C	4.522926	6.40565	22.257894
C	4.793519	7.807615	22.082984
H	4.756256	8.453241	22.957353
C	5.109278	8.328405	20.862227
H	5.328121	9.38692	20.749278
C	5.164089	7.488703	19.698985
C	5.508436	7.957307	18.413448
H	5.745549	9.008825	18.273218
C	5.551309	7.074312	17.347893
H	5.824263	7.403565	16.351017
C	5.223587	5.727257	17.576727
H	5.241753	4.998689	16.770103
C	4.862184	6.106787	19.83742
C	4.560065	5.537671	21.130092
C	6.264077	2.532472	18.59643
C	6.239599	1.886732	17.338315
C	7.525214	2.819433	19.166143
C	7.433208	1.547922	16.685731
C	8.705355	2.46657	18.49765
C	8.680171	1.832088	17.250863
H	7.391226	1.047522	15.718407
H	9.667874	2.690352	18.957459
C	9.961642	1.489389	16.525938
H	9.826295	0.636664	15.851375
H	10.311919	2.332949	15.915146
H	10.767241	1.244601	17.227384
C	7.623658	3.531455	20.500614
H	7.243922	4.557707	20.425249
H	7.014585	3.038225	21.266473
H	8.656926	3.579266	20.861472
C	4.918386	1.552608	16.671459
H	4.276972	0.962367	17.336714
H	4.354705	2.460332	16.419932
H	5.061126	0.985072	15.745664

- Thermochemistry -

Temperature 298.150 Kelvin. Pressure 1.00000 Atm.

Zero-point correction= 0.518109 (Hartree/Particle)
Thermal correction to Energy= 0.550233
Thermal correction to Enthalpy= 0.551177
Thermal correction to Gibbs Free Energy= 0.451344
Sum of electronic and zero-point Energies= -2722.716398
Sum of electronic and thermal Energies= -2722.684275
Sum of electronic and thermal Enthalpies= -2722.683331
Sum of electronic and thermal Free Energies= -2722.783163

Charge = 0 Multiplicity = 6

10. References

1. S. Stoll and A. Schweiger, *J. Magn. Reson.*, 2006, **178**, 42–55.
2. CrysAlisPro 171.41.116a ed., Rigaku Corporation, Oxford, UK, **2021**.
3. G. Sheldrick, *Acta Crystallogr. A*, 2015, **71**, 3–8.
4. G. Sheldrick, *Acta Crystallogr. C*, 2015, **71**, 3–8.
5. O. V. Dolomanov, L. J. Bourhis, R. J. Gildea, J. A. K. Howard and H. Puschmann, *J. Appl. Cryst.*, 2009, **42**, 339–341.
6. C. P. Agilent and P. CrysAlis, *Yarnton, Oxfordshire, England*, 2014, **2014**.
7. M. J. Frisch, G. W. Trucks, H. B. Schlegel, G. E. Scuseria, M. A. Robb, J. R. Cheeseman, G. Scalmani, V. Barone, G. A. Petersson, H. Nakatsuji, X. Li, M. Caricato, A. V. Marenich, J. Bloino, B. G. Janesko, R. Gomperts, B. Mennucci, H. P. Hratchian, J. V. Ortiz, A. F. Izmaylov, J. L. Sonnenberg, Williams, F. Ding, F. Lipparini, F. Egidi, J. Goings, B. Peng, A. Petrone, T. Henderson, D. Ranasinghe, V. G. Zakrzewski, J. Gao, N. Rega, G. Zheng, W. Liang, M. Hada, M. Ehara, K. Toyota, R. Fukuda, J. Hasegawa, M. Ishida, T. Nakajima, Y. Honda, O. Kitao, H. Nakai, T. Vreven, K. Throssell, J. A. Montgomery Jr., J. E. Peralta, F. Ogliaro, M. J. Bearpark, J. J. Heyd, E. N. Brothers, K. N. Kudin, V. N. Staroverov, T. A. Keith, R. Kobayashi, J. Normand, K. Raghavachari, A. P. Rendell, J. C. Burant, S. S. Iyengar, J. Tomasi, M. Cossi, J. M. Millam, M. Klene, C. Adamo, R. Cammi, J. W. Ochterski, R. L. Martin, K. Morokuma, O. Farkas, J. B. Foresman and D. J. Fox, *Gaussian 16*, 2016.
8. F. Neese, F. Wennmohs, U. Becker and C. Riplinger, *J. Chem. Phys.*, 2020, **152**.
9. A. D. Becke, *J. Chem. Phys.*, 1993, **98**, 5648–5652.
10. C. T. Lee, W. T. Yang and R. G. Parr, *Phys. Rev. B*, 1988, **37**, 785–789.
11. S. H. Vosko, L. Wilk and M. Nusair, *Can. J. Phys.*, 1980, **58**, 1200–1211.
12. P. J. Stephens, F. J. Devlin, C. F. Chabalowski and M. J. Frisch, *J. Phys. Chem-Us.*, 1994, **98**, 11623–11627.
13. S. Grimme, J. Antony, S. Ehrlich and H. Krieg, *J Chem Phys*, 2010, **132**.
14. F. Weigend and R. Ahlrichs, *Phys. Chem. Chem. Phys.*, 2005, **7**, 3297–3305.
15. S. Miertus, E. Scrocco and J. Tomasi, *Chem. Phys.*, 1981, **55**, 117–129.
16. S. Miertus and J. Tomasi, *Chem Phys*, 1982, **65**, 239–245.
17. J. L. Pascualahuir, E. Silla and I. Tunon, *J Comput. Chem.*, 1994, **15**, 1127–1138.
18. J. M. Tao, J. P. Perdew, V. N. Staroverov and G. E. Scuseria, *Phys. Rev. Lett.*, 2003, **91**.
19. V. N. Staroverov, G. E. Scuseria, J. M. Tao and J. P. Perdew, *J. Chem. Phys.*, 2003, **119**, 12129–12137.
20. T. Lu and F. W. Chen, *J. Comput. Chem.*, 2012, **33**, 580–592.
21. F. Neese, *Inorg. Chim. Acta.*, 2002, **337**, 181–192.
22. T. Nakajima and K. Hirao, *Chem. Rev.*, 2012, **112**, 385–402.
23. M. Römelt, S. F. Ye and F. Neese, *Inorg. Chem.*, 2009, **48**, 784–785.
24. R. Bjomsson, F. Neese and S. DeBeer, *Inorg. Chem.*, 2017, **56**, 1470–1477.
25. R. B. Bedford, P. B. Brenner, E. Carter, P. M. Cogswell, M. F. Haddow, J. N. Harvey, D. M. Murphy, J. Nunn and C. H. Woodall, *Angew. Chem. Int. Ed. Engl.*, 2014, **53**, 1804–1808.
26. S. Grimme, *J. Comput. chem.*, 2004, **25**, 1463–1473.
27. G. Petersson, T. G. Tensfeldt and J. Montgomery Jr, *J. of chem. phys.*, 1991, **94**, 6091–6101.
28. A. Klamt and G. Schüürmann, *J. Chem. Soc., Perkin Trans. 2*, 1993, 799–805.
29. C. Legault, *Sherbrooke, QC: http://www. cylview. org*, 2009.
