# Supplementary material for: Iron tris-mesityl: a homoleptic iron(ii) ferrate species for directed C–H activation
Source: Chem Sci. 2026 Feb 3. Online ahead of print. doi: 10.1039/d5sc08832a (PMC12896185; doi:10.1039/d5sc08832a)
Supplement: Supplementary file 1 [file SC-OLF-D5SC08832A-s001.pdf]

Supplementary Information (SI) for Chemical Science.
This journal is © The Royal Society of Chemistry 2026

Supporting Information for

Iron Tris-Mesityl: A Homoleptic Iron(II) Ferrate Species for Directed C-H Activation

Aleksa Radović,[a†] Maria C. Healy,[b†] Arnadeep Datta,[c] Deborshee Das,[c] Likun Cai,[a] Steven Diaz,[a]Achyut Ranjan Gogoi,[c] Nikki J. Wolford,[a] Stephanie H. Carpenter,[a] William W. Brenessel,[a] David McCamant[a*], Osvaldo Gutierrez[c*] and Michael L Neidig[b*]

Table of Contents

1. **Experimental Procedures** S3
2. **Mössbauer Spectra** S8
3. **EPR Spectra** S12
4. **Cyclic Voltammograms** S13
5. **UV-Vis Spectra** S15
6. **NMR Spectra** S19
7. **Transient Absorption Spectra and Kinetics.** S23
8. **Single Crystal X-Ray Diffraction** S28
9. **Density Functional Theory** S43
10. **References** S81

1. Experimental Procedures

1.1 General Considerations – All solvents and chemicals were purchased from commercial sources. All air and moisture sensitive manipulations were carried out in an MBraun inert-atmosphere (N_2) dry box equipped with a direct liquid nitrogen inlet line. All anhydrous solvents were further dried using activated alumina/4 Å molecular sieves and stored under N_2-atmosphere over 4 Å molecular sieves. NMR spectra were recorded at 400 MHz on a Bruker DPX-400 spectrometer.

1.2 ^{57}Fe Mössbauer Spectroscopy – All samples were prepared in an N_2 atmosphere dry glovebox equipped with a liquid N_2 fill port to enable sample freezing to 77 K. Each sample was loaded into a Mössbauer sample cup manufactured from Delrin and stored, handled, and loaded under liquid N_2. Low-temperature ^{57}Fe Mössbauer measurements were performed using a See Co. MS4 Mössbauer spectrometer integrated with a Janis SVT-400T He/N_2 cryostat for measurements at 80 K. Isomer shifts were determined relative to α-Fe at 298 K. All Mössbauer spectra were fit using the program WMoss (SeeCo). Errors of the fit analyses were the following: $\delta \pm 0.02$ mm/s and $\Delta E_Q \pm 3\%$. For multicomponent fits, the quantitation errors of individual components were ± 3%.

1.3 Electron Paramagnetic Resonance (EPR) Spectroscopy – All samples for EPR spectroscopy were prepared in an N_2 atmosphere glove box equipped with a liquid N_2 fill port to enable sample freezing to 77 K. EPR samples were prepared in 4 mm OD suprasil quartz EPR tubes from Wilmad Labglass. X-band EPR spectra were recorded on a Bruker EMXplus spectrometer equipped with a 4119HS cavity and an Oxford ESR-900 helium flow cryostat for measurements at 10 K. The instrumental parameters employed for all samples were as follows: 1 mW power; modulation amplitude 8 Gauss; frequency ≈ 9.38199 GHz; modulation frequency 100 kHz. Simulation of the EPR spectra was performed using EasySpin.[1]

1.4 Cyclic Voltammetry – Cyclic voltammetry (CV) was carried out in the glovebox under inert conditions with EMStat4s. Electrodes: Working – glassy carbon. Counter – platinum wire. Pseudo reference: Silver wire. Reference – Ag/AgCl (leakproof). Electrolyte - [NBu_4][PF_6]. Solvent – MeCN or DCM.

1.5 UV-Vis-NIR Spectroscopy – Spectra were collected using a Cary 6000i UV-Vis-NIR spectrometer fitted with a Unisoku cryostat in an air-tight 1 cm quartz cuvettes. Samples for TA spectroscopy measurements were measured before and after experiment in 1 or 2 mm air-tight quartz cuvettes.

1.6 Transient absorption (TA) spectroscopy – For transient absorption measurements, an 800 nm 1-kHz femtosecond laser system (Spectra Physics Spitfire Pro) was used to generate both broadband probe and actinic pump pulses. The broadband probe pulse was generated by focusing a portion of the 800 nm fundamental through either a sapphire or calcium fluoride crystal. The sapphire continuum provided a probe from approximately 480 to 1000 nm and the calcium fluoride provided a probe from approximately 320 nm to 900 nm. The reader will notice increased noise at the edges of the probe spectrum, where there were limited detector counts. When using a calcium fluoride crystal to generate a broadband probe, the crystal was translated continuously to ensure a stable continuum generation. The 800 nm actinic pump was used directly from the output of the amplifier with no further compression. A 600 nm actinic pump was made by using a home-build non-collinear optical parametric amplifier (NOPA) and compressed using a prism compressor. A 400 nm pump was made by frequency doubling the 800 nm fundamental using a BBO crystal; the 400 nm pump was used without further compression. Pump pulse energy was varied from 0 to 200 nJ/pulse, to confirm the kinetics were independent of pump power and the pump beam was

focused to a 40-60 micron beam diameter at the sample. Every other pump pulse was blocked using an optical chopper while every probe pulse was dispersed using a monochromator (Acton, 300 mm fl, 150 gr/mm) and collected onto a CCD (Princeton Instruments, Pixis 100BR). Samples were contained in 1 or 2 mm air-tight quartz cuvettes and translated continuously to prevent photodamage. Transient absorption scans were collected by averaging 1000 pump-on:pump-off spectra per time point using LabView (National Instruments) and data processing was performed in Igor Pro 8 (Wavemetrics). Additionally, UV-visible absorption spectra were collected of each sample before and after irradiation, and no photodamage to the sample was observed.

1.7 Single crystal X-ray diffraction (SC-XRD) – A crystal was placed onto a thin glass optical fiber or a nylon loop and mounted on a Rigaku XtaLAB Synergy-S Dualflex diffractometer equipped with a HyPix-6000HE HPC area detector for data collection at specified temperature for all cases except $[FeMes_3]^-[MgBr(THF)_5]^+$. A preliminary set of cell constants and an orientation matrix were calculated from a small sampling of reflections.[2] A short pre-experiment was run, from which an optimal data collection strategy was determined. The full data collection was carried out using a PhotonJet (Cu) X-ray source. The structure was solved using SHELXT[3] and refined using SHELXL.[4] The space group was determined based on systematic absences and intensity statistics. Most or all non-hydrogen atoms were assigned from the solution. Full-matrix least squares/difference Fourier cycles were performed which located any remaining non-hydrogen atoms. All non-hydrogen atoms were refined with anisotropic displacement parameters. All hydrogen atoms were placed in ideal positions and refined as riding atoms with relative isotropic displacement parameters. Structure manipulation and figure generation were performed using Olex2.[5] Single-crystal X-ray diffraction data of $[FeMes_3]^-[MgBr(THF)_5]^+$ was collected on an Oxford Diffraction/Agilent SuperNova diffractometer equipped with a 135 mm Atlas CCD area detector. The crystal was selected under a nitrogen atmosphere, mounted on a MicroMesh sample pin and cooled using an Oxford Cryosystems open flow N2 cooling device. Data was collected at 150 K using mirror monochromated Cu K(α) radiation (wavelength = 1.5418 Å; Oxford Diffraction Supernova). Data was then processed using CrysAlisPro package,[6] structures solved ab initio from the integrated intensities using SHELXT[3] and refined using SHELXL[4] with the graphical interface OLEX2.[5]

1.8 Density functional theory (DFT) calculations – DFT calculations were carried out using Gaussian 16 (Rev.A03)[7] for structure optimizations, frequency and TDDFT calculations, while ORCA software (v 4.2.1)[8], was used for calculation of ^{57}Fe Mössbauer parameters. Optimized structures were calculated using B3LYP[9-12] functional along with atom-pairwise dispersion correction with the Becke-Johnson damping, D3BJ[13] and def2-TZVP[14] basis set on all atoms. Experimentally obtained crystal structures were used as starting point for geometry optimizations. Charged complexes were optimized without counter-ion. To account for solvent effects PCM[15-17] solvent model was used. Frequency calculations were performed to ensure that the stationary points were true energy minima. TDDFT calculations were performed utilizing hybrid TPSSh[18, 19] functional, considering contributions from lowest 50 excited states. Contributions to the excited state transitions were calculated using Multiwfn[20] software. The ^{57}Fe Mössbauer parameters were calculated using core polarized CP(PPP)[21] basis set for Fe and def2-TZVP for all other atoms. Scalar relativistic effects were included through second-order Douglas–Kroll–Hess approximation.[22] Isomer shifts were calculated from electron densities at the Fe nucleus using a previously reported procedure.[23, 24]

1.9 Synthesis of Complexes

1.9.1 Synthesis of $FeMes_3^-$ - $FeMes_3^-$ was prepared using a modified preparation based upon previously reported syntheses of a similar Fe(II) homoleptic ferrate species.[25] A 14 mL scintillation vial was charged with $FeBr_2$ (17 mg, 0.078 mmol) and THF (4 mL) Mesityl magnesium bromide solution (0.50 mL, 1M in THF, 0.50 mmol) was added dropwise. The solution was stirred at room temperature for 5 minutes, then 2 mL of Et_2O was added. The solution was filtered over celite and stored in the glovebox freezer at -30 °C. After two days, colourless needle-shaped crystals suitable for SC-XRD were formed (33 mg, 48% yield). **^{57}Fe Mössbauer parameters (solid):** δ = 0.21 mm/s, $|\Delta E_Q|$ = 1.43 mm/s. **^{1}H NMR (400 MHz, MeCN-*d₃*):** δ= 127.60 (s, 6H, *meta*-CH), 111.67 (s, 9H, *para*-CH_3), 23.31 (s, 18H, *ortho*-CH_3), 3.62(s, THF), 1.77 (s,THF).

1.9.2 Preparation of Tris-cyclometalated Fe(III) complexes

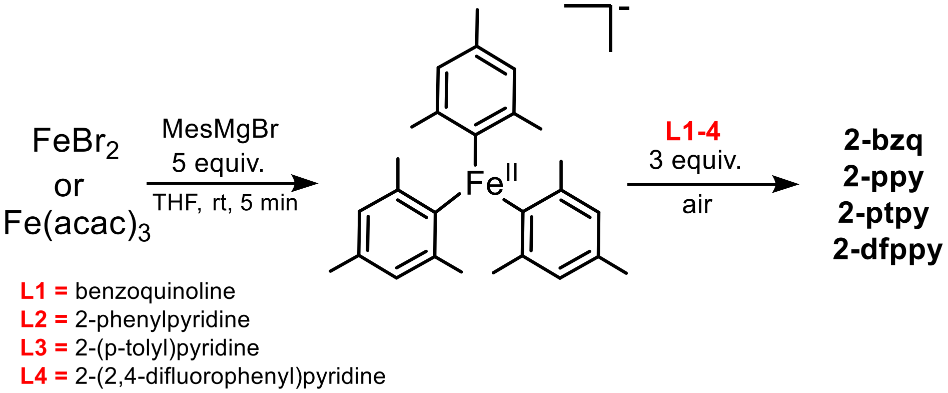

Scheme 1. General method for the preparation of tris-cyclometalated Fe(III) complexes using either $FeBr_2$ or $Fe(acac)_3$.

1.9.2.1 General Method A - A 14 mL scintillation vial fitted with a teflon stir bar was charged with $Fe(acac)_3$ (132.0 mg, 0.372 mmol), **L1-4** (1.120 mmol), and 4 mL of THF. After stirring the mixture at RT for 10 min, MesMgBr (2240 µL, 1.0 M in THF, 2.24 mmol) was added dropwise to the reaction mixture at RT. The resulting pale yellow solution was stirred for 1 h at RT. The vial was removed from the glovebox and exposed to dry air. The reaction mixture was left in the glovebox for 2 days, yielding crystals suitable for SC-XRD. Formed solids were washed with MeCN (**2-bzq**) or MeOH (**2-ppy**, **2-ptpy**, **2-dfppy**) prior to further use.

1.9.2.2 General Method B - A 14 mL scintillation vial fitted with a Teflon stir bar was charged with $FeBr_2$ (30 mg, 0.14 mmol) and THF (4 mL). Mesityl magnesium bromide solution (0.43 mL, 1 M in THF, 0.43 mmol) was added dropwise. Benzoquinoline (75 mg, 0.43 mmol) was added. The solution was heated to 60 °C and stirred for 12 hours. The vial was removed from the glovebox and exposed to air. The vial was returned to the glovebox and allowed to stir for a further 2 hours at 60 °C. The precipitate was collected via suction filtration and washed with MeCN (**2-bzq**) or chilled MeOH (**2-ppy, 2-ptpy, 2-dfppy**). The solid was used without any further purification.

1.9.2.3 Fe(III) Complex Characterisation

2-bzq – **Method A:** Dark needle shaped crystals, 25% yield. **Method B:** Dark blue powder, 85% yield. **^{57}Fe Mössbauer parameters** (solid sample): δ = 0.04, $|\Delta E_Q|$ = 1.95 mm/s. **EPR parameters** (solid sample): $g_{||}$ = 2.47, $g_{\perp}$ = 1.84. **UV-Vis-NIR** (DCM): λ (ε) = 602 (4610), 549 (4060), 499 (3300), 431 (2650), 360 (10170), 347 (9930) nm (M^{-1} cm^{-1}).

2-ppy – **Method A:** dark violet crystals, 48% yield. **Method B:** dark violet powder, 28% yield. **^{57}Fe Mössbauer parameters** (solid sample): δ = -0.01, $|\Delta E_Q|$ = 1.82 mm/s. **EPR parameters** (solid sample): $g_{||}$ = 2.42, $g_{\perp}$ = 1.90. **UV-Vis-NIR** (DCM): λ (ε) = 578 (4660), 487 (3050), 370 (3880) nm (M^{-1} cm^{-1}); (MeCN): λ (ε) = 568 (4100), 476 (2700), 366 (3670) nm (M^{-1} cm^{-1}).

2-ptpy – **Method A:** dark violet crystals, < 10% yield. **Method B:** violet powder, 39% yield. Note: the use of $FeBr_2$ in method B resulted in the desired product **2-ppy** forming, however the resulting product could not be isolated. Instead, $Fe(acac)_3$ was used and the reaction mixture was cooled to -60 °C to precipitate the product. **^{57}Fe Mössbauer parameters** (solid sample): δ = 0.01, $|\Delta E_Q|$ = 1.82 mm/s. **EPR parameters** (solid sample): $g_{||}$ = 2.46, $g_{\perp}$ = 1.87. **UV-Vis-NIR** (MeCN): λ (ε) = 566 (4450), 502 (3580) nm (M^{-1} cm^{-1}).

2-dfppy – **Method A:** orange crystals, < 10% yield. **Method B:** orange powder, 32% yield. **^{57}Fe Mössbauer parameters** (solid sample): δ = -0.01, $|\Delta E_Q|$ = 1.83 mm/s. **EPR parameters** (solid sample): $g_{||}$ = 2.46, $g_{\perp}$ = 2.00. **UV-Vis-NIR** (MeCN): λ (ε) = 515 (3600), 476 (4350) nm (M^{-1} cm^{-1}).

1.9.3 Preparation of Tris-cyclometalated Fe(II) complexes

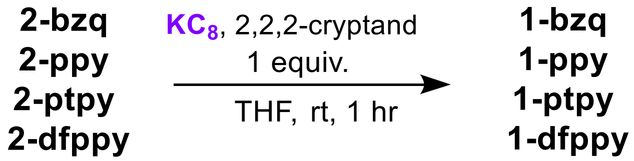

Scheme 2. General method for the preparation of tris-cyclometalated Fe(II) complexes.

1.9.3.1 General Method - To a 14 mL scintillation vial fitted with a teflon stir bar was added **2-ppy**, **2-ptpy** or **2-dfppy** (0.052 mmol), potassium graphite (7.1 mg, 0.052 mmol), 4,7,13,16,21,24-Hexaoxa-1,10-diazabicyclo[8.8.8]hexacosane (2.2.2 cryptand, 19.6 mg, 0.052 mmol) and 4 mL of THF (**2-ppy**, **2-ptpy**) or 4 mL of DME (**2-dfppy**). After stirring mixture at RT for 1 h, solution was filtrated through Celite and 4 mL of Et_2O were added prior storing solution at -30 °C. Crystals suitable for SC-XRD were observed after 1 day. Crystals were collected via suction filtration and washed with Et_2O (3 x 1 mL).

1.9.3.2 Synthesis of 1-bzq– A 14 mL scintillation vial was charged with a teflon stir bar, $Fe(bzq)_3$ (15 mg, 0.025 mmol), potassium graphite (3.4 mg, 0.025 mmol), 4,7,13,16,21,24-Hexaoxa-1,10-diazabicyclo[8.8.8]hexacosane (2.2.2-cryptand, 9.4 mg, 0.025 mmol) and MeCN (4 mL). After stirring mixture at RT for 1 h, solution was filtered through Celite and 8 mL of Et_2O was added. The precipitate was collected via suction filtration and washed with Et_2O (3 x 2 mL) (54 % yield). To grow crystals suitable for SC-XRD, the reaction mixture post filtration (0.5 mL) was set up for vapor diffusion with Et_2O (1 mL). After two days stored at room temperature in the glovebox, crystals suitable for SC-XRD were formed.

1.9.3.3 Fe(II) Complex Characterisation

1-bzq – Dark green crystals, 54% yield. **^{57}Fe Mössbauer parameters** (solid sample): δ = 0.06, $|\Delta E_Q|$ = 0.65 mm/s. **UV-Vis-NIR** (**MeCN**): λ (ε) = 918 (12180), 696 (10230), 628 (9000), 535 (5440), 476 (5780), 436 (5870), 356 (26310) nm (M^{-1} cm^{-1}). **^{1}H NMR (400 MHz, MeCN-*d*$_3$):** δ=7.60-7.65 (m, 9H, *j = 7 Hz,* CH), 7.49 (d, 3H, *j = 8 Hz,* CH), 6.93 (d, 3H, *j = 8 Hz,* CH), 6.80-6.86 (m, 6H, *j = 8 Hz,* CH), 6.66 (d, 3H, *j = 8 Hz,* CH), 3.58 (s, 12H, CH_2), 3.52 (t, 12H, *j = 6 Hz,* CH_2), 2.53 (t, 12H, *j = 4 Hz,* CH_2).

1-ppy – Dark crystal, 30 % yield suitable for SC-XRD were formed.**^{57}Fe Mössbauer parameters** (solid sample): δ = 0.04, $|\Delta E_Q|$ = 0.83 mm/s. **UV-Vis-NIR** (**MeCN**): λ (ε) = 900 (8100), 748 (14930), 535 (10340), 418 (10340) nm (M^{-1} cm^{-1}). **^{1}H NMR (400 MHz, MeCN-*d*$_3$):** δ = 7.56 (s, 8H, CH), 7.34 (s, 4H, CH), 6.39 (s, 4H, CH), 6.23 (s, 4H, CH), 5.86 (s, 4H, CH), 3.56-3.51 (d, 24H, *j =20 Hz*, CH_2), 2.52 (s, 12 H, CH_2).

1-ptpy - **^{57}Fe Mössbauer parameters** (solid sample): δ = 0.04, $|\Delta E_Q|$ = 0.81 mm/s. **UV-Vis-NIR** (MeCN): λ (ε) = 900 (4460), 748 (9410), 542 (7570), 425 (7150) nm (M^{-1} cm^{-1}). **^{1}H NMR (400 MHz, MeCN-*d*$_3$):** δ = 7.47-7.55 (m, 6H, *J= 8 Hz*, CH), 7.17-7.21 (t, 3H, *J=8 Hz*, CH), 6.40 (s, 3H, CH), 6.22-6.24 (d, 3H, *J= 8Hz,* CH), 6.13 (s, 6H, CH), 3.50-3.56 (m, 24H, CH_2), 2.51-2.52 (m, 12H, CH_2).

1-dfppy – **^{57}Fe Mössbauer parameters** (solid sample): δ = 0.03, $|\Delta E_Q|$ = 0.65 mm/s. **UV-Vis-NIR** (MeCN): λ (ε) = 716 (8980), 685 (9290), 569 (6510), 503 (4950), 415 (8270) nm (M^{-1} cm^{-1}). **^{1}H NMR (400 MHz, MeCN-*d*$_3$):** δ = 8.05 (s, 3H, CH), 7.36-7.26 (d, 6H, *j= 40* Hz, CH), 6.66 (s, 3H, CH), 6.02 (s, 6H, CH), 3.54 (s, 24H, CH_2), 2.51 (s, 12 H, CH_2). **^{19}F NMR (400 MHz, MeCN-*d*$_3$):** δ = -113.72, -118.39.

1.9.4 Preparation of tris-cyclometalated Fe(IV) complex

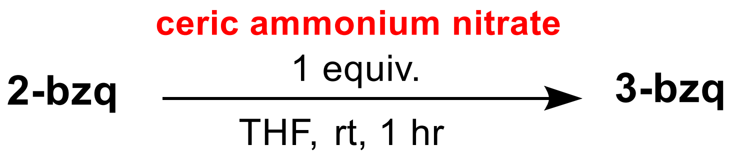

Scheme 3. General method for the preparation of tris-cyclometalated Fe(Iv) complexes

3-bzq – To a 20 mL scintillation vial fitted with a teflon stir bar was added $Fe(bzq)_3$ (0.025 mmol), ceric ammonium nitrate (13.7 mg, 0.025 mmol) and 4 mL of MeCN. After stirring the mixture at RT for 1 h, the solution was filtrated through Celite and 2 mL of Et_2O was added prior to storing the solution at -30 °C. After a day, dark green crystals (5.2 mg, 10% yield) suitable for SC-XRD were formed. **^{57}Fe Mössbauer parameters** (solid sample): δ = -0.05, $|\Delta E_Q|$ = 1.30 mm/s. **UV-Vis-NIR** (MeCN): λ (ε) = 702 (6160), 515 (2340), 403 (9100), 352 (21740) nm (M^{-1} cm^{-1}).

2. Mössbauer spectra

2.1 Solution Mössbauer Spectra

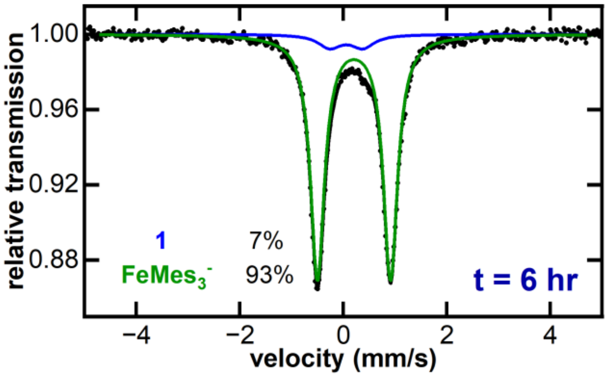

Figure S1. Zero-field, 80 K ^{57}Fe Mössbauer Spectrum of **FeMes$_3^-$** reacted with 3.1 equiv. of Hbzq at RT for 6 hours. Black dotted trace: raw data. Black solid trace: best fit. Coloured traces: simulation. **FeMes$_3^-$:** δ = 0.21, $|\Delta E_Q|$ = 1.43 mm/s. **1:** δ = 0.06, $|\Delta E_Q|$ = 0.71 mm/s.

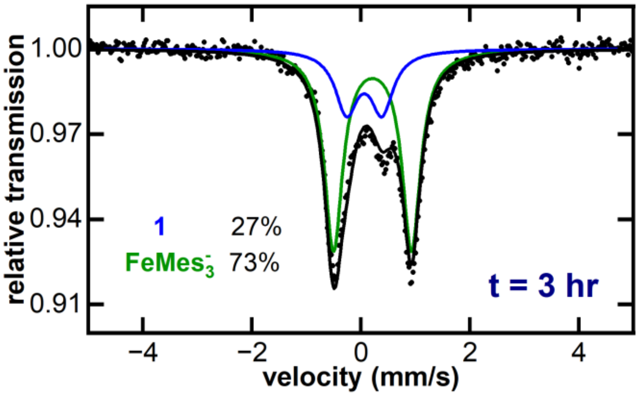

Figure S2. Zero-field, 80 K ^{57}Fe Mössbauer Spectrum of **FeMes$_3^-$** reacted with 1 equiv. of Hbzq at 60 °C for 3 hours. Black dotted trace: raw data. Black solid trace: best fit. Coloured traces: simulation. **FeMes$_3^-$**, green trace: δ = 0.21, $|\Delta EQ|$ = 1.43 mm/s. **1,** blue trace: δ = 0.06, $|\Delta EQ|$ = 0.71 mm/s.

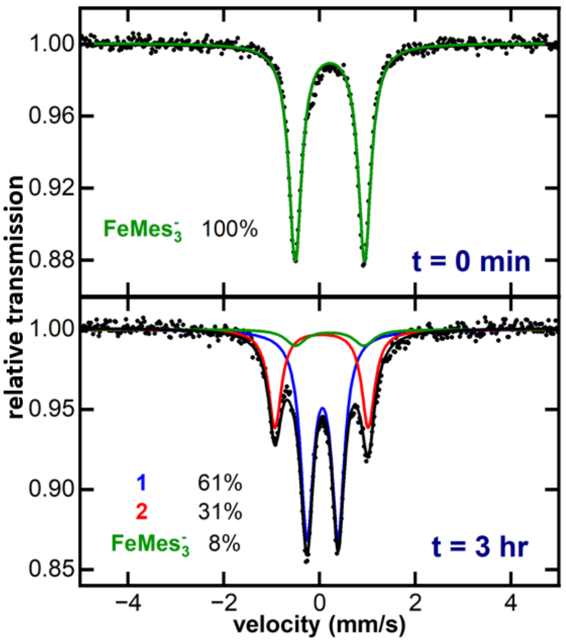

Figure S3. Zero-field, 80 K ^{57}Fe Mössbauer spectra of in situ generated **FeMes$_3^-$** reacted with 3.1 equiv. of Hbzq at 60 °C for 3 hours. Black dotted trace: raw data. Black solid trace: best fit. Coloured traces: simulation. **FeMes$_3^-$**, green trace: δ = 0.21, $|\Delta E_Q|$ = 1.43 mm/s. **1**, blue trace: δ = 0.06, $|\Delta E_Q|$ = 0.71 mm/s. **2**, red trace: δ = 0.04, $|\Delta E_Q|$ = 1.95 mm/s.

2.2 Solid Mössbauer Spectra

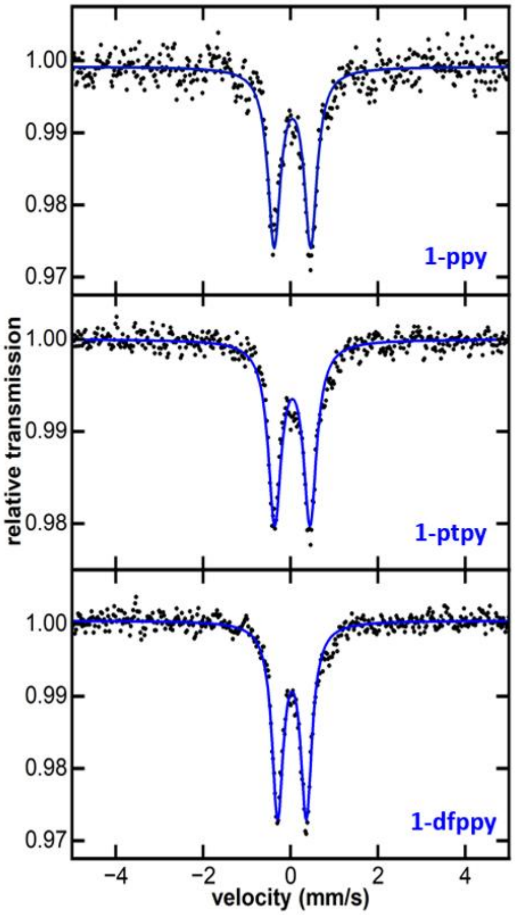

Figure S4. Zero-field, 80 K ^{57}Fe Mössbauer spectra of solid **1-ppy, 1-ptpy** and **1-dfppy**. Black dotted trace: raw data. Blue trace: simulation. **1-ppy:** δ = 0.04, $|\Delta E_Q|$ = 0.83 mm/s. **1-ptpy:** δ = 0.04, $|\Delta E_Q|$ = 0.81 mm/s **1-dfppy** δ = 0.03, $|\Delta E_Q|$ = 0.65 mm/s.

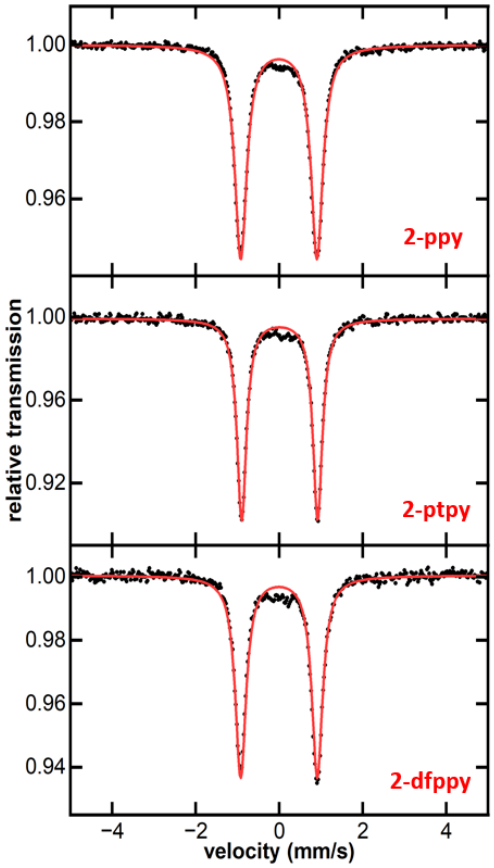

Figure S5. Zero-field, 80 K ^{57}Fe Mössbauer spectra of solid samples **2-ppy**, **2-ptpy** and **2-dfppy**. Black dotted trace: raw data. Red trace: simulation. **2-ppy:** δ = -0.01, $|\Delta E_Q|$ = 1.82 mm/s. **2-ptpy:** δ = 0.01, $|\Delta E_Q|$ = 1.82 mm/s. **2-dfppy:** δ = -0.01, $|\Delta E_Q|$ = 1.83 mm/s.

3. EPR spectra

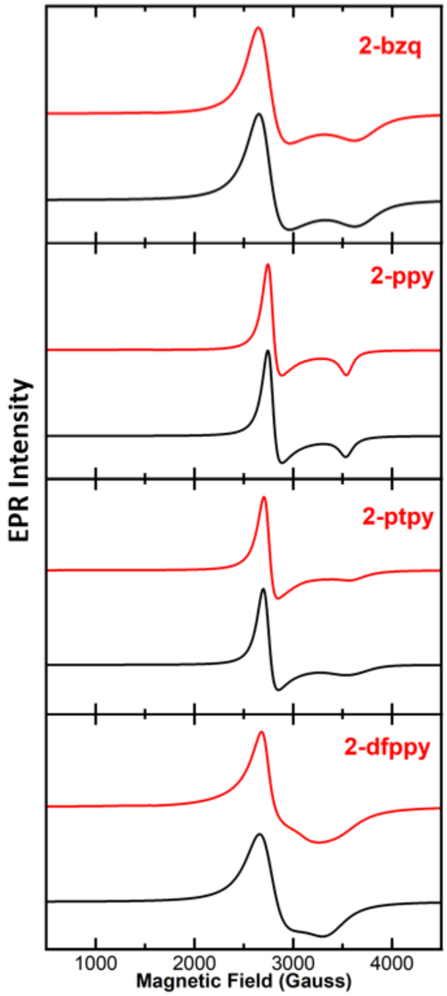

Figure S6. 10 K EPR spectra of solid **2-bzq**, **2-ppy**, **2-ptpy** and **2-dfppy**. Black trace: experimental data. Red trace: corresponding simulations.

4. Cyclic Voltammograms

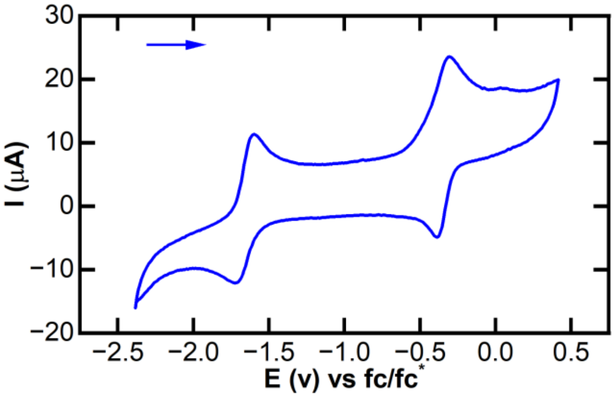

Figure S7. Cyclic voltammogram of **1-bzq** in 0.3 M $[NBu_4][PF_6]$ in MeCN at 298 K, scan rate = 100 mV s^{-1}.

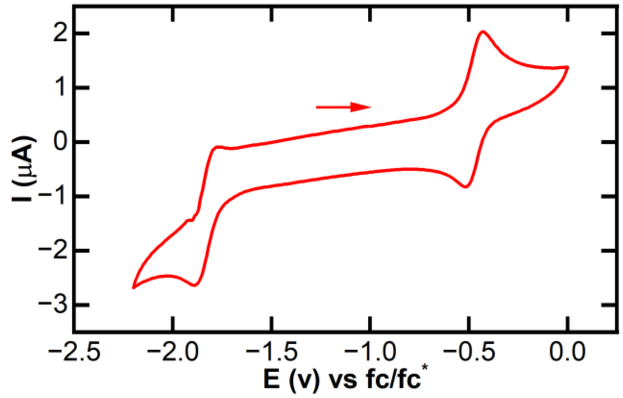

Figure S8. Cyclic voltammogram of **2-bzq** in 0.1 M $[NBu_4][PF_6]$ in DCM at 298 K, scan rate = 25 mV s^{-1}.

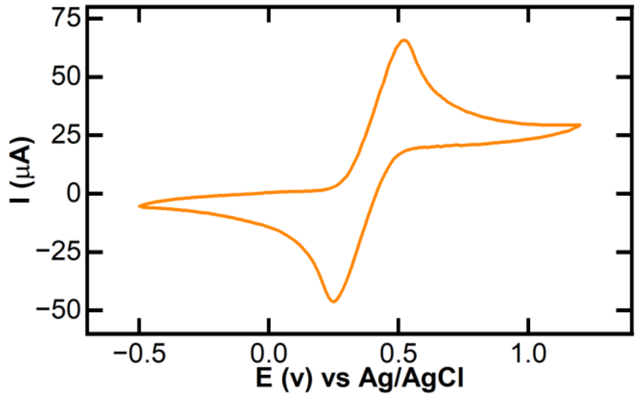

Figure S9. Cyclic voltammogram of ferrocene in 0.3 M $[NBu_4][PF_6]$ in MeCN at 298 K, scan rate = 100 mV s^{-1}.

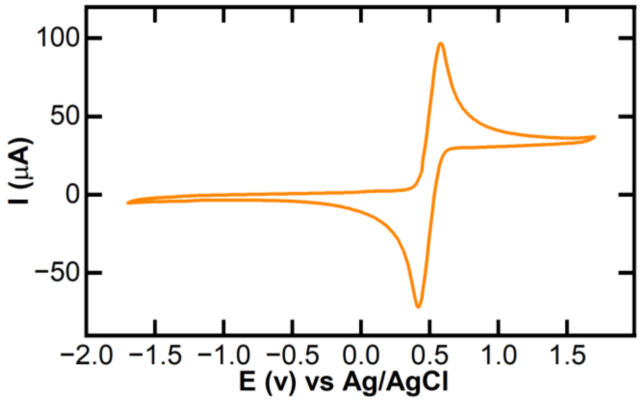

Figure S10. Cyclic voltammogram of ferrocene in 0.3 M $[NBu_4][PF_6]$ in DCM at 298 K, scan rate = 100 mV s^{-1}.

5. UV-VIS Spectra

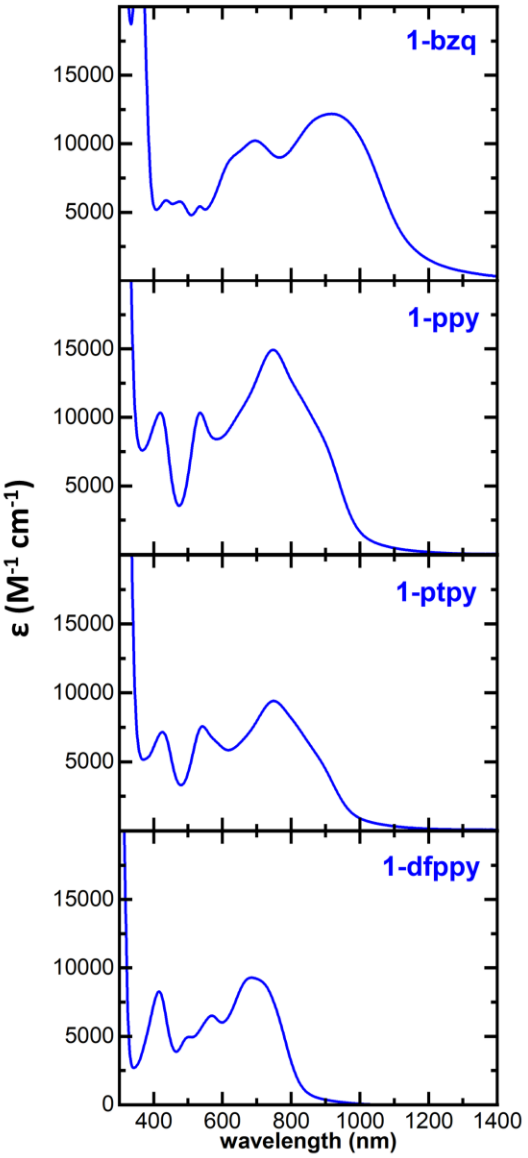

Figure S11. Electronic absorption spectra of complexes **1-bzq, 1-ppy**, **1-ptpy** and **1-dfppy** in MeCN.

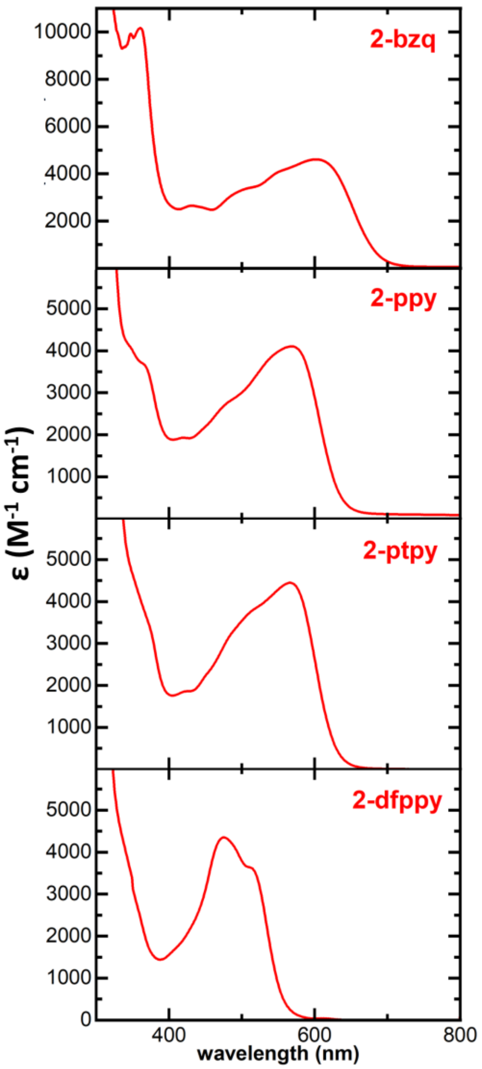

Figure S12. Electronic absorption spectra of complexes **2-bzq** in DCM and **2-ppy**, **2-ptpy** and **2-dfppy** in MeCN.

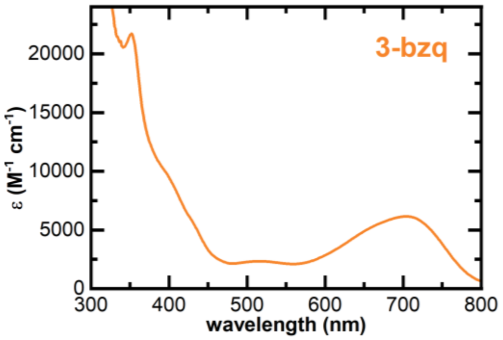

Figure S13. Electronic absorption spectra of complex **3-bzq** in MeCN.

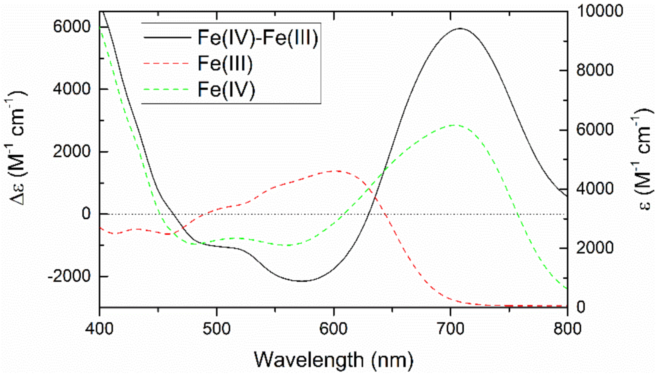

Figure S14. Electronic absorption difference spectrum of Fe(IV) and Fe(III) complexes with bzq ligand.

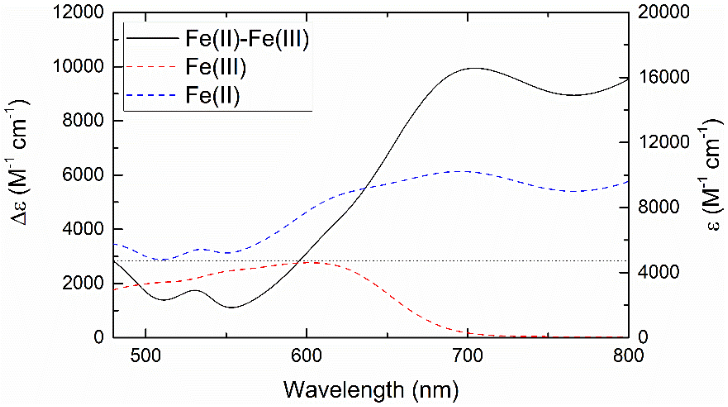

Figure S15. Electronic absorption difference spectrum of Fe(II) and Fe(III) complexes with bzq ligand.

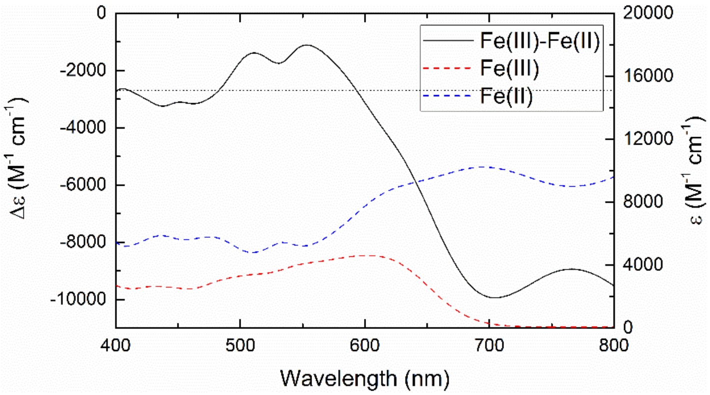

Figure S16. Electronic absorption difference spectrum of Fe(III) and Fe(II) complexes with bzq ligand.

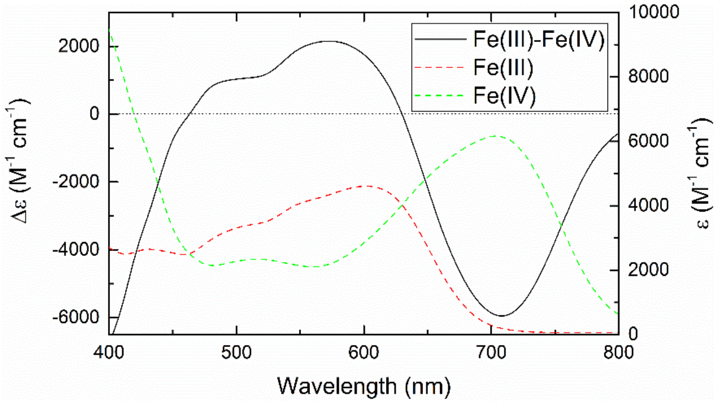

Figure S17. Electronic absorption difference spectrum of Fe(III) and Fe(IV) complexes with bzq ligand.

6. NMR data

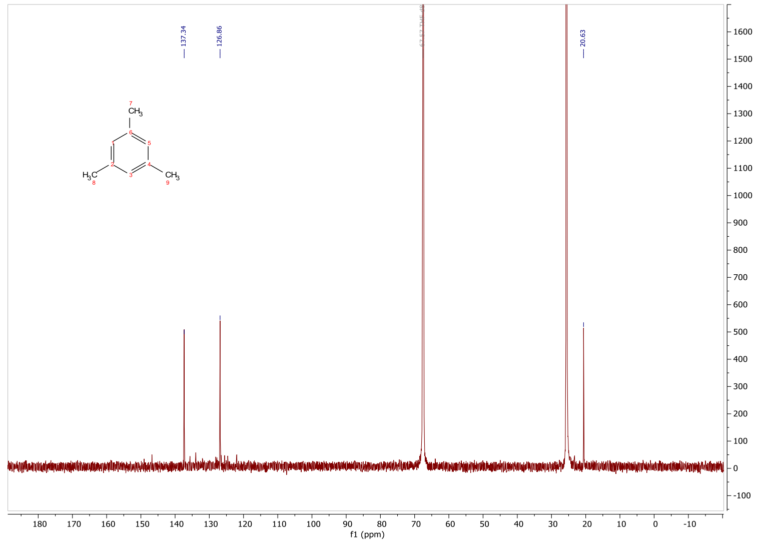

Figure S18. $^{13}C\{^{1}H\}$ NMR spectrum of crude reaction mixture in THF. $FeBr_2$ (30 mg, 0.14 mmol) was suspended in THF (4 mL). Mesityl Magnesium Bromide solution (0.43 mL, 1 M in THF, 0.43 mmol) was added dropwise. Hbzq (75 mg, 0.43 mmol) was added. The solution was heated to 60 °C for 6 hours, at which point the iron precipitated. The solution was filtered over celite and NMR was run on the crude reaction.

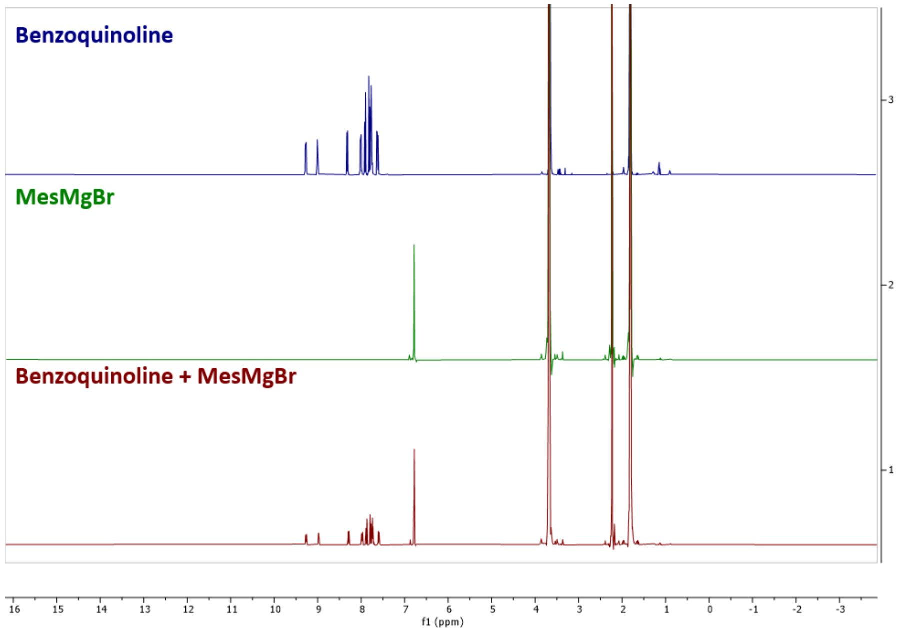

Figure S19. ^{1}H NMR of the reaction of benzoquinoline ligand (blue spectrum) with mesityl magnesium bromide (green spectrum) in MeCN-d_3 resulting in no discernable changes (red spectrum).

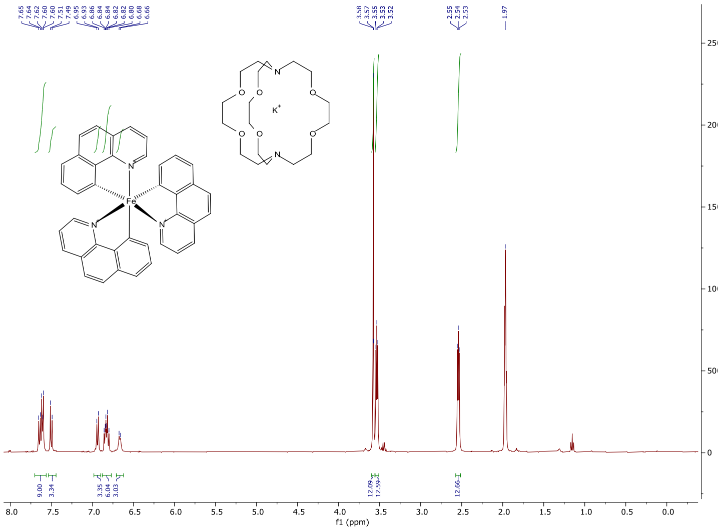

Figure S20. ^{1}H NMR spectrum of complex **1-bzq** in MeCN-d_3.

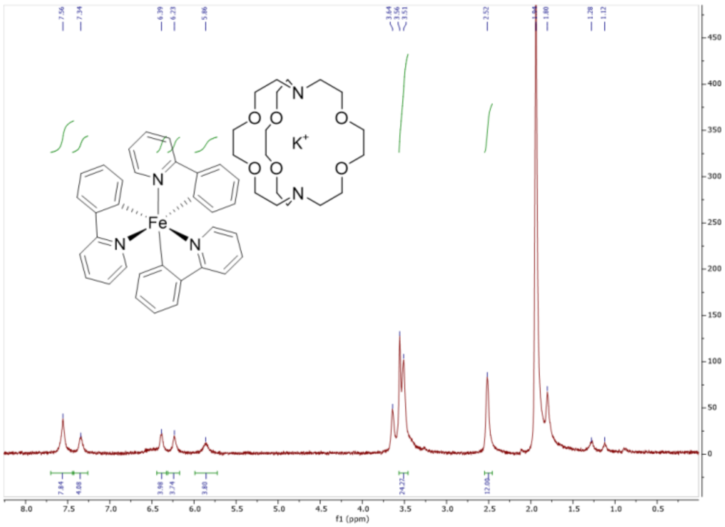

Figure S21. ^{1}H NMR spectrum of complex **1-ppy** in MeCN-d_3. Slight broadening of the signals is attributed to trace amounts of the paramagnetic Fe(III) complex **2-ppy** in solution.

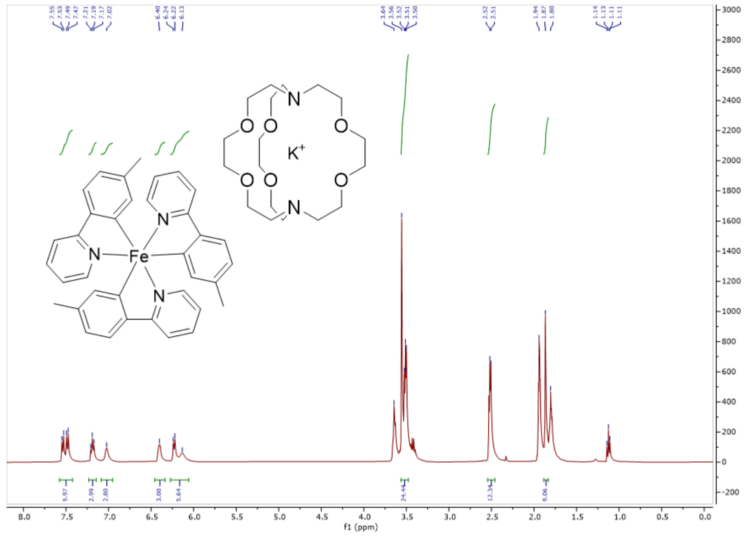

Figure S22. ^{1}H NMR spectrum of complex **1-ptpy** in MeCN-d_3.

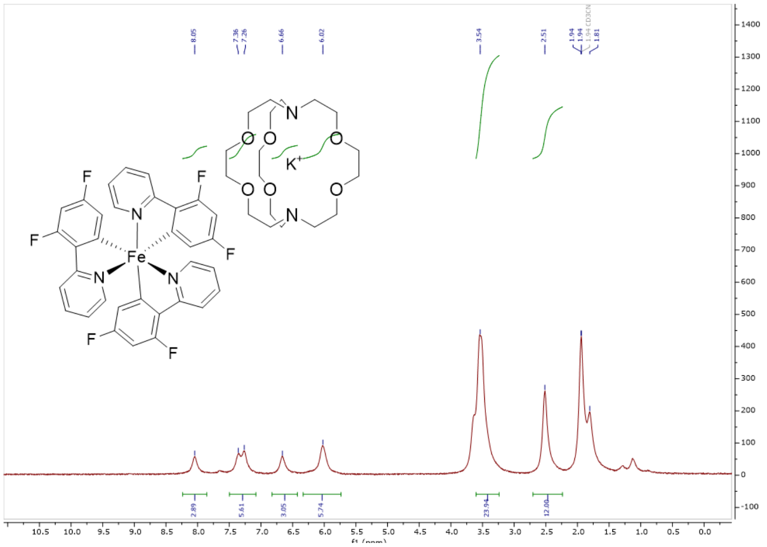

Figure S23. ^{1}H NMR spectrum of complex **1-dfppy** in MeCN-d_3. Slight broadening of the signals is attributed to trace amounts of the paramagnetic Fe(III) complex **2-dfppy** in solution.

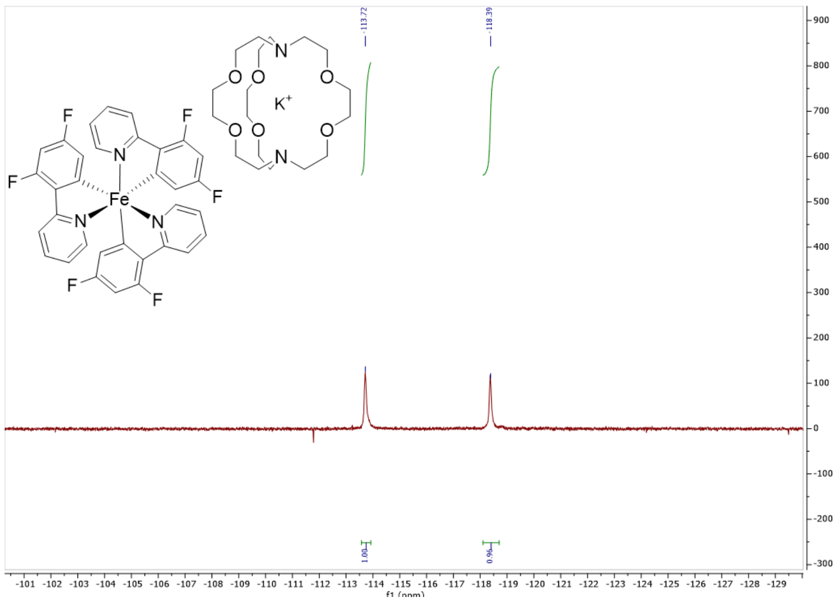

Figure S24. ^{19}F NMR spectrum of complex **1-dfppy** in MeCN-d_3.

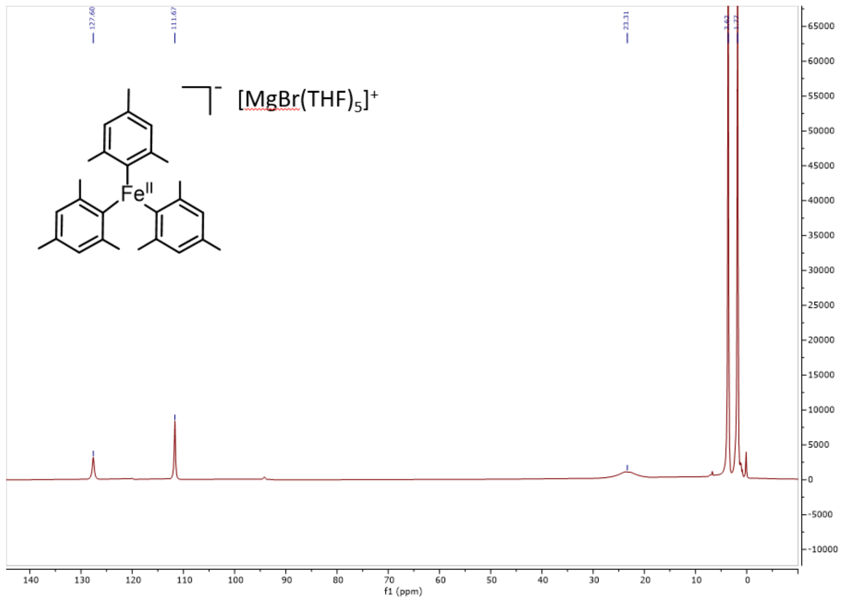

Figure S25. ^{1}H NMR spectrum of **FeMes$_3$**$^-$ ([Fe(Mes)$_3$][MgBr(THF)$_5$]) in THF-d_8.

7. Transient Absorption Spectra and Kinetics

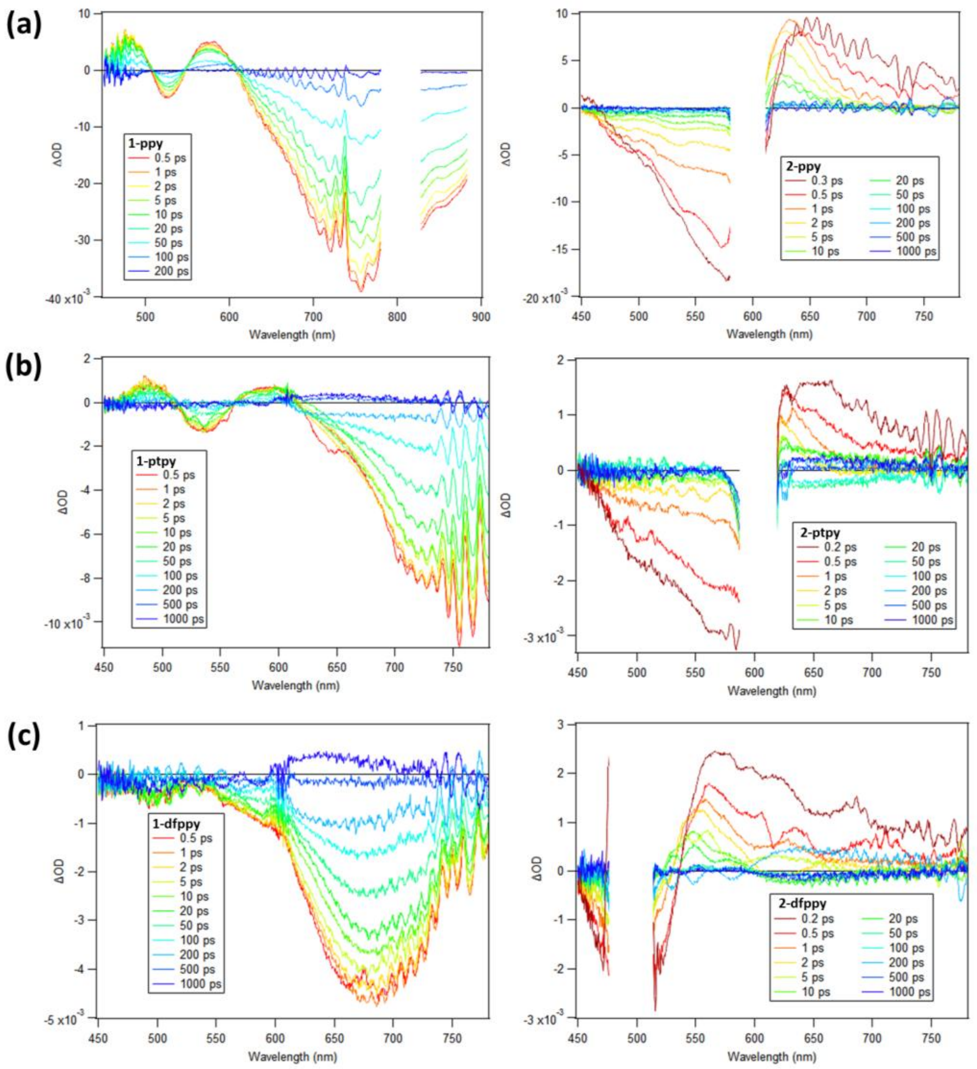

Figure S26. Transient absorption spectra of (a) **1-ppy** and **2-ppy** (b) **1-ptpy** and **2-ptpy**, and (c) **1-dfppy** and **2-dfppy.**

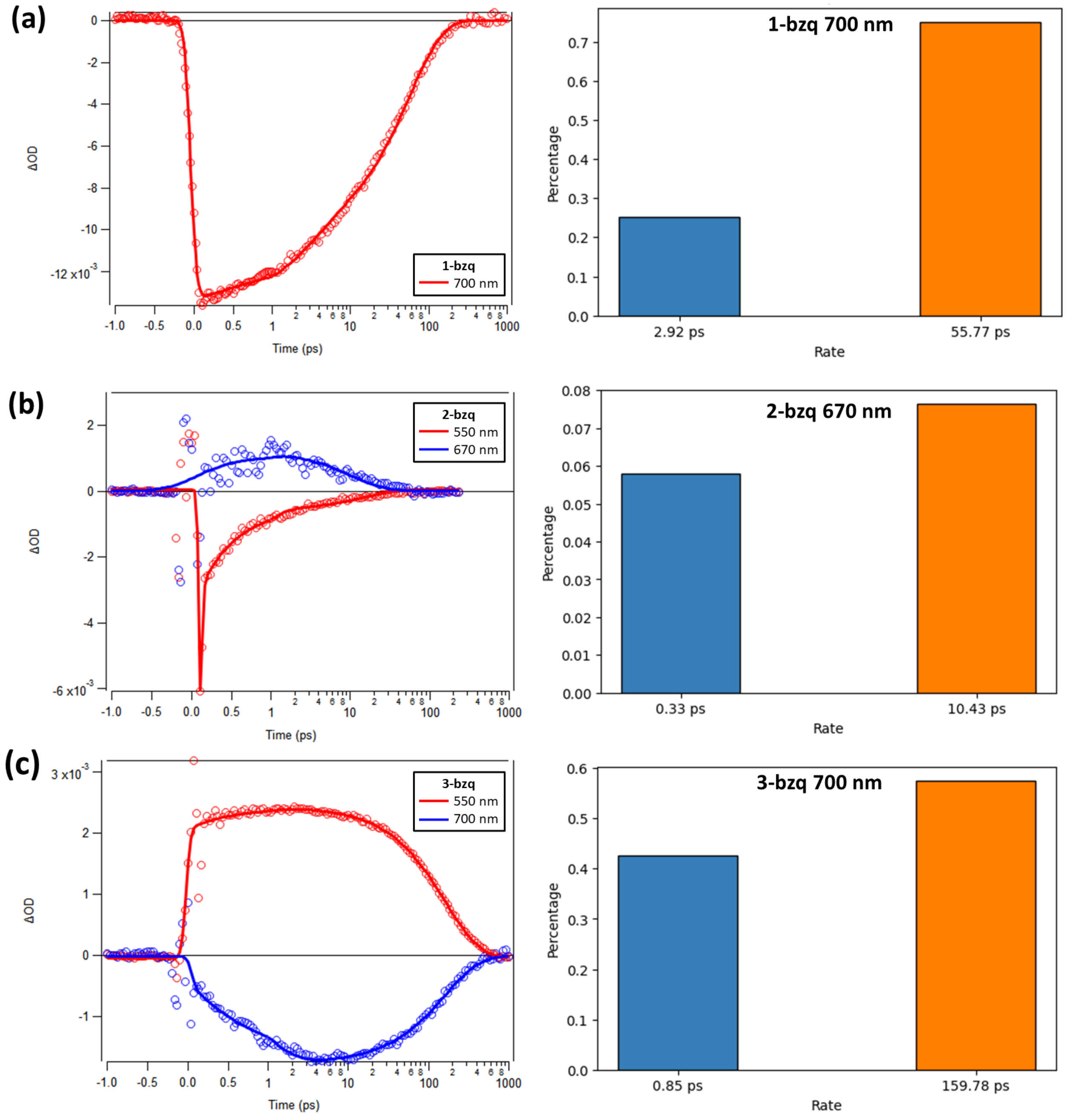

Figure S27. (left) Transient absorption kinetic traces (unfilled circles) alongside their fitted curves (solid lines), and (right) the percentages of decay versus time constants for the selected kinetic traces of **(a) 1-bzq**, **(b) 2-bzq**, and **(c) 3-bzq**. **1-bzq** exhibits a 56 ps lifetime. **2-bzq** exhibits an ultrafast relaxation to the ground state in 0.33 ps, followed by vibrational cooling in 10 ps. **3-bzq** exhibits a 160 ps excited-state lifetime.

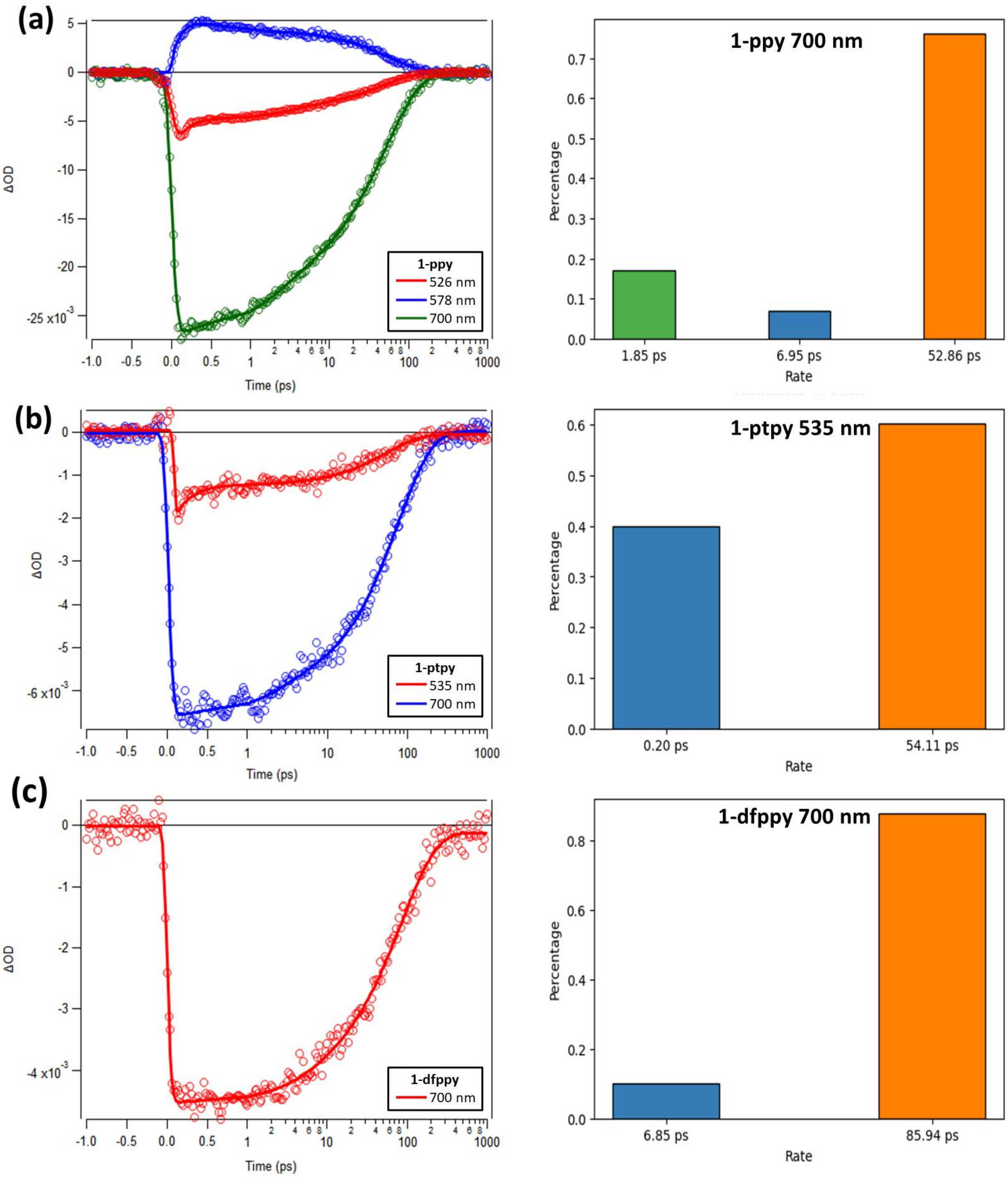

Figure S28. (left) Transient absorption kinetic traces (unfilled circles) alongside their fitted curves (solid lines), and (right) the percentages of decay versus time constants for the selected kinetic traces of (a) **1-ppy**, (b) **1-ptpy** and (c) **1-dfppy** and **1-ppy** exhibits a 47-53 ps excited-state lifetime. We interpret the kinetics of **1-ptpy** to imply a 54 ps excited state lifetime. **1-dfppy** exhibits a clear 86 ps lifetime.

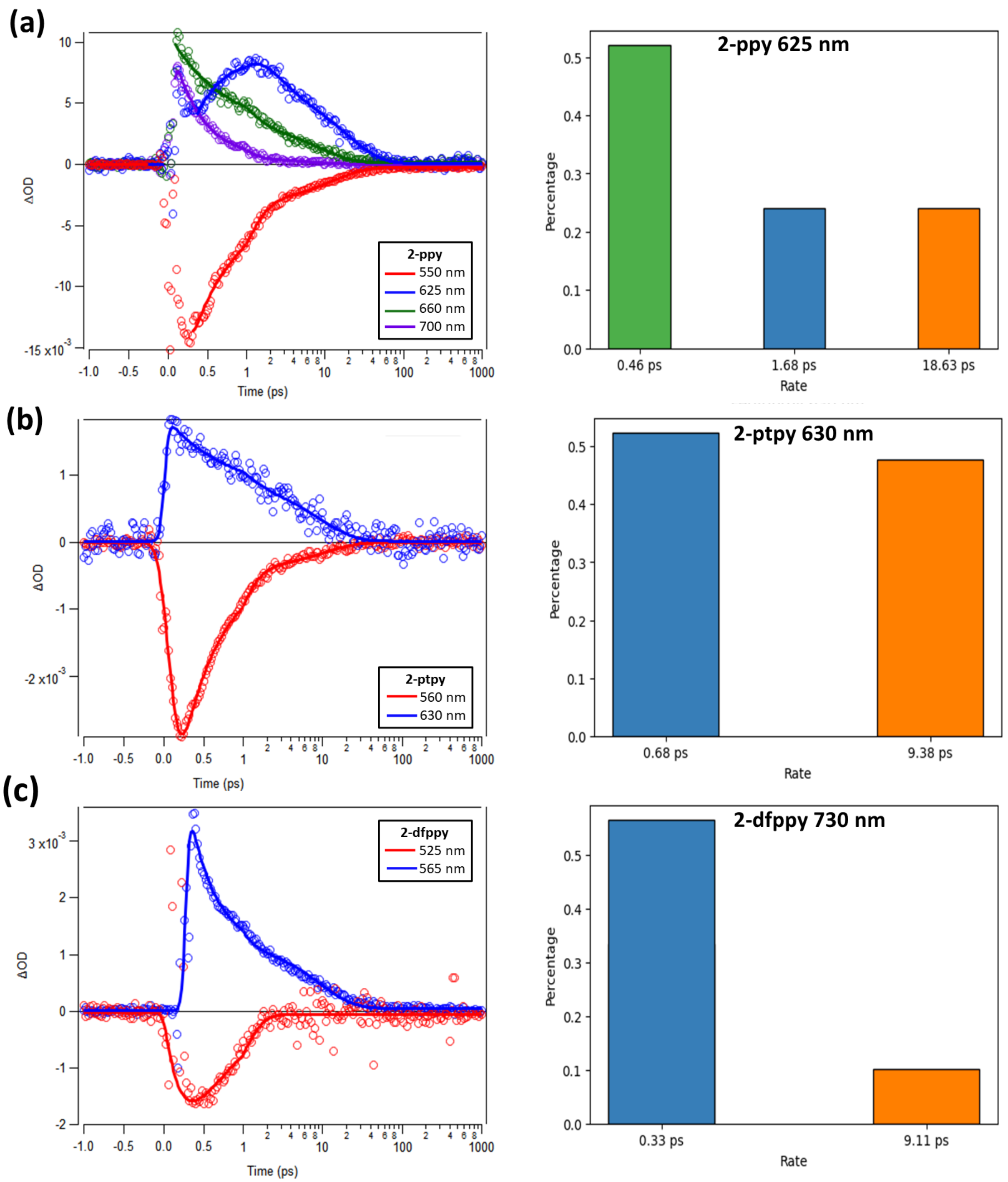

Figure S29. (left) Transient absorption kinetic traces (unfilled circles) alongside their fitted curves (solid lines), and (right) the percentages of decay versus time constants for the selected kinetic traces of (a) **2-ppy**, (b) **2-ptpy** and (c) **2-dfppy**. **2-ppy** exhibits ultrafast relaxation to the ground state in 0.5 ps, followed by vibrational cooling in 2-19 ps. **2-ptpy** exhibits a <1 ps excited state lifetime followed by vibrational cooling in 9 ps. **1-dfppy** shows complicated rapid relaxation that we interpret as 0.3 ps relaxation to the ground state followed by vibrational cooling in 9 ps.

Table S1. Multiexponential regression results for all kinetic traces.

Sample Wavelength (nm)	*t1 (ps)*	*Ampl.%*	*t2 (ps)*	*Ampl.%*	*t3 (ps)*	*Ampl.%*	*Long-term ampl.%*
1-bzq							
700	55.76	-74.9%	2.92	-25.1%	--	--	--
2-bzq							
550	0.012	-86.6%	0.45	-10.7%	14.87	-2.7%	--
670	0.016	86.6%	0.33	-5.8%	10.43	7.6%	--
3-bzq							
550	163.60	86.5%	0.546	-13.5%	--	--	--
700	159.78	-57.4%	0.847	42.6%	--	--	--
1-ppy							
526	0.13	43.4%	3.14	15.3%	47.46	41.4%	--
578	0.15	-45.0%	0.37	23.0%	55.53	32.0%	--
700	1.85	16.7%	6.95	7.3%	52.86	76.1%	--
2-ppy							
550	0.60	79.2%	12.13	19.3%	--	--	1.5%
625	0.46	-51.9%	1.68	23.5%	18.63	24.4%	0.2%
660	0.64	70.5%	8.40	28.5%	--	--	1.0%
700	0.37	94.1%	5.66	5.8%	--	--	0.1%
1-ptpy							
535	54.11	-60.2%	0.20	-39.8%	--	--	--
700	75.69	-88.6%	2.51	-11.4%	--	--	--
2-ptpy							
560	0.49	-89.6%	10.20	-10.4%	--	--	--
630	0.68	52.3%	9.38	47.7%	--	--	--
1-dfppy							
700	85.94	-87.5%	6.85	-10.0%	--	--	-2.4%
2-dfppy							
525	0.30	50.2%	0.43	-49.8%	--	--	--
565	0.33	-33.4%	0.33	56.5%	9.11	10.1%	--

8. Single Crystal X-Ray Diffraction

Table S2. Selected structural parameters of Fe(II) complexes.

**Complex**	Fe-N_{avg} (Å)	Fe-C_{avg} (Å)	N-Fe-N_{avg} (°)	C-Fe-C_{avg} (°)	trans C-Fe-N_{avg} (°)	C-Fe-N_{avg} (°)
**1-bzq**	1.9983	1.9515	91.90	93.05	173.25	82.88
**1-ppy**	1.9930	1.9540	93.08	92.56	171.99	81.68
**1-ptpy**	1.9869	1.9420	93.60	94.45	174.30	82.37
**1-dfppy**	1.9880	1.9320	93.27	94.36	174.35	82.22

Table S3. Selected structural parameters of Fe(III) complexes.

**Complex**	Fe-N_{avg} (Å)	Fe-C_{avg} (Å)	N-Fe-N_{avg} (°)	C-Fe-C_{avg} (°)	trans C-Fe-N_{avg} (°)	C-Fe-N_{avg} (°)
**2-bzq**	2.0511	1.9545	90.88	94.98	173.02	82.62
**2-ppy**	2.0090	1.9780	95.62	93.76	174.76	81.68
**2-ptpy**	2.0357	1.9529	93.80	93.55	173.98	82.15
**2-dfppy**	2.0252	1.9500	94.38	93.88	174.93	82.04
**2-mppy**	2.0360	1.9436	94.18	94.84	173.44	80.85
**2-dfpfpy**	2.0223	1.9490	94.48	93.74	174.49	82.03

Table S4. Selected structural parameters of Fe(IV) complex.

**Complex**	Fe-N_{avg} (Å)	Fe-C_{avg} (Å)	N-Fe-N_{avg} (°)	C-Fe-C_{avg} (°)	trans C-Fe-N_{avg} (°)	C-Fe-N_{avg} (°)
**3-bzq**	2.051	1.972	92.30	90.44	173.56	84.30

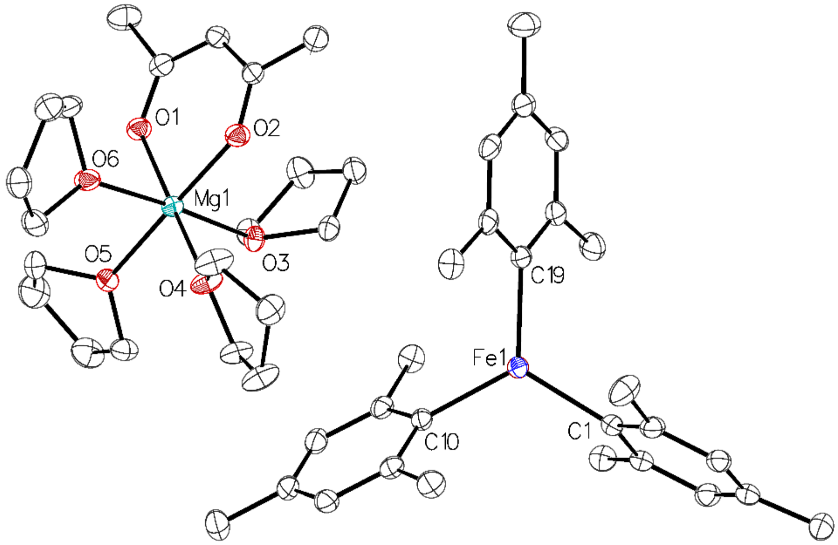

Figure S30. Crystal structure of **[Fe(Mes)$_3$][Mg(acac)(THF)$_4$]**. Hydrogen atoms are omitted for clarity and the thermal ellipsoids are shown at 50% probability. Crystal was isolated from a reaction mixture of **2-bzq** method A.

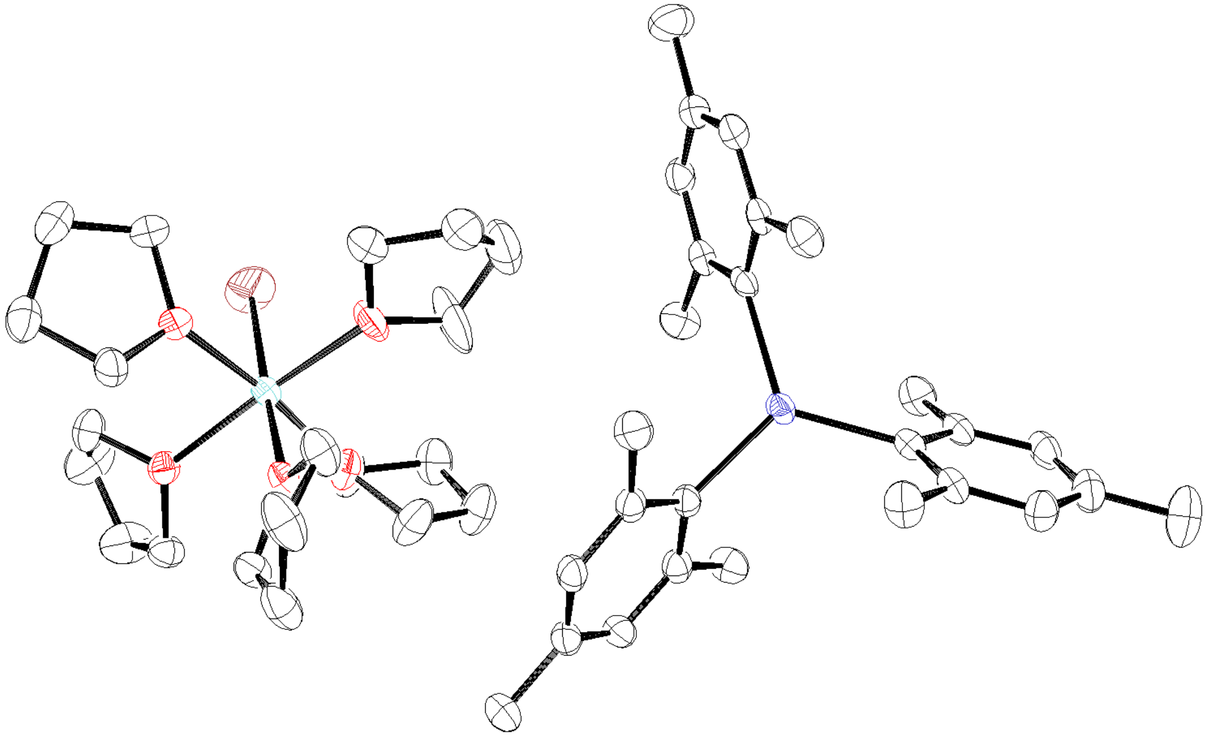

Figure S31. Crystal structure of **[Fe(Mes)$_3$][MgBr(THF)$_5$]**. Hydrogen atoms are omitted for clarity and the thermal ellipsoids are shown at 50% probability.

Table S5. Crystal data and structure refinement of **[Fe(Mes)$_3$][Mg(acac)(THF)$_4$]** and **[Fe(Mes)$_3$][MgBr(THF)$_5$]**.

	**[Fe(Mes)$_3$][Mg(acac)(THF)$_4$]**	**[Fe(Mes)$_3$][MgBr(THF)$_5$]**.
Empirical formula	C48 H72 Fe Mg O6	C47 H73 Br Fe Mg O5
Formula weight	825.21 g mol^{-1}	878.13 g mol^{-1}
Temperature	100.00(10) K	150 K
Wavelength	1.54184 Å	1.54184 Å
Crystal system	orthorhombic	monoclinic
Space group	$P2_12_12_1$	
Unit cell dimensions	*a* = 12.38100(10) Å α = 90°	*a* = 12.4231Å α = 90°
	b = 15.93360(10) Å β = 90°	*b* = 17.6759 Å β = 95.467°
	c = 23.8962(2) Å γ = 90°	*c* = 21.3412 Å γ = 90°
Volume	4714.10(6) Å^3	4665.0 Å^3
Z	4	4
Density (calculated)	1.163 g cm^{-3}	1.250 g cm^{-3}
Absorption coefficient	3.037 mm^{-1}	4.047 mm^{-1}
F(000)	1784	1872
Crystal color, morphology	colourless, block	Colourless, needle
Crystal size	0.357 x 0.218 x 0.16 mm^3	0.38 x 0.12 x 0.07 mm^3
Theta range for data collection	3.334 to 78.369°	7.922 to 154.006°
Index ranges	$-15 \le h \le 12$, $-20 \le k \le 20$, $-30 \le l \le 29$	$-15 \le h \le 14$, $-21 \le k \le 22$, $-24 \le l \le 26$
Reflections collected	38695	41369
Independent reflections	9906 [*R*(int) = 0.0396]	9585 [*R*(int) = 0.0925]
Observed reflections	9541	6868
Completeness to theta = 74.504°	100.0%	97.1%
Absorption correction	Multi-scan	Gaussian
Max. and min. transmission	1.00000 and 0.33973	0.295 and 1.000
Refinement method	Full-matrix least-squares on F^2	Full-matrix least-squares on F^2
Data / restraints / parameters	9906 / 0 / 516	9585 / 0 / 505
Goodness-of-fit on F^2	1.051	1.025
Final *R* indices [*I*>2sigma(*I*)]	*R*1 = 0.0301, *wR*2 = 0.0782	*R*1 = 0.0593, *wR*2 = 0.1344
R indices (all data)	*R*1 = 0.0314, *wR*2 = 0.0790	*R*1 = 0.0858, *wR*2 = 0.1486
Absolute structure parameter	-0.0091(12)	
Largest diff. peak and hole	0.407 and -0.294 e.Å^{-3}	0.77 and -1.03 e.Å^{-3}

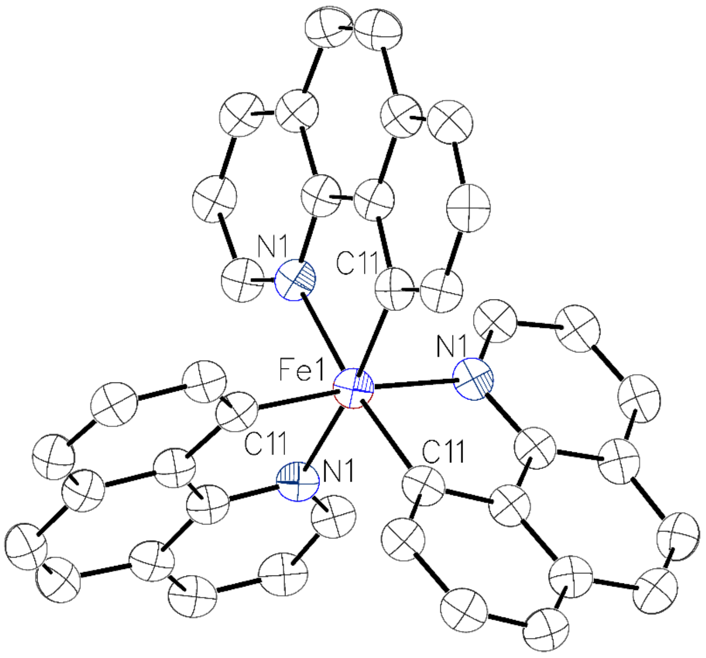

Figure S32. Crystal structure of **2-bzq**. Hydrogen atoms are omitted for clarity and the thermal ellipsoids are shown at 50% probability.

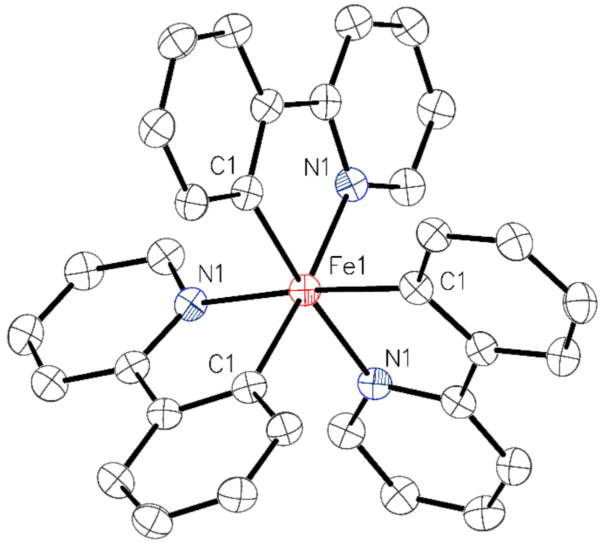

Figure S33. Crystal structure of **2-ppy**. Hydrogen atoms are omitted for clarity and the thermal ellipsoids are shown at 50% probability.

Table S6. Crystal data and structure refinement of **2-bzq** and **2-ppy**.

	**2-bzq**	**2-ppy**
Empirical formula	C39 H24 Fe N3	C33 H24 Fe N3
Formula weight	590.46	518.40
Temperature	100.00(10) K	100.01(10) K
Wavelength	1.54184 Å	1.54184 Å
Crystal system	trigonal	trigonal
Space group	*P*-3*c*1	*P*-3*c*1
Unit cell dimensions	*a* = 15.0670(6) Å α= 90°	*a* = 16.5115(2) Å α = 90°
	b = 15.0670(6) Å β = 90°	*b* = 16.5115(2) Å β = 90°
	c = 15.2096(6) Å γ = 120°	*c* = 15.46360(10) Å γ = 120°
Volume	2990.2(3) $Å^3$	3651.02(9) $Å^3$
Z	4	6
Density (calculated)	1.312 Mg/m^3	1.415 Mg/m^3
Absorption coefficient	4.286 mm^{-1}	5.177 mm^{-1}
F(000)	1220	1614
Crystal color, morphology	purple-black, needle	red-violet, needle
Crystal size	0.251 x 0.099 x 0.071 mm^3	0.309 x 0.051 x 0.035 mm^3
Theta range for data collection	3.387 to 77.925°	3.090 to 80.186°
Index ranges	$-9 \le h \le 18$, $-19 \le k \le 11$, $-18 \le l \le 19$	$-21 \le h \le 21$, $-20 \le k \le 21$, $-19 \le l \le 14$
Reflections collected	9024	29954
Independent reflections	2104 [*R*(int) = 0.0365]	2644 [*R*(int) = 0.0414]
Observed reflections	1860	2494
Completeness to theta = 74.504°	100.0%	99.8%
Absorption correction	Multi-scan	Multi-scan
Max. and min. transmission	1.00000 and 0.54887	1.00000 and 0.59775
Refinement method	Full-matrix least-squares on F^2	Full-matrix least-squares on F^2
Data / restraints / parameters	2104 / 0 / 130	2644 / 0 / 170
Goodness-of-fit on F^2	1.096	1.093
Final *R* indices [*I*>2sigma(*I*)]	*R*1 = 0.0389, *wR*2 = 0.1099	*R*1 = 0.0424, *wR*2 = 0.1145
R indices (all data)	*R*1 = 0.0433, *wR*2 = 0.1130	*R*1 = 0.0444, *wR*2 = 0.1166
Largest diff. peak and hole	0.227 and -0.435 e.$Å^{-3}$	2.123 and -0.335 e.$Å^{-3}$

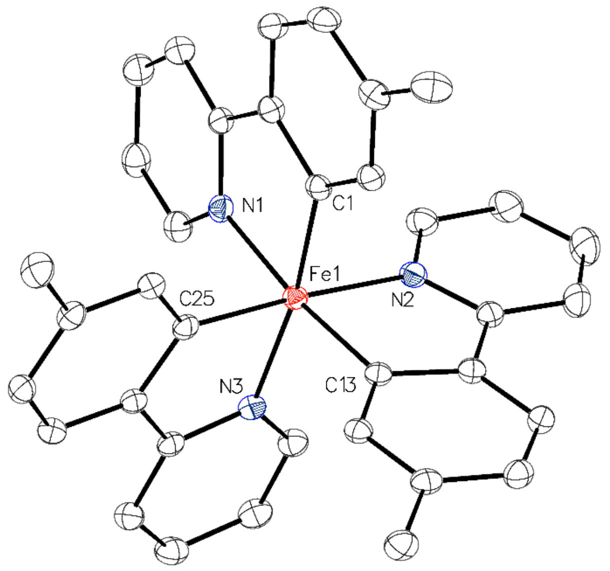

Figure S34. Crystal structure of **2-ptpy**. Hydrogen atoms and solvent molecules are omitted for clarity and the thermal ellipsoids are shown at 50% probability.

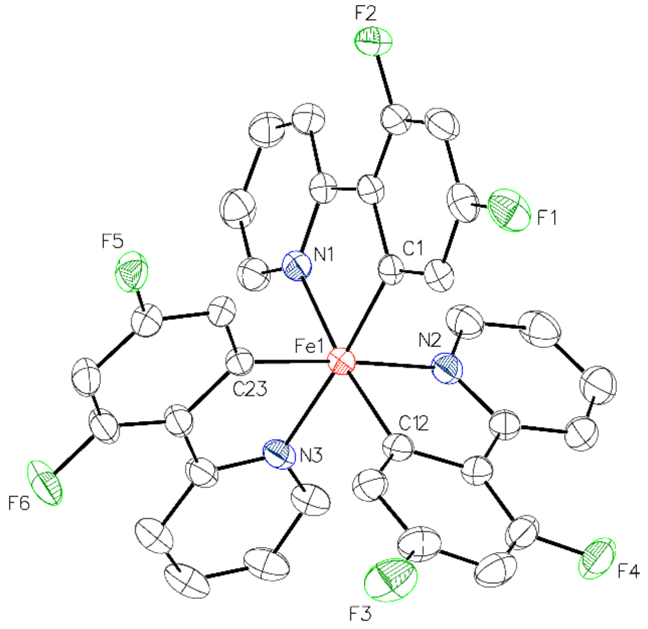

Figure S35. Crystal structure of **2-dfppy**. Hydrogen atoms are omitted for clarity and the thermal ellipsoids are shown at 50% probability.

Table S7. Crystal data and structure refinement of **2-ptpy** and **2-dfppy**.

	**2-ptpy**	**2-dfppy**
Empirical formula	C40 H38 Fe N3 O	C33 H18 F6 Fe N3
Formula weight	632.58	626.35
Temperature	100.00(10) K	99.99(10) K
Wavelength	1.54184 Å	1.54184 Å
Crystal system	monoclinic	monoclinic
Space group	$P2_1/n$	$P2_1/n$
Unit cell dimensions	a = 15.08620(10) Å α = 90°	a = 9.10500(10) Å α = 90°
	b = 9.95440(10) Å β= 91.4460(10)°	b = 23.3523(2) Å β= 97.9310(10)°
	c = 20.74630(10) Å γ= 90°	c = 12.21890(10) Å γ= 90°
Volume	3114.56(4) Å^3	2573.17(4) Å^3
Z	4	4
Density (calculated)	1.349 Mg/m^3	1.617 Mg/m^3
Absorption coefficient	4.167 mm^{-1}	5.361 mm^{-1}
F(000)	1332	1268
Crystal color, morphology	dark violet, plate	orange, needle
Crystal size	0.19 x 0.129 x 0.055 mm^3	0.133 x 0.041 x 0.019 mm^3
Theta range for data collection	3.580 to 80.207°	3.786 to 80.348°
Index ranges	$-18 \le h \le 15$, $-12 \le k \le 12$, $-26 \le l \le 26$	$-11 \le h \le 11$, $-23 \le k \le 29$, $-15 \le l \le 15$
Reflections collected	54363	41066
Independent reflections	6710 [R(int) = 0.0394]	5567 [R(int) = 0.0391]
Observed reflections	6236	4954
Completeness to theta = 74.504°	100.0%	100.0%
Absorption correction	Multi-scan	Multi-scan
Max. and min. transmission	1.00000 and 0.72581	1.00000 and 0.78666
Refinement method	Full-matrix least-squares on F^2	Full-matrix least-squares on F^2
Data / restraints / parameters	6710 / 0 / 409	5567 / 0 / 388
Goodness-of-fit on F^2	1.075	1.090
Final R indices [I>2sigma(I)]	$R1$ = 0.0349, $wR2$ = 0.0893	$R1$ = 0.0402, $wR2$ = 0.1096
R indices (all data)	$R1$ = 0.0379, $wR2$ = 0.0910	$R1$ = 0.0456, $wR2$ = 0.1128
Largest diff. peak and hole	0.422 and -0.390 e.Å^{-3}	0.573 and -0.351 e.Å^{-3}

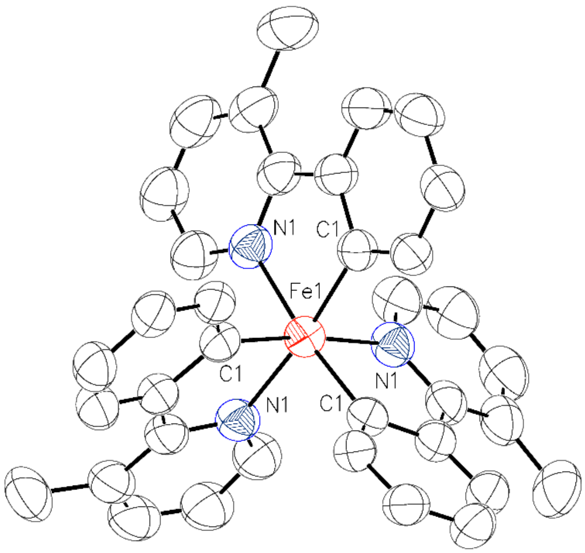

Figure S36. Crystal structure of **2-mppy**. Hydrogen atoms are omitted for clarity and the thermal ellipsoids are shown at 50% probability.

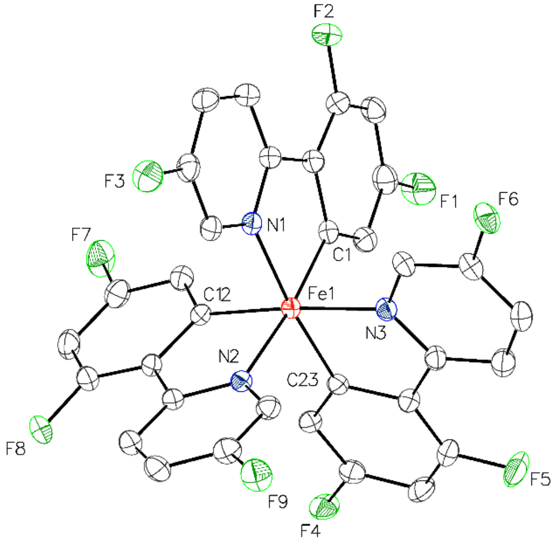

Figure S37. Crystal structure of **2-dfpfpy**. Hydrogen atoms are omitted for clarity and the thermal ellipsoids are shown at 50% probability.

Table S8. Crystal data and structure refinement of **2-mppy** and **2-dfpfpy**.

	**2-mppy**	**2-dfpfpy**
Empirical formula	C36 H30 Fe N3	C33 H15 F9 Fe N3
Formula weight	560.48	680.33
Temperature	173.00(10) K	100.00(10) K
Wavelength	1.54184 Å	1.54184 Å
Crystal system	trigonal	monoclinic
Space group	*R*-3	$P2_1/n$
Unit cell dimensions	*a* = 15.0750(4) Å α= 90°	*a* = 9.46120(10) Å α= 90°
	b = 15.0750(4) Å β= 90°	*b* = 23.5947(2) Å β= 98.1220(10)°
	c = 28.6238(8) Å γ= 120°	*c* = 12.11550(10) Å γ= 90°
Volume	5633.4(3) $Å^3$	2677.47(4) $Å^3$
Z	6	4
Density (calculated)	0.991 Mg/m^3	1.688 Mg/m^3
Absorption coefficient	3.384 mm^{-1}	5.375 mm^{-1}
F(000)	1758	1364
Crystal color, morphology	dark red, block	orange, plate
Crystal size	0.326 x 0.197 x 0.16 mm^3	0.119 x 0.08 x 0.032 mm^3
Theta range for data collection	3.721 to 81.301°	3.747 to 80.187°
Index ranges	$-17 \le h \le 18$, $-18 \le k \le 15$, $-36 \le l \le 36$	$-12 \le h \le 12$, $-29 \le k \le 29$, $-15 \le l \le 13$
Reflections collected	16014	33566
Independent reflections	2720 [*R*(int) = 0.0323]	5726 [*R*(int) = 0.0352]
Observed reflections	2384	5139
Completeness to theta = 74.504°	100.0%	99.9%
Absorption correction	Multi-scan	Multi-scan
Max. and min. transmission	1.00000 and 0.57851	1.00000 and 0.81725
Refinement method	Full-matrix least-squares on F^2	Full-matrix least-squares on F^2
Data / restraints / parameters	2720 / 0 / 122	5726 / 0 / 415
Goodness-of-fit on F^2	1.108	1.113
Final *R* indices [*I*>2sigma(*I*)]	*R*1 = 0.0383, *wR*2 = 0.1111	*R*1 = 0.0366, *wR*2 = 0.0933
R indices (all data)	*R*1 = 0.0431, *wR*2 = 0.1164	*R*1 = 0.0419, *wR*2 = 0.0957
Largest diff. peak and hole	0.317 and -0.211 e.$Å^{-3}$	0.341 and -0.458 e.$Å^{-3}$

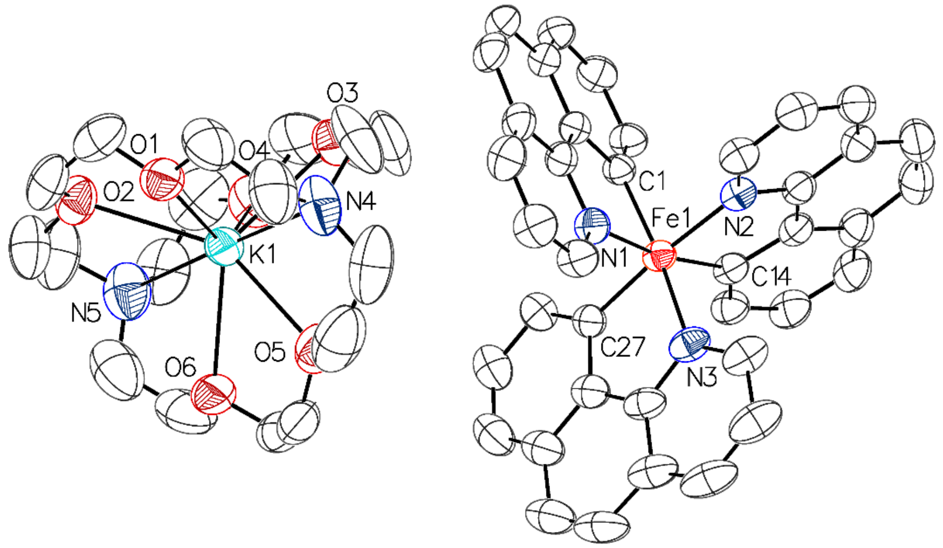

Figure S38. Crystal structure of **1-bzq**. Hydrogen atoms are omitted for clarity and the thermal ellipsoids are shown at 50% probability.

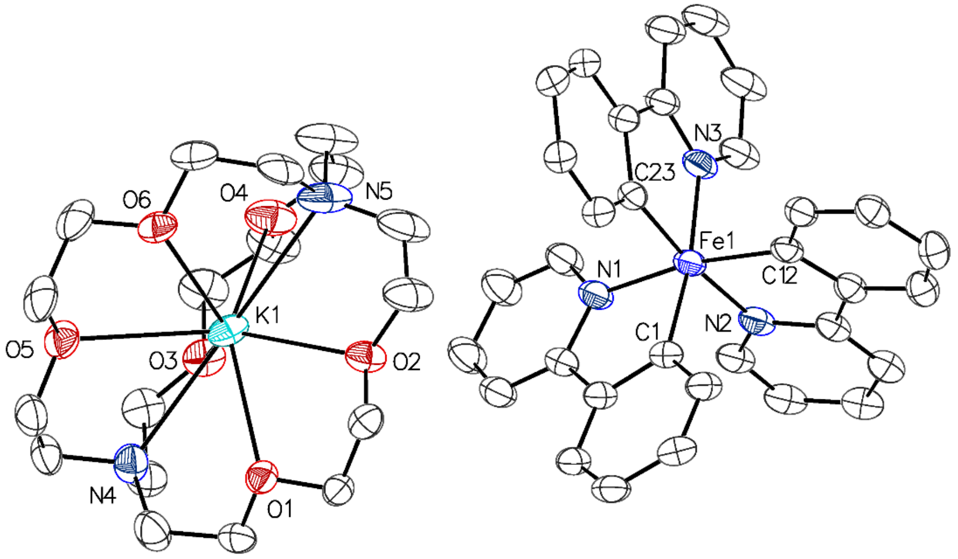

Figure S39. Crystal structure of **1-ppy**. Hydrogen atoms and solvent molecules are omitted for clarity and the thermal ellipsoids are shown at 50% probability.

Table S9. Crystal data and structure refinement of **1-bzq** and **1-ppy**.

	**1-bzq**	**1-ppy**
Empirical formula	C57 H60 Fe K N5 O6	C59 H76 Fe K N5 O8
Formula weight	1006.05	1078.19
Temperature	232.99(10) K	100.00(10) K
Wavelength	1.54184 Å	1.54184 Å
Crystal system	monoclinic	triclinic
Space group	$P2_1/n$	*P*-1
Unit cell dimensions	*a* = 12.6152(2) Å α= 90°	*a* = 12.9303(2) Å α= 91.8400(10)°
	b = 13.0318(2) Å β= 99.3230(10)°	*b* = 13.4933(2) Å β= 90.5310(10)°
	c = 31.1990(4) Å γ= 90°	*c* = 15.5169(2) Å γ= 93.0570(10)°
Volume	5061.33(13) $Å^3$	2701.86(7) $Å^3$
Z	4	2
Density (calculated)	1.320 Mg/m^3	1.325 Mg/m^3
Absorption coefficient	3.576 mm^{-1}	3.409 mm^{-1}
F(000)	2120	1148
Crystal color, morphology	green-black, needle	orange-black, plate
Crystal size	0.246 x 0.122 x 0.072 mm^3	0.3 x 0.12 x 0.033 mm^3
Theta range for data collection	2.871 to 77.879°	3.282 to 80.614°
Index ranges	$-15 \leq h \leq 15$, $-16 \leq k \leq 14$, $-39 \leq l \leq 39$	$-16 \leq h \leq 16$, $-17 \leq k \leq 14$, $-19 \leq l \leq 19$
Reflections collected	66201	91029
Independent reflections	10634 [*R*(int) = 0.0675]	11619 [*R*(int) = 0.0632]
Observed reflections	9158	10296
Completeness to theta = 74.504°	99.7%	99.8%
Absorption correction	Multi-scan	Multi-scan
Max. and min. transmission	1.00000 and 0.54361	1.00000 and 0.64132
Refinement method	Full-matrix least-squares on F^2	Full-matrix least-squares on F^2
Data / restraints / parameters	10634 / 0 / 631	11619 / 162 / 785
Goodness-of-fit on F^2	1.051	1.079
Final *R* indices [*I*>2sigma(*I*)]	*R*1 = 0.0464, *wR*2 = 0.1285	*R*1 = 0.0550, *wR*2 = 0.1542
R indices (all data)	*R*1 = 0.0531, *wR*2 = 0.1342	*R*1 = 0.0610, *wR*2 = 0.1603
Largest diff. peak and hole	0.463 and -0.374 e.$Å^{-3}$	0.677 and -0.748 e.$Å^{-3}$

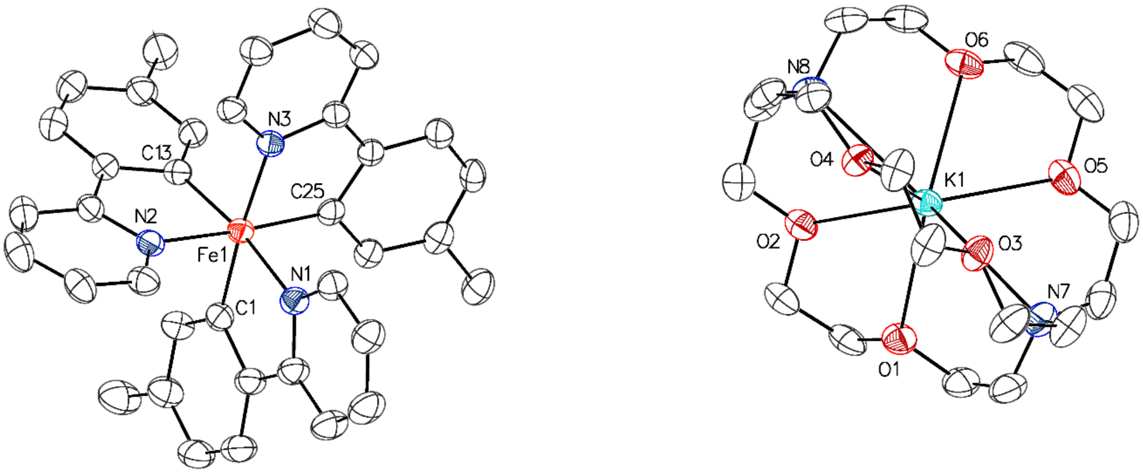

Figure S40. Crystal structure of **1-ptpy**. Hydrogen atoms and solvent molecules are omitted for clarity and the thermal ellipsoids are shown at 50% probability.

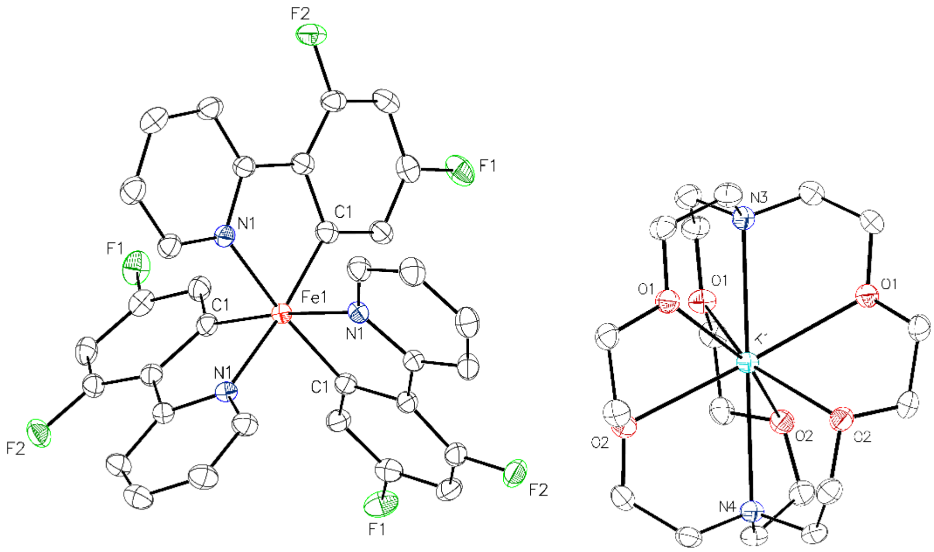

Figure S41. Crystal structure of **1-dfppy**. Hydrogen atoms are omitted for clarity and the thermal ellipsoids are shown at 50% probability.

Table S10. Crystal data and structure refinement of **1-ptpy** and **1-dfppy**.

	**1-ppy**	**1-dfppy**
Empirical formula	C62 H83 Fe K N5 O8	C51 H54 F6 Fe K N5 O6
Formula weight	1121.28	1041.94
Temperature	100.00(11) K	100.00(10) K
Wavelength	1.54184 Å	1.54184 Å
Crystal system	triclinic	trigonal
Space group	*P*-1	*R*3*c*
Unit cell dimensions	*a* = 17.7676(2) Å α= 114.6990(10)°	*a* = 14.66160(10) Å α= 90°
	b = 19.7393(2) Å β= 111.7550(10)°	*b* = 14.66160(10) Å β= 90°
	c = 20.0446(2) Å γ= 91.6040(10)°	*c* = 75.2940(6) Å γ= 120°
Volume	5791.03(12) $Å^3$	14017.0(2) $Å^3$
Z	4	12
Density (calculated)	1.286 Mg/m^3	1.481 Mg/m^3
Absorption coefficient	3.200 mm^{-1}	4.083 mm^{-1}
F(000)	2396	6504
Crystal color, morphology	dark blue, block	dark blue, block
Crystal size	0.142 x 0.109 x 0.06 mm^3	0.188 x 0.121 x 0.092 mm^3
Theta range for data collection	3.301 to 77.978°	3.674 to 80.403°
Index ranges	$-22 \leq h \leq 22, -24 \leq k \leq 22, -25 \leq l \leq 25$	$-18 \leq h \leq 18, -18 \leq k \leq 18, -91 \leq l \leq 95$
Reflections collected	89789	99250
Independent reflections	23903 [*R*(int) = 0.0335]	6745 [*R*(int) = 0.0438]
Observed reflections	20234	6574
Completeness to theta = 74.504°	99.7%	100.0%
Absorption correction	Multi-scan	Multi-scan
Max. and min. transmission	1.00000 and 0.88290	1.00000 and 0.85026
Refinement method	Full-matrix least-squares on F^2	Full-matrix least-squares on F^2
Data / restraints / parameters	23903 / 200 / 1559	6745 / 1 / 421
Goodness-of-fit on F^2	1.023	1.090
Final *R* indices [*I*>2sigma(*I*)]	*R*1 = 0.0490, *wR*2 = 0.1297	*R*1 = 0.0340, *wR*2 = 0.0978
R indices (all data)	*R*1 = 0.0583, *wR*2 = 0.1351	*R*1 = 0.0347, *wR*2 = 0.0984
Largest diff. peak and hole	0.545 and -0.615 e.$Å^{-3}$	0.297 and -0.802 e.$Å^{-3}$

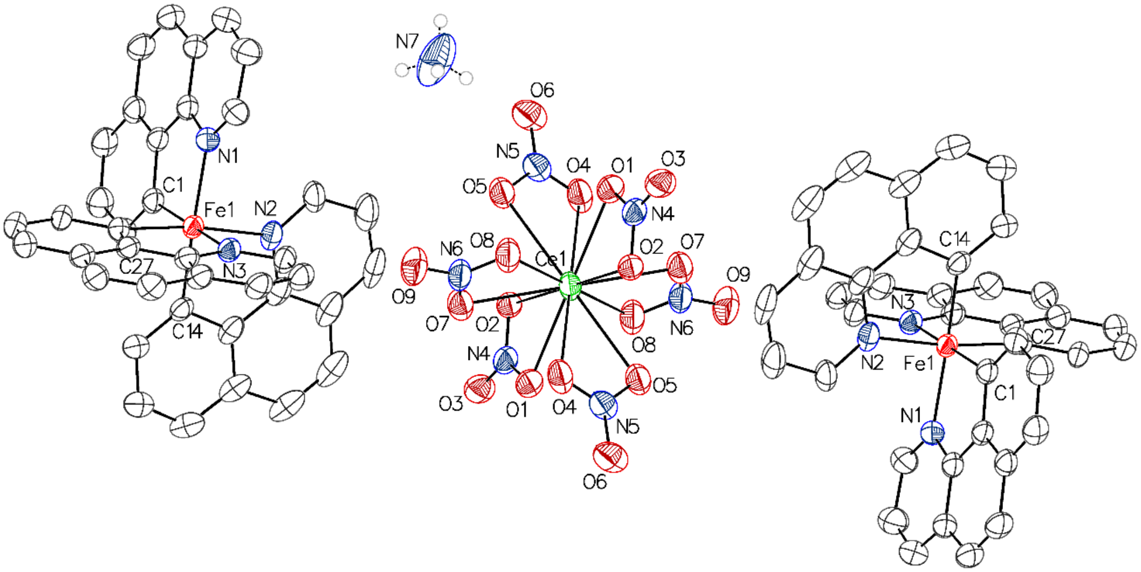

Figure S42. Crystal structure of **3-bzq**. Hydrogen atoms and solvent molecules are omitted for clarity and the thermal ellipsoids are shown at 50% probability.

Table S16. Crystal data and structure refinement of **3-bzq.**

	**3-bzq**
Empirical formula	C94 H92 Ce Fe2 N13 O26
Formula weight	2071.62
Temperature	100.00(10) K
Wavelength	1.54184 Å
Crystal system	triclinic
Space group	*P*-1
Unit cell dimensions	*a* = 11.9758(3) Å α= 105.165(2)°
	b = 13.1767(3) Å β= 106.901(3)°
	c = 16.3831(4) Å γ= 101.067(2)°
Volume	2284.72(10) $Å^3$
Z	1
Density (calculated)	1.506 Mg/m^3
Absorption coefficient	7.019 mm^{-1}
F(000)	1065
Crystal color, morphology	brown-green, plate
Crystal size	0.147 x 0.115 x 0.029 mm^3
Theta range for data collection	3.632 to 77.895°
Index ranges	-15 ≤ *h* ≤ 12, -16 ≤ *k* ≤ 16, -20 ≤ *l* ≤ 20
Reflections collected	53682
Independent reflections	9500 [*R*(int) = 0.0752]
Observed reflections	7795
Completeness to theta = 74.504°	99.2%
Absorption correction	Multi-scan
Max. and min. transmission	1.00000 and 0.53611
Refinement method	Full-matrix least-squares on F^2
Data / restraints / parameters	9500 / 127 / 680
Goodness-of-fit on F^2	1.052
Final *R* indices [*I*>2sigma(*I*)]	*R*1 = 0.0596, *wR*2 = 0.1608
R indices (all data)	*R*1 = 0.0717, *wR*2 = 0.1703
Largest diff. peak and hole	1.618 and -1.904 e.$Å^{-3}$

9. Density Functional Theory

9.1 Computation Details

All geometry optimizations of intermediates and transition states were achieved using spin- unrestricted UB3LYP[10]-D3[26]/6-31G(d)[27] method in THF solvent using the CPCM solvent model[28] with "opt=noeigen" and "guess=(mix,always)" keywords as implemented in Gaussian16[29]. Frequency calculations were also done for all the stationary points, and transition states were characterized by the presence of one unique imaginary frequency, which suggested that they were first-order saddle points on the potential energy surface. Intrinsic Reaction Coordinate (IRC) calculations were done on the transition states to verify that they were the correct transition state associated with the reaction. The endpoint geometries obtained from the IRC calculations were further optimized to verify the authenticity of the transition state. The thermochemistry: enthalpy (ΔH) and free energy (ΔG) were obtained at the temperature of 298 K. Energies were further corrected using single point calculations at UB3LYP-D3/def2SVP[14]-CPCM[28](THF) level of theory. All structural figures were generated with CYLview.[30] Distances in structural figures are shown in Å and energies are in kcal/mol.

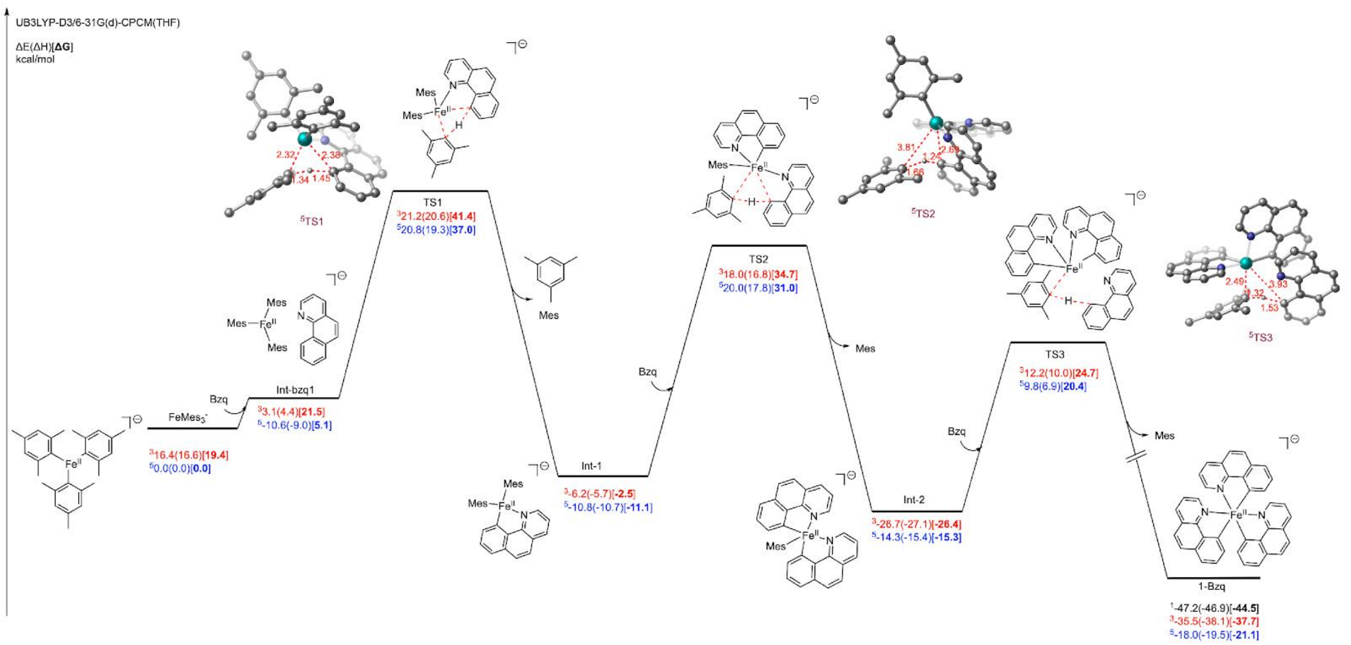

Figure S43. Gibbs free energy profile for full C-H activation pathways calculated at UB3LYP-D3-6-31G(d)-CPCM(THF) for triplet and quintet spin states.

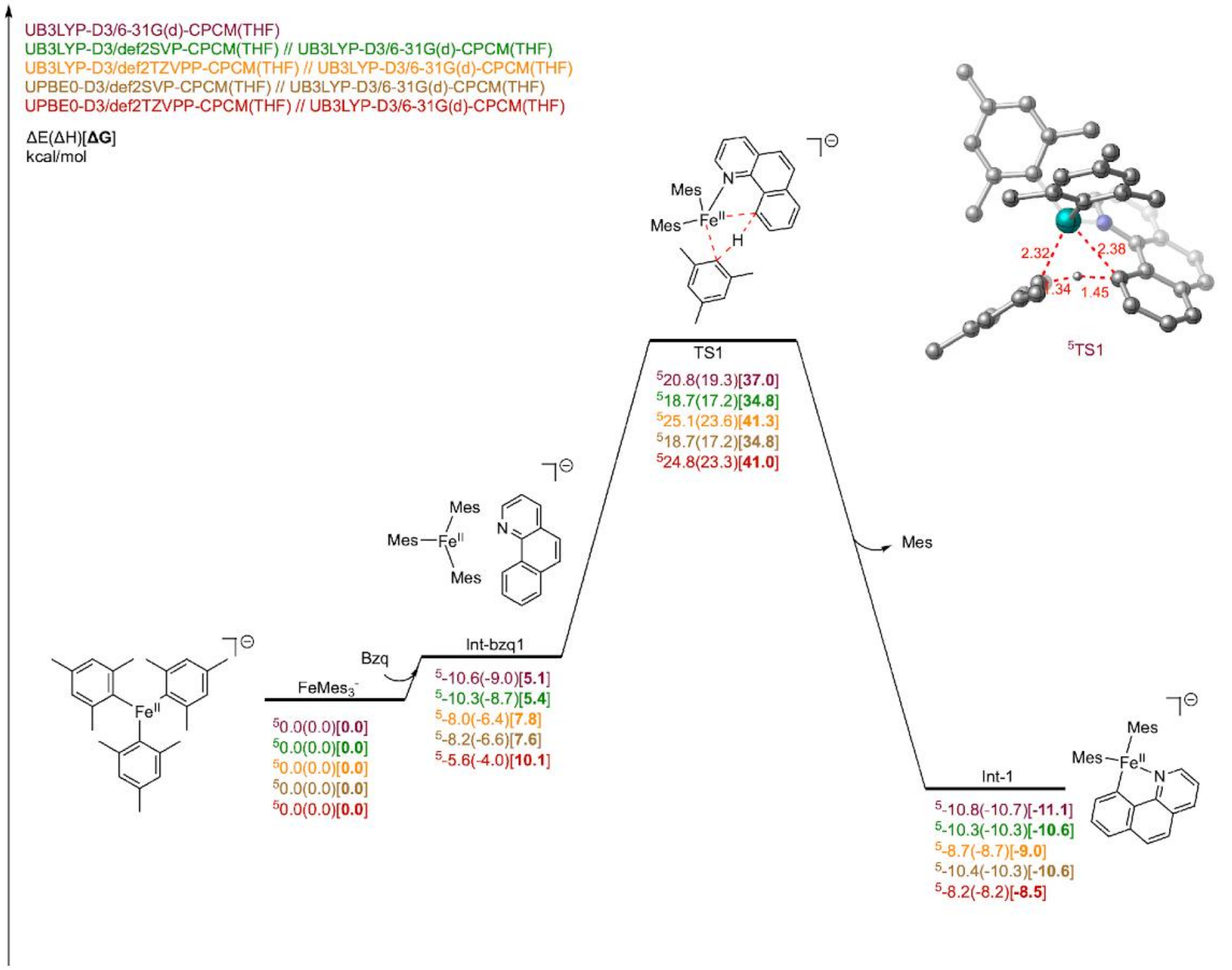

Figure S44. Gibbs free energy profile for first sp2 C-H activation step calculated at five different levels of theory for quintet spin state (lowest energy spin state).

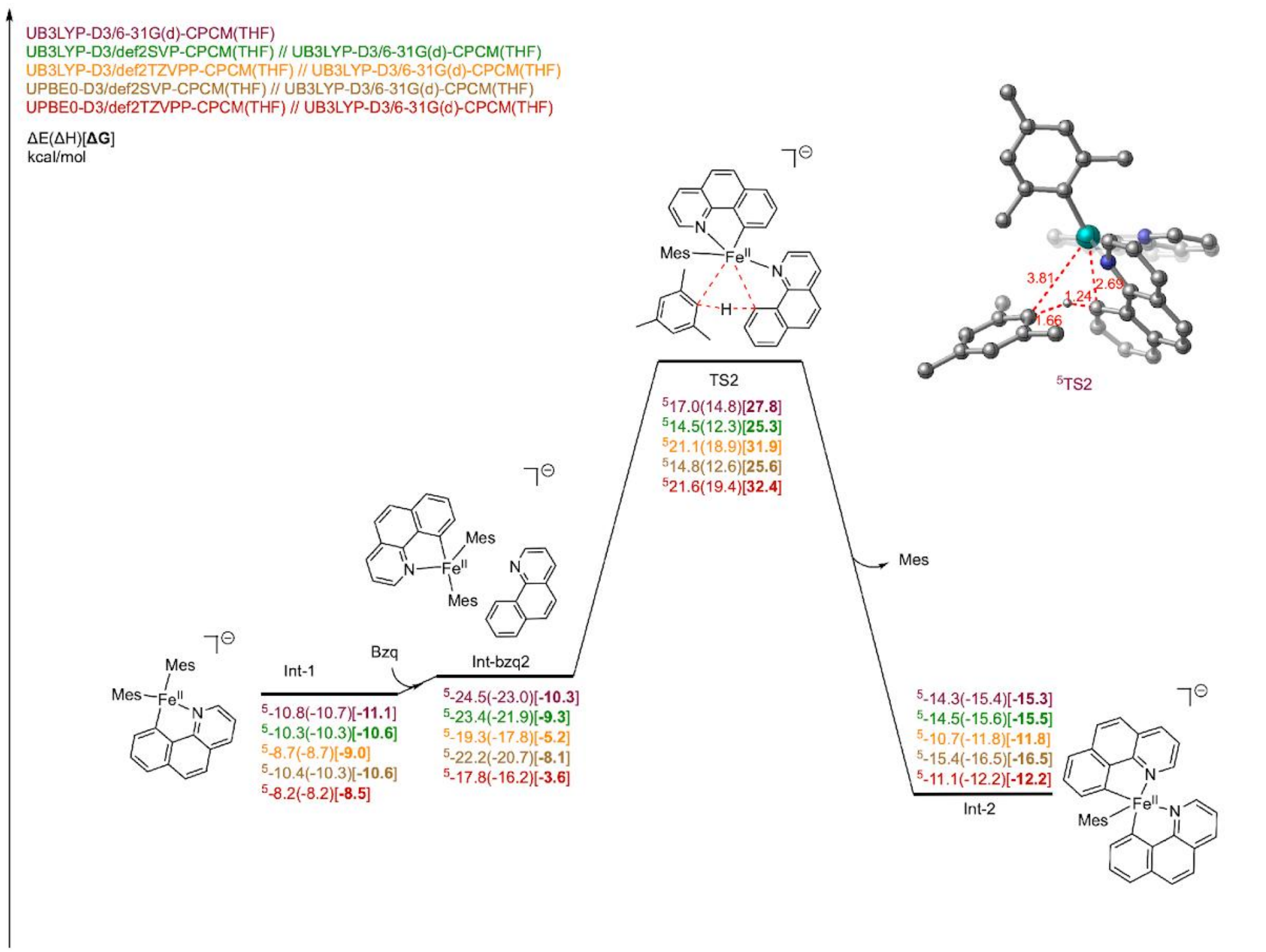

Figure S45. Gibbs free energy profile for second sp2 C-H activation step calculated at five different levels of theory for quintet spin state (lowest energy spin state).

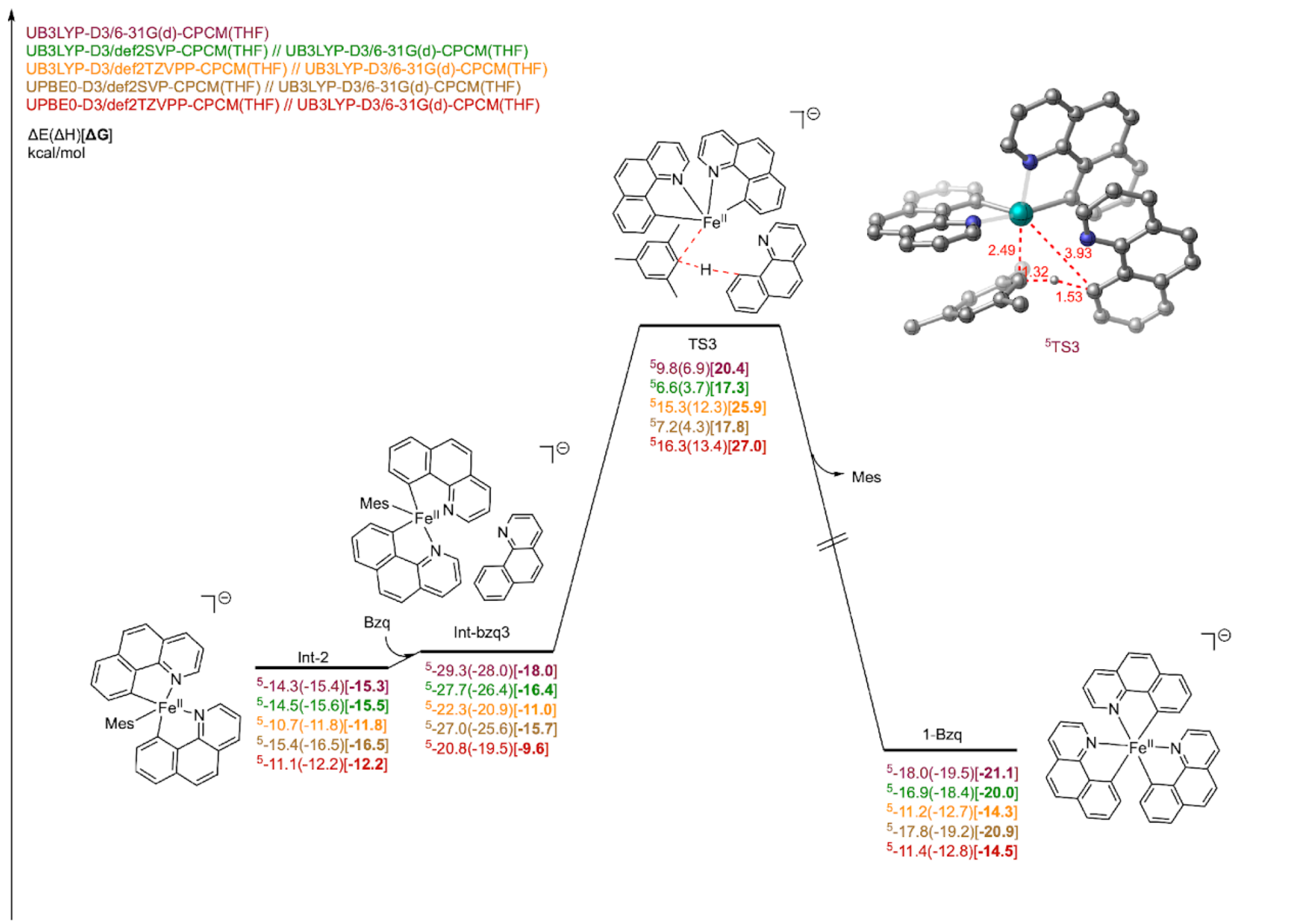

Figure S46. Gibbs free energy profile for third sp2 C-H activation step calculated at five different levels of theory for quintet spin state (lowest energy spin state).

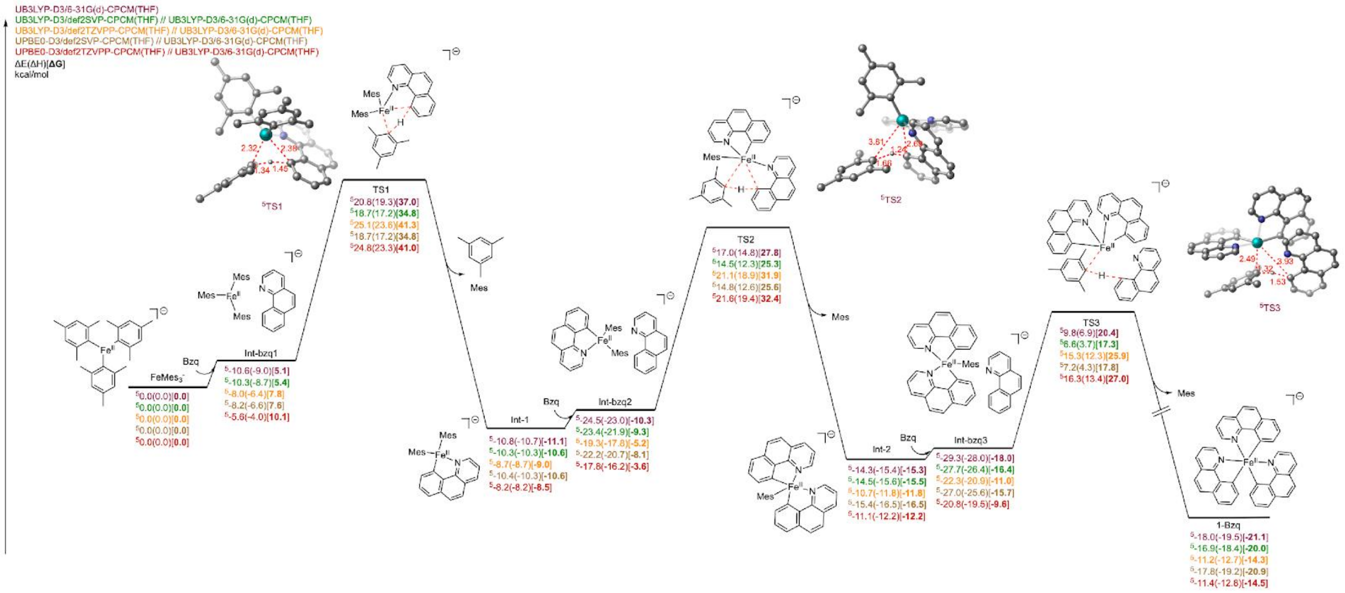

Figure S47. Gibbs free energy profile for the full sp2 C-H activation pathway calculated at five different levels of theory for quintet spin state (lowest energy spin state).

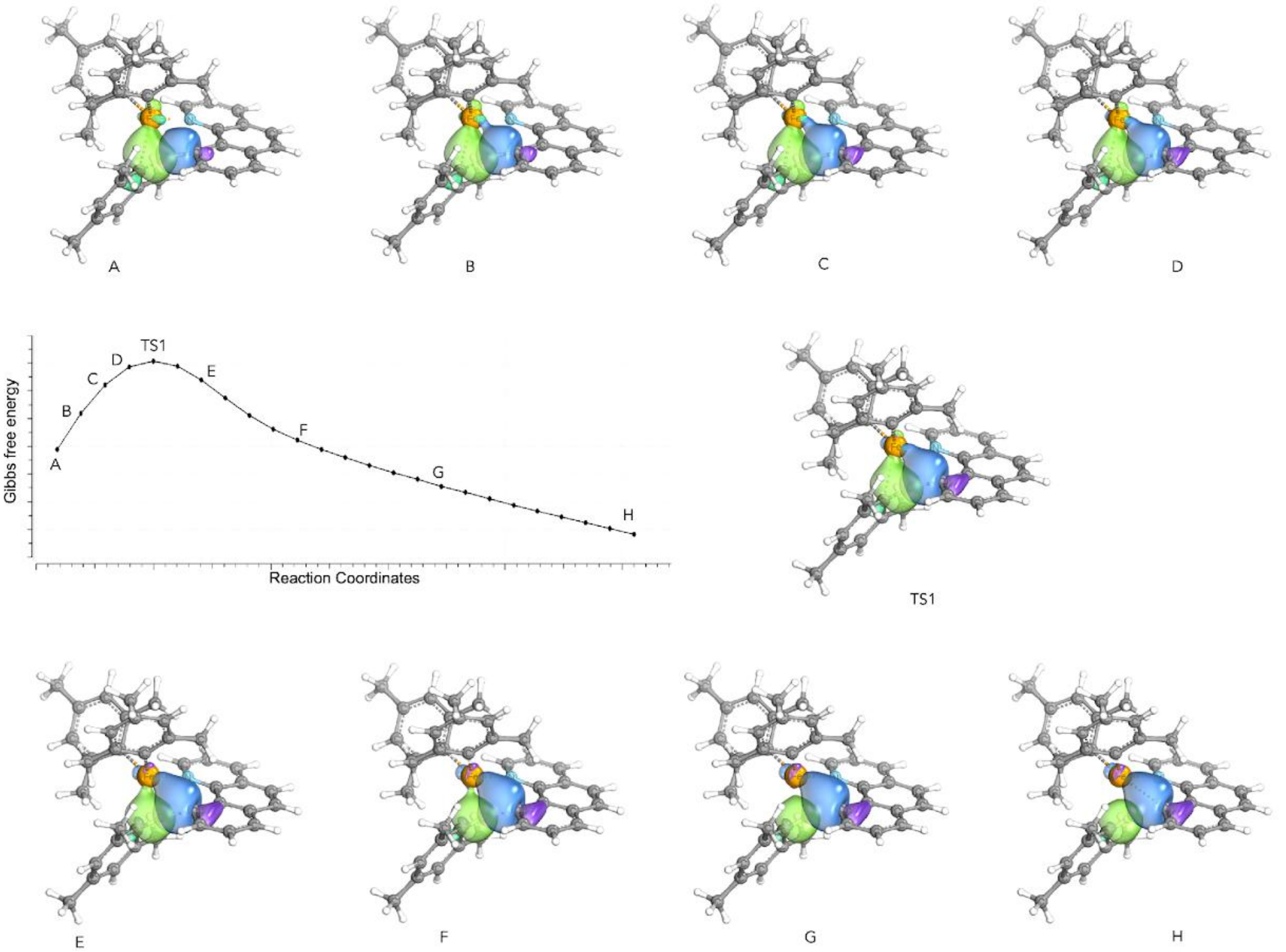

Figure S48. Intrinsic bond orbitals (IBO) analysis for the transition state of the first sp2 C-H activation step TS1 for quintet spin state (lowest energy spin state) along with its intrinsic reaction coordinates (IRC).

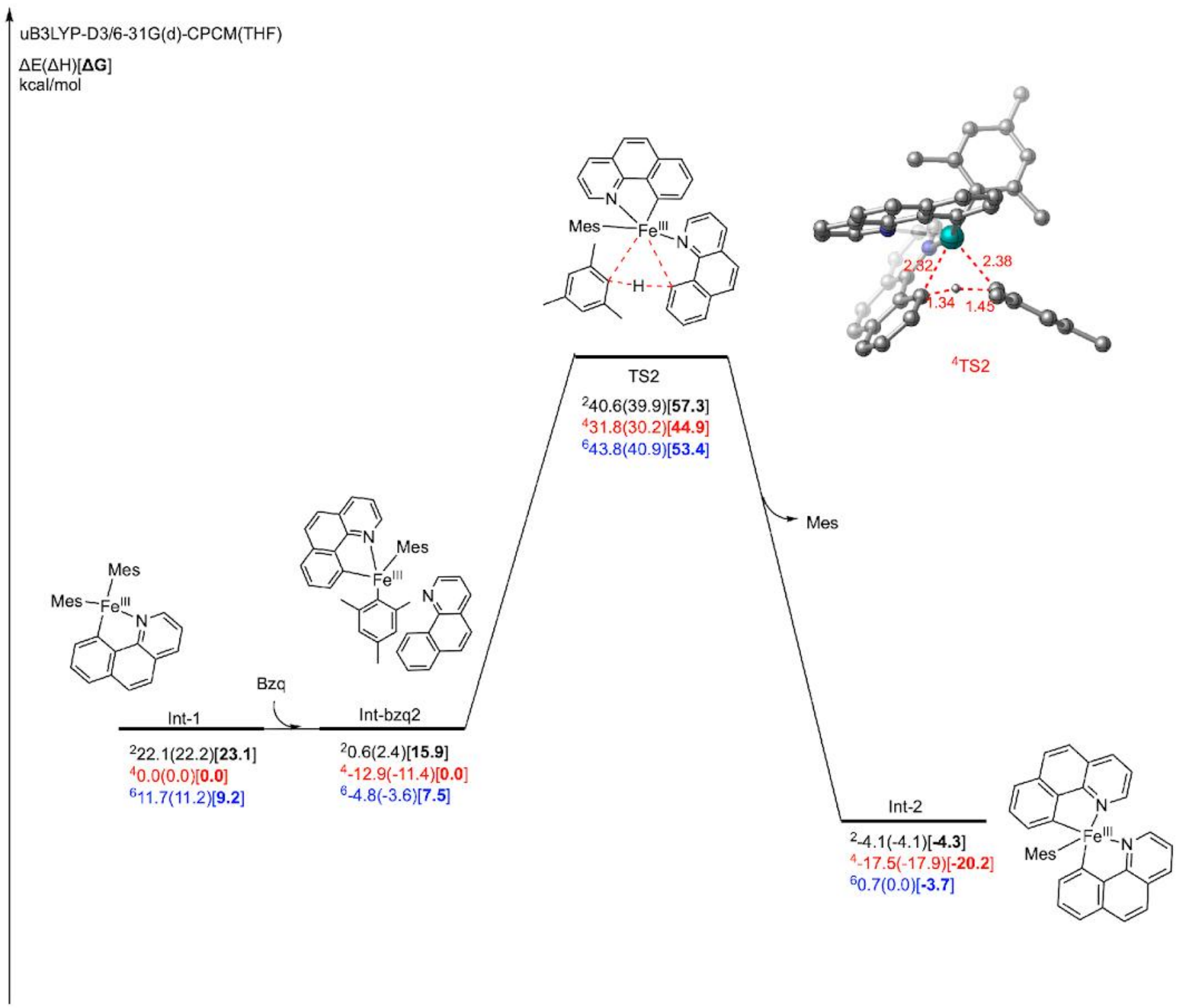

Figure S49. Gibbs free energy profile for the second sp2 C-H activation step starting from the oxidized Fe(III)-intermediate Int-1 calculated at UB3LYP-D3-6-31G(d)-CPCM(THF) for doublet, quartet and sextet spin states.

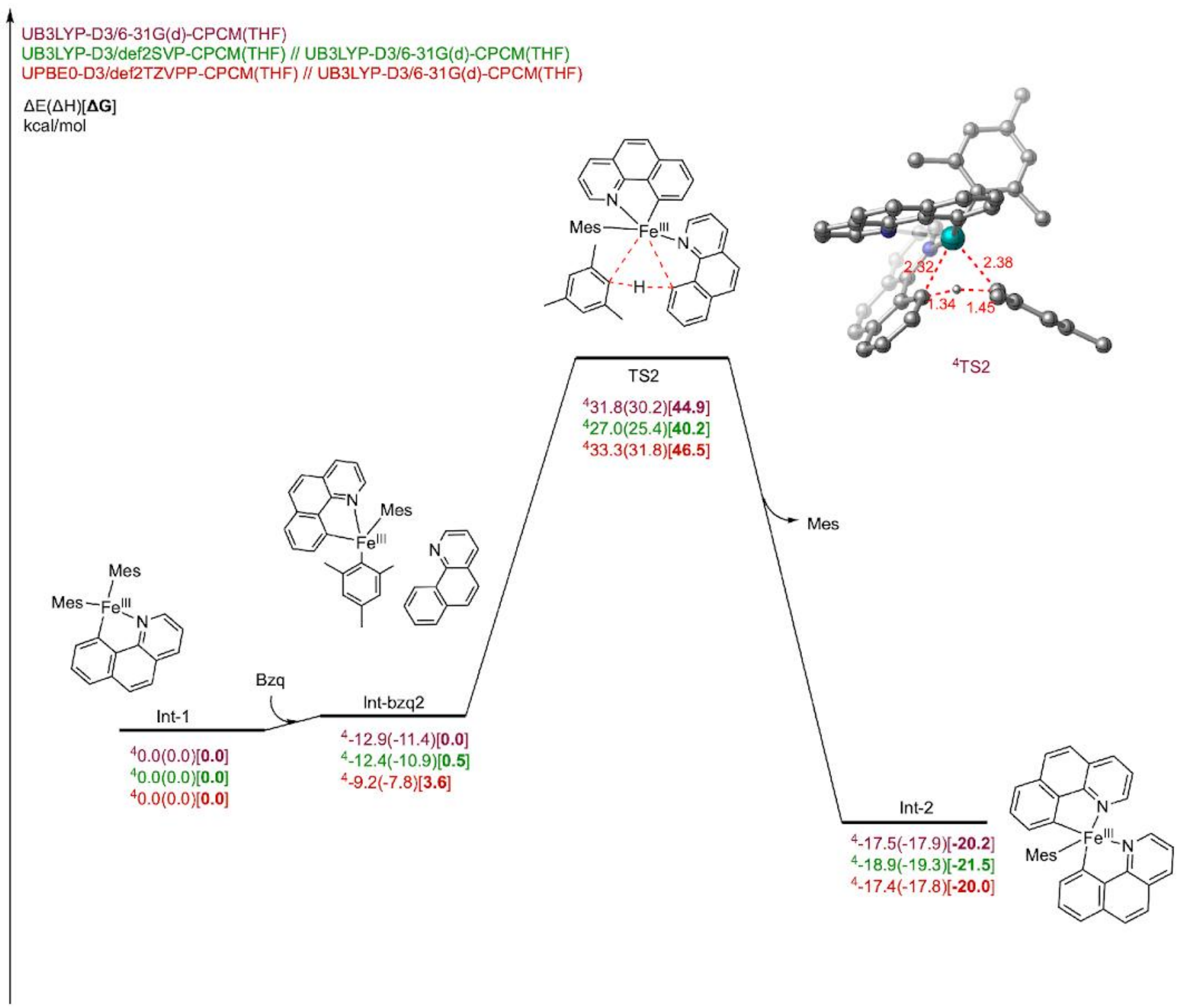

Figure S50. Gibbs free energy profile for the second sp2 C-H activation step starting from the oxidized Fe(III)-intermediate Int-1 at three different levels of theory for quartet spin state (lowest energy spin state).

9.2 Molecular Coordinates

Cartesian coordinates (xyz format) and energies of all the structures involved in each reaction mechanism studied calculated at the UB3LYP-D3/6-31-G (d) level of theory.

Bzq

E(UB3LYP) = -555.597306431 a.u.

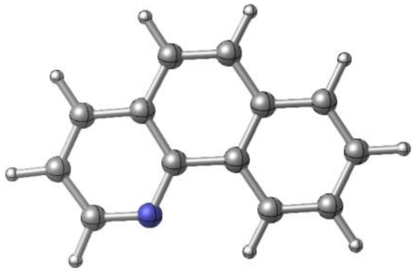

C	-5.049561	-1.937854	17.895506
C	-3.668734	-1.911321	17.894374
C	-2.962631	-0.686113	17.895949
C	-3.687693	0.542857	17.898717
C	-5.100505	0.483172	17.899872
C	-5.76894	-0.727788	17.8983
C	-1.526834	-0.665822	17.894831
C	-2.946053	1.794082	17.900262
C	-1.519607	1.764626	17.899135
C	-0.833789	0.504198	17.896383
C	-1.365122	4.057457	17.902971
C	-2.765631	4.205098	17.904171
C	-3.55161	3.06955	17.902814
H	-1.000175	-1.617236	17.892724
H	-5.57944	-2.886313	17.894276
H	-3.101627	-2.839217	17.892254
H	-5.681864	1.399346	17.902084
H	-6.855384	-0.743421	17.899245
H	0.251114	0.5319	17.895595
H	-0.723721	4.93812	17.904044
H	-3.211305	5.195331	17.906121
H	-4.632372	3.167085	17.90365
N	-0.751441	2.885523	17.900521

- Thermochemistry -

Temperature 298.150 Kelvin. Pressure 1.00000 Atm.

Zero-point correction= 0.183213 (Hartree/Particle)
Thermal correction to Energy= 0.192443
Thermal correction to Enthalpy= 0.193387
Thermal correction to Gibbs Free Energy= 0.148588
Sum of electronic and zero-point Energies= -555.414094
Sum of electronic and thermal Energies= -555.404864
Sum of electronic and thermal Enthalpies= -555.403920
Sum of electronic and thermal Free Energies= -555.448718

Charge = 0 Multiplicity = 1

Mes

E(RB3LYP) = -350.215982865 a.u.

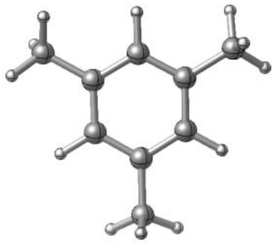

C	-0.749986	3.419174	-0.341344
C	0.64888	3.400843	-0.342559
C	1.333778	4.620975	-0.358881
C	0.650221	5.841596	-0.364004
C	-0.748982	5.824637	-0.362522
C	-1.464264	4.622372	-0.353815
H	-1.294944	2.476523	-0.334918
H	2.422624	4.620311	-0.366251
H	-1.292922	6.767837	-0.372782
C	-2.975302	4.622134	-0.395982
H	-3.390717	5.519317	0.075789
H	-3.342485	4.598988	-1.430905
H	-3.391399	3.747036	0.1152
C	1.406301	7.150345	-0.341836
H	0.847957	7.946099	-0.847011
H	1.58842	7.484752	0.688429
H	2.382524	7.058726	-0.830425
C	1.404534	2.092435	-0.297485
H	2.372003	2.169617	-0.805719
H	1.605175	1.786772	0.738252
H	0.837018	1.283523	-0.770386

- Thermochemistry -

Temperature 298.150 Kelvin. Pressure 1.00000 Atm.

Zero-point correction= 0.183578 (Hartree/Particle)
Thermal correction to Energy= 0.193476
Thermal correction to Enthalpy= 0.194420
Thermal correction to Gibbs Free Energy= 0.146420
Sum of electronic and zero-point Energies= -350.032405
Sum of electronic and thermal Energies= -350.022507
Sum of electronic and thermal Enthalpies= -350.021562
Sum of electronic and thermal Free Energies= -350.069563

Charge = 0 Multiplicity = 1

^{1}SM

E(UB3LYP) = -2312.43029686 a.u.

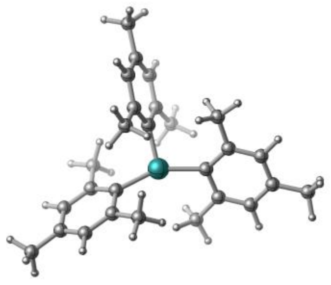

Fe	3.306685	4.075409	17.74168
C	0.064915	1.801576	19.582888
C	-0.982059	2.727206	19.635272
C	-0.760806	3.984227	19.067823
H	-1.563899	4.723653	19.087001
C	0.463986	4.308412	18.469328
C	1.293765	2.114494	18.9889
C	5.420781	1.176049	15.540361
C	4.690796	1.011764	14.358723
C	3.523937	1.770132	14.211496
H	2.938579	1.66927	13.295412
C	3.089395	2.653817	15.203665
C	5.005969	2.060265	16.54267
C	5.638056	7.379083	18.89414
C	6.301431	7.908355	17.785098
C	6.133565	7.262652	16.555325
H	6.643936	7.664084	15.677702

C	5.331843	6.120712	16.424135
C	4.836663	6.236807	18.771876
C	4.158318	5.677674	20.016599
H	4.32016	6.315783	20.894546
H	3.068968	5.577181	19.901763
H	4.544746	4.67963	20.267693
C	5.210986	5.469101	15.062586
H	5.581277	4.439277	15.086074
H	4.163288	5.39804	14.750828
H	5.761709	6.024206	14.291802
C	7.198003	9.119647	17.911862
H	8.254042	8.835688	18.033023
H	7.141126	9.758736	17.021447
H	6.927688	9.731315	18.781484
H	5.750278	7.864509	19.865557
C	4.649637	5.567749	17.535841
C	0.626243	5.687156	17.849155
H	1.478569	6.235632	18.27118
H	0.814929	5.613877	16.771265
H	-0.26703	6.309233	17.991266
C	-2.292999	2.388663	20.308425
H	-2.543799	1.326539	20.191554
H	-2.265467	2.592397	21.389739
H	-3.122179	2.9757	19.893759
C	2.387059	1.064522	18.981624
H	2.82273	0.963335	17.983281
H	3.21018	1.350441	19.652373
H	2.022531	0.083113	19.312385
H	-0.086436	0.809435	20.01294
C	1.539328	3.382056	18.400218
C	3.820879	2.827789	16.406019
C	5.125773	0.027623	13.296833
H	6.21421	-0.11435	13.300988
H	4.673822	-0.966207	13.440134
H	4.838329	0.36459	12.292342
C	1.82827	3.459494	14.989625
H	1.112044	3.273407	15.798342
H	2.044463	4.537713	15.009756
H	1.347305	3.234808	14.029052
C	5.851447	2.235492	17.785781
H	6.184095	3.27745	17.877853
H	5.274165	2.010044	18.692806
H	6.735855	1.585226	17.782625
H	6.340853	0.604992	15.679507

- Thermochemistry -

Temperature 298.150 Kelvin. Pressure 1.00000 Atm.

Zero-point correction= 0.517400 (Hartree/Particle)
Thermal correction to Energy= 0.549962
Thermal correction to Enthalpy= 0.550906
Thermal correction to Gibbs Free Energy= 0.450952
Sum of electronic and zero-point Energies= -2311.912897
Sum of electronic and thermal Energies= -2311.880335
Sum of electronic and thermal Enthalpies= -2311.879391
Sum of electronic and thermal Free Energies= -2311.979345

Charge = -1 Multiplicity = 1

^{3}SM

E(UB3LYP) = -2312.45352840 a.u.

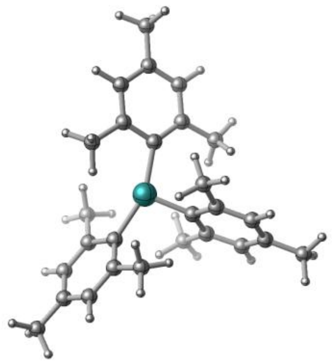

Fe	3.29017	4.16226	17.76850
C	0.09789	1.72674	19.54637
C	-0.98906	2.60522	19.57798
C	-0.79943	3.88180	19.04476
H	-1.63147	4.58875	19.05165
C	0.43154	4.26913	18.49870
C	1.33293	2.09984	19.00100
C	5.35335	1.21387	15.53339
C	4.60962	1.06535	14.35864
C	3.45205	1.83869	14.22862
H	2.85669	1.75242	13.31781
C	3.03453	2.71901	15.23256
C	4.96353	2.09749	16.54675
C	5.60832	7.45816	18.86179
C	6.39600	7.88692	17.79577
C	6.32022	7.16737	16.59411
H	6.92474	7.49807	15.74720
C	5.49130	6.04988	16.45327

C	4.77628	6.33578	18.72806
C	3.96179	5.89427	19.93822
H	3.49235	6.74179	20.45448
H	3.13986	5.20366	19.68812
H	4.58322	5.36542	20.67375
C	5.46528	5.32422	15.12499
H	5.87251	4.31167	15.21811
H	4.43870	5.19341	14.76820
H	6.03750	5.86014	14.35621
C	7.31225	9.08357	17.92055
H	8.37140	8.78830	17.90667
H	7.17108	9.79031	17.09167
H	7.13670	9.62787	18.85617
H	5.63892	8.00483	19.80666
C	4.68725	5.59265	17.53065
C	0.55514	5.67380	17.93086
H	1.36931	6.23858	18.40490
H	0.78311	5.65053	16.85874
H	-0.36689	6.25402	18.06758
C	-2.30771	2.19875	20.19603
H	-2.50626	1.12861	20.05452
H	-2.33000	2.38630	21.28043
H	-3.14624	2.75517	19.75845
C	2.46144	1.08865	19.01747
H	2.92098	1.00378	18.02877
H	3.25856	1.40177	19.70586
H	2.12108	0.09505	19.33788
H	-0.02594	0.72106	19.95301
C	1.54706	3.38811	18.44450
C	3.78054	2.87011	16.42716
C	5.02272	0.08282	13.28668
H	6.11168	-0.05321	13.26294
H	4.58016	-0.91310	13.44320
H	4.70741	0.41754	12.29001
C	1.78199	3.54047	15.02510
H	1.07550	3.37401	15.84609
H	2.01499	4.61495	15.02671
H	1.28577	3.30625	14.07446
C	5.84194	2.25768	17.76803
H	6.17523	3.29894	17.86085
H	5.28874	2.02701	18.68711
H	6.72542	1.60700	17.72892
H	6.26923	0.63319	15.65783

- Thermochemistry -

Temperature 298.150 Kelvin. Pressure 1.00000 Atm.

Zero-point correction= 0.517442 (Hartree/Particle)
Thermal correction to Energy= 0.549957
Thermal correction to Enthalpy= 0.550902
Thermal correction to Gibbs Free Energy= 0.449829
Sum of electronic and zero-point Energies= -2311.936086
Sum of electronic and thermal Energies= -2311.903571
Sum of electronic and thermal Enthalpies= -2311.902627

Sum of electronic and thermal Free Energies= -2312.003700

Charge = -1 Multiplicity = 3

^{5}SM
E(UB3LYP) = -2312.47726269 a.u.

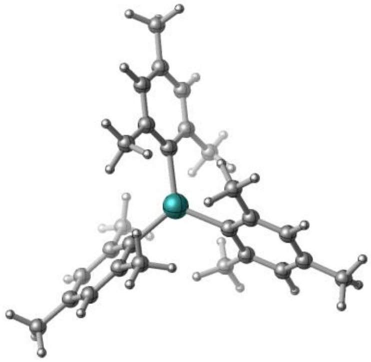

				C	5.72958	0.89888	15.30769
Fe	3.27283	3.86127	17.36701	C	5.05327	0.60336	14.11870
C	0.04969	2.43748	19.96997				
C	-1.00826	3.32653	19.75435	C	3.84174	1.25713	13.87507
C	-0.81411	4.36367	18.83568	H	3.30106	1.04408	12.95076
H	-1.63007	5.06443	18.64776	C	3.31563	2.17541	14.79368
C	0.40121	4.51017	18.15549	C	5.20780	1.81416	16.22947
C	1.26802	2.58400	19.29422	C	5.74981	6.98226	19.21240

C	6.06466	7.85296	18.16462	H	-2.51159	2.14678	20.78335
C	5.55793	7.55975	16.89388	H	-2.29836	3.76725	21.45170
H	5.78613	8.23180	16.06437	H	-3.16003	3.55641	19.92535
C	4.76492	6.42703	16.67336	C	2.39736	1.60754	19.56471
C	4.95634	5.84732	18.99899	H	2.79406	1.20241	18.62469
C	4.65003	4.91842	20.15872	H	3.23626	2.11037	20.06698
H	4.92770	5.34973	21.12905	H	2.08841	0.76639	20.19882
H	3.58213	4.66747	20.18124	H	-0.08350	1.61753	20.67860
H	5.19383	3.96840	20.05222	C	1.48680	3.62563	18.36191
C	4.22645	6.14391	15.28350	C	3.98200	2.48468	16.00157
H	4.49650	5.13061	14.96215	C	5.59957	-0.41788	13.14596
H	3.12806	6.19133	15.27364	H	6.69699	-0.42389	13.14160
H	4.59535	6.85769	14.53585	H	5.27555	-1.43870	13.39862
C	6.95101	9.05751	18.39052	H	5.26153	-0.21928	12.12122
H	8.01153	8.82431	18.21260	C	2.01058	2.88267	14.48015
H	6.68886	9.88328	17.71704	H	1.35206	2.88418	15.35825
H	6.87311	9.42728	19.42059	H	2.18857	3.93602	14.21882
H	6.12928	7.19804	20.21308	H	1.47051	2.42227	13.64274
C	4.43971	5.53348	17.72107	C	5.96829	2.11275	17.50792
C	0.57333	5.64172	17.15944	H	6.17242	3.18694	17.59679
H	1.46695	6.23353	17.39631	H	5.37258	1.83539	18.38932
H	0.71939	5.24925	16.14295	H	6.92130	1.57156	17.56813
H	-0.29056	6.31848	17.13255	H	6.67977	0.40239	15.51412
C	-2.30902	3.19111	20.51414				

- Thermochemistry -

Temperature 298.150 Kelvin. Pressure 1.00000 Atm.

Zero-point correction= 0.516237 (Hartree/Particle)
Thermal correction to Energy= 0.549531
Thermal correction to Enthalpy= 0.550475
Thermal correction to Gibbs Free Energy= 0.444735
Sum of electronic and zero-point Energies= -2311.961026
Sum of electronic and thermal Energies= -2311.927732
Sum of electronic and thermal Enthalpies= -2311.926788

Sum of electronic and thermal Free Energies= -2312.032527

Charge = -1 Multiplicity = 5

^{1}Int1
E(UB3LYP) = -2868.05423433 a.u.

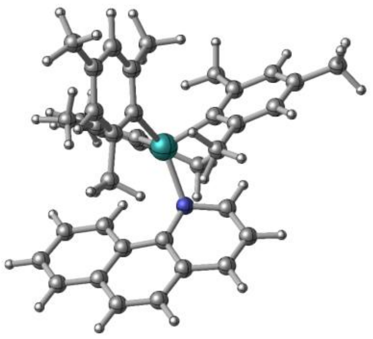

Fe	3.52874	3.51806	18.26737
C	-0.66136	2.74410	18.44026
C	-1.28547	3.97340	18.64625
C	-0.45934	5.08564	18.78783
H	-0.91327	6.05370	19.00083
C	0.93902	5.00811	18.68086
C	0.72955	2.62744	18.33517
C	6.11074	1.73286	15.47636
C	5.34983	1.22259	14.41946
C	3.98681	1.47982	14.44711
H	3.36214	1.12517	13.62570
C	3.37025	2.19408	15.49360
C	5.53637	2.44351	16.53084
C	5.83570	7.07916	18.34398
C	5.65755	7.71692	17.11741
C	4.73418	7.15327	16.23632
H	4.53590	7.65351	15.28741

C	4.04210	5.97262	16.53591	C	5.99375	0.43080	13.30531
C	5.15257	5.90004	18.67229	H	6.86932	0.95032	12.89355
C	5.37253	5.33793	20.06008	H	6.34350	-0.55126	13.65457
H	5.82413	4.34175	20.04933	H	5.29216	0.25474	12.48146
C	3.01135	5.50864	15.53197	C	1.88331	2.42976	15.29730
H	3.40497	4.71129	14.89464	H	1.32496	1.48405	15.29230
H	2.13150	5.11013	16.04084	H	1.45439	3.06074	16.06895
H	2.69094	6.33401	14.88387	H	1.70338	2.91288	14.32781
C	6.43218	8.96287	16.75573	C	6.49119	2.98726	17.57583
H	7.36376	8.72153	16.22302	H	6.60878	4.06968	17.46912
H	5.85053	9.62267	16.09990	H	6.15587	2.79341	18.59706
H	6.71172	9.53656	17.64793	H	7.47907	2.52287	17.47978
H	6.51586	7.51581	19.07611	H	7.18983	1.57521	15.47603
C	4.25746	5.28227	17.75674	C	2.17274	4.98963	23.47896
C	1.68823	6.29566	18.96262	C	3.42311	4.54590	23.86247
H	2.51739	6.13183	19.65372	C	4.15278	3.62136	23.07681
H	2.11528	6.73819	18.05966	C	3.59319	3.16077	21.83280
H	1.01702	7.03304	19.41920	C	2.281188	3.590981	21.502676
C	-2.79008	4.09229	18.71195	C	1.590977	4.486051	22.291829
H	-3.23016	4.22077	17.71227	C	5.420725	3.124546	23.488059
H	-3.24607	3.19579	19.14994	C	4.353624	2.300405	20.974169
H	-3.09934	4.95566	19.31338	C	5.558278	1.725168	21.480658
C	1.28048	1.22160	18.23767	C	6.072781	2.186798	22.735043
H	0.50410	0.51189	17.92776	C	6.203253	0.702438	20.744301
H	1.67327	0.90359	19.20910	H	7.111491	0.245723	21.128072
H	-1.27151	1.84301	18.37244	C	5.575316	0.242761	19.574421
C	1.57947	3.76988	18.39524	C	4.474542	0.905425	19.084996
C	4.12397	2.66502	16.59541	H	5.849518	3.482234	24.422602

H	1.631292	5.705398	24.093393	H	4.018999	0.588851	18.156926
H	3.871117	4.899712	24.790162	N	3.936484	2.052572	19.654577
H	0.59417	4.798124	21.990476	H	2.107859	1.145867	17.532488
H	7.023466	1.776422	23.07206	H	6.028148	5.984917	20.653706
H	5.956102	-0.619131	19.031958	H	4.431055	5.244689	20.609463

- Thermochemistry -

Temperature 298.150 Kelvin. Pressure 1.00000 Atm.

Zero-point correction= 0.704418 (Hartree/Particle)
Thermal correction to Energy= 0.746243
Thermal correction to Enthalpy= 0.747187
Thermal correction to Gibbs Free Energy= 0.630544
Sum of electronic and zero-point Energies= -2867.349816
Sum of electronic and thermal Energies= -2867.307992
Sum of electronic and thermal Enthalpies= -2867.307047
Sum of electronic and thermal Free Energies= -2867.423691

Charge = -1 Multiplicity = 1

^{3}Int1

E(UB3LYP) = -2868.07417640 a.u.

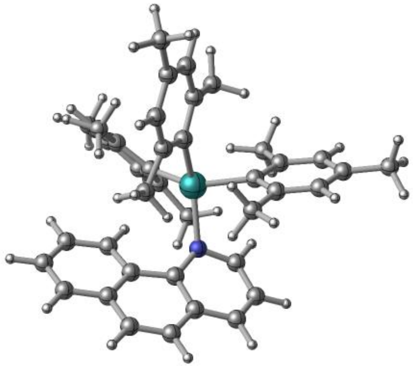

Fe	3.35683	3.44105	18.24217
C	-0.98119	3.15655	18.43769
C	-1.50090	4.44514	18.54361
C	-0.59015	5.47866	18.75828
H	-0.96965	6.49075	18.90673
C	0.79586	5.26132	18.79902
C	0.39857	2.91083	18.48793
C	6.60525	1.82456	15.75973
C	5.95749	1.27424	14.64928
C	4.57219	1.37989	14.60535
H	4.03266	0.94679	13.76103
C	3.83931	2.02102	15.62096
C	5.90168	2.47092	16.77902
C	5.46144	7.14601	18.17388
C	5.16478	7.75977	16.95789
C	4.27103	7.10270	16.11042
H	3.99203	7.57247	15.16655
C	3.71530	5.86014	16.43836
C	4.91048	5.90907	18.53895
C	5.20273	5.41074	19.93807
H	5.48988	4.35924	19.96727
C	2.72986	5.25141	15.47208
H	3.18478	4.41437	14.93386
H	1.85583	4.86597	16.00605
H	2.38586	5.98507	14.73300
C	5.79379	9.07675	16.56608
H	6.71432	8.92941	15.98238
H	5.11582	9.67852	15.94803
H	6.06319	9.67203	17.44706
H	6.12556	7.65263	18.87481
C	4.03913	5.21870	17.65739
C	1.64601	6.47492	19.12090
H	2.46587	6.21191	19.79051
H	2.09457	6.91941	18.22756
H	1.04277	7.24751	19.61485
C	-2.98498	4.70970	18.43919
H	-3.28806	4.91758	17.40242

H	-3.57311	3.84798	18.77856	C	3.79814	3.02887	21.73613
H	-3.28261	5.57817	19.03988	C	2.534787	3.641672	21.540131
C	0.82142	1.45546	18.53187	C	2.048477	4.595474	22.409434
H	-0.02550	0.78634	18.33686	C	5.744123	2.693484	23.218432
H	1.23815	1.20816	19.51430	C	4.345544	2.071794	20.810812
H	-1.66759	2.31487	18.33528	C	5.480903	1.306171	21.220352
C	1.35214	3.96568	18.60311	C	6.177358	1.677234	22.417994
C	4.48623	2.59895	16.74119	C	5.881949	0.193385	20.451614
C	6.74021	0.60993	13.54006	H	6.740492	-0.398623	20.756711
H	7.23752	1.34889	12.89523	C	5.102999	-0.149548	19.337131
H	7.52688	-0.04459	13.93755	C	4.090995	0.691417	18.931288
H	6.09111	0.00239	12.89818	H	6.3009	2.973326	24.111002
C	2.32876	1.98948	15.44379	H	2.4235	5.751729	24.213216
H	1.93965	0.97231	15.59065	H	4.593419	4.637528	24.671319
H	1.80364	2.64388	16.13828	H	1.080269	5.047601	22.209197
H	2.04342	2.29224	14.42793	H	7.079036	1.121845	22.670453
C	6.69424	3.01484	17.94220	H	5.307657	-1.045743	18.758134
H	6.65520	4.10853	17.96089	H	3.547384	0.477676	18.020659
H	6.28898	2.65228	18.89127	N	3.773147	1.883611	19.543165
H	7.74651	2.71064	17.89302	H	1.600578	1.216206	17.807714
H	7.69056	1.74331	15.83290	H	6.010609	5.986086	20.405008
C	2.80133	4.98617	23.53947	H	4.320749	5.503397	20.581435
C	4.01294	4.37159	23.78905	H	1.951526	3.364117	20.674584
C	4.52754	3.37700	22.92358				

- Thermochemistry -

Temperature 298.150 Kelvin. Pressure 1.00000 Atm.

Zero-point correction= 0.704141 (Hartree/Particle)
Thermal correction to Energy= 0.746059
Thermal correction to Enthalpy= 0.747003
Thermal correction to Gibbs Free Energy= 0.629156
Sum of electronic and zero-point Energies= -2867.370036

Sum of electronic and thermal Energies= -2867.328117
Sum of electronic and thermal Enthalpies= -2867.327173
Sum of electronic and thermal Free Energies= -2867.445021

Charge = -1 Multiplicity = 3

^{5}Int1

E(UB3LYP) = -2868.09500056 a.u.

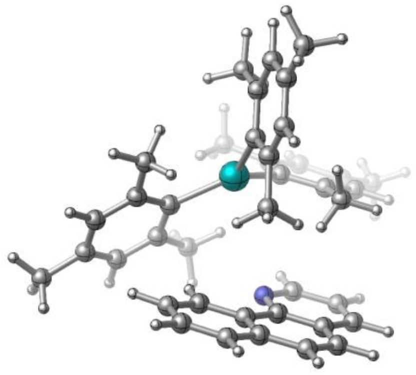

Fe	3.20826	4.18643	17.49890
C	-0.71681	2.99375	19.18626
C	-1.23041	4.15462	19.76095
C	-0.50365	5.33681	19.58273
H	-0.89464	6.26512	20.00373
C	0.70929	5.35337	18.88280
C	0.49666	2.99477	18.47752
C	5.90806	1.10915	15.89003
C	5.20445	0.49860	14.84351
C	3.94950	1.01322	14.51680
H	3.39249	0.56863	13.68963
C	3.39338	2.08786	15.23018
C	5.36071	2.17775	16.60746

C	5.93070	7.44739	18.74259	H	4.37346	4.73370	24.57062
C	6.10096	8.20017	17.57759	H	0.85143	4.78190	22.08237
C	5.42505	7.78168	16.42605	H	7.22653	1.45367	22.62446
H	5.53387	8.36119	15.50695	H	5.75463	-0.79761	18.61306
C	4.61013	6.64380	16.44104	H	3.50164	0.19932	18.22100
C	5.11839	6.30432	18.75999	N	3.52822	1.38749	19.89101
H	6.44072	7.76242	19.65536	H	1.80091	2.99722	20.68668
C	4.42619	5.86300	17.60822	C	1.43016	6.68331	18.76320
H	-1.26983	2.05861	19.29529	H	2.32100	6.71634	19.40054
C	1.27141	4.17296	18.31716	H	1.79395	6.85654	17.74600
C	4.06361	2.68060	16.32447	H	0.78085	7.52172	19.04747
H	6.90370	0.74056	16.14491	C	0.94096	1.66284	17.90212
C	2.55212	4.86357	23.43478	H	1.95546	1.71935	17.50876
C	3.80228	4.34980	23.72755	H	0.92030	0.87456	18.66516
C	4.36909	3.32984	22.92864	H	0.28226	1.34571	17.08060
C	3.62783	2.83175	21.81881	C	-2.50789	4.13704	20.56961
C	2.34839	3.36128	21.54597	H	-3.07744	5.06685	20.44511
C	1.82167	4.36646	22.33734	H	-3.15753	3.30274	20.27805
C	5.67637	2.80180	23.20095	H	-2.30646	4.02724	21.64562
C	4.22591	1.81726	20.97027	C	2.05535	2.64156	14.77594
C	5.52677	1.32410	21.27497	H	1.37957	1.85767	14.40908
C	6.23137	1.83954	22.41248	H	1.556675	3.171025	15.594028
C	6.07430	0.34753	20.41389	H	2.189613	3.364449	13.957943
H	7.07045	-0.04165	20.61578	C	6.175423	2.822692	17.710146
C	5.35219	-0.07829	19.31891	H	7.142431	2.326558	17.859067
C	4.07783	0.48276	19.09776	H	6.348652	3.882567	17.49352
H	6.22476	3.19740	24.05342	H	5.637413	2.790554	18.664008
H	2.13526	5.65916	24.04796	C	5.783622	-0.690192	14.109655

H	6.863302	-0.577333	13.946623	H	5.616087	4.620315	20.038631
H	5.643262	-1.625902	14.671372	H	5.263629	6.101445	20.934352
H	5.309515	-0.828486	13.130403	H	3.954673	5.169385	20.188797
C	3.88137	6.227078	15.176825	C	7.006681	9.411184	17.553224
H	4.093672	6.887811	14.326344	H	6.641471	10.174727	16.854414
H	4.151962	5.202031	14.892323	H	7.085659	9.874773	18.54461
H	2.792568	6.228541	15.334202	H	8.028807	9.15288	17.237513
C	4.983097	5.518143	20.048078				

- Thermochemistry -

Temperature 298.150 Kelvin. Pressure 1.00000 Atm.

Zero-point correction= 0.701792 (Hartree/Particle)
Thermal correction to Energy= 0.745636
Thermal correction to Enthalpy= 0.746580
Thermal correction to Gibbs Free Energy= 0.619091
Sum of electronic and zero-point Energies= -2867.393209
Sum of electronic and thermal Energies= -2867.349365
Sum of electronic and thermal Enthalpies= -2867.348420
Sum of electronic and thermal Free Energies= -2867.475910

Charge = -1 Multiplicity = 5

$^{1}TS_{1-2}$
E(UB3LYP) = -2868.02847934 a.u.

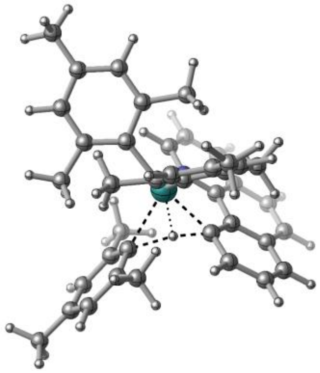

Fe	3.70445	3.73233	18.67025
C	-0.73058	2.52632	19.01029
C	-1.46230	3.66957	18.67495
C	-0.75771	4.86353	18.56691
H	-1.30054	5.78378	18.34456
C	0.63422	4.92597	18.74969
C	0.65650	2.56063	19.18034
C	5.86913	1.99324	15.34911
C	4.93487	1.56417	14.40775
C	3.59618	1.79638	14.70816
H	2.83494	1.46770	13.99859
C	3.18654	2.43606	15.89150
C	5.48976	2.63772	16.53433
C	6.34817	7.26446	18.59808
C	5.89525	8.04975	17.54055
C	4.84739	7.53495	16.77701
H	4.48995	8.10498	15.91743
C	4.25217	6.30049	17.06907
C	5.75691	6.03223	18.91970
C	6.40936	5.28339	20.07078
H	6.19245	4.21671	20.03535
C	3.18444	5.82540	16.10535
H	3.61612	5.16428	15.34560
H	2.41508	5.24384	16.61005
H	2.70014	6.66693	15.59224
C	6.50164	9.40118	17.23937
H	7.53938	9.46381	17.59046
H	6.49882	9.61391	16.16258
H	5.94781	10.21759	17.72714
H	7.19240	7.61704	19.19426
C	4.65247	5.51163	18.18610
C	1.24307	6.31354	18.70140
H	2.33079	6.28805	18.70337
H	0.92898	6.85826	17.80214
H	0.91020	6.90560	19.56559

C	-2.95307	3.60117	18.43741
H	-3.18543	3.21599	17.43401
H	-3.44657	2.93456	19.15643
H	-3.41927	4.59015	18.52106
C	1.33785	1.25858	19.53483
H	0.60751	0.45448	19.68585
H	1.93197	1.34945	20.44872
H	-1.25889	1.58038	19.13706
C	1.39618	3.75999	18.99438
C	4.12153	2.89618	16.86622
C	5.35882	0.91054	13.11269
H	4.56591	0.26989	12.70663
H	5.60002	1.65369	12.33795
H	6.25474	0.29057	13.24760
C	1.68541	2.57761	16.05212
H	1.27721	1.83656	16.74717
H	1.39345	3.54800	16.45043
H	1.17551	2.43529	15.09076
C	6.62610	3.04568	17.45133
H	6.72772	4.13309	17.48780
H	6.46341	2.71367	18.48051
H	7.58064	2.62189	17.11419
H	6.92977	1.82491	15.15472
C	3.59736	5.93567	22.67417
C	4.09613	4.94697	23.51245
C	4.35027	3.66097	22.99785
C	4.08988	3.42020	21.61846
C	3.554731	4.414603	20.750716
C	3.311373	5.665763	21.326426
C	4.877405	2.593879	23.802444
C	4.450194	2.159452	21.055154
C	4.966553	1.114315	21.869042
C	5.158286	1.370611	23.2689
C	5.28367	-0.107963	21.24924
H	5.676659	-0.929428	21.844696
C	5.096886	-0.237093	19.880858
C	4.612062	0.849691	19.146598
H	5.05472	2.7832	24.859738
H	3.420136	6.934692	23.068407
H	4.295132	5.152892	24.562413
H	2.916945	6.463932	20.709839
H	5.554981	0.570584	23.890718
H	5.331558	-1.162889	19.364209
H	4.48121	0.793306	18.072713
N	4.296147	2.030891	19.698378
H	2.025216	0.944458	18.742704
H	7.500642	5.404181	20.04194
H	6.064209	5.648752	21.045829
H	2.632048	4.058268	19.890425

- Thermochemistry -

Temperature 298.150 Kelvin. Pressure 1.00000 Atm.

Zero-point correction= 0.701980 (Hartree/Particle)
Thermal correction to Energy= 0.742931
Thermal correction to Enthalpy= 0.743875
Thermal correction to Gibbs Free Energy= 0.629402
Sum of electronic and zero-point Energies= -2867.326500
Sum of electronic and thermal Energies= -2867.285549
Sum of electronic and thermal Enthalpies= -2867.284604
Sum of electronic and thermal Free Energies= -2867.399078

Charge = -1 Multiplicity = 1

$^{3}TS_{1\text{-}2}$
E(UB3LYP) = -2868.04819793 a.u.

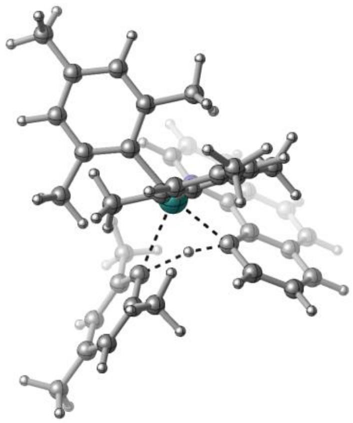

Fe	3.69673	3.74100	18.64774
C	-0.72450	2.45844	18.97437
C	-1.48087	3.60191	18.69778
C	-0.79805	4.81123	18.62413
H	-1.35981	5.72914	18.44216
C	0.59548	4.89027	18.78905
C	0.66388	2.51136	19.12621
C	5.85374	2.05789	15.29437
C	4.91275	1.63771	14.35504
C	3.57772	1.88814	14.65369
H	2.81322	1.58045	13.93856
C	3.17393	2.52493	15.84126
C	5.48378	2.69651	16.48504

C	6.35602	7.26686	18.68644	H	1.97375	1.26750	20.32602
C	5.89098	8.09041	17.66346	H	-1.23461	1.49861	19.06834
C	4.83476	7.60417	16.89352	C	1.38098	3.73053	18.98378
H	4.46785	8.20507	16.05954	C	4.11722	2.95876	16.81743
C	4.24238	6.35914	17.14583	C	5.32652	0.92448	13.08840
C	5.76897	6.02373	18.96971	H	6.24069	1.35709	12.66157
C	6.42443	5.23728	20.09223	H	5.53253	-0.14174	13.26617
H	6.21212	4.17191	20.01895	H	4.54221	0.97799	12.32352
C	3.16959	5.91772	16.17202	C	1.67469	2.69302	15.99317
H	3.59587	5.27684	15.39211	H	1.23961	1.91426	16.62876
H	2.39624	5.32744	16.66009	H	1.39964	3.63874	16.45509
H	2.68920	6.77655	15.68506	H	1.17709	2.62990	15.01712
C	6.49411	9.45235	17.40639	C	6.62485	3.10028	17.39651
H	7.54293	9.49492	17.72613	H	6.72439	4.18755	17.44147
H	6.45736	9.71532	16.34151	H	6.46716	2.75966	18.42300
H	5.96042	10.24639	17.95014	H	7.57721	2.68043	17.04923
H	7.20562	7.59832	19.28683	H	6.91274	1.88945	15.09266
C	4.65699	5.53245	18.22844	C	3.60079	5.87691	22.67760
C	1.18412	6.28763	18.77078	C	4.10951	4.88125	23.50602
H	2.27210	6.27638	18.75163	C	4.37097	3.60454	22.97699
H	0.84521	6.85424	17.89404	C	4.11236	3.37603	21.59446
H	0.86221	6.85077	19.65808	C	3.562174	4.375762	20.735855
C	-2.97422	3.51657	18.48252	C	3.310773	5.620505	21.330928
H	-3.44777	2.83471	19.20084	C	4.905986	2.530279	23.767711
H	-3.45189	4.49813	18.58621	C	4.485482	2.130849	21.017000
H	-3.21793	3.14040	17.47833	C	5.00718	1.075372	21.815541
C	1.37845	1.20963	19.41015	C	5.194823	1.315425	23.219275
H	0.67009	0.37937	19.51911	C	5.332061	-0.134152	21.178218

H	5.729182	-0.9629	21.760658	H	5.389601	-1.158908	19.274672
C	5.147858	-0.242527	19.804837	H	4.537145	0.811233	18.011721
C	4.66156	0.852519	19.087254	N	4.338019	2.025241	19.656292
H	5.08144	2.707418	24.827488	H	2.071808	0.956183	18.601191
H	3.419523	6.87086	23.082686	H	7.514869	5.366000	20.069957
H	4.308715	5.077772	24.557727	H	6.074312	5.566484	21.078186
H	2.907246	6.422821	20.726267	H	2.633165	4.022122	19.912074
H	5.595955	0.510372	23.831863				

- Thermochemistry -

Temperature 298.150 Kelvin. Pressure 1.00000 Atm.

Zero-point correction= 0.700789 (Hartree/Particle)
Thermal correction to Energy= 0.741904
Thermal correction to Enthalpy= 0.742849
Thermal correction to Gibbs Free Energy= 0.626609
Sum of electronic and zero-point Energies= -2867.347409
Sum of electronic and thermal Energies= -2867.306294
Sum of electronic and thermal Enthalpies= -2867.305349
Sum of electronic and thermal Free Energies= -2867.421589

Charge = -1 Multiplicity = 3

^{5}TS$_{1-2}$
E(UB3LYP) = -2868.04860345 a.u.

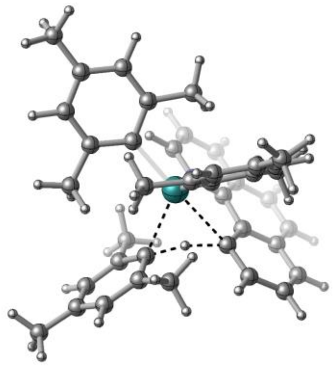

Fe	3.69927	3.62502	18.52898
C	-0.74937	2.65845	18.85489
C	-1.38421	3.79917	18.35110
C	-0.65310	4.98609	18.31865
H	-1.13792	5.90046	17.97308
C	0.68523	5.03737	18.73602
C	0.58988	2.68321	19.25175
C	5.72974	1.83816	15.03623
C	4.74492	1.46505	14.12084
C	3.41399	1.63968	14.50306
H	2.62345	1.36200	13.80354
C	3.06960	2.16131	15.75903
C	5.40262	2.36837	16.29284
C	6.47559	7.12158	18.59161
C	6.08416	7.88719	17.49302
C	5.02369	7.40945	16.72040
H	4.71672	7.97248	15.83706
C	4.35770	6.22002	17.04460
C	5.81223	5.93396	18.93664
H	7.32084	7.45742	19.19550
C	4.70905	5.45038	18.18731
H	-1.31484	1.72876	18.93216
C	1.36311	3.86554	19.13986
C	4.05320	2.55101	16.70562
H	6.77939	1.72205	14.76048
C	3.37205	5.73423	23.11753
C	4.01683	4.75338	23.85422
C	4.35092	3.52613	23.24621
C	4.03893	3.33756	21.86143
C	3.34574	4.31863	21.10544
C	3.02054	5.49725	21.77564
C	4.98835	2.47596	23.98724
C	4.47396	2.11519	21.22819
C	5.07613	1.07715	22.00190
C	5.31814	1.29327	23.39904

C	5.42692	-0.12414	21.35817
H	5.88076	-0.92717	21.93587
C	5.18952	-0.26174	20.00276
C	4.63610	0.81749	19.30510
H	5.19936	2.64126	25.04285
H	3.12322	6.68526	23.58754
H	4.26525	4.91205	24.90231
H	2.47848	6.27709	21.24495
H	5.78801	0.49545	23.97085
H	5.43935	-1.17333	19.46852
H	4.46683	0.77105	18.23416
N	4.30163	1.98040	19.87663
H	2.44231	4.02664	19.99082
C	1.35190	6.39775	18.78672
H	2.43295	6.32039	18.91189
H	1.16238	6.97750	17.87488
H	0.95155	6.98328	19.62651
C	1.20733	1.40968	19.78422
H	1.90256	0.97014	19.05923
H	1.78460	1.60129	20.69365
H	0.44254	0.65738	20.01243
C	-2.81123	3.73992	17.85715
H	-2.86184	3.38111	16.81908
H	-3.41954	3.05567	18.46198
H	-3.28792	4.72710	17.88202
C	1.59440	2.29650	16.07584
H	0.97048	2.12204	15.19000
H	1.282346	1.579351	16.843331
H	1.351067	3.284357	16.475332
C	6.550935	2.775202	17.197341
H	7.519559	2.451705	16.795661
H	6.575811	3.862091	17.325186
H	6.448336	2.350849	18.202338
C	5.105173	0.867239	12.779532
H	6.06869	1.245608	12.415971
H	5.188978	-0.228862	12.828985
H	4.346624	1.095812	12.020533
C	3.276444	5.744799	16.092653
H	2.886435	6.565344	15.476239
H	3.66452	4.968607	15.423288
H	2.43345	5.292802	16.621939
C	6.339919	5.173717	20.13793
H	7.414495	5.348519	20.281914
H	5.826846	5.471489	21.059687
H	6.180867	4.098744	20.023504
C	6.770789	9.192296	17.15932
H	6.265717	10.050357	17.627614
H	7.810184	9.201669	17.510535
H	6.779809	9.378516	16.077895

- Thermochemistry -

Temperature 298.150 Kelvin. Pressure 1.00000 Atm.

Zero-point correction= 0.698103 (Hartree/Particle)
Thermal correction to Energy= 0.740582
Thermal correction to Enthalpy= 0.741526
Thermal correction to Gibbs Free Energy= 0.620573
Sum of electronic and zero-point Energies= -2867.350500
Sum of electronic and thermal Energies= -2867.308022
Sum of electronic and thermal Enthalpies= -2867.307078
Sum of electronic and thermal Free Energies= -2867.428030

Charge = -1 Multiplicity = 5

[1]Int2
E(UB3LYP) = -2517.85907337 a.u.

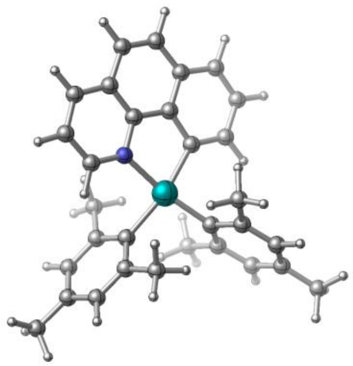

Fe	0.05306	0.01253	-0.06199
N	-1.19330	-1.54802	-0.41684
C	1.32626	-1.47412	0.37426
C	2.65012	-1.49993	0.83220
H	3.18004	-0.55633	0.95168
C	3.32946	-2.70166	1.12358
H	4.36167	-2.66254	1.47177
C	2.71717	-3.93958	0.96439
H	3.25371	-4.86196	1.18219
C	1.38409	-3.99688	0.50818
C	0.66296	-5.22128	0.29003
H	1.16848	-6.16439	0.49471
C	-0.62330	-5.22582	-0.17313
H	-1.14825	-6.16439	-0.34149
C	-1.31486	-3.99669	-0.45065
C	-2.63247	-3.91275	-0.94294
H	-3.19619	-4.82331	-1.13751

C	-3.19002	-2.66379	-1.17431	H	5.26782	4.40582	0.69696
H	-4.20296	-2.56150	-1.55267	C	0.97794	2.18511	2.26323
C	-2.44467	-1.50916	-0.89240	H	-0.07621	2.38145	2.04095
H	-2.86130	-0.51508	-1.01356	H	1.02647	1.18642	2.71868
C	-0.62158	-2.78161	-0.21911	H	1.31898	2.91035	3.01359
C	0.72852	-2.76269	0.24007	C	2.13956	0.46380	-2.34723
C	-1.50853	1.30809	0.02451	H	2.34966	-0.56424	-2.02504
C	-2.38097	1.24725	1.14555	H	1.09874	0.47620	-2.69644
C	-3.49783	2.08599	1.26876	H	2.78319	0.69222	-3.20692
H	-4.12830	2.01239	2.15732	H	-3.22892	3.79119	-1.63421
C	-3.82708	3.01425	0.27919	C	-1.02626	2.36851	-2.22380
C	-2.99599	3.07540	-0.84318	H	0.01745	2.57416	-1.96595
C	-1.87121	2.25015	-0.97266	H	-1.03031	1.42649	-2.79028
C	1.48093	1.36738	-0.07441	H	-1.38557	3.15907	-2.89571
C	2.34773	1.42986	-1.19815	C	-2.12698	0.22833	2.24235
C	1.80625	2.23418	0.99838	H	-2.34041	-0.79016	1.89315
C	3.41877	2.33018	-1.25941	H	-1.07835	0.23198	2.56022
C	2.88645	3.12255	0.92782	H	-2.74704	0.41413	3.12914
C	3.70446	3.19896	-0.20326	C	-5.05137	3.89385	0.39820
H	4.05249	2.34856	-2.14854	H	-4.88326	4.88225	-0.04881
C	4.83441	4.19970	-0.29041	H	-5.92531	3.45930	-0.11122
H	5.64260	3.83996	-0.94044	H	-5.33616	4.04643	1.44686
H	4.50189	5.16611	-0.69985	H	3.097247	3.774701	1.777852

- Thermochemistry -

Temperature 298.150 Kelvin. Pressure 1.00000 Atm.

Zero-point correction= 0.517735 (Hartree/Particle)
Thermal correction to Energy= 0.549291
Thermal correction to Enthalpy= 0.550235
Thermal correction to Gibbs Free Energy= 0.454596
Sum of electronic and zero-point Energies= -2517.341338
Sum of electronic and thermal Energies= -2517.309782
Sum of electronic and thermal Enthalpies= -2517.308838
Sum of electronic and thermal Free Energies= -2517.404477
Charge = -1 Multiplicity = 1

^{3}Int2

E(UB3LYP) = -2517.87727312 a.u.

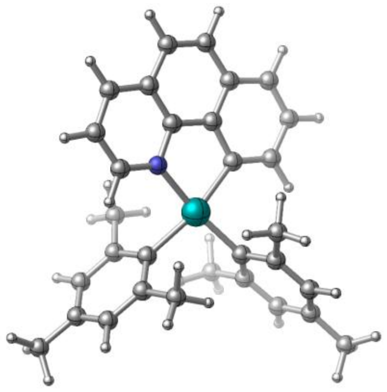

				C	1.36889	-4.03391	0.56317
Fe	0.05697	-0.03786	-0.13600	C	0.62547	-5.24999	0.40723
N	-1.15887	-1.56275	-0.47849	H	1.10973	-6.19400	0.65474
C	1.35481	-1.52593	0.33722	C	-0.66556	-5.24223	-0.04999
C	2.67942	-1.54498	0.77106	H	-1.21086	-6.17685	-0.17181
H	3.22355	-0.60476	0.84811	C	-1.33637	-4.01808	-0.38664
C	3.34729	-2.74828	1.10348	C	-2.65664	-3.94210	-0.89404
H	4.38232	-2.71031	1.44295	H	-3.23833	-4.84913	-1.04160
C	2.71115	-3.97582	1.00422	C	-3.17475	-2.68788	-1.20283
H	3.23262	-4.89795	1.25803	H	-4.18075	-2.58220	-1.60062

C	-2.41337	-1.53704	-0.98942
H	-2.81108	-0.54739	-1.18039
C	-0.62232	-2.81114	-0.21055
C	0.72821	-2.80364	0.24709
C	-1.50448	1.26434	-0.03921
C	-2.34673	1.21619	1.10223
C	-3.41524	2.10866	1.26812
H	-4.03151	2.04531	2.16687
C	-3.71757	3.07410	0.30569
C	-2.91484	3.11682	-0.83741
C	-1.83819	2.23763	-1.01238
C	1.47362	1.35394	-0.08150
C	2.32306	1.46636	-1.21022
C	1.76103	2.20854	1.00793
C	3.35273	2.41533	-1.26190
C	2.79911	3.14623	0.94486
C	3.60183	3.27797	-0.19207
H	3.98301	2.47509	-2.15093
C	4.68621	4.32892	-0.26721
H	5.49577	4.02506	-0.94281
H	4.30101	5.28999	-0.63991
H	5.12808	4.52368	0.71834
C	0.94751	2.09335	2.27607
H	-0.10574	2.32383	2.08376
H	0.98330	1.06400	2.65655
H	1.31259	2.76089	3.06693
C	2.15883	0.49935	-2.36710
H	2.36241	-0.52621	-2.03420

H	1.13577	0.50649	-2.76313
H	2.83542	0.73086	-3.19945
H	-3.13313	3.85679	-1.60969
C	-1.02140	2.33330	-2.28290
H	0.01993	2.58761	-2.05801
H	-1.00388	1.36641	-2.80441
H	-1.42026	3.08273	-2.97857
C	-2.12829	0.15175	2.16247
H	-2.36472	-0.84407	1.76846
H	-1.08616	0.11468	2.49709
H	-2.75465	0.32291	3.04734
C	-4.89299	4.01068	0.47323
H	-4.69656	4.98961	0.01764
H	-5.80521	3.61599	0.00054
H	-5.12742	4.17604	1.53221
H	2.989858	3.790415	1.805001

- Thermochemistry -

Temperature 298.150 Kelvin. Pressure 1.00000 Atm.

Zero-point correction= 0.517079 (Hartree/Particle)
Thermal correction to Energy= 0.548711
Thermal correction to Enthalpy= 0.549656
Thermal correction to Gibbs Free Energy= 0.452673
Sum of electronic and zero-point Energies= -2517.360194
Sum of electronic and thermal Energies= -2517.328562
Sum of electronic and thermal Enthalpies= -2517.327617
Sum of electronic and thermal Free Energies= -2517.424600

Charge = -1 Multiplicity = 3

^{5}Int2
E(UB3LYP) = -2517.87683661 a.u.

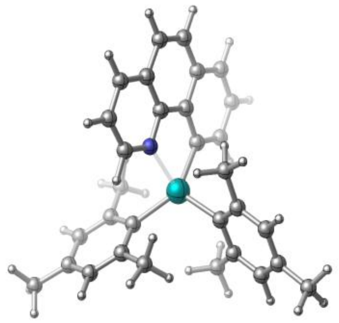

Fe	0.14451	0.28894	0.45488
N	-0.77252	-1.22320	-0.79985
C	0.85660	-1.45476	1.38850
C	1.69603	-1.68519	2.48418
H	2.00716	-0.84056	3.10029
C	2.17318	-2.96468	2.83722
H	2.82202	-3.08043	3.70553
C	1.82675	-4.08260	2.09311
H	2.19137	-5.07231	2.36454
C	0.98453	-3.93670	0.97097
C	0.56821	-5.05622	0.17220
H	0.93629	-6.04607	0.43998
C	-0.27921	-4.90872	-0.88725
H	-0.59406	-5.77066	-1.47276

C	-0.77970	-3.61373	-1.25127	H	0.38731	3.05150	1.52275
C	-1.67849	-3.39326	-2.31463	H	1.58152	2.17453	2.46249
H	-2.01990	-4.23732	-2.91114	H	1.84445	3.89376	2.10726
C	-2.12550	-2.10748	-2.57763	C	2.11170	-0.19504	-2.21106
H	-2.82991	-1.90675	-3.37965	H	2.07505	-1.06653	-1.54687
C	-1.64377	-1.05013	-1.79193	H	1.10967	-0.10470	-2.65020
H	-1.96850	-0.02579	-1.95276	H	2.81821	-0.40922	-3.02387
C	-0.35522	-2.48623	-0.49515	H	-3.53435	3.83686	-0.81755
C	0.52424	-2.62647	0.64044	C	-1.08205	2.85057	-1.32237
C	-1.64581	1.36946	0.66718	H	-0.19972	3.35185	-0.90902
C	-2.59047	1.02908	1.66657	H	-0.68617	1.99864	-1.88732
C	-3.82626	1.67940	1.77131	H	-1.56885	3.53649	-2.02824
H	-4.52634	1.38969	2.55757	C	-2.25808	-0.08126	2.64522
C	-4.18819	2.69622	0.88175	H	-2.02316	-1.01081	2.11292
C	-3.27305	3.04442	-0.11339	H	-1.35959	0.16041	3.22615
C	-2.02993	2.40062	-0.22414	H	-3.07956	-0.28001	3.34627
C	1.76100	1.38329	-0.26796	C	-5.54052	3.36739	0.97423
C	2.48527	1.05427	-1.43700	H	-5.52719	4.36411	0.51607
C	2.18099	2.55027	0.41480	H	-6.322196	2.787303	0.460948
C	3.53878	1.85323	-1.90307	H	-5.863699	3.482138	2.016931
C	3.23290	3.34629	-0.05775	H	3.525476	4.241315	0.495108
C	3.92411	3.01427	-1.22686				
H	4.07507	1.56711	-2.81024				
C	5.03279	3.89642	-1.75623				
H	5.77135	3.31766	-2.32537				
H	4.64959	4.67735	-2.43043				
H	5.56315	4.40930	-0.94372				
C	1.46629	2.95006	1.69261				

- Thermochemistry –

Temperature 298.150 Kelvin. Pressure 1.00000 Atm.

Zero-point correction= 0.515363 (Hartree/Particle)
Thermal correction to Energy= 0.548271
Thermal correction to Enthalpy= 0.549215
Thermal correction to Gibbs Free Energy= 0.445521
Sum of electronic and zero-point Energies= -2517.361474
Sum of electronic and thermal Energies= -2517.328566
Sum of electronic and thermal Enthalpies= -2517.327621
Sum of electronic and thermal Free Energies= -2517.431316

Charge = -1 Multiplicity = 5

10. References

1. S. Stoll and A. Schweiger, *J Magn Reson*, 2006, **178**, 42–55.
2. *Journal*, 2021.
3. G. Sheldrick, *Acta Crystallographica Section A*, 2015, **71**, 3–8.
4. G. Sheldrick, *Acta Crystallographica Section C*, 2015, **71**, 3–8.
5. O. V. Dolomanov, L. J. Bourhis, R. J. Gildea, J. A. K. Howard and H. Puschmann, *Journal of Applied Crystallography*, 2009, **42**, 339–341.
6. C. P. Agilent and P. CrysAlis, *Yarnton, Oxfordshire, England*, 2014, **2014**.
7. M. J. Frisch, G. W. Trucks, H. B. Schlegel, G. E. Scuseria, M. A. Robb, J. R. Cheeseman, G. Scalmani, V. Barone, G. A. Petersson, H. Nakatsuji, X. Li, M. Caricato, A. V. Marenich, J. Bloino, B. G. Janesko, R. Gomperts, B. Mennucci, H. P. Hratchian, J. V. Ortiz, A. F. Izmaylov, J. L. Sonnenberg, Williams, F. Ding, F. Lipparini, F. Egidi, J. Goings, B. Peng, A. Petrone, T. Henderson, D. Ranasinghe, V. G. Zakrzewski, J. Gao, N. Rega, G. Zheng, W. Liang, M. Hada, M. Ehara, K. Toyota, R. Fukuda, J. Hasegawa, M. Ishida, T. Nakajima, Y. Honda, O. Kitao, H. Nakai, T. Vreven, K. Throssell, J. A. Montgomery Jr., J. E. Peralta, F. Ogliaro, M. J. Bearpark, J. J. Heyd, E. N. Brothers, K. N. Kudin, V. N. Staroverov, T. A. Keith, R. Kobayashi, J. Normand, K. Raghavachari, A. P. Rendell, J. C. Burant, S. S. Iyengar, J. Tomasi, M. Cossi, J. M. Millam, M. Klene, C. Adamo, R. Cammi, J. W. Ochterski, R. L. Martin, K. Morokuma, O. Farkas, J. B. Foresman and D. J. Fox, *Journal*, 2016.
8. F. Neese, F. Wennmohs, U. Becker and C. Riplinger, *J Chem Phys*, 2020, **152**.
9. A. D. Becke, *J Chem Phys*, 1993, **98**, 5648–5652.
10. C. T. Lee, W. T. Yang and R. G. Parr, *Phys Rev B*, 1988, **37**, 785–789.
11. S. H. Vosko, L. Wilk and M. Nusair, *Can J Phys*, 1980, **58**, 1200–1211.
12. P. J. Stephens, F. J. Devlin, C. F. Chabalowski and M. J. Frisch, *J Phys Chem-Us*, 1994, **98**, 11623–11627.
13. S. Grimme, J. Antony, S. Ehrlich and H. Krieg, *J Chem Phys*, 2010, **132**.
14. F. Weigend and R. Ahlrichs, *Phys Chem Chem Phys*, 2005, **7**, 3297–3305.
15. S. Miertus, E. Scrocco and J. Tomasi, *Chem Phys*, 1981, **55**, 117–129.
16. S. Miertus and J. Tomasi, *Chem Phys*, 1982, **65**, 239–245.
17. J. L. Pascualahuir, E. Silla and I. Tunon, *J Comput Chem*, 1994, **15**, 1127–1138.
18. J. M. Tao, J. P. Perdew, V. N. Staroverov and G. E. Scuseria, *Phys Rev Lett*, 2003, **91**.
19. V. N. Staroverov, G. E. Scuseria, J. M. Tao and J. P. Perdew, *J Chem Phys*, 2003, **119**, 12129–12137.
20. T. Lu and F. W. Chen, *J Comput Chem*, 2012, **33**, 580–592.
21. F. Neese, *Inorg Chim Acta*, 2002, **337**, 181–192.
22. T. Nakajima and K. Hirao, *Chem Rev*, 2012, **112**, 385–402.
23. M. Römelt, S. F. Ye and F. Neese, *Inorg Chem*, 2009, **48**, 784–785.
24. R. Bjomsson, F. Neese and S. DeBeer, *Inorg Chem*, 2017, **56**, 1470–1477.
25. R. B. Bedford, P. B. Brenner, E. Carter, P. M. Cogswell, M. F. Haddow, J. N. Harvey, D. M. Murphy, J. Nunn and C. H. Woodall, *Angew Chem Int Ed Engl*, 2014, **53**, 1804–1808.
26. S. Grimme, *Journal of computational chemistry*, 2004, **25**, 1463–1473.
27. G. Petersson, T. G. Tensfeldt and J. Montgomery Jr, *The Journal of chemical physics*, 1991, **94**, 6091–6101.
28. A. Klamt and G. Schüürmann, *Journal of the Chemical Society, Perkin Transactions 2*, 1993, 799–805.
29. M. Frisch, G. Trucks, H. Schlegel, G. Scuseria, M. Robb, J. Cheeseman, G. Scalmani, V. Barone, G. Petersson and H.

Nakatsuji, *Gaussian Inc. Wallingford CT*, 2016, **1**, 572.

30. C. Legault, *Sherbrooke, QC: http://www. cylview. org*, 2009.
